# A systematic review and meta-analysis of Zika virus epidemiology

**DOI:** 10.1038/s44360-025-00051-4

**Published:** 2026-02-13

**Authors:** Kelly McCain, Anna Vicco, Christian Morgenstern, Thomas Rawson, Tristan M. Naidoo, Sangeeta Bhatia, Dominic P. Dee, Patrick Doohan, Keith Fraser, Anna-Maria Hartner, Sequoia I. Leuba, Shazia Ruybal-Pesántez, Richard J. Sheppard, H. Juliette T. Unwin, Kelly Charniga, Zulma M. Cucunubá, Gina Cuomo-Dannenburg, Natsuko Imai-Eaton, Edward S. Knock, Adam Kucharski, Mantra Kusumgar, Paul Liétar, Rebecca K. Nash, Sabine van Elsland, Pathogen Epidemiology Review Group, Kelly McCain, Anna Vicco, Christian Morgenstern, Thomas Rawson, Sangeeta Bhatia, Patrick Doohan, Keith Fraser, H. Juliette T. Unwin, Kelly Charniga, Gina Cuomo-Dannenburg, Natsuko Imai-Eaton, Mantra Kusumgar, Paul Liétar, Aaron Morris, Alpha Forna, Amy Dighe, Anna-Maria Hartner, Anne Cori, Arran Hamlet, Ben Lambert, Bethan Cracknell Daniels, Charles Whittaker, Cosmo Santoni, Cyril Geismar, Dariya Nikitin, David Jorgensen, Dominic P. Dee, Edward S. Knock, Hayley Thompson, Isobel Routledge, Jack Wardle, Janetta Skarp, Joseph Hicks, Kanchan Parchani, Kieran Drake, Lily Geidelberg, Lorenzo Cattarino, Mara Kont, Marc Baguelin, Pablo N. Perez-Guzman, Paula Christen, Rebecca Nash, Richard Fitzjohn, Richard Sheppard, Rob Johnson, Sabine van Elsland, Sequoia I. Leuba, Shazia Ruybal-Pesántez, Sreejith Radhakrishnan, Tristan M. Naidoo, Zulma M. Cucunubá, Ruth McCabe, Ilaria Dorigatti, Nuno R. Faria, Anne Cori, Ruth McCabe, Ilaria Dorigatti

**Affiliations:** 1 MRC Centre for Global Infectious Disease Analysis & WHO Collaborating Centre for Infectious Disease Modelling, Jameel Institute, School of Public Health, Imperial College London, London, UK; 2 Centre for Artificial Intelligence in Public Health Research, Robert Koch Institute, Berlin, Germany; 3 London School of Hygiene and Tropical Medicine, London, UK; 4 Instituto de Microbiología, Universidad San Francisco de Quito, Quito, Ecuador; 5 School of Mathematics, University of Bristol, Bristol, UK; 6 Public Health Institute, Pontificia Universidad Javeriana, Bogotá, Colombia; 7 Health Protection Research Unit in Modelling and Economics, London, UK; 8 University of Oxford, Oxford, UK; 9 University of Georgia, Athens, GA USA; 10 Johns Hopkins University, Baltimore, MD USA; 11 PATH, Seattle, WA USA; 12 University of California, San Francisco (UCSF), Oakland, CA USA; 13 UK Health Security Agency (UKHSA), London, UK; 14 University of Glasgow, Glasgow, UK

**Keywords:** Viral infection, Epidemiology

## Abstract

Zika virus (ZIKV), classified as a priority pathogen by the World Health Organization, is an *Aedes*-borne arbovirus that can cause neurological complications and birth defects in newborns of mothers infected during pregnancy. We conducted a systematic review of peer-reviewed studies reporting ZIKV epidemiological parameters, transmission models and outbreaks (PROSPERO CRD42023393345) to characterize its transmissibility, seroprevalence, risk factors, disease sequelae and natural history. We performed meta-analyses of the proportions of congenital Zika syndrome, pregnancy loss among ZIKV-infected mothers and symptomatic cases. We extracted information from 574 studies. Across 418 included studies assigned a high-quality score, we extracted 969 parameters, 127 outbreak records and 154 models. Using random-effects models, we estimated proportions of congenital Zika syndrome (4.65%, 95% confidence interval (CI): 3.38–6.67%), pregnancy loss (2.48%, 95% CI: 1.62–3.78%) and symptomatic cases (51.20%, 95% CI: 38.00–64.23%). Seroprevalence estimates (*n* = 354) were retrieved beyond South America and French Polynesia. Basic reproduction number estimates (*n* = 77) ranged between 1.12 and 7.4. We found 66 human epidemiological delay estimates, including the intrinsic incubation period (*n* = 11, range: 4–12.1 days), infectious period (*n* = 15, range: 3–50 days), extrinsic incubation period (*n* = 22, range: 5.1–24.2 days) and serial interval (*n* = 27, range: 7.4–32.9 days).

These data are available in the R package ‘epireview’ (version 1.4.5). We provide a comprehensive systematic summary of ZIKV epidemiology, revealing large heterogeneities and inconsistencies in the reporting of parameter estimates, study designs and parameter definitions and underscoring the need for standardized epidemiological definitions.

## Abstract (web summary)

A systematic review including 574 studies extracts information about transmissibility, epidemiological delays and outbreaks for Zika virus disease at global scale.

## Main

Arboviruses pose a substantial threat to public health, with the World Health Organization (WHO) reporting close to 5.5 billion people at risk globally^1,2^. ZIKV is an arbovirus of concern that is mainly transmitted via *Aedes* mosquitoes but also human-to-human via sexual transmission^3^. Initially discovered in Uganda in 1947, ZIKV was only detected on the African and Asian continents until the mid-2000s^4^. The first outbreak of ZIKV outside these regions occurred in the Federated States of Micronesia in 2007 (ref. ^5^), with the virus subsequently spreading to the Americas^6,7^. An increase in cases of ZIKV, microcephaly, congenital Zika syndrome (CZS) and Guillain–Barré syndrome in Brazil in 2015 led to the declaration of a Public Health Emergency of International Concern (PHEIC) by the WHO from February to November 2016 (refs. ^8–10^).

While many ZIKV infections are asymptomatic^3,5,11^, the serious complications in the fetus and negative pregnancy outcomes associated with ZIKV infection during pregnancy, including CZS and its association with Guillain–Barré syndrome and other neurological disorders, underscore why future ZIKV re-emergence remains a global public health concern^10^. To date, no licensed vaccines or therapeutics exist, and although ZIKV cases have declined globally since 2017, they continue to be detected worldwide^12^. ZIKV remains listed among the pathogens with pandemic potential and is prioritized for research and development by the WHO^13,14^ and the UK Health Security Agency (UKHSA)^15^.

Mathematical models play a critical role in outbreak response and risk assessment by providing insights into the potential location, timing and magnitude of disease transmission. These tools can help guide the selection of sites for vaccine or therapeutic trials and inform the prepositioning of protocols and other preparedness activities. Central to these activities is the need for quantitative estimates of key epidemiological parameters, such as the reproduction number, epidemiological delays and seroprevalence, together with their variation and a catalogue of existing mathematical models. Here, we conducted a systematic review to generate a comprehensive database to aid the global modelling and public health community in their response to future ZIKV outbreaks. This systematic review is part of a set of reviews of nine WHO priority pathogens^16–19^ and contributes to a wider central, up-to-date and accessible database of information critical to mathematical modelling for timely outbreak response.

## Results

**Fig. 1:**
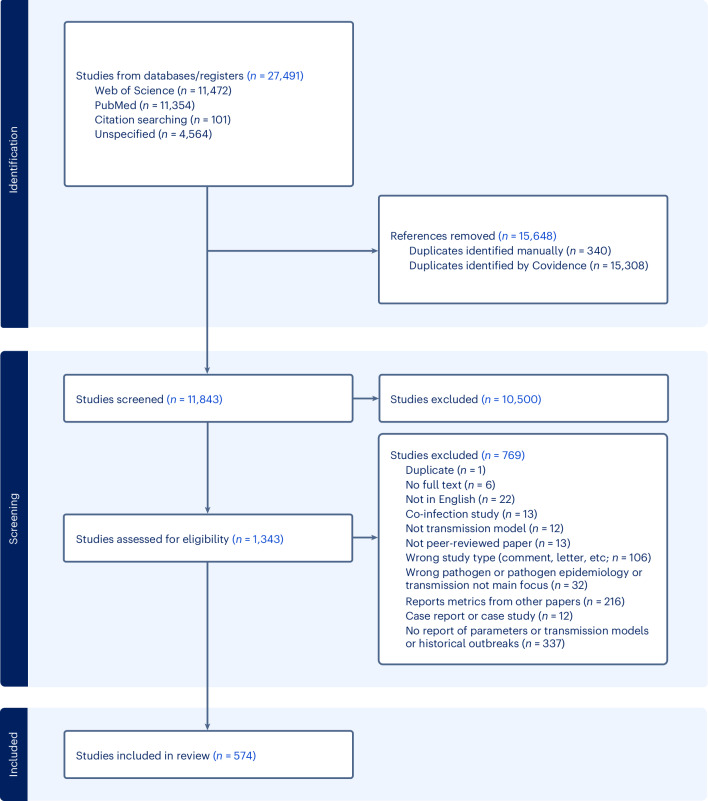
Flow chart for study selection. Studies were screened according to the Preferred Reporting Items for Systematic Reviews and Meta-Analyses (PRISMA) guidelines (Supplementary Information). Reasons for exclusion at the abstract or title stage were not collected by the Covidence software.

From the 27,491 studies identified through the database search, 11,845 studies were screened following de-duplication, of which 101 were identified through backwards citation screening of the systematic reviews. A total of 10,500 studies were excluded at the abstract/title screening stage, and we undertook full-text review of the remaining 1,343 studies, of which 574 met the criteria for inclusion (Fig. 1 and Supplementary Table A1). We extracted 159 outbreak records, 229 models and 1,334 parameters, of which 127 outbreak records, 154 models and 969 parameters belonged to publications that met the quality score ≥ 50% and are included in our main analysis. All extracted data and the full list of publications included in the main text of this review are available in the R package ‘epireview’ (version 1.4.5)^20^. The full analysis, including all publications, regardless of their quality assessment score, is provided in Supplementary Figs. B25–B28). Information about the extracted models is provided in Table B7 and Fig. B4.

### Outbreaks

**Fig. 2:**
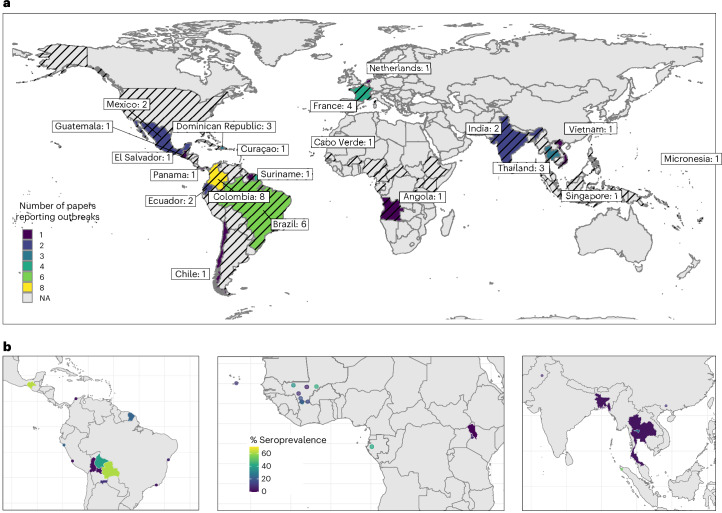
ZIKV outbreak and seroprevalence mapping. **a**, Countries with studies reporting ZIKV outbreak information, coloured by number of studies. Outbreaks reported in France and the Netherlands reflect outbreaks in overseas regions. Countries with black diagonal stripes indicate locations where ZIKV transmission has been reported by the WHO^78^. **b**, Geolocated areas or regions with ZIKV seroprevalence studies (using IgG assay, HAI/HI, MIA, NS1 BOB ELISA, IFA, capture ELISA and neutralization assays) conducted in the general population in the Americas (left), Africa (middle) and Asia (right). Each dot represents a location-specific estimate, while shaded areas indicate estimates at the administrative-unit level (region, province, district or entire country). Base maps adapted from GADM^79^. NA, not applicable.

Forty studies reported 127 outbreak records between 2006 (Thailand) and 2021 (India) from 29 countries across the Americas (*n* = 97), Oceania (*n* = 13), Asia (*n* = 7), Africa (*n* = 4) and Europe (*n* = 2; Fig. 2 and Supplementary Table B8). The largest number of records was reported in Colombia across several subregions (*n* = 55), followed by French Polynesia (*n* = 12) and Suriname (*n* = 11). The largest ZIKV outbreaks were reported in Puerto Rico between 2015 and 2016 (with 39,717 suspected cases, of which 36,390 were laboratory-confirmed) and in Colombia between 2015 and 2017 (with 108,087 suspected cases, 62% of which were among women, and 9,802 of which were laboratory-confirmed).

### Parameters

The 969 extracted parameter estimates came from 281 studies, comprising seroprevalence (*n* = 354), risk factors (*n* = 242), reproduction numbers (*n* = 117), CZS proportion (*n* = 53), epidemiological delays in humans (*n* = 66), attack rates (*n* = 52), extrinsic incubation period (*n* = 22), evolutionary rate (*n* = 15), substitution rate (*n* = 3), pregnancy loss proportion (*n* = 23), case fatality ratio (CFR; *n* = 6), proportion of symptomatic cases (*n* = 10), relative contributions to transmission (*n* = 2) and growth rates (*n* = 4) (Supplementary Fig. B3).

### Seroprevalence

A total of 139 studies reported 354 ZIKV seroprevalence estimates from serosurveys conducted between 1980 (Nigeria^21^) and 2022 (El Salvador^22^) in 59 countries across the Americas (*n* = 143), Africa (*n* = 97), Asia (*n* = 62), Europe (*n* = 36) and Oceania (*n* = 16). All seroprevalence estimates extracted in this study are reported in Supplementary Table B10. Almost one-third of these estimates were from surveys of the general population (*n* = 107), while the rest were from pregnant women (*n* = 58), suspected ZIKV infection (*n* = 64), children (*n* = 35), mixed population groups (*n* = 35), blood donors (*n* = 10) and other at-risk populations (*n* = 43) (Supplementary Figs. B9 and B10). The timing of serosurveys coincided with the major epidemics (Supplementary Figs. B9 and B10). The majority of ZIKV seroprevalence estimates were derived from immunoglobulin G (IgG) assays (*n* = 139) and neutralization tests (*n* = 109; Supplementary Fig. B11).

Seroprevalence estimates varied widely from 100% in a hospital-based study of 29 individuals in Cúcuta, Colombia^23^, to 0% in local regions in Austria^24^, Iran^25^ and Taiwan^26^, and specific locations within Brazil^27,28^, Bolivia^29^, Kenya^30^, Indonesia^31^ and Thailand^32^. Within Brazil, ZIKV exposure in Salvador (Bahia) ranged between 7% (95% CI: 4–10%) in 2014 and 63% (95% CI: 60–65%) in 2015 (ref. ^33^), consistent with the timing of the major outbreak in the Americas. In contrast, ZIKV seroprevalence in Rio de Janeiro was reported to be 3% in 2018 (ref. ^34^). In Asia, a national serosurvey conducted in Thailand during 2017–2020 yielded an average seroprevalence of 2.8%^35^, but estimates were as high as 23.5% from a serosurvey conducted in 2011–2012 in Nakhon Sawan city^36^. In Africa, serosurveys conducted from 2013 to 2017 reported ZIKV seroprevalence less than 11.5% in several sites of Kenya^30^, 42.1% in Gabon^37^, 10.9% in Cabo Verde^38^ and ranging from 3.1% to 43.3% in Mali^25^.

### Transmissibility

We extracted estimates of the basic reproduction number *R*_0_ (*n* = 77), human-to-human (sexual) ${R}_{0}^{\rm{h}}$ (*n* = 14) and vector-borne ${R}_{0}^{\rm{v}}$ (*n* = 9), effective reproduction number *R*_e_ (*n* = 15) and sexual ${R}_{\rm{e}}^{\rm{h}}$ (*n* = 2) from 40 studies. The methods used to estimate *R*_0_ and *R*_e_ varied across the different studies. Compartmental models were used most frequently (*n* = 45), and among these, the next-generation matrix method was used for 27 estimates. Branching processes were used for 16 estimates. Most estimates were from the 2013–2014 outbreaks in French Polynesia (*n* = 24), and the 2015–2016 epidemics in Brazil (*n* = 26) and Colombia (*n* = 15).

**Fig. 3:**
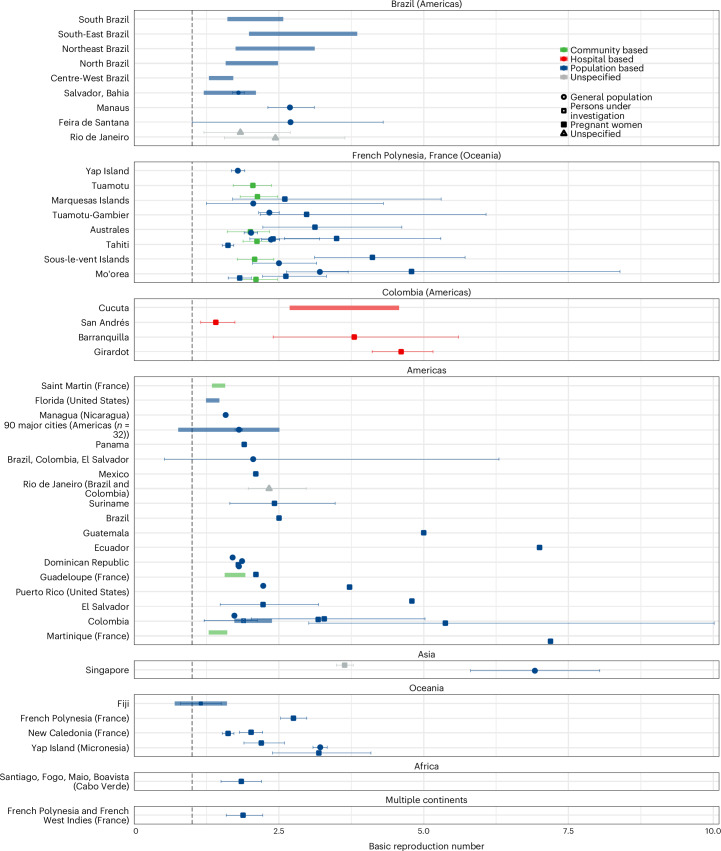
ZIKV basic reproduction number estimates. *R*_0_ estimates by geographic location, type of sample and population group. Sub-national estimates for Brazil, Colombia and French Polynesia are shown in separate panels because of the high number of estimates; all other estimates are shown in the respective continent panels. Overall country estimates for Brazil, Colombia and French Polynesia are shown in the corresponding continent panels. Points are central estimates reported in the published studies (defined as mean, median or unspecified central as estimated in the extracted paper), error bars are 95% confidence or credible intervals, and thicker shaded bars are ranges of central estimates over disaggregated groups. The grey vertical dashed line marks 1. When multiple estimates for the same location were available, the estimates were jittered. The sample sizes for each study shown in the figure are reported in Supplementary Table B12.

The majority (63/77) of the central estimates of *R*_0_ were between 1.5 and 4 (Fig. 3). Estimates of the other transmission type-specific reproduction numbers are shown in Supplementary Figs. B5 and B7 and Table B12).

### Epidemiological delays

**Fig. 4:**
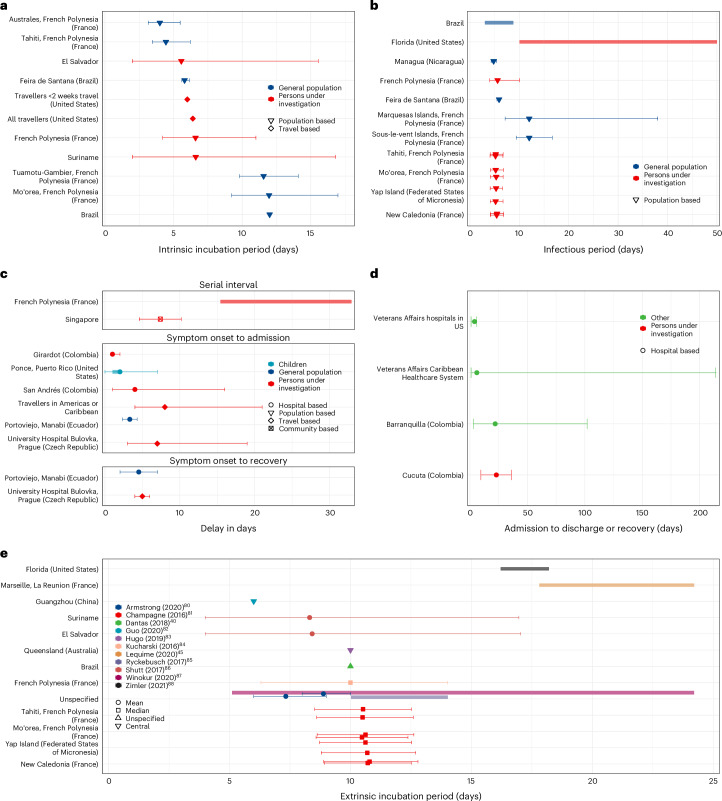
Epidemiological delays in human and mosquitoes. **a**–**e**, Estimates by location and type of sample of Zika intrinsic incubation period (**a**), infectious period (**b**), serial interval, symptom onset to admission and symptom onset to recovery (**c**) and admission to discharge or recovery (**d**). Estimates by location and study of ZIKV extrinsic incubation period in the mosquito (**e**). Points are the central estimates reported in the studies (defined as mean, median or unspecified central estimates as shown in the extracted paper), error bars are 95% confidence or credible intervals, and shaded bars are ranges of central estimates over disaggregated groups. When multiple estimates for the same location were available, the estimates were jittered. The sample sizes for each study shown in the figure are reported in Supplementary Table B9.

We analysed 66 human epidemiological delays from 29 studies and 22 extrinsic incubation period estimates from 12 studies (Fig. 4). The 11 central intrinsic incubation period estimates ranged from 4 days (95% CI: 3.15–5.48) in Australes, French Polynesia in 2013–2014 (ref. ^39^) to 12.1 days in Brazil in 2016 (ref. ^40^), although roughly half of the reported estimates (*n* = 6/11) were between 5 and 7 days. The 15 estimates of the human infectious period were highly variable, ranging from central estimates of 3 days in Brazil in 2016 (ref. ^40^) to 50 days among asymptomatic cases in Florida, United States^41^ in July to September 2016. We extracted only two estimates from two studies of the serial interval: a mean of 7.4 days (95% CI: 4.59–10.2) in Singapore in August to November 2016 (ref. ^42^) and of 32.9 days in French Polynesia between October 2013 and October 2016 (ref. ^43^). The single extracted estimate for the generation time was estimated from data collected in the French West Indies (Guadeloupe, Martinique and Saint-Martin) in 2015–2017 and was described by a mean of 2.5 weeks (s.d. 0.7).

The 22 central estimates of the extrinsic incubation period ranged from 5.1 (ref. ^44^) to 24.2 days^44,45^. The central estimates of the time from symptom onset to admission to care ranged from 1 (interquartile range (IQR) 1–2) day among hospitalized patients in Colombia^46^ to 8 (IQR 4–21) days among travellers^47^. The central estimates of time from admission to recovery or discharge ranged from 4 (ref. ^48^) to 23 (IQR 9.5–36)^23^ days. All epidemiological delays extracted in this study are listed in Supplementary Table B9.

### Severity and adverse birth outcomes

**Fig. 5:**
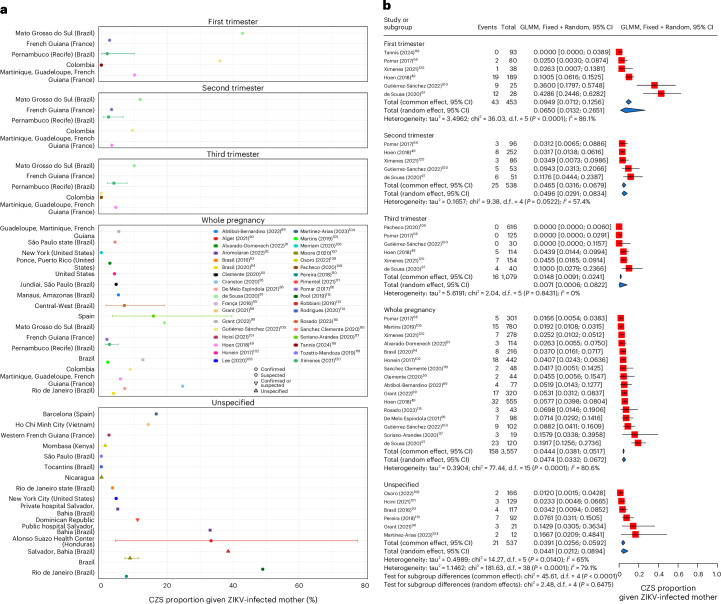
Proportion of CZS. **a**, Estimates of reported CZS probabilities by pregnancy stage of the infected mother of an infant with CZS. ‘Unspecified’ is used when no specific pregnancy stage was reported. Points are central estimates (defined as mean, median or unspecified central values as estimated in the extracted paper), solid lines are confidence or credible intervals, and shaded segments are ranges of central estimates across disaggregated groups. **b**, Meta-analysis of CZS risk stratified by pregnancy stage of the infected mother of an infant with CZS. ‘Unspecified’ is used when no specific pregnancy stage was reported. We used generalized linear mixed-effect model (GLMM) estimates of CZS proportion. Red squares represent the observed study effect sizes, and the solid black horizontal lines are CIs. Blue diamonds represent pooled common and random-effect estimates by trimester and overall. Common effect estimates assume that all aggregated data come from a single data-generating process with one common CZS proportion, and overall random-effect estimates allow the CZS proportion to vary by study and give different weights to each study in the overall estimate. The ‘Events’ column indicates the reported number of CZS cases. The sample sizes for each study shown in the figure are reported in Supplementary Table B16. Statistical analyses were performed using the chi-squared test for heterogeneity, with two-sided significance. Between-study variance was estimated using tau², and *I*² indicates the proportion of total variation due to heterogeneity rather than chance. Figures report the *P* value for each group, rounding to <0.0001 when values fall below this threshold in the meta-analysis.

We extracted 53 estimates of CZS proportion given a ZIKV-infected mother from 36 studies. The CZS proportion was highly variable across studies (Fig. 5a), 92.5% (49/53) of estimates were from the Americas, and all estimates were obtained from data collected between January 2015 and February 2020.

Among the 53 estimates, 39 estimates from 24 studies were eligible for meta-analysis based on our stringent meta-analysis inclusion criteria (see ‘Data analysis’ in the Methods). The pooled CZS proportion, restricted to non-trimester-specific estimates, was 4.65% (95% CI: 3.38–6.37%) using the random-effects model (*I*^2^ = 77.10%; Supplementary Fig. B33). Stratifying the meta-analysis by trimester of maternal ZIKV exposure showed a higher pooled estimate of CZS proportion in the first trimester (6.50%, 95% CI: 1.31–26.51%, *I*^2^ = 86.10%) compared to the second (4.96%, 95% CI: 2.91–8.34%, *I*^2^ = 57.40%) or third (0.71%, 95% CI: 0.06–8.22%, *I*^2^ = 0.00%) trimester (Fig. 5b). Sensitivity analyses of the CZS proportion by continent showed that the estimate for the Americas (4.43%, 95% CI: 3.21–6.07%, *I*^2^ = 78.10%) was lower than that from travel-based studies in Europe (16.13%, 95% CI: 6.88–33.37%, *I*^2^ = 0.00%); Supplementary Fig. B31). The random-effects estimate for Brazil (5.80%, 95% CI: 3.74–8.91%, *I*^2^ = 85.20%) was marginally higher than the overall estimate (Supplementary Fig. B32).

We extracted 23 central estimates of the proportion of pregnancy loss associated with maternal ZIKV infection, which were derived from 17 studies and ranged from 0%^49–51^ to 25%^52^ (Supplementary Fig. B14). Of these, we included only 14 studies (20 estimates) in the meta-analysis because of our more stringent meta-analysis inclusion criteria (see ‘Data analysis’ in the Methods). The pooled overall estimates of the proportion of pregnancy loss were 2.48% (95% CI: 1.62–3.78%, *I*^2^ = 91.10%) using the random-effects model (Supplementary Fig. B33). When we stratified based on the time of pregnancy loss, the pooled estimate of miscarriage proportion (2.04%, 95% CI: 1.21–3.43%, *I*^2^ = 64.70%) was marginally lower than the pooled estimate for general pregnancy loss (3.16%, 95% CI: 1.76–5.61%, *I*^2^ = 95.40%); Supplementary Fig. B34).

The CFR estimates (Supplementary Fig. B15 and Table B14) extracted from five studies conducted between 2015 and 2017 in the Dominican Republic^53^, the West Indies^54^, Brazil^55,56^ and Colombia^57^ varied from 0.04% reported in a population-based study of 5,161 individuals in the Dominican Republic in 2016 (ref. ^53^), to 38% in a small hospital-based cohort of 16 persons under investigation in Brazil in 2016 (ref. ^56^). The proportion of symptomatic individuals (Supplementary Fig. B16 and Table B14) was reported in three studies, yielding ten estimates ranging from 23.2% in pregnant women in French Guiana^58^ to 71% (95% CI: 66–76%) in children in the Society Islands (French Polynesia)^59^. Of the three studies, two had sample size information and thus were included in our meta-analysis, which showed a pooled estimate of the proportion of symptomatic cases of 51.20% (95% CI: 38.00–64.23%, *I*^2^ = 96.70%; Supplementary Figs. B35 and B36).

## Discussion

In this systematic review, we collated and analysed epidemiological parameters, transmission models and outbreak records of ZIKV to inform future modelling in response to ZIKV outbreaks. One of the clearest findings of this review is the concentration of Zika-related publications within a narrow time window—from the 2015 outbreak in Brazil to the onset of the coronavirus disease 2019 pandemic in 2020. The 2015–2016 ZIKV epidemic in Latin America marked a turning point in ZIKV research: before this, only 6 of the 574 studies included in this review were published, none of which involved modelling. With the emergence of ZIKV as a global health concern, research activity surged, particularly following the outbreak in Brazil in 2015 and the subsequent PHEIC declaration, resulting in a sharp increase in publications (often with a quality assessment score < 50%; Supplementary Fig. B2). Since 2020, however, the volume of new studies has declined despite continued ZIKV circulation, likely due to reduced data availability and shifts in funding priorities after the end of the PHEIC.

Among the 354 estimates characterizing ZIKV exposure through seroprevalence included in this review, there is a marked heterogeneity in geographic representation, with the Americas accounting for the majority of the published seroprevalence estimates. While these statistics reflect the attention that ZIKV received during the 2015–2016 epidemic in the Americas, they also underscore gaps in our understanding of the population-level immunity of populations outside these regions, which is critical to evaluate global outbreak risks now and in the future, and to design clinical trials for future vaccine and therapeutics. Seroprevalence estimates should also be interpreted with caution, as variations in assay sensitivity, specificity and cross-reactivity—especially with other flaviviruses like dengue—could bias the results. Our analysis has demonstrated that ZIKV is moderately to highly transmissible (*R*₀ typically 1.5–4, up to >7), with both vector-borne and sexual transmission contributing to infection. In combination with a pooled CZS proportion of 4.65% and a pooled pregnancy loss proportion of 2.48%, continued monitoring is essential to track outbreak potential and protect vulnerable populations. Moreover, our meta-analysis on the proportion of ZIKV-infected symptomatic cases suggests that up to half of ZIKV infections may go undetected in surveillance systems that rely on symptomatic reporting (51.20%, 95% CI: 38.00–64.23%, *I*^2^ = 96.70%).

Estimates of CZS proportion vary widely due to differences in study design (that is, recruitment based on confirmed infection versus retrospective serology), population characteristics, case definitions and methods used for the identification of ZIKV infections during pregnancy. Several studies from which we extracted CZS proportion were not specifically designed to assess the CZS proportion in recently infected mothers (that is, studies tested for past exposure by IgG assays rather than recent infections through IgM detection). Our meta-analysis produced an overall estimate of 4.65% (95% CI: 3.38–6.37%, *I*^2^ = 77.10%), which is slightly higher than other published estimates^60–63^, although the CIs overlap. We also found only one published review that conducted a trimester-specific meta-analysis of the proportion of CZS^64^ including only two studies^65,66^. Our meta-analysis of CZS proportion by trimester, based on seven studies, yielded higher estimates than those published in Gallo et al.^64^, with consistently higher estimates in earlier trimesters.

Although we extracted 969 parameters from the literature, it was not possible to perform meta-analyses across most parameters (other than CZS, pregnancy loss probabilities and proportion of symptomatic cases) due to low numbers of comparable estimates, unavailability of sample size information and substantial heterogeneity in study contexts and methodologies. For example, only one estimate of the generation time and two estimates of the serial interval have been published. We also noted substantial variation in the terminology and methods used for estimation in studies reporting attack rates and reproduction number estimates, where basic and effective reproduction numbers were occasionally defined interchangeably, and in studies reporting genomic information, where mutation rates (per generation) and evolutionary or substitution rates (per year) were often conflated.

This study has limitations. We included only peer-reviewed publications in English, which may have led to the exclusion of relevant studies published in other languages and in grey literature or outbreak reports. Studies reporting outbreak dynamics or seroprevalence estimates that did not meet the formal inclusion criteria were excluded^67–71^; hence, the outbreak data reported in this systematic review should not be considered a complete compendium, as several sources of ZIKV outbreaks exist outside peer-reviewed publications, specifically PAHO^72^ and national Ministries of Health. Moreover, while we attempted to extract disaggregated parameter estimates where available, for feasibility purposes, we prioritized summarizing data over fully disaggregated information (for example, by age or sex; Supplementary Information). All information is, however, included in our open-source database (also in Supplementary Tables B7–B16) that is accessible via the R package ‘epireview’ to support future reuse. Finally, data extraction was conducted by a team of 16 reviewers using detailed, standardized guidelines^73^. Although double extractions were conducted at early stages, a residual risk of inconsistency and human error remains.

Despite these limitations, this study has several strengths as it summarizes the information published in 418 studies on 984 epidemiological parameters, 154 models and 127 outbreak records, which provides the most thorough quantitative overview of ZIKV epidemiology and disease risk available to date. As well as documenting key epidemiological parameters needed for outbreak response and preparedness planning, the richness of the data collated in this systematic review allows us to provide global, regional and country-level estimates of the proportion of adverse effects of ZIKV, including CZS, pregnancy loss and symptomatic cases.

In summary, this study presents a dynamic, open-source resource designed to support continuous updates by the research community to provide timely quantitative information on ZIKV epidemiology. By enabling the development of data-driven and evidence-based analytical, modelling and computational tools, this resource will aid mathematical modellers, public health officials and policymakers in outbreak prevention, preparedness, response and disease control planning.

## Methods

We followed the PRISMA guidelines and registered our study protocol with PROSPERO (International Prospective Register of Systematic Reviews, CRD42023393345). The checklist is included in the Supplementary Information.

### Search and screening

We searched PubMed and Web of Science for studies published from database inception to 31 October 2024. Results were imported into Covidence^74^ and de-duplicated. Titles, abstracts and then full texts were independently screened by two reviewers, and conflicts were resolved by consensus. The Cohen’s Kappa scores for screening and full-text review are reported in Supplementary Fig. A1. Non-peer-reviewed literature and non-English language studies were excluded. We also excluded papers with co-infection studies, comments, letters, case reports, case studies and those that reported estimates from other papers. Systematic reviews (*n* = 66) were excluded from extraction (Supplementary Information Section A), but peer-reviewed literature identified through backwards citation screening meeting the selection criteria was included. Further details on inclusion and exclusion criteria are provided in the Supplementary Information.

### Data extraction

Of the full texts meeting the inclusion criteria, 16% (*n* = 91) were randomly selected for double extraction to ensure concordance between a team of 16 extractors. After consensus was reached, reviewers independently conducted single extractions on the remaining full texts.

Data were extracted using a custom-made Microsoft Access database (version 2305; Supplementary Tables A4–A6). We collected information on publication details and three categories: transmission models, outbreaks and epidemiological parameters. In the transmission model category, we extracted information on the mathematical or statistical models of transmission used in the included papers. In the outbreak category, we reported outbreak data only when it was newly observed, rather than derived from another publication. Finally, the epidemiological parameters included were basic and effective reproduction numbers, epidemiological delays (the intrinsic incubation period, the extrinsic incubation period, the time between symptom onset to hospitalization or from symptom onset to outcome), CFRs, attack rates, growth rates, evolutionary, mutation and substitution rates, the proportion of symptomatic cases, overdispersion (quantifying heterogeneity in transmission), seroprevalence, proportion of CZS/microcephaly in newborns born to infected mothers, proportion of pregnancy loss in infected pregnant women, relative contributions to transmission from different routes and risk factors related to Zika or CZS. Pregnancy loss proportion was extracted as either the proportion of miscarriage if the study explicitly reported this, or general pregnancy loss, which included both the sum of miscarriage and stillbirth and pregnancy loss with no specified information on the trimester of loss.

We extracted estimates of the basic and effective reproduction numbers (${R}_{0}$ and ${R}_{e}$, representing the average number of secondary infections generated by a case in a fully susceptible and partially susceptible population, respectively), the basic vector-borne reproduction number (${R}_{0}^{\rm{v}}$, representing the average number of new infections generated via vector-borne transmission in a fully susceptible population) and the basic and effective sexual reproduction numbers (${R}_{0}^{\rm{h}}$ and ${R}_{e}^{\rm{h}}$, representing the average number of new cases generated via sexual transmission in a fully susceptible and partially susceptible population, respectively).

We used a custom quality assessment tool to assess the quality of each study (Supplementary Table A2). Full details on the data extraction process and extracted data are provided in the Supplementary Information.

### Data analysis

The analysis was conducted in R software using the ‘orderly2’ workflow package (version 1.99.14)^75^. The quality score of each study was calculated as the proportion of ‘yes’ answers to the total number of applicable questions in the quality assessment tool for that study. Only parameters with a quality score ≥ 50% were included in the analyses presented in the main text. Sensitivity analyses including all studies, regardless of the quality assessment score, are presented in Supplementary Information Section B4.

We conducted meta-analyses for the proportion of symptomatic cases, proportion of CZS and proportion of pregnancy loss among confirmed ZIKV-infected mothers. For the latter two, we applied more meta-analysis inclusion criteria by only including estimates with at least ten pregnant women with confirmed ZIKV infection and with a study design that did not select for the outcome (CZS or miscarriage), using the ‘meta’ R package (version 8.2-1)^76^. We used random-effect and fixed-effect models using the logit link function to calculate pooled estimates for both parameters, with 95% CIs and *I*^2^ heterogeneity estimates. We report the random-effects estimates in the paper, and the fixed-effect estimate is reported in meta-analysis plots (Fig. 5 and Supplementary Information Section B5). We estimated the pooled CZS and pregnancy loss probabilities by country, continent and sample population type using maximum likelihood to estimate the between-studies variance. Subgroup analyses were considered to assess differences in CZS and pregnancy loss probabilities. Funnel plots assessing publication bias are shown in Supplementary Figs. B29 and B30.

### Reporting summary

Further information on research design is available in the Nature Portfolio Reporting Summary linked to this article.

## Supplementary information

Supplementary Information Supplementary Methods, Figs. A1–B36 and Tables A1–C19.

Reporting Summary

Peer Review File

## Data Availability

Data are publicly available from https://github.com/mrc-ide/epireview/.

## Code availability

Code and a vignette to reproduce the analysis are available from https://github.com/mrc-ide/priority-pathogens and https://mrc-ide.github.io/priority-pathogens/articles/pathogen_zika.html. All analyses were performed in R (version 4.5.0) with the following packages: epireview (1.4.5), orderly2 (1.99.14), prioritypathogens (https://github.com/mrc-ide/priority-pathogens/tree/zika_v2 or via Zenodo at 10.5281/zenodo.17201039 (ref. ^77^)), metafor (4.8-0), meta (8.2-1), estmeansd (1.0.1), mixdist (0.5-5), ggplot2 (4.0.0), ggsci (3.2.0), sf (1.0-21), ragg (1.4.0), ggspatial (1.1.10), ggforce (0.5.0), png (0.1-8), grid (4.5.1), patchwork (1.3.2), gridExtra (2.3), readxl (1.4.5), harrypotter (2.1.1), rnaturalearth (1.1.0), rnaturalearthdata (1.0.0), ggrepel (0.9.6), gt (1.0.0), scales (1.4.0), gpkg (0.0.12), RSQLite (2.4.3), countrycode (1.6.1), ggpattern (1.2.1), tidyverse (2.0.0), stringr (1.5.2), maps (3.4.3) and tidygeocoder (1.0.6). An archived version of the code used for the analysis is available via Zenodo at 10.5281/zenodo.17201039 (ref. ^77^).

## References

[1] Global arbovirus initiative. *World Health Organization* https://iris.who.int/server/api/core/bitstreams/2a55ec8b-bbf5-46c9-a5bb-696f15196d49/content (2025).

[2] Lim, A. et al. The overlapping global distribution of dengue, chikungunya, Zika and yellow fever. *Nat. Commun.* **16**, 3418 (2025). 40210848 10.1038/s41467-025-58609-5 PMC11986131

[3] Zika virus disease. *World Health Organization* https://www.who.int/health-topics/zika-virus-disease/ (2025).

[4] The history of Zika virus. *World Health Organization* https://www.who.int/news-room/feature-stories/detail/the-history-of-zika-virus/ (2016).

[5] Duffy, M. R. et al. Zika virus outbreak on Yap Island, Federated States of Micronesia. *N. Engl. J. Med.* **360**, 2536–2543 (2009). 19516034 10.1056/NEJMoa0805715

[6] Zika virus. *World Health Organization* https://www.who.int/news-room/fact-sheets/detail/zika-virus/ (2025).

[7] Faria, N. R. et al. Zika virus in the Americas: early epidemiological and genetic findings. *Science* **352**, 345–349 (2016). 27013429 10.1126/science.aaf5036 PMC4918795

[8] Waddell, L. A. & Greig, J. D. Scoping review of the Zika virus literature. *PLoS ONE* **11**, e0156376 (2016). 27244249 10.1371/journal.pone.0156376 PMC4887023

[9] Musso, D., Ko, A. I. & Baud, D. Zika virus infection—after the pandemic. *N. Engl. J. Med.* **381**, 1444–1457 (2019). 31597021 10.1056/NEJMra1808246

[10] *WHO statement on the first meeting of the International Health Regulations (2005) (IHR 2005) Emergency Committee on Zika virus and observed increase in neurological disorders and neonatal malformations* (World Health Organization, 2016) https://www.who.int/news/item/01-02-2016-who-statement-on-the-first-meeting-of-the-international-health-regulations-(2005)-(ihr-2005)-emergency-committee-on-zika-virus-and-observed-increase-in-neurological-disorders-and-neonatal-malformations/

[11] Haby, M. M., Pinart, M., Elias, V. & Reveiz, L. Prevalence of asymptomatic Zika virus infection: a systematic review. *Bull. World Health Organ.* **96**, 402–413D (2018). 29904223 10.2471/BLT.17.201541 PMC5996208

[12] Zika virus disease contracted during travel: likely places of infection reported by travellers to the EU/EEA. *European Centre for Disease Control* https://www.ecdc.europa.eu/en/zika-virus-infection/surveillance-and-disease-data/travel-associated-cases/ (2025).

[13] Pathogens prioritization: a scientific framework for epidemic and pandemic research preparedness. *World Health Organization* https://www.who.int/publications/m/item/pathogens-prioritization-a-scientific-framework-for-epidemic-and-pandemic-research-preparedness/ (2024).

[14] *2018 Annual review of diseases prioritized under the Research and Development Blueprint* (World Health Organization, 2018) https://www.who.int/docs/default-source/blue-print/2018-annual-review-of-diseases-prioritized-under-the-research-and-development-blueprint.pdf

[15] Priority pathogen families research and development (R&D) tool. *UK Health Security Agency* https://assets.publishing.service.gov.uk/media/67f5259ae3c60873d6c90d08/UKHSA-priority-pathogen-families-research-and-development-tool.pdf (2025).

[16] Cuomo-Dannenburg, G. et al. Marburg virus disease outbreaks, mathematical models, and disease parameters: a systematic review. *Lancet Infect. Dis.* **24**, e307–e317 (2024). 38040006 10.1016/S1473-3099(23)00515-7 PMC7615873

[17] Nash, R. K. et al. Ebola virus disease mathematical models and epidemiological parameters: a systematic review. *Lancet Infect. Dis.*e762–e773 (2024). 10.1016/S1473-3099(24)00374-8 PMC7616620 39127058

[18] Doohan, P. et al. Lassa fever outbreaks, mathematical models, and disease parameters: a systematic review and meta-analysis. *Lancet Glob. Health* **12**, e1962–e1972 (2024). 39577970 10.1016/S2214-109X(24)00379-6

[19] Morgenstern, C. et al. Severe acute respiratory syndrome (SARS) mathematical models and disease parameters: a systematic review. *Lancet Microbe* **6**, 101144 (2025). 40713974 10.1016/j.lanmic.2025.101144

[20] Naidoo, T. et al. epireview: tools to update and summarise the latest pathogen data from the Pathogen Epidemiology Review Group (PERG). R package version 1.4.4 https://github.com/mrc-ide/epireview (2025).

[21] Adekolu-John, E. O. & Fagbami, A. H. Arthropod-borne virus antibodies in sera of residents of Kainji Lake Basin, Nigeria 1980. *Trans. R. Soc. Trop. Med. Hyg.* **77**, 149–151 (1983). 6306872 10.1016/0035-9203(83)90053-6

[22] Lynn, M. K. et al. Perinatal dengue and Zika virus cross-sectional seroprevalence and maternal-fetal outcomes among El Salvadoran women presenting for labor-and-delivery. *Matern. Health Neonatol. Perinatol.* **10**, 7 (2024). 38561854 10.1186/s40748-024-00177-5 PMC10985905

[23] Anaya, J. M. et al. A comprehensive analysis and immunobiology of autoimmune neurological syndromes during the Zika virus outbreak in Cucuta, Colombia. *J. Autoimmun.* **77**, 123–138 (2017). 28062188 10.1016/j.jaut.2016.12.007

[24] Borena, W. et al. No molecular or serological evidence of Zikavirus infection among healthy blood donors living in or travelling to regions where *Aedes albopictus* circulates. *PLoS ONE* **12**, e0178175 (2017). 28542611 10.1371/journal.pone.0178175 PMC5443526

[25] Ziyaeyan, M. et al. Widespread circulation of West Nile virus, but not Zika virus in southern Iran. *PLoS Negl. Trop. Dis.* **12**, e0007022 (2018). 30557321 10.1371/journal.pntd.0007022 PMC6312345

[26] Chien, Y. et al. Low seroprevalence of Zika virus infection among adults in Southern Taiwan. *BMC Infect. Dis.*10.1186/s12879-019-4491-4 (2019). 10.1186/s12879-019-4491-4 PMC6813068 31646973

[27] Alves, L., Leal, C. & Alves, J. Zika virus seroprevalence in women who gave birth during Zika virus outbreak in Brazil - a prospective observational study. *Heliyon*10.1016/j.heliyon.2020.e04817 (2020). 10.1016/j.heliyon.2020.e04817 PMC7490532 32964154

[28] Souza Gonçalves, M. et al. Prevalence of arboviruses in sickle cell disease patients from two different regions of Brazil, the North and Northeast. *Braz. J. Infect. Dis.* **28**, 103741 (2024). 38670165 10.1016/j.bjid.2024.103741 PMC11070587

[29] Saba Villarroel, P. M. et al. Zika virus epidemiology in Bolivia: a seroprevalence study in volunteer blood donors. *PLoS Negl. Trop. Dis.* **12**, e0006239 (2018). 29513667 10.1371/journal.pntd.0006239 PMC5858838

[30] Chepkorir, E. et al. Serological evidence of *Flavivirus* circulation in human populations in Northern Kenya: an assessment of disease risk 2016–2017. *Virol. J.*10.1186/s12985-019-1176-y (2019). 10.1186/s12985-019-1176-y PMC6525424 31101058

[31] Putri, N. D. et al. Absence of evidence of Zika virus infection in cord blood and urine from newborns with congenital abnormalities, Indonesia. *Am. J. Trop. Med. Hyg.* **102**, 876–879 (2020). 32043460 10.4269/ajtmh.19-0593 PMC7124925

[32] Ngo-Giang-Huong, N. et al. Lack of association between adverse pregnancy outcomes and Zika antibodies among pregnant women in Thailand between 1997 and 2015. *Viruses*10.3390/v13081423 (2021). 10.3390/v13081423 PMC8402824 34452289

[33] Rodriguez-Barraquer, I. et al. Impact of preexisting dengue immunity on Zika virus emergence in a dengue endemic region. *Science* **363**, 607–610 (2019). 30733412 10.1126/science.aav6618 PMC8221194

[34] Périssé, A. R. S. et al. Zika, dengue and chikungunya population prevalence in Rio de Janeiro city, Brazil, and the importance of seroprevalence studies to estimate the real number of infected individuals. *PLoS ONE* **15**, e0243239 (2020). 33332373 10.1371/journal.pone.0243239 PMC7746276

[35] Kitro, A. et al. Seroprevalence of dengue, Japanese encephalitis and Zika among long-term expatriates in Thailand. *J. Travel Med.*10.1093/jtm/taae022 (2024). 10.1093/jtm/taae022 38335250

[36] Yamanaka, A. et al. Seroprevalence of *Flavivirus* neutralizing antibodies in Thailand by High-throughput neutralization assay: endemic circulation of Zika virus before 2012. *mSphere*10.1128/mSphere.00339-21 (2021). 10.1128/mSphere.00339-21 PMC8386448 34259560

[37] Ushijima, Y. et al. Surveillance of the major pathogenic arboviruses of public health concern in Gabon, Central Africa: increased risk of West Nile virus and dengue virus infections. *BMC Infect. Dis.* **21**, 265 (2021). 33731022 10.1186/s12879-021-05960-9 PMC7966894

[38] Ward, D. et al. Sero-epidemiological study of arbovirus infection following the 2015–2016 Zika virus outbreak in Cabo Verde. *Sci. Rep.*10.1038/s41598-022-16115-4 (2022). 10.1038/s41598-022-16115-4 PMC9271056 35810191

[39] Rahman, M., Bekele-Maxwell, K., Cates, L., Banks, H. & Vaidya, N. Modeling Zika virus transmission dynamics: parameter estimates, disease characteristics, and prevention. *Sci. Rep.*10.1038/s41598-019-46218-4 (2019). 10.1038/s41598-019-46218-4 PMC6646355 31332269

[40] Dantas, E., Tosin, M. & Cunha, A. Calibration of a SEIR-SEI epidemic model to describe the Zika virus outbreak in Brazil. *Appl. Math Comput.* **338**, 249–259 (2018).

[41] Tuncer, N., Marctheva, M., LaBarre, B. & Payoute, S. Structural and practical identifiability analysis of Zika epidemiological models. *Bull. Math Biol.* **80**, 2209–2241 (2018). 29948883 10.1007/s11538-018-0453-z

[42] Singapore Zika Study Group. Outbreak of Zika virus infection in Singapore: an epidemiological, entomological, virological, and clinical analysis. *Lancet. Infect. Dis.* **17**, 813–821 (2017). 10.1016/S1473-3099(17)30249-9 28527892

[43] Riou, J., Poletto, C. & Boelle, P. Y. A comparative analysis of Chikungunya and Zika transmission. *Epidemics* **19**, 43–52 (2017). 28139388 10.1016/j.epidem.2017.01.001

[44] Winokur, O., Main, B., Nicholson, J. & Barker, C. Impact of temperature on the extrinsic incubation period of Zika virus in *Aedes aegypti*. *PLoS Negl. Trop. Dis.*10.1371/journal.pntd.0008047 (2020). 10.1371/journal.pntd.0008047 PMC7105136 32187187

[45] Lequime, S. et al. Modeling intra-mosquito dynamics of Zika virus and its dose-dependence confirms the low epidemic potential of *Aedes albopictus*. *PLOS Pathog***.**10.1371/journal.ppat.1009068 (2020). 10.1371/journal.ppat.1009068 PMC7774846 33382858

[46] Rojas, D. P. et al. The epidemiology and transmissibility of Zika virus in Girardot and San Andres island, Colombia, September 2015 to January 2016. *Euro Surveill.*10.2807/1560-7917.es.2016.21.28.30283 (2016). 10.2807/1560-7917.ES.2016.21.28.30283 PMC5124348 27452806

[47] Angelo, K. et al. Zika among international travellers presenting to GeoSentinel sites, 2012–2019: implications for clinical practice. *J. Travel Med.*10.1093/jtm/taaa061 (2020). 10.1093/jtm/taaa061 PMC7604850 32330261

[48] Schirmer, P. L. et al. Zika virus infection in the Veterans Health Administration (VHA), 2015–2016. *PLoS Negl. Trop. Dis.* **12**, e0006416 (2018). 29795560 10.1371/journal.pntd.0006416 PMC5967711

[49] Hoen, B. et al. Pregnancy outcomes after ZIKV infection in French Territories in the Americas. *N. Engl. J. Med.* **378**, 985–994 (2018). 29539287 10.1056/NEJMoa1709481

[50] Clemente, N. et al. Zika virus infection in pregnancy and adverse fetal outcomes in SAo Paulo State, Brazil: a prospective cohort study. *Sci. Rep.*10.1038/s41598-020-69235-0 (2020). 10.1038/s41598-020-69235-0 PMC7391725 32728054

[51] Wongsawat, J. et al. Characteristics, risk factors, and outcomes related to Zika virus infection during pregnancy in Northeastern Thailand: a prospective pregnancy cohort study. *PLoS Negl. Trop. Dis.* **18**, 2018–2020 (2024). 10.1371/journal.pntd.0012176 PMC11139345 38758964

[52] Marbán-Castro, E. et al. Zika virus infection in pregnant travellers and impact on childhood neurodevelopment in the first two years of life: A prospective observational study. *Travel Med. Infect. Dis.*10.1016/j.tmaid.2021.101985 (2021). 10.1016/j.tmaid.2021.101985 33601028

[53] Petrone, M. et al. Asynchronicity of endemic and emerging mosquito-borne disease outbreaks in the Dominican Republic. *Nat. Commun.*10.1038/s41467-020-20391-x (2021). 10.1038/s41467-020-20391-x PMC7794562 33420058

[54] Lannuzel, A. et al. Long-term outcome in neuroZika: when biological diagnosis matters. *Neurology* **92**, e2406–e2420 (2019). 31028126 10.1212/WNL.0000000000007536

[55] Cunha, A. J., de Magalhaes-Barbosa, M. C., Lima-Setta, F., Medronho, R. A. & Prata-Barbosa, A. Microcephaly case fatality rate associated with Zika virus infection in Brazil: current estimates. *Pediatr. Infect. J.* **36**, 528–530 (2017). 10.1097/INF.0000000000001486 28403061

[56] Pereira, J. et al. The role of amniocentesis in the diagnosis of congenital Zika syndrome. *Clin. Infect. Dis.* **69**, 713–716 (2019). 30624579 10.1093/cid/ciz013 PMC6669287

[57] Charniga, K. et al. Descriptive analysis of surveillance data for Zika virus disease and Zika virus-associated neurological complications in Colombia, 2015–2017. *PLoS ONE*10.1371/journal.pone.0252236 (2021). 10.1371/journal.pone.0252236 PMC8208586 34133446

[58] Flamand, C. et al. The proportion of asymptomatic infections and spectrum of disease among pregnant women infected by Zika virus: systematic monitoring in French Guiana, 2016. *Euro Surveill.*10.2807/1560-7917.es.2017.22.44.17-00102 (2017). 10.2807/1560-7917.ES.2017.22.44.17-00102 PMC5710134 29113627

[59] Aubry, M. et al. Zika virus seroprevalence, French Polynesia, 2014–2015. *Emerg. Infect. Dis.* **23**, 669–672 (2017). 28084987 10.3201/eid2304.161549 PMC5367400

[60] Melo, N. D. L., Sousa, D. F. D. & Laporta, G. Z. Microcephaly and associated risk factors in newborns: a systematic review and meta-analysis study. *Trop. Med. Infect. Dis.* **7**, 261 (2022). 36288003 10.3390/tropicalmed7100261 PMC9611276

[61] Nithiyanantham, S. F. & Badawi, A. Maternal infection with Zika virus and prevalence of congenital disorders in infants: systematic review and meta-analysis. *Can. J. Public Health* **110**, 638–648 (2019). 31077071 10.17269/s41997-019-00215-2 PMC6964464

[62] Martins, M. M. et al. Fetal, neonatal, and infant outcomes associated with maternal Zika virus infection during pregnancy: a systematic review and meta-analysis. *PLoS ONE* **16**, e0246643 (2021). 33606729 10.1371/journal.pone.0246643 PMC7894820

[63] Coelho, A. & Crovella, S. Microcephaly prevalence in infants born to Zika Virus-infected women: a systematic review and meta-analysis. *Int. J. Mol. Sci.* **18**, 1714 (2017). 28783051 10.3390/ijms18081714 PMC5578104

[64] Gallo, L. G. et al. Another piece of the Zika puzzle: assessing the associated factors to microcephaly in a systematic review and meta-analysis. *BMC Public Health* **20**, 827 (2020). 32487247 10.1186/s12889-020-08946-5 PMC7266116

[65] França, G. V. A. et al. Congenital Zika virus syndrome in Brazil: a case series of the first 1501 livebirths with complete investigation. *Lancet* **388**, 891–897 (2016). 27372398 10.1016/S0140-6736(16)30902-3

[66] Pomar, L. et al. Association between Zika virus and fetopathy: a prospective cohort study in French Guiana. *Ultrasound Obstet. Gynecol.* **49**, 729–736 (2017). 28078779 10.1002/uog.17404

[67] Delatorre, E., Fernández, J. & Bello, G. Investigating the role of Easter Island in migration of Zika virus from South Pacific to Americas. *Emerg. Infect. Dis.* **24**, 2119–2121 (2018). 30334729 10.3201/eid2411.180586 PMC6200010

[68] Ferguson, N. M. et al. Countering the Zika epidemic in Latin America. *Science* **353**, 353–354 (2016). 27417493 10.1126/science.aag0219 PMC5475255

[69] Parker, D. M. et al. High Seroprevalence to *Aedes*-borne arboviruses in Ethiopia: a cross-sectional survey in 2024. 10.1101/2024.09.19.24313917 (2024).

[70] Gobillot, T. A. et al. Zika virus circulates at low levels in Western and Coastal Kenya. *J. Infect. Dis.* **222**, 847–852 (2020). 32242626 10.1093/infdis/jiaa158 PMC7399697

[71] Fourié, T., Grard, G., Leparc-Goffart, I., Briolant, S. & Fontaine, A. Variability of Zika virus incubation period in humans. *Open Forum Infect. Dis.* **5**, ofy261 (2018). 30397624 10.1093/ofid/ofy261 PMC6207619

[72] Zika: data and analysis. *Pan American Health Organization* https://www.paho.org/en/arbo-portal/zika-data-and-analysis/ (2025).

[73] Pathogen Epidemiology Review Group. Priority pathogens: wiki. https://github.com/mrc-ide/priority-pathogens/wiki/ (2025).

[74] Covidence systematic review software. (Covidence, 2025) https://www.covidence.org/

[75] Fitzjohn, R. et al. orderly2: orderly next generation. https://github.com/mrc-ide/orderly2 (2025).

[76] Schwarzer, G. meta: General package for meta-analysis. R version 8.2-1 https://cran.r-project.org/web/packages/meta/index.html (2025).

[77] Nash, R. et al. mrc-ide/priority-pathogens: Zika revisions release. *Zenodo*10.5281/zenodo.17201039 (2025).

[78] *Countries and territories with current or previous Zika virus transmission, by WHO regional office.* (World Health Organization, accessed 7 June 2025); https://cdn.who.int/media/docs/default-source/documents/emergencies/zika/countries-with-zika-and-vectors-table_february-2022.pdf?sfvrsn=4dc1f8ab_5

[79] GADM database of Global Administrative Areas. (GADM, accessed 13 May, 2025); https://gadm.org/ (2025).

[80] Armstrong, P. M. et al. Successive blood meals enhance virus dissemination within mosquitoes and increase transmission potential. *Nat. Microbiol.* **5**, 239–247 (2020). 31819213 10.1038/s41564-019-0619-y PMC7199921

[81] Champagne, C. et al. Structure in the variability of the basic reproductive number (R0) for Zika epidemics in the Pacific islands. *eLife* **5**, e19874 (2016). 27897973 10.7554/eLife.19874 PMC5262383

[82] Guo, X. et al. Vector competence and vertical transmission of Zika virus in *Aedes albopictus* (Diptera: Culicidae). *Vector Borne Zoonotic Dis* **20**, 374–379 (2020). 31934825 10.1089/vbz.2019.2492

[83] Hugo, L. E. et al. Vector competence of Australian Aedes aegypti and *Aedes albopictus* for an epidemic strain of Zika virus. *PLoS Negl. Trop. Dis.* **13**, e0007281 (2019). 30946747 10.1371/journal.pntd.0007281 PMC6467424

[84] Kucharski, A. J. et al. Transmission dynamics of Zika virus in island populations: a modelling analysis of the 2013–14 French Polynesia outbreak. *PLoS Negl. Trop. Dis.* **10**, e0004726 (2016). 27186984 10.1371/journal.pntd.0004726 PMC4871342

[85] Ryckebusch, F., Berthet, M., Missé, D. & Choumet, V. Infection of a French population of *Aedes albopictus* and of *Aedes aegypti* (Paea Strain) with Zika virus reveals low transmission rates to these vectors’ Saliva. *Int. J. Mol. Sci.* **18**, 2384 (2017). 29125545 10.3390/ijms18112384 PMC5713353

[86] Shutt, D. P., Manore, C. A., Pankavich, S., Porter, A. T. & Del Valle, S. Y. Estimating the reproductive number, total outbreak size, and reporting rates for Zika epidemics in South and Central America. *Epidemics* **21**, 63–79 (2017). 28803069 10.1016/j.epidem.2017.06.005

[87] Winokur, O. C., Main, B. J., Nicholson, J. & Barker, C. M. Impact of temperature on the extrinsic incubation period of Zika virus in *Aedes aegypti*. *PLoS Negl. Trop. Dis.* **14**, e0008047 (2020). 32187187 10.1371/journal.pntd.0008047 PMC7105136

[88] Zimler, R. A. & Alto, B. W. The extrinsic incubation period of Zika virus in Florida mosquitoes *Aedes aegypti* and *Ae. albopictus*. *Pathogens* **10**, 1252 (2021). 34684201 10.3390/pathogens10101252 PMC8537051

[89] Abtibol-Bernardino, M. R. et al. Would Zika virus infection in pregnancy be a sentence of poor neurological prognosis for exposed children? Neurodevelopmental outcomes in a cohort from Brazilian Amazon. *Viruses* **14**, 2659 (2022). 36560662 10.3390/v14122659 PMC9782914

[90] Alger, J. et al. Microcephaly outcomes among Zika virus–infected pregnant women in Honduras. *Am. J. Trop. Med. Hyg.* **104**, 1737–1740 (2021). 33724927 10.4269/ajtmh.20-1483 PMC8103474

[91] Alvarado-Domenech, L. I. et al. Early childhood neurodevelopmental outcomes in children with prenatal Zika virus exposure: a cohort study in Puerto Rico. *J. Pediatr.* **247**, 38–45.e5 (2022). 35577118 10.1016/j.jpeds.2022.05.016 PMC10188121

[92] Aromolaran, A. et al. Unequal burden of Zika-associated microcephaly among populations with public and private healthcare in Salvador, Brazil. *Int. J. Infect. Dis.* **120**, 201–204 (2022). 35470025 10.1016/j.ijid.2022.04.030 PMC9119857

[93] Brasil, P. et al. Zika virus infection in pregnant women in Rio de Janeiro. *N. Engl. J. Med.* **375**, 2321–2334 (2016). 26943629 10.1056/NEJMoa1602412 PMC5323261

[94] Brasil, P. et al. Zika virus vertical transmission in children with confirmed antenatal exposure. *Nat. Commun.* **11**, 3510 (2020). 32665616 10.1038/s41467-020-17331-0 PMC7360785

[95] Cranston, J. S. et al. Association between antenatal exposure to Zika virus and anatomical and neurodevelopmental abnormalities in children. *JAMA Netw. Open* **3**, e209303 (2020). 32633763 10.1001/jamanetworkopen.2020.9303 PMC7341180

[96] De Melo Espindola, O. et al. ZIKA virus neutralizing antibody kinetics in antenatally exposed infants. *J. Infect. Dis.* **224**, 1060–1068 (2021). 33528564 10.1093/infdis/jiab054 PMC8448431

[97] de Sousa, I. B. A. et al. Gestational outcomes in women infected by Zika virus during pregnancy in Mato Grosso do Sul, Brazil: a cross-sectional study. *Int. J. Infect. Dis.* **98**, 359–365 (2020). 32619757 10.1016/j.ijid.2020.06.084

[98] Grant, R. et al. Maternal and neonatal outcomes related to Zika virus in pregnant women in Southern Vietnam: an epidemiological and virological prospective analysis. *Lancet Reg. Health West. Pac.* **11**, 100163 (2021). 34327365 10.1016/j.lanwpc.2021.100163 PMC8315393

[99] Grant, R. et al. Consequences of in utero Zika virus exposure and adverse pregnancy and early childhood outcomes: a prospective cohort study. *Viruses* **14**, 2755 (2022). 36560760 10.3390/v14122755 PMC9788325

[100] Gutiérrez-Sánchez, L. Á. et al. Fetal central nervous system anomalies according to RT-PCR and trimester of maternal infection with Zika virus: a prospective cohort study. *Acta Obstet. Gynecol. Scand.* **101**, 221–231 (2022). 34904224 10.1111/aogs.14301 PMC9564424

[101] Hcini, N. et al. Association between confirmed congenital Zika infection at birth and outcomes up to 3 years of life. *Nat. Commun.* **12**, 3270 (2021). 34075035 10.1038/s41467-021-23468-3 PMC8169933

[102] Honein, M. A. et al. Birth defects among fetuses and infants of US women with evidence of possible Zika virus infection during pregnancy. *JAMA* **317**, 59–68 (2017). 27960197 10.1001/jama.2016.19006

[103] Lee, E. H. et al. First 12 Months of life for infants in New York City, New York, with possible congenital Zika virus exposure. *J. Pediatr. Infect. Dis. Soc.* **9**, 311–319 (2020). 10.1093/jpids/piz027 PMC7358042 31125410

[104] Martínez-Arias, A. et al. Zika virus screening during pregnancy: results and lessons learned from a screening program and a post-delivery follow-up analysis (2016–2022). *Birth Defects Res.* **115**, 1646–1657 (2023). 37668290 10.1002/bdr2.2236

[105] Martins, R. S. et al. The role of pregnant women with rash in the Zika virus sentinel surveillance. *Rev. Soc. Bras. Med. Trop.* **52**, e20180351 (2019). 30892549 10.1590/0037-8682-0351-2018

[106] Merriam, A. A., Nhan-Chang, C.-L., Huerta-Bogdan, B. I., Wapner, R. & Gyamfi-Bannerman, C. A single-center experience with a pregnant immigrant population and Zika virus serologic screening in New York City. *Am. J. Perinatol.* **37**, 731–737 (2020). 31146294 10.1055/s-0039-1688819

[107] Moore, S. M. et al. Leveraging multiple data types to estimate the size of the Zika epidemic in the Americas. *PLoS Negl. Trop. Dis.* **14**, e0008640 (2020). 32986701 10.1371/journal.pntd.0008640 PMC7544039

[108] Osoro, E. et al. Prevalence of microcephaly and Zika virus infection in a pregnancy cohort in Kenya, 2017–2019. *BMC Med.* **20**, 291 (2022). 36100910 10.1186/s12916-022-02498-8 PMC9470235

[109] Pacheco, O. et al. Zika virus disease in Colombia—preliminary report. *N. Engl. J. Med.* **383**, e44 (2020). 27305043 10.1056/NEJMoa1604037

[110] Pereira, J. P. et al. Association of prenatal ultrasonographic findings with adverse neonatal outcomes among pregnant women with Zika virus infection in Brazil. *JAMA Netw. Open* **1**, e186529–e186529 (2018). 30646333 10.1001/jamanetworkopen.2018.6529 PMC6324324

[111] Pimentel, R. et al. Birth defects and long-term neurodevelopmental abnormalities in infants born during the Zika virus epidemic in the Dominican Republic. *Ann. Glob. Health* **87**, 4 (2021). 33505863 10.5334/aogh.3095 PMC7792457

[112] Pool, K.-L. et al. Association between neonatal neuroimaging and clinical outcomes in Zika-exposed infants from Rio de Janeiro, Brazil. *JAMA Netw. Open* **2**, e198124–e198124 (2019). 31365112 10.1001/jamanetworkopen.2019.8124 PMC6669783

[113] Robbiani, D. F. et al. Risk of Zika microcephaly correlates with features of maternal antibodies. *J. Exp. Med.* **216**, 2302–2315 (2019). 31413072 10.1084/jem.20191061 PMC6781003

[114] Rodrigues, Md. S. P. et al. Repercussions of Zika virus emergency on the health of the population of Tocantins state, Brazil, 2015 and 2016: a descriptive study. *Epidemiol. Serv. Saude* **29**, e2020096 (2020). 32756832 10.5123/s16/79-49742020000400008

[115] Rosado, L. E. P. et al. Risk of adverse pregnancy and infant outcomes associated with prenatal Zika virus infection: a post-epidemic cohort in Central-West Brazil. *Sci. Rep.* **13**, 7335 (2023). 37147405 10.1038/s41598-023-33334-5 PMC10161159

[116] Sanchez Clemente, N. et al. Can Zika virus infection in high risk pregnant women be differentiated on the basis of symptoms? *Viruses* **12**, 1263 (2020). 33167566 10.3390/v12111263 PMC7694531

[117] Soriano-Arandes, A. et al. Clinical outcomes of a Zika virus mother–child pair cohort in Spain. *Pathogens* **9**, 352 (2020). 32392815 10.3390/pathogens9050352 PMC7281364

[118] Tannis, A. et al. Birth outcomes related to prenatal Zika, Dengue, and other flavivirus infections in the Zika en Embarazadas y Niños Prospective Cohort Study in Colombia. *Am. J. Trop. Med. Hyg.* **111**, 622 (2024). 38981499 10.4269/ajtmh.23-0873 PMC11376175

[119] Tozetto-Mendoza, T. R. et al. Zika virus infection among symptomatic patients from two healthcare centers in Sao Paulo State, Brazil: prevalence, clinical characteristics, viral detection in body fluids and serodynamics. *Rev. Inst. Med. Trop. Sao Paulo* **61**, e19 (2019). 10.1590/S1678-9946201961019 PMC6453419 30970110

[120] Ximenes, R. A. d. A. et al. Zika-related adverse outcomes in a cohort of pregnant women with rash in Pernambuco, Brazil. *PLoS Negl. Trop. Dis.* **15**, e0009216 (2021). 33684110 10.1371/journal.pntd.0009216 PMC7971861

