# Supplementary material for: A systematic review and meta-analysis of Zika virus epidemiology
Source: Nat Health. 2026 Feb 13;1(3):355–67. doi: 10.1038/s44360-025-00051-4 (PMC12965883; doi:10.1038/s44360-025-00051-4)
Supplement: Supplementary file 1 — Supplementary Information Supplementary Methods, Figs. A1–B36 and Tables A1–C19. [file 44360_2025_51_MOESM1_ESM.pdf]

nature health

Supplementary information https://doi.org/10.1038/s44360-025-00051-4

A systematic review and meta-analysis of Zika virus epidemiology

In the format provided by the authors and unedited

Contents

A Extended Methods **4**
- A.1 Search strategy and screening . . . 4
- A.2 Data extraction . . . 7
  - A.2.1 Outbreaks . . . 7
  - A.2.2 Models . . . 7
  - A.2.3 Parameters . . . 7
  - A.2.4 Quality assessment . . . 8
- A.3 Data analysis . . . 8
  - A.3.1 Meta-Analysis . . . 8
  - A.3.2 Doubling time calculation . . . 9
  - A.3.3 Reporting Uncertainty . . . 9
- A.4 Extraction Fields . . . 9
  - A.4.1 Model extraction fields . . . 9
  - A.4.2 Parameter extraction fields . . . 10
  - A.4.3 Outbreak extraction fields . . . 12

B Supplementary results **13**
- B.1 Quality assessment results . . . 13
- B.2 Summary of extracted models and parameters . . . 13
- B.3 Additional plots of parameters . . . 15
  - B.3.1 Basic reproduction numbers . . . 15
  - B.3.2 Effective reproduction numbers . . . 18
  - B.3.3 Seroprevalence . . . 20
  - B.3.4 Genomic parameters . . . 21
  - B.3.5 Pregnancy loss proportion . . . 22
  - B.3.6 Severity . . . 24
  - B.3.7 Other transmission parameters . . . 27
  - B.3.8 Risk factors . . . 31
- B.4 Main figures with no QA filtering . . . 34
- B.5 Extended meta-analyses on CZS proportion and pregnancy loss proportion . . . 38
- B.6 Meta-analyses on proportion of symptomatic individuals . . . 44
- B.7 Tables of extracted parameters, models, and outbreaks . . . 46
  - B.7.1 Models . . . 46
  - B.7.2 Outbreaks . . . 52
  - B.7.3 Delays . . . 56
  - B.7.4 Seroprevalence . . . 61
  - B.7.5 Risk factors . . . 82
  - B.7.6 Transmission . . . 100
  - B.7.7 Genomic parameters . . . 111
  - B.7.8 Severity . . . 112
  - B.7.9 Relative contributions . . . 113
  - B.7.10 Adverse birth outcomes . . . 114

C PRISMA 2020 Checklists **118**

List of Figures

A.1 Cohen's Kappa for screening and full text review . . . 6
B.2 Article count and Quality assessment results . . . 13
B.3 Summary of extracted parameters . . . 14
B.4 Summary of extracted models . . . 15
B.5 Basic reproduction numbers, sexual and vector-borne . . . 16
B.6 Basic reproduction numbers, sexual and vector-borne, from all studies. . . . 17
B.7 Effective reproduction numbers . . . 18
B.8 Effective reproduction numbers from all studies. . . . 19
B.9 Number Seroprevalence studies by population group . . . 20
B.10 Seroprevalence by population group . . . 20
B.11 Seroprevalence by assay . . . 21
B.12 Genomic information . . . 21
B.13 Genomic information not filtered by quality assessment score . . . 22
B.14 Miscarriage proportion estimates . . . 23
B.15 Case fatality ratio estimates . . . 24
B.16 Proportion of cases that are symptomatic from all studies . . . 25
B.17 Proportion of cases that are symptomatic from studies with high QA score . . . 26
B.18 Attack rate from all studies . . . 28
B.19 Attack rate from studies with high QA score . . . 29
B.20 Growth rate from all studies . . . 30
B.21 Sexual relative contribution to transmission from all studies . . . 30
B.22 Sexual relative contribution to transmission from studies with high QA score . . . 31
B.23 Most commonly reported risk factors and associated outcomes . . . 32
B.24 Other reported outcomes and risk factors . . . 33
B.25 Reproduction of figure 2 in main text, but with no filtering by QA score . . . 34
B.26 Reproduction of figure 3 in main text, but with no filtering by QA score . . . 35
B.27 Reproduction of figure 4 in main text, but with no filtering by QA score . . . 36
B.28 Reproduction of figure 5 in main text, but with no filtering by QA score . . . 37
B.29 Meta-analysis bias assessment for CZS proportion . . . 38
B.30 Meta-analysis bias assessment for pregnancy loss proportion . . . 39
B.31 CZS proportion meta-analysis by continent . . . 40
B.32 CZS proportion meta-analysis of estimates from Brazil, stratified by trimester of ZIKV exposure . . . 41
B.33 Pregnancy loss proportion meta-analysis . . . 42
B.34 Pregnancy loss proportion meta-analysis, stratified by pregnancy outcome. . . . 43
B.35 Meta-analysis of the proportion of symptomatic individuals . . . 44
B.36 Meta-analysis bias assessment for proportion of symptomatic individuals . . . 45

List of Tables

A.1 Inclusion and exclusion criteria for papers. . . . 5
A.2 Available Vignettes in the `epireview` Package . . . 7
A.3 Quality assessment questionnaire . . . 9
A.4 Model form fields . . . 10
A.5 Parameters form fields . . . 12
A.6 Outbreak form fields . . . 12
B.7 Overview of extracted transmission models. . . . 51
B.8 Overview of extracted outbreaks. Starred locations indicate outbreaks with reported dates but with no case numbers reported, which are included for completeness. . . . 55
B.9 Overview of extracted delay parameters. . . . 60
B.10 Overview of extracted seroprevalence parameters. . . . 81
B.11 Overview of extracted risk factors . . . 99
B.12 Overview of extracted transmission parameters. . . . 110
B.13 Overview of extracted genomic parameters. . . . 111

B.14 Overview of extracted severity parameters. 112
B.15 Overview of extracted relative contribution parameters. 113
B.16 Overview of extracted CZS proportion and pregnancy loss proportion parameters. . . . 117
C.17 PRISMA 2020 Abstracts Checklist. ([14]) 118
C.18 PRISMA 2020 Checklist. ([14]) 118
C.18 PRISMA 2020 Checklist. ([14]) 119
C.18 PRISMA 2020 Checklist. ([14]) 120
C.18 PRISMA 2020 Checklist. ([14]) 121

Overview

We provide further details of the methodology (section A) and additional results (section B), including tables of all of the information extracted as part of the review (section B.7). We report the PRISMA checklists in Section C. Section ?? lists all members of the Pathogen Epidemiology Review Group who contributed to this work.

A Extended Methods

This systematic review and meta-analysis for Zika Virus Disease is part of a series of systematic reviews of nine priority pathogens on WHO's 2018 R&D Blueprint List [1] and was registered on PROSPERO (International Prospective Register of Systematic Reviews, CRD42023393345). Other systematic reviews in this series completed to date studied Marburg virus disease [2], Ebola virus disease [3], Lassa fever [4], and SARS-CoV-1[5].

The code to reproduce the results presented in this manuscript is available at `https://github.com/mrc-ide/priority-pathogens`.

A.1 Search strategy and screening

Database search term:

```
zika AND ((transmissi* OR epidemiolog*) OR (model* NOT imag*) OR (severity OR "case
    fatality ratio*" OR CFR OR "case fatality rate*" OR "mortality rate*" OR "attack
    rate*") OR ("infectious period*" OR "serial interval*" OR "incubation period*" OR
    "generation time*" OR "generation interval*" OR "latent period*" OR latency) OR
    (heterogeneit* OR superspread* OR "super spread*" OR super-spread* OR
    overdispersion OR overdispersed OR over-dispersion OR over-dispersed OR "over
    dispersion" OR "over dispersed") OR (infectivity OR infectiousness OR "growth
    rate*" OR "reproduction number*" OR "reproductive number*" OR R0 OR "reproduction
    ratio*" OR "reproductive rate*") OR ("pre-existing immunity" OR serological OR
    serology OR serosurvey*) OR (evolution* OR mutation* OR substitution*) OR
    (outbreak* OR cluster* OR epidemic*) OR (risk factor*") OR ("extrinsic incubation
    period" OR EIP))
```

This search term is analogous for all pathogens in the series of systematic reviews. For this review, "extrinsic incubation period OR EIP" was added to the end. The database search was conducted on 6 March 2019 and repeated on 31 October 2024. All articles were imported into *Covidence*, a systematic review management software for screening.

We included peer-reviewed original research articles in English if they reported ZIKV disease parameters (including seroprevalence, reproduction numbers, genomic information, severity, risk factors, Zika congenital syndrome/microcephaly proportion, pregnancy loss proportion, or delays), outbreaks, and/or transmission models. The inclusion and exclusion criteria are listed below A.1.

Inclusion	Exclusion
Measures/estimates of human: Reproduction numbers (R, R_0, R_t, r, R_e), growth rate (r), doubling times, generation time, serial interval, incubation/latent period, case fatality ratio (CFR), attack rate, mutation rate (e.g. from phylogenetic study), overdispersion, risk factors (risk and the measure), microcephaly or pregnancy loss proportion.	Non-English language publication
Mention of historical or any outbreak in humans: size, year, location, duration, spatial scale	Studies of co-infections. (local, regional, national, international).
Measures/estimates of animal: R, R_0, R_t, r, R_e, growth rate, mutation rate.	Animal studies that do not report R, R_t etc.
Mathematical or statistical model of transmission.	Qualitative studies, e.g., KAP studies.
Measures of seroprevalence and negative seroprevalence in humans.	Pathogen not the primary focus of study.
Relative ratio of human-human vs animal introductions.	Duplicates.
Reviews that report inclusion criteria for reference checking.	Does not match any of the inclusion criteria.
For "small" pathogens[1], include case reports to potentially reconstruct serial interval distribution, etc.	In-vitro studies.
	Non-peer reviewed publications, conference proceedings, abstracts, posters, letters to the editor

Table A.1: Inclusion and exclusion criteria for papers.

Two independent reviewers for both the abstract screening and full-text review stages screened the identified articles to assess eligibility and accordance with the inclusion/exclusion criteria (see list of the reviewers in the Methods section of main text). Any disagreements between reviewers were resolved by consensus between the two reviewers. We show the Cohen's Kappa statistic of the inter-rater reliability in A.1.

Per the original research criterion in A.1, all reviews were excluded. From the systematic reviews identified in our search (**n=66**), we extracted the citations from these reviews and cross-referenced them with those already screened in Covidence. Among the citations that had not been previously screened in Covidence, we filtered the list to include only those with the word "Zika" in the title (n=350), which we manually checked for duplicates, relevance, and publication type. In total, 103 new papers were added to Covidence to be screened, of which some were duplicates or not found. Of the 69 left, we included 6 studies from which we extracted data. This list of studies is provided in epireview.

We provide a csv of articles included in the study hosted in the epireview package. The below code can be used to generate this list from epireview.

```
require(tidyverse)

is_epireview_available <- require("epireview")

if(!is_epireview_available)
{
  is_remotes_available   <- require('remotes')

  if(!is_remotes_available)
    install.packages('remotes')

  remotes::install_github('mrc-ide/epireview')
}

zika      <- epireview::load_epidata('zika')
```

The epireview package has multiple vignettes available, listed below.

[1]The inclusion/exclusion criteria are reported as stated in the PROSPERO registration for the overall priority pathogen project. The threshold is determined on a case-by-case basis for each pathogen, and ZIKV is not considered a "small" pathogen.

Title and abstract screening

Reviewer A	Reviewer B	n	Proportionate Agreement	Yes Probability	No Probability	Random Agreement Probability	Cohen's Kappa
P3	P1	2547	98.23	1.07	80.38	81.45	90.48
P14	P10	1644	98.36	0.09	94.07	94.16	71.88
P3	P5	1586	93.88	5.31	59.22	64.53	82.76
P2	P3	1446	98.06	0.01	97.66	97.67	16.7
P10	P11	1272	91.59	0.9	81.48	82.39	52.24
P14	P11	1271	95.12	0.35	88.55	88.89	56.08
P1	P5	1213	83.76	5.25	59	64.25	54.56
P2	P1	422	89.57	2.44	71.16	73.6	60.5
P2	P5	167	78.44	5.72	57.82	63.54	40.87
P13	P10	156	94.87	0.43	86.33	86.76	61.27
P15	P5	95	80	57.91	5.27	63.18	45.68
P2	P8	12	100	0	100	100	NA
P3	P8	5	100	0	100	100	NA
P1	P8	5	100	0	100	100	NA
P5	P4	2	50	0	50	50	0

Full text review

Reviewer A	Reviewer B	n	Proportionate Agreement	Yes Probability	No Probability	Random Agreement Probability	Cohen's Kappa
P3	P1	430	93.26	21.66	28.4	50.06	86.5
P2	P1	221	77.38	28.74	20.15	48.89	55.73
P10	P11	209	72.73	34.89	16.71	51.6	43.66
P3	P5	143	82.52	15.03	37.41	52.44	63.24
P2	P3	116	97.41	13.41	40.13	53.54	94.43
P3	P4	81	74.07	5.76	57.61	63.37	29.21
P9	P1	31	74.19	29.97	20.29	50.26	48.12
P12	P1	22	81.82	24.79	24.79	49.59	63.93
P1	P8	20	90	8.75	48.75	57.5	76.47
P1	P4	16	100	0	100	100	NA
P1	P6	15	73.33	52	5.33	57.33	37.5
P1	P5	8	100	1.56	76.56	78.12	100
P7	P1	7	57.14	24.49	24.49	48.98	16
P2	P8	5	100	100	0	100	NA
P12	P5	5	100	0	100	100	NA
P5	P4	4	100	0	100	100	NA
P2	P4	2	100	0	100	100	NA
P3	P7	2	50	0	50	50	0
P2	P5	2	50	50	0	50	0
P3	P8	1	0	0	0	0	0
P12	P3	1	100	0	100	100	NA
P12	P7	1	100	0	100	100	NA
P2	P7	1	0	0	0	0	0

Figure A.1: Cohen's Kappa for screening and full text review. P1-P15 are members of PERG (see supplement D), and we have provided who participated at each stage in the methods section of the main text. n is the number of studies considered by each Reviewer A/B pair. We note that two people screened every study at both the Abstract/title and full-text review stages and any conflicts were resolved by discussion and mutual agreement of Reviewer A and B.

Table A.2: Available Vignettes in the `epireview` Package

**Title**	**Description**
Disease Overview	Vignette for Marburg Virus Disease, Lassa fever, SARS-CoV-1 and Zika with tables and figures from the respective papers. This will be updated as data are added to the database.
Field Options	Vignette listing the options for each outbreak, model, or parameter field and explaining how to access them using functions in the package.
Database Update	Vignette explaining the process of updating the database with new article, outbreak, model, or parameter data.

A.2 Data extraction

Once studies were identified as eligible for data extraction, they moved to the data extraction stage. We used a custom Microsoft Access database (Version 2503) that collected information on article metadata (including first author, title, journal, publication year, volume) and a quality assessment score (see A.2.4 below). We also extracted information on models, outbreaks, and parameters. See the wiki on Github for more details.

To validate data extraction, 16% (e.g. >10% required in our protocol) of identified papers were randomly selected for double extraction by two independent extractors to ensure concordance in the team of 16 extractors (see Methods in main text). Any discordance was resolved between the pairs of extractors. All of the remaining studies were extracted by a single extractor, but data were further validated during cleaning and analysis.

A.2.1 Outbreaks

Because of the complexity of the definition of ZIKV outbreaks that may not be well-defined, outbreaks were extracted from studies only if defined as such (either as "outbreaks" or "epidemics") by the study authors. We note that many outbreaks are reported in non-peer-reviewed literature, such as reports or correspondences, which would have been excluded from this review due to the inclusion/exclusion criteria (Table A.1).

A.2.2 Models

Information about transmission models for ZIKV disease was extracted from eligible papers. We collected information including model type, whether it modelled a stochastic or deterministic process, transmission pathways and interventions modelled, and compartments (e.g. SEIR-SEI) of compartmental models. We also extracted information about model assumptions, whether they were fitted to data or theoretical, and code availability.

A.2.3 Parameters

We extracted parameter values and types of values (e.g. mean, median), uncertainty intervals and types (e.g. confidence or credible intervals), variability (e.g. ranges or IQRs around a central estimate), and ranges of central tendency values across multiple estimates obtained from some form of disaggregation of data (e.g. age-stratified estimates or estimates from different populations). Parameter contexts were also recorded (e.g. population sample type, population group, location, country, ages, survey dates, sample size, and trimester of pregnancy where available).

For any genomic information, we recorded the part of the genome studied and if any new sequences were available. We recorded information about the method used to estimate reproduction numbers. Additionally, for reproduction numbers, we investigated estimates that were outliers from the others; in doing this, we found two estimates of the basic reproduction number that were in fact effective reproduction numbers (estimates of R after control measures were implemented). These two estimates were relabelled as Effective Reproduction numbers in the database.

For case fatality ratios, CZS proportion, seroprevalence, and pregnancy loss proportion, we recorded the numerators and denominators if reported and whether estimates were adjusted, either for censoring, test sensitivity/specificity or in any other way, or not.

Risk factors are highly dependent on the context of the data used to estimate it, so we extracted only the outcome (e.g. ZIKV infection, microcephaly) and risk factor (e.g. age, ZIKV-infected mother, sex, occupation), the type of occupation if specified, and whether the estimate(s) were statistically significant and/or adjusted. We chose not to extract the values of the risk factor estimates (e.g. odds or risk ratios) or the direction of the estimate for simplicity, as estimates are dependent on the context of the study and the reference group used, which requires many further fields for extraction. Levels of statistical significance were taken as reported in the paper. We show an overview of reported risk factors for a range of outcomes that may be useful for parameterising or designing transmission models. For each study, we combined risk factors and outcomes together; for example, if both age and sex were significant, adjusted risk factors for ZIKV infection and death, we would have included age and sex in the risk factor column and infection and death in the risk factor outcome column, resulting in a single risk factor entry in the database. For each study, the maximum number of risk factors that could have been extracted was 4 - significant/adjusted, significant/unadjusted, non-significant/adjusted, non-significant/unadjusted.

We extracted multiple forms of the reproduction number. The overall reproduction number can be separated into multiple routes of transmission, where the vector-borne reproduction number represents the average number of new human infections from a single infectious human via vector-borne transmission, while the sexual reproduction number refers to the average number of new cases via sexual transmission. The overall reproduction number is the sum of the transmission-route-specific estimates, assuming independence in transmission route.

Many papers reported multiple parameter values for the same parameter (e.g. across multiple locations, time periods, assumptions, sample types, etc.). For these, we applied the 'rule of 3', where if more than 3 parameter values were reported across these disaggregated groups, we only captured the range of central estimates reported for this parameter. We applied the 'rule of 3' to all disaggregation groups except locations (e.g. if R_0 estimates were reported across 10 admin 1 units, we extracted each estimate separately, whereas if there were 6 estimates corresponding to different age groups, we would only extract the range of the central estimates).

A.2.4 Quality assessment

For each study, we provided a quality assessment (QA) score derived using a custom questionnaire (A.3, the wiki on Github). The overall QA score was calculated as the mean number of "Yes" answers, excluding any non-applicable questions. We show the distribution of QA scores and trends of QA scores by publication year (based on a fit of a local polynomial regression using `ggplot2::geom_smooth()` in R) in B.2.

A.3 Data analysis

A.3.1 Meta-Analysis

The meta-analyses for the proportion of CZS and pregnancy loss among ZIKV-infected mothers (in Figure 5 in the main text) followed a standard approach and was conducted using the `meta` R package [6]. Estimates were included in the meta-analyses if they were assigned a QA score of ≥50%, had ≥10 pregnant women with confirmed ZIKV infection in the denominator, and used a study design that was deemed by the authors to not bias estimates by selecting for the outcome (pregnancy loss or CZS). We combined the 3 cluster-level estimates from Robbiani et al. [7] into a single overall estimate for the meta-analysis.

A mixed-effects model is a linear model $y_i = \beta_0 + \sum_{j=1} \beta_j x_{ij} + u_i + \epsilon_i$, where y_i are the observed data, x_{ij} are explanatory variables, β_j are the fixed effects, u_i are the random effects (centered around zero and independent across i), and ϵ_i are error terms. Meta-analysis is a special case of the above mixed-effects model with only an intercept term β_0 (a fixed-effects/common-effects model) or only

Table A.3: Quality assessment questionnaire: possible responses for each question listed were: Yes, No, and Non-applicable (NA).

Theme	Question
Is the methodological/statistical approach suitable? (how the data are used)	1. Clear and reproducible<br>2. Robust and appropriate for the aim [subjective criteria]
Are the assumptions appropriate? (input parameters/assumptions - what goes into the methodology)	3. Clear and reproducible<br>4. Justified (published study or analysis of data)[objective criteria]
Are the data appropriate for the selected methodological approach?	5. Clearly described and reproducible<br>6. Are issues in the data clearly discussed and acknowledged?<br>7. Are issues in the data accounted for in the chosen methodological approach?

an intercept term β_0 and a random-effects term u_i associated with that intercept (a random-effects model).

We used a generalised logistic mixed-effects model (GLMM) in which the individual CZS proportion estimates were transformed using the logit-transformation $y_i = \log\left(\frac{CZS_i}{1-CZS_i}\right)$ to ensure that the distribution was approximately normal. A GLMM was then applied to the transformed values to estimate the pooled effect. A comprehensive overview of the methodology is provided by [8].

In Figure 5 of the main text, we perform a sub-group analysis of CZS proportion, in which estimates are grouped by population sample type. The meta-analysis in Panel B reports the common and random effects for each sub-group to investigate any patterns between groups.

The risk of publication bias was assessed through funnel plots (see Figures B.29, B.30) for the meta-analysis of CZS proportion and pregnancy loss proportion.

The code to reproduce our results is available on our GitHub repository.

A.3.2 Doubling time calculation

We report doubling times derived from extracted growth rates per day. Doubling times in days were calculated as: $T_d = \frac{log(2)}{r}$ where r is the daily growth rate.

A.3.3 Reporting Uncertainty

There was a wide variety of formats reported for uncertainty and ranges in the extracted studies. While most studies reported a central estimate for parameters of interest, some reported a range of central values, or we applied the 'rule of 3' as described in appendix A.2.3. When central estimates across more than 3 groups were reported, this is shown in our plots as thick shaded colour bars. When we refer to a range of central estimates in the main text, the maximum and minimum values of these are included.

We also extracted uncertainty values (e.g. standard deviation or variance) as well as paired uncertainty (e.g. confidence or credible intervals). Uncertainty intervals are represented as thin solid line error bars, and we did not plot standard deviation, variance, or standard error in our figures.

A.4 Extraction Fields

A.4.1 Model extraction fields

Data field	Expected data type	Variable name	Notes
Article ID	integer	article_id	ID to connect to article form
Model data ID	integer	model_data_id	ID assigned by database
Model type	character	model_type	General type of model - from dropdown list
Stochastic or deterministic	character	stoch_deter	Stochastic or deterministic model as reported
Transmission route	character	transmission_route	Transmission route(s) modelled - from dropdown list
Assumptions	character	assumptions	General assumptions for the model - from dropdown list
Compartmental type	character	compartmental_type	Specific type of compartmental model - from dropdown list
Theoretical model	logical	theoretical_model	Tick box whether the model was fitted to data (TRUE) or just theoretical (NA)
Intervention type	character	interventions_type	Type of intervention(s) modelled - from dropdown list
Code available	logical	code_available	Tick box whether code for model was publicly available and reported in the paper

Table A.4: Model form fields: refer to epireview package documentation for dropdown options.

A.4.2 Parameter extraction fields

Data field	Expected type	Variable name	Notes
Article ID	integer	article_ID	ID to connect to article form
Parameter data ID	integer	parameter_data_ID	ID assigned by database
Parameter type	character	parameter_type	category of parameter - see dropdown list
Parameter value	numeric	parameter_value	central parameter value
Parameter exponent	integer	exponent	parameter value exponent (base 10)
Inverse parameter	logical	inverse_param	tick box to indicate that only inverse of parameter is reported (e.g., recovery rate from fitted model instead of infectious period)
Parameter unit	character	parameter_unit	units for parameter value, applies to central estimate and ranges/uncertainty intervals - see dropdown list
Parameter value type	character	parameter_value_type	type of central parameter value - see dropdown list
Parameter lower bound	numeric	parameter_lower_bound	lower bound of the parameter range if a range was reported or if data are disaggregated
Parameter upper bound	numeric	parameter_upper_bound	upper bound of the parameter range if a range was reported or if data are disaggregated
Statistical approach	character	statistical_approach	Parameter estimated or observed - see dropdown list
Parameter uncertainty - single type	character	parameter_uncertainty_singe_type	type of uncertainty for central parameter value if single value was reported - see dropdown list
Parameter uncertainty - single value	numeric	parameter_uncertainty_single_value	value for uncertainty for central parameter value if a single value was reported (e.g. value of std. dev.)
Parameter uncertainty paired type	character	parameter_uncertainty_type	type of uncertainty for central parameter value if paired values were reported - see dropdown list
Parameter uncertainty - lower value	numeric	parameter_uncertainty_lower_value	lower bound for uncertainty for central parameter value if paired values were reported
Parameter uncertainty - upper value	numeric	parameter_uncertainty_upper_value	upper bound for uncertainty for central parameter value if paired values were reported
Distribution type	logical	distribution_type	type of distribution for estimated parameter - see dropdown list
First distribution parameter type	logical	distribution_par1_type	type of value for first distribution parameter - see dropdown list
First distribution parameter value	logical	distribution_par1_value	value for first distribution parameter (e.g. shape or scale parameter for a gamma distribution)
First distribution parameter uncertainty	logical	distribution_par1_uncertainty	tick box for whether uncertainty is estimated for the first distribution parameter (true) or not (false)
Second distribution parameter type	logical	distribution_par2_type	type of value for second distribution parameter - see dropdown list

Second distribution parameter value	logical	distribution_par2_value	value for second distribution parameter (e.g. shape or scale parameter for a gamma distribution)
Second distribution parameter uncertainty	logical	distribution_par2_uncertainty	tick box for whether uncertainty is estimated for the second distribution parameter (true) or not (false)
Disaggregated data available	logical	method_disaggregated	tick box if disaggregated estimates are available (true) or not (false)
Parameter estimates disaggregated by	character	method_disaggregated_by	categories for disaggregation of parameter estimates
Only disaggregated data available	logical	method_disaggregated_only	tick box if only disaggregated estimates are available (true) or if a central estimate is also available (false)
Is parameter from supplement?	logical	method_from_supplement	tick box for whether parameter was extracted from supplement (true) or not (false)
Is parameter in a figure only?	logical	parameter_fromfigure	tick box to indicate that parameter is plotted in a figure but not reported numerically in the text/table
Study population country	character	population_country	country of the survey population - see dropdown list
Study population location	character	population_location	region/district/province/city of the survey population - see dropdown list
Start day of study	integer	population_study_start_day	study start day
Start month of study	character	population_study_start_month	study start month - see dropdown list
Start year of study	integer	population_study_start_year	study start year - see dropdown list
End day of study	integer	population_study_end_day	study end day
End month of study	character	population_study_end_month	study end month - see dropdown list
End year of study	integer	population_study_end_year	study end year - see dropdown list
Survey timing related to outbreak	character	method_moment_value	timing of the survey in relation to the outbreak, if specified in paper - see dropdown list
Study population sample size	integer	population_sample_size	sample size of the population used for parameter estimation
Study population minimum age (years)	numeric	population_age_min	minimum age of the survey population in years
Study population maximum age (years)	numeric	population_age_max	maximum age of the survey population in years
Sex of study population	character	population_sex	sex of survey population - see dropdown list
Population sample setting	character	population_sample_type	general setting of the survey - see dropdown list
Population group	character	population_group	specific group of the survey population - see dropdown list
genome site	character	genome_site	site of genome or gene studied
Trimester of pregnant woman	character	trimester	trimester of pregnant woman's ZIKV exposure - see dropdown list
Urban/Rural area	character	urban_rural	urban or rural setting of ZIKV circulation - see dropdown list
Data availability	character	data_availability	Statement about availability of the data presented in the paper - see dropdown list
Gene	logical	Gene	Name of the genomic sequence or gene under analysis
Genomic sequence available?	logical	genomic_sequence_available	tick box whether genomic sequence data are available (true) or not (false)
Reproduction number pathway	character	R_pathway	transmission pathway that reproduction number is based on for vector-borne diseases
Method to estimate R	character	method_R	method used for estimation of the reproduction number - see dropdown list
Other delay start point	character	other_delay_start	start point for delays not in the parameter type dropdown list (e.g., delay from ... to ...)
Other delay end point	character	other_delay_end	end point for delays not in the parameter type dropdown list (e.g., delay from ... to ...)
Numerator	integer	cfr_ifr_numerator	numerator of either cfr/ifr (deaths) or seroprevalence (number seropositive) estimates
Denominator	integer	cfr_ifr_denominator	denominator of either cfr/ifr (cases) or seroprevalence (number tested) estimates
Is the cfr/ifr estimate adjusted?	character	cfr_ifr_method	is the cfr/ifr estimate adjusted, unadjusted, or unspecified - see dropdown list
Is the seroprevalence estimate adjusted?	character	serop_method	is the seroprevalence estimate adjusted (accounting for assay's sensitivity and specificity) - see dropdown list
Outcome for risk factor(s)	character	riskfactor_outcome	outcome for risk factor(s) - see dropdown list
Risk factor name	character	riskfactor_name	risk factor name - see dropdown list
Risk factor occupation	character	riskfactor_occupation	if risk factor is an occupation, then specified occupation as risk factor - see dropdown list

Risk factor adjusted	character	riskfactor_adjusted	adjustment status of risk factor(s) - see dropdown list
Risk factor significant	character	riskfactor_significant	statistical significance of risk factor(s) - see dropdown list

Table A.5: Parameters form fields: refer to epireview package documentation for dropdown options.

A.4.3 Outbreak extraction fields

Data field	Expected type	Variable name	Notes
Article ID	integer	article_id	ID to connect to article form
Outbreak data ID	integer	outbreak_data_id	ID assigned by database
Pre-outbreak baseline	Logical	pre_outbreak_baseline	Dropdown list for whether a baseline period was specified before the outbreak
Outbreak start	Date (day, month, year)	outbreak_start_day, outbreak_start_month, outbreak_start_year	Start date of the outbreak
Outbreak end	Date (day, month, year)	outbreak_end_day, outbreak_end_month, outbreak_end_year	End date of the outbreak
Outbreak ongoing	Logical	outbreak_ongoing	Tick if the outbreak was ongoing at time of reporting
Duration (months)	Numeric	outbreak_duration_months	Duration of the outbreak in months
Outbreak country	Character	outbreak_country	Country where the outbreak occurred (dropdown list)
Outbreak location	Character	outbreak_location	Subnational region or city of the outbreak
Number of cases confirmed	Integer	cases_confirmed	Total confirmed cases
Mode detection	Character	mode_detection	How a case was detected (dropdown list)
Number of cases suspected	Integer	cases_suspected	Total suspected cases
Number of unspecified cases	Integer	cases_unspecified	Number of cases without classification
Number of asymptomatic cases	Integer	cases_asymptomatic	Number of reported asymptomatic infections
Asymptomatic transmission described	Logical	asymptomatic_transmission	Tick if asymptomatic transmission was described
Number of severe cases	Integer	cases_severe	Total number of severe cases
Number of deaths	Integer	cases_deaths	Total number of deaths due to the outbreak
Population size of geographical area	Integer	population_size	Total population of the affected area
Type of cases disaggregated by sex	Character	sex_disaggregation_type	Type of sex disaggregation (dropdown list)
Male cases	Integer	male_cases	Number of male cases
Proportion of cases in males	Numeric	male_proportion	Proportion of cases that are male
Female cases	Integer	female_cases	Number of female cases
Proportion of cases in females	Numeric	female_proportion	Proportion of cases that are female

Table A.6: Outbreak form fields: refer to epireview package documentation for dropdown options.

B Supplementary results

B.1 Quality assessment results

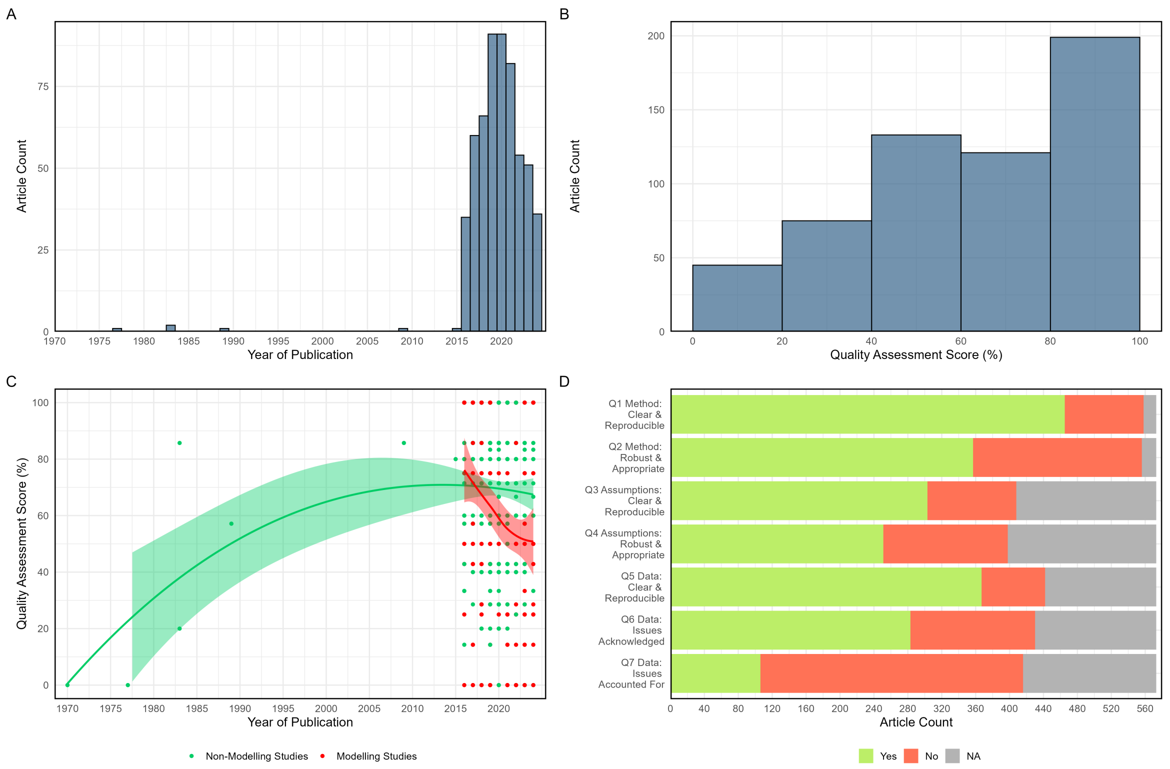

Figure B.2: (A) Article count by year of publication. (B) Article count by quality assessment score (defined as the proportion of "Yes" answers relative to the sum of "Yes" and "No" answers for each paper, excluding non-applicable questions). (C) Quality assessment score by year of publication (time trends for articles with and without transmission models fitted via local polynomial regression). (D) Article count for each quality assessment question scoring 'Yes', 'No' or 'Non-Applicable'.

B.2 Summary of extracted models and parameters

Figures B.3 and B.4 present an overview of the types of parameters and models extracted in the review, respectively.

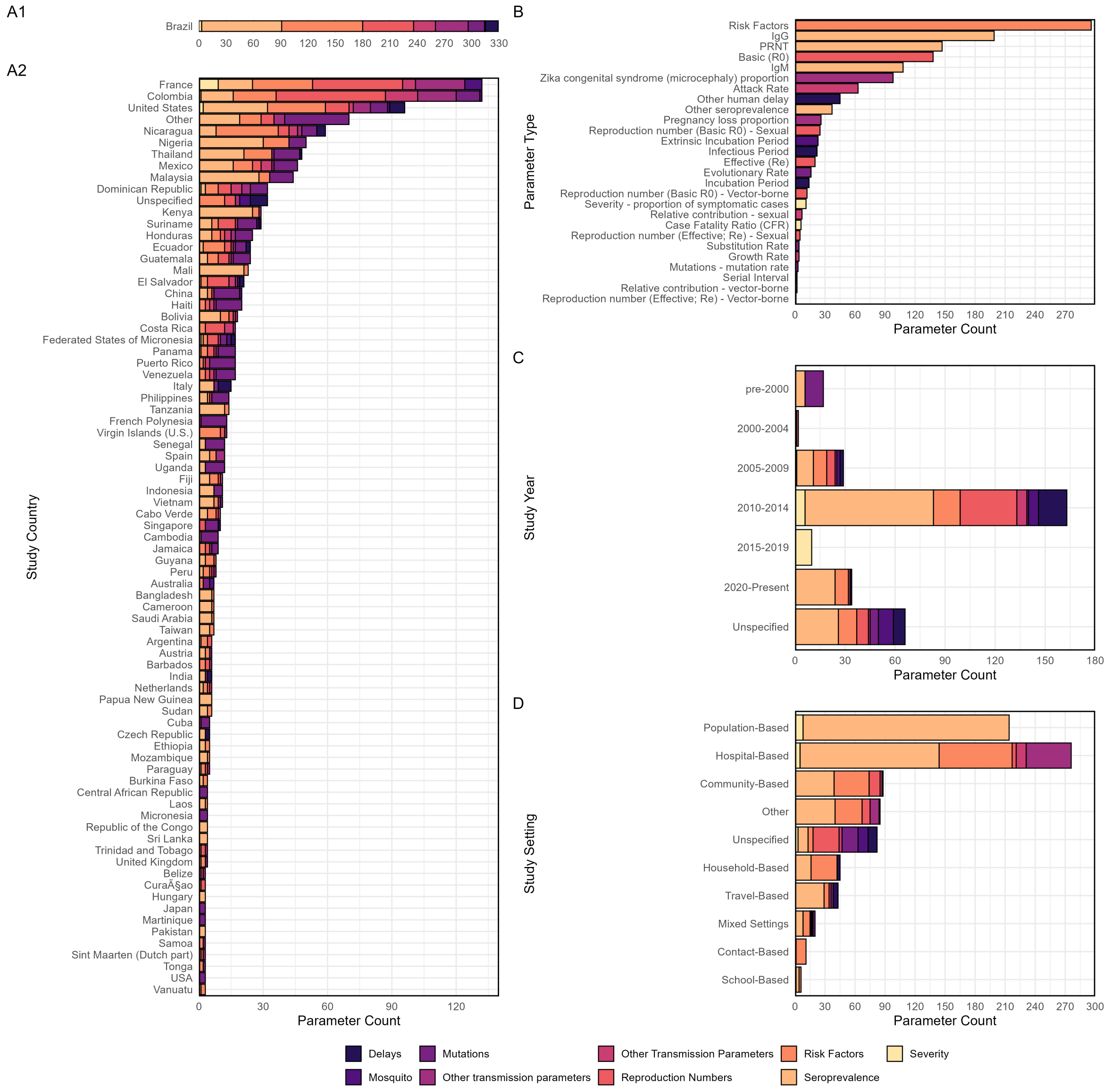

Figure B.3: Summary of extracted parameters by country, parameter type, study year, and study setting. Brazil is separate because of the large number of parameters compared to the other countries. Countries with less than three estimates are grouped into the "Other" label in Panel A2.

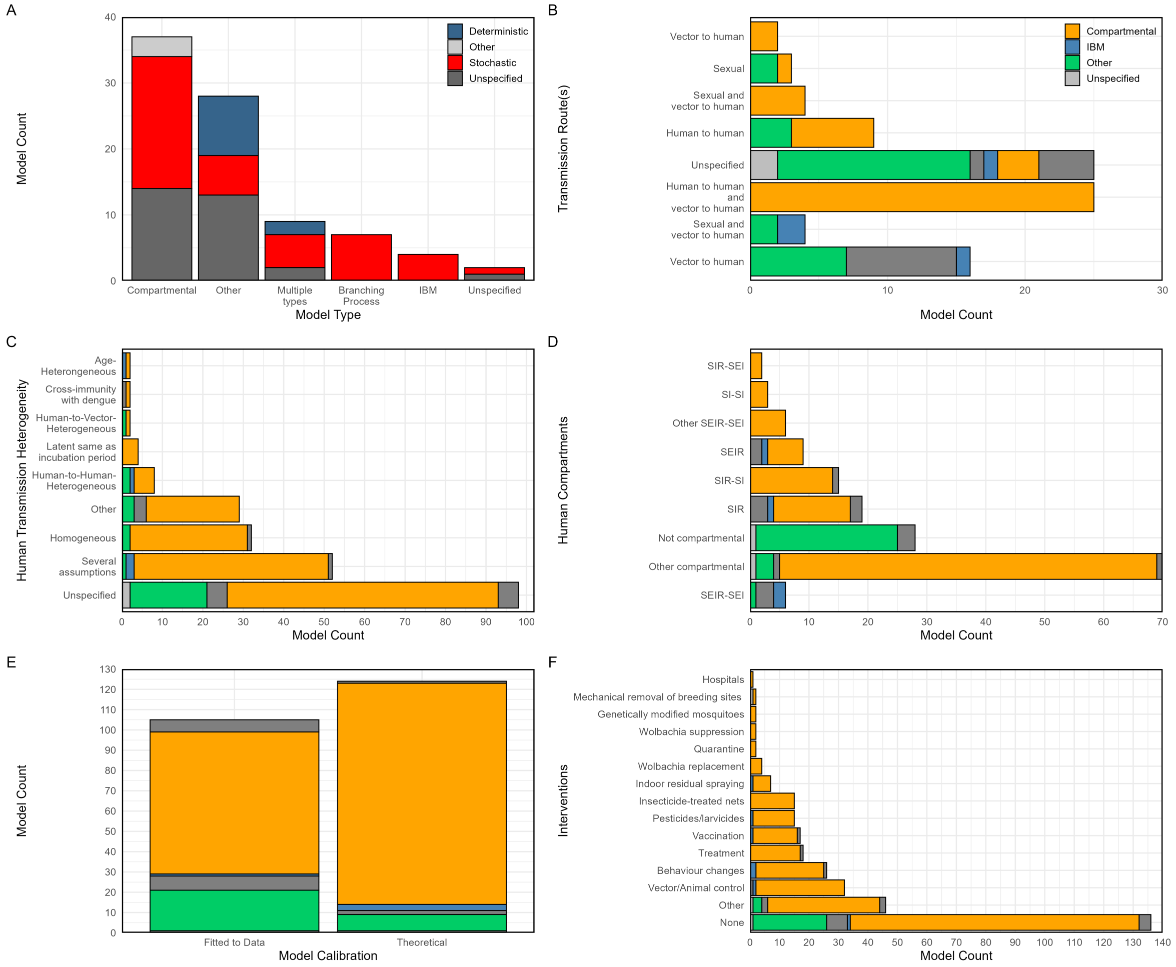

Figure B.4: Summary of extracted models by model type, transmission route, assumptions, compartments, calibration type, and interventions.

B.3 Additional plots of parameters

Unless otherwise specified, data in all plots within this section have been filtered to studies with a QA score of at least 50%.

B.3.1 Basic reproduction numbers

The basic reproduction number (R_0) represents the average number of secondary infections generated by a case in a fully susceptible population. The basic vector-borne reproduction number (R_0^v) represents the average number of new infections generated via vector-borne transmission in a fully susceptible population, and the basic sexual reproduction number (R_0^h) represents the average number of new cases generated via sexual transmission in a fully susceptible population.

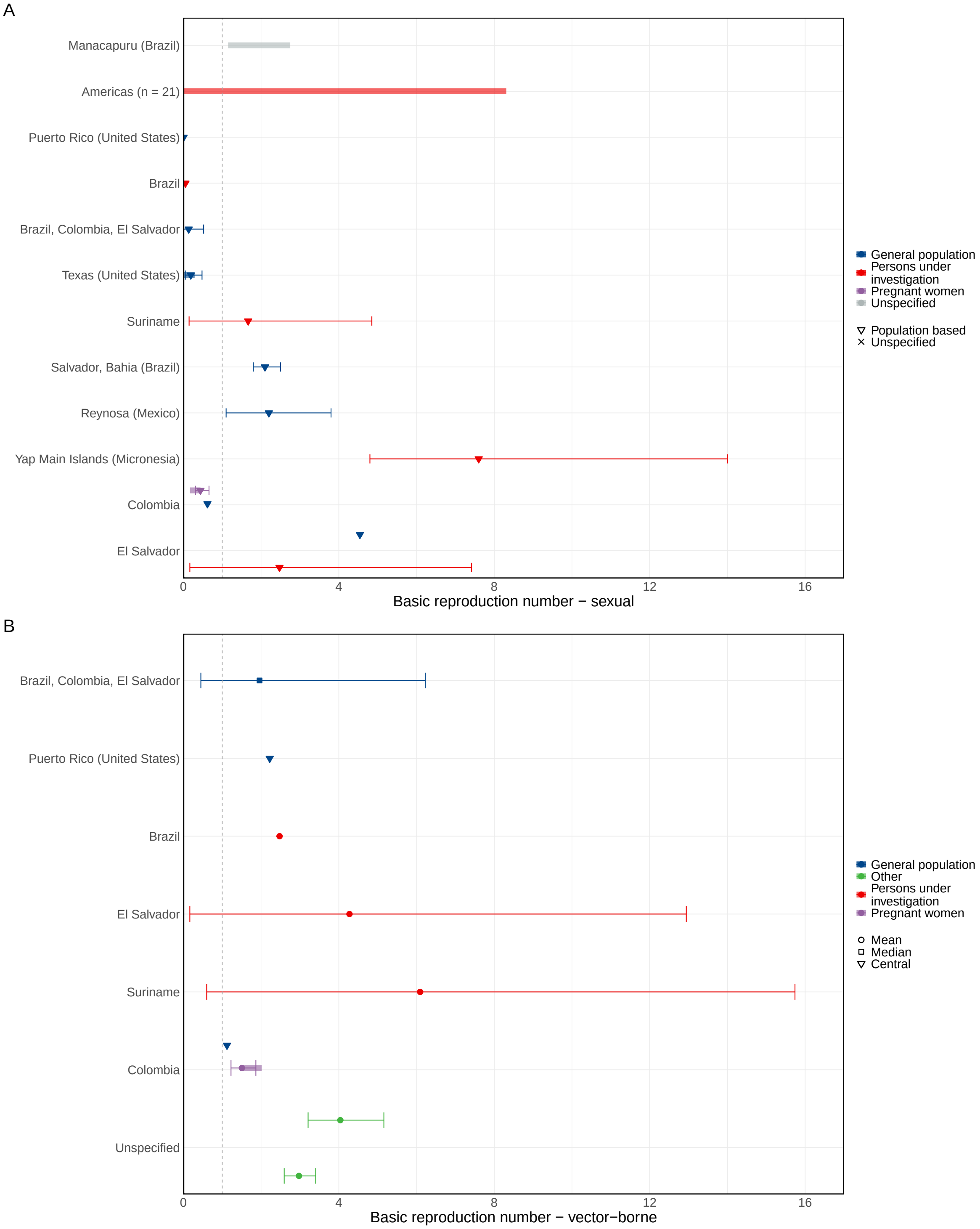

Figure B.5: Basic sexual reproduction numbers (R_0^h) (A), and basic vector-borne reproduction numbers (R_0^v) (B). Points are central estimates reported in the published studies (defined as either mean, median or unspecified central as estimated in the extracted paper), error bars are 95% confidence or credible intervals, thicker shaded bars are ranges of central estimates over disaggregated groups. The grey vertical dotted line marks 1. When multiple estimates for the same location were available, the estimates were jittered. Note: the plot of overall basic reproduction numbers from studies with high QA scores is Figure 3 in the main text. The sample sizes for each study shown in the figure are reported in Table B.12 of the Supplementary Information.

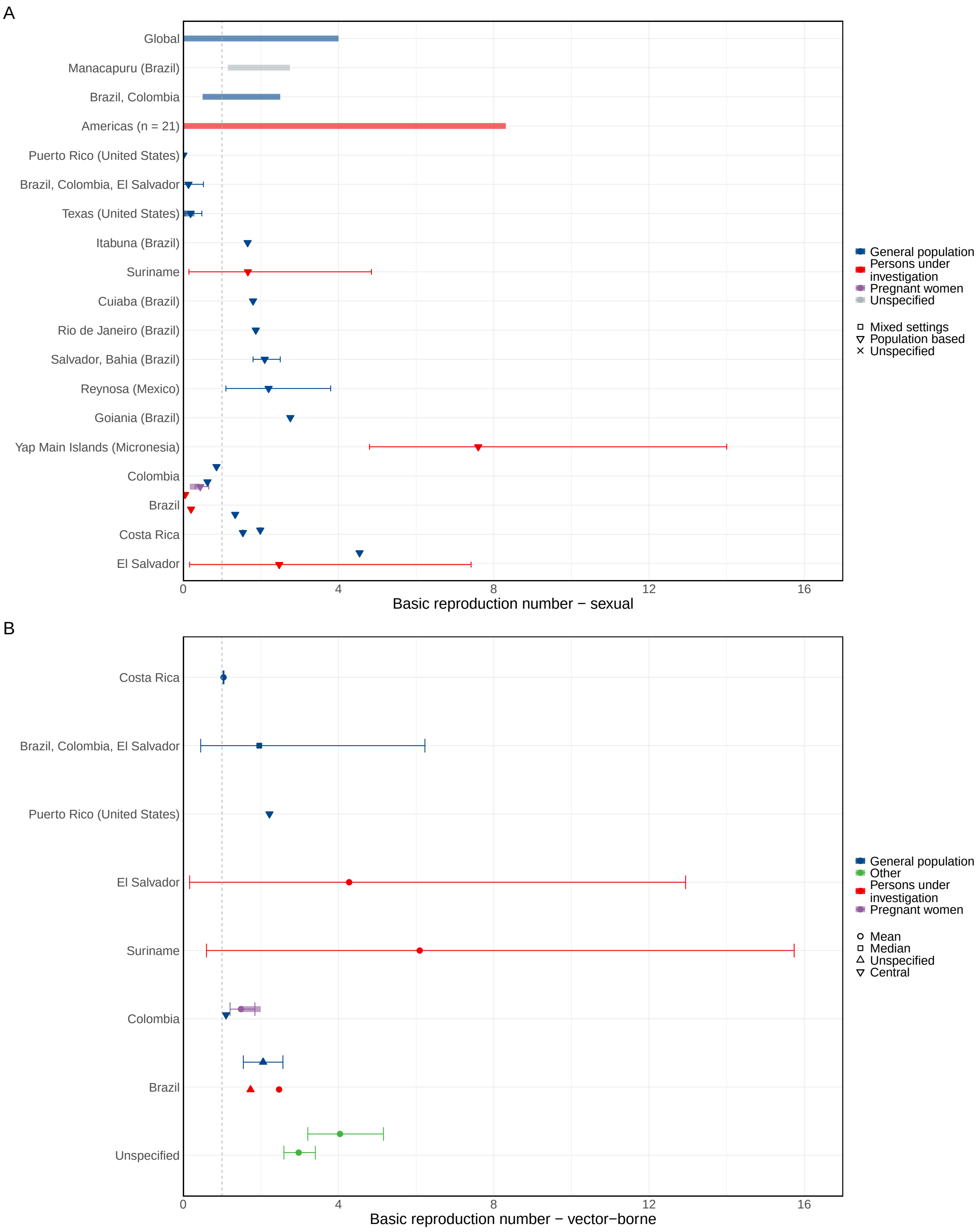

Figure B.6: Basic sexual reproduction numbers (R_0^h) (A), and basic vector-borne reproduction numbers (R_0^v) (B). Points are central estimates reported in the published studies (defined as either mean, median or unspecified central as estimated in the extracted paper), error bars are 95% confidence or credible intervals, thicker shaded bars are ranges of central estimates over disaggregated groups. The grey vertical dotted line marks 1. When multiple estimates for the same location were available, the estimates were jittered. Note: the plot of overall basic reproduction numbers from all studies (regardless of QA score) is Figure figure B.26. The sample sizes for each study shown in the figure are reported in Supplementary Table B.12.

B.3.2 Effective reproduction numbers

The effective reproduction number (R_e) represents the average number of secondary infections generated by a case in a partially susceptible population. The basic vector-borne reproduction number (R_e^v) represents the average number of new infections generated via vector-borne transmission in a partially susceptible population, and the effective sexual reproduction number (R_e^h) represents the average number of new cases generated via sexual transmission in a partially susceptible population.

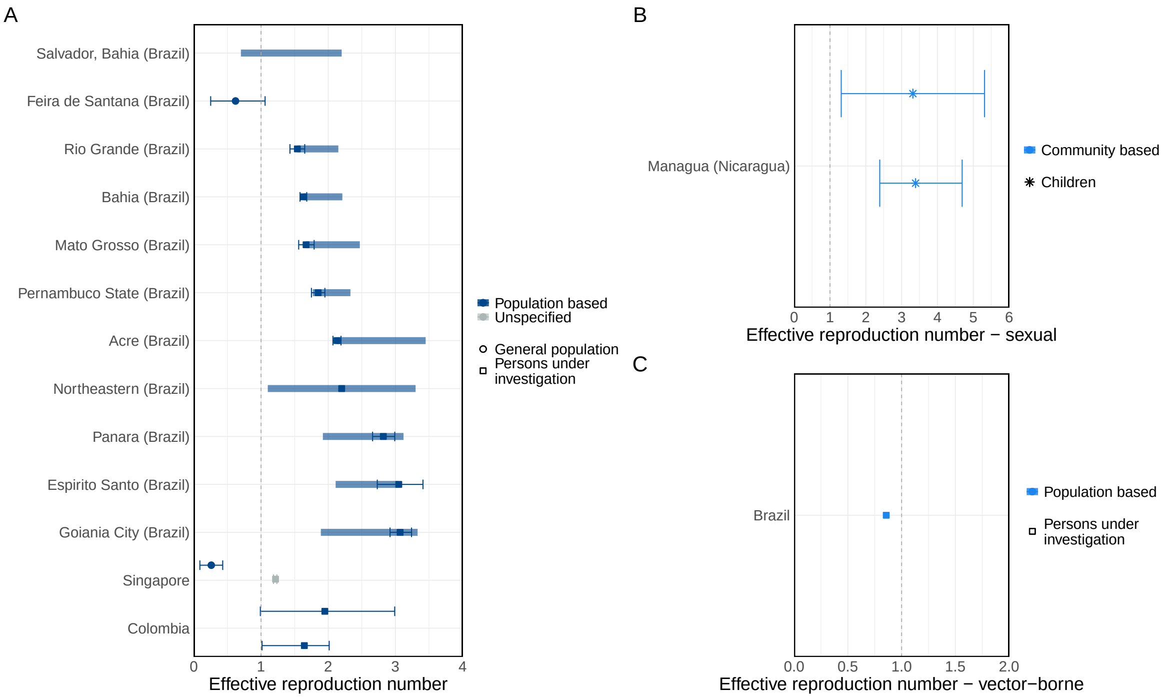

Figure B.7: Effective reproduction numbers, overall (R_e) (A), sexual (R_e^h) (B), and vector-borne (R_e^h) (C). Points are central estimates reported in the published studies (defined as either mean, median or unspecified central as estimated in the extracted paper), error bars are 95% confidence or credible intervals, thicker shaded bars are ranges of central estimates over disaggregated groups. The grey vertical dotted line marks 1. When multiple estimates for the same location were available, the estimates were jittered. The sample sizes for each study shown in the figure are reported in Supplementary Table B.12.

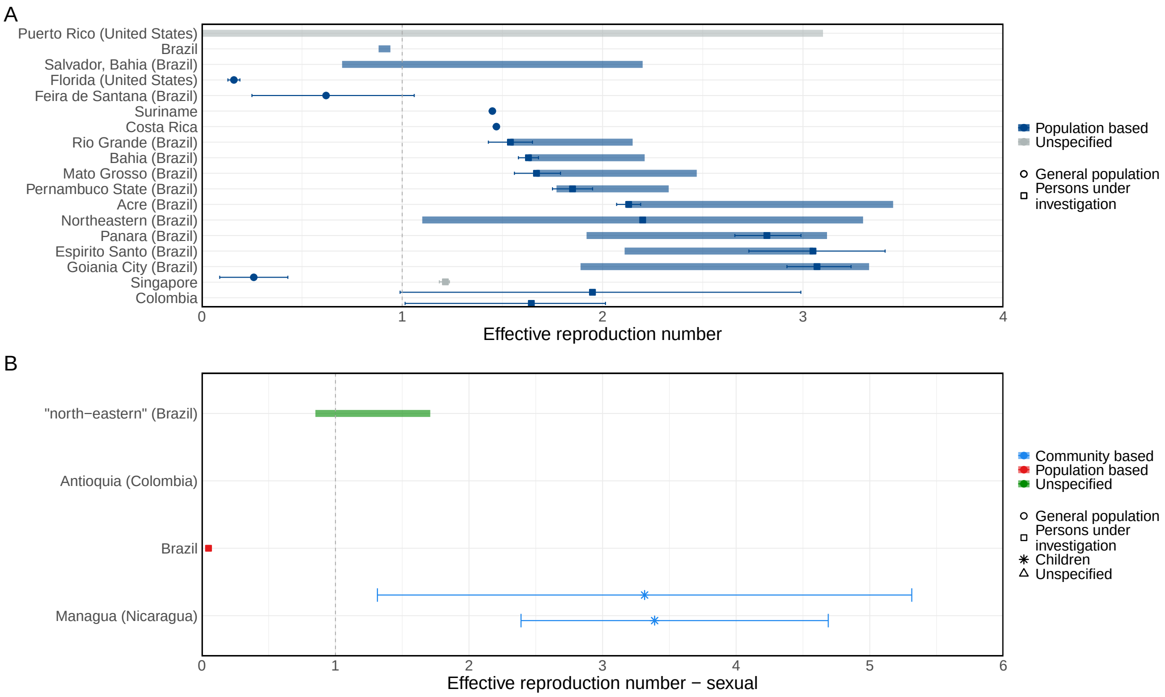

Figure B.8: Effective reproduction numbers, overall (R_e) (A), sexual (R_e^h) (B), and vector-borne (R_e^h) (C). Points are central estimates reported in the published studies (defined as either mean, median or unspecified central as estimated in the extracted paper), error bars are 95% confidence or credible intervals, thicker shaded bars are ranges of central estimates over disaggregated groups. The grey vertical dotted line marks 1. When multiple estimates for the same location were available, the estimates were jittered. This figure includes all studies regardless of QA score. The sample sizes for each study shown in the figure are reported in Supplementary Table B.12.

B.3.3 Seroprevalence

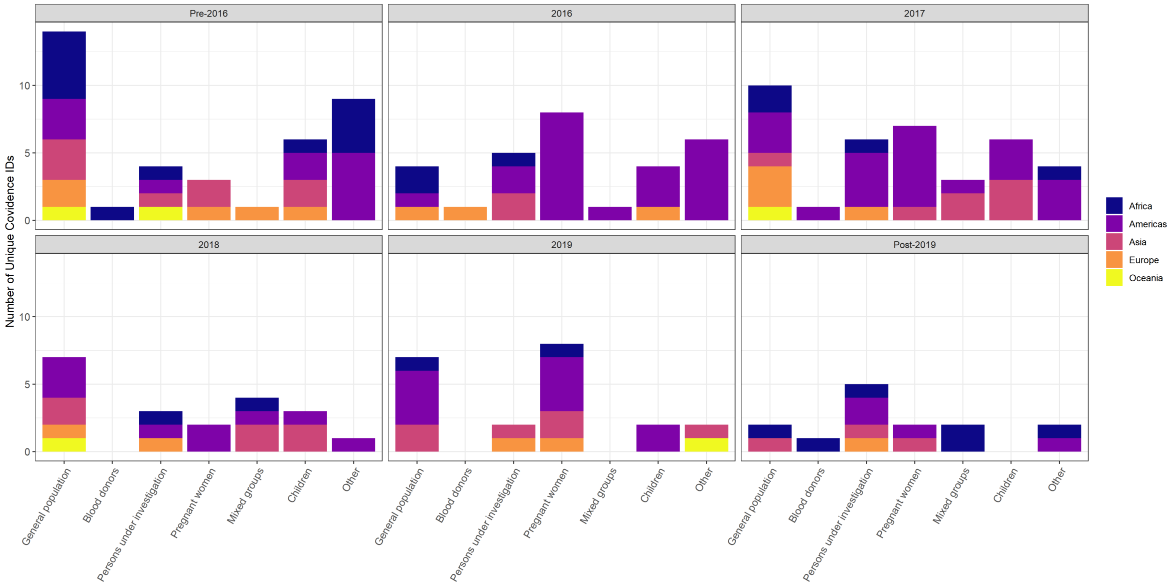

Figure B.9: Number of seroprevalence-related publications by population group. Number of unique studies in each population group, coloured by continent of the sero-survey.

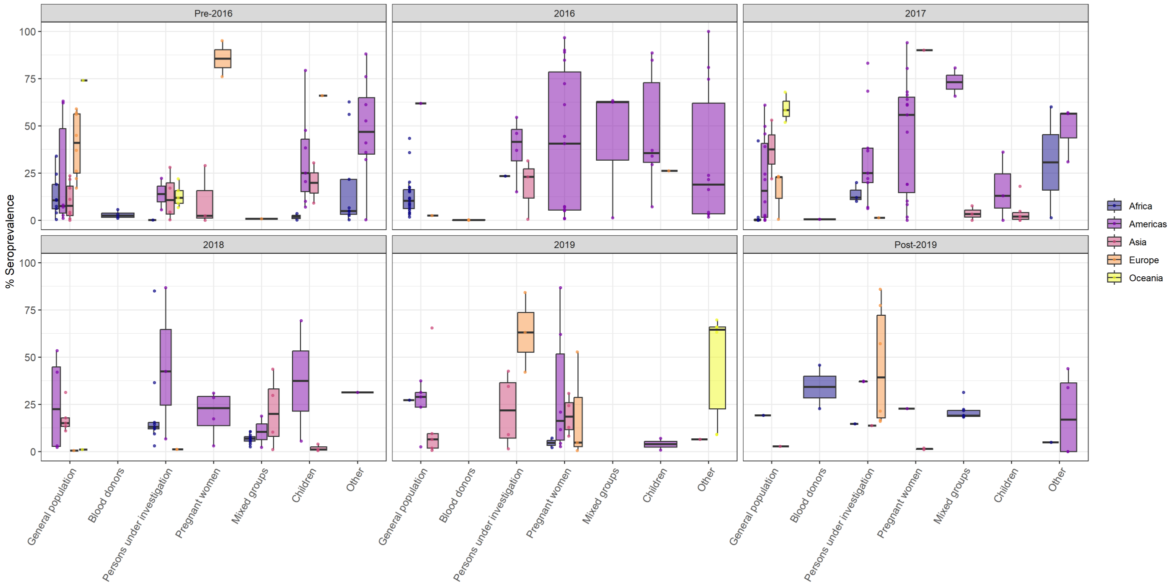

Figure B.10: Seroprevalence estimate by population group. Distribution of seroprevalence estimates in each population group, coloured by continent of the sero-survey. The sample sizes for each study shown in the figure are reported in Supplementary Table B.10.

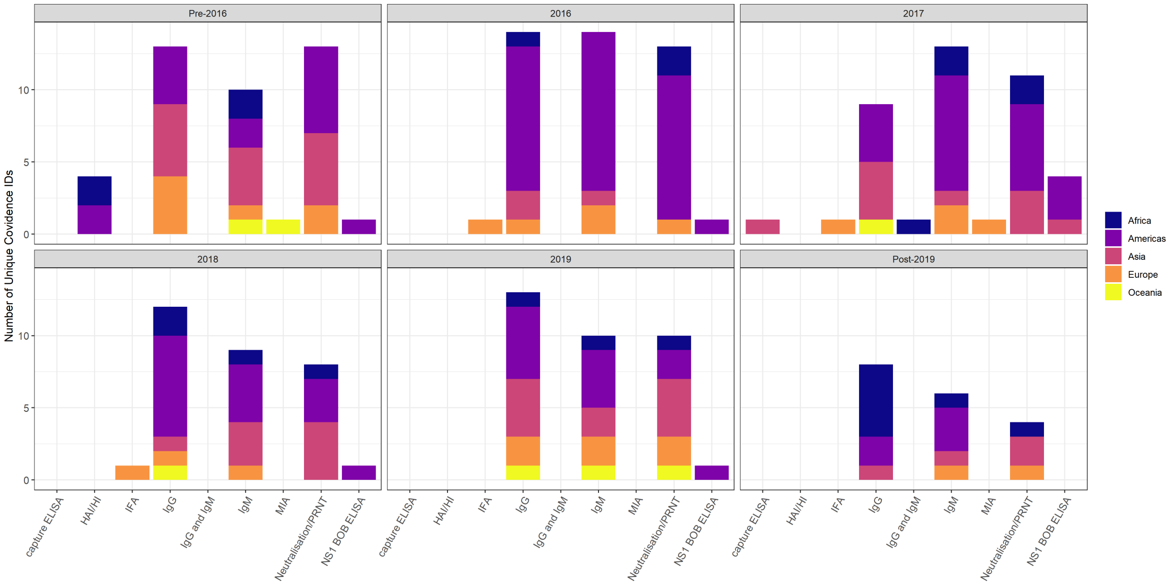

Figure B.11: Seroprevalence by assay. Number of unique studies for each type of assay, coloured by continent of the sero-survey.

B.3.4 Genomic parameters

We extracted 15 evolutionary rates and 3 substitution rates from 15 studies, of which 3 were for Brazil, 1 for the United States, while the majority came from multi-country analyses including countries in Europe, the Americas, Asia, Africa and Oceania (n=14). Figures B.12 and B.13 and Table B.13 summarise these estimates across different sampling intervals (in years). The evolutionary rate estimates tended to decrease as the variance in sampling dates increased, but most values clustered around 1×10^{-3} substitutions per site per year, with substitution rates showing higher uncertainty at longer sampling intervals.

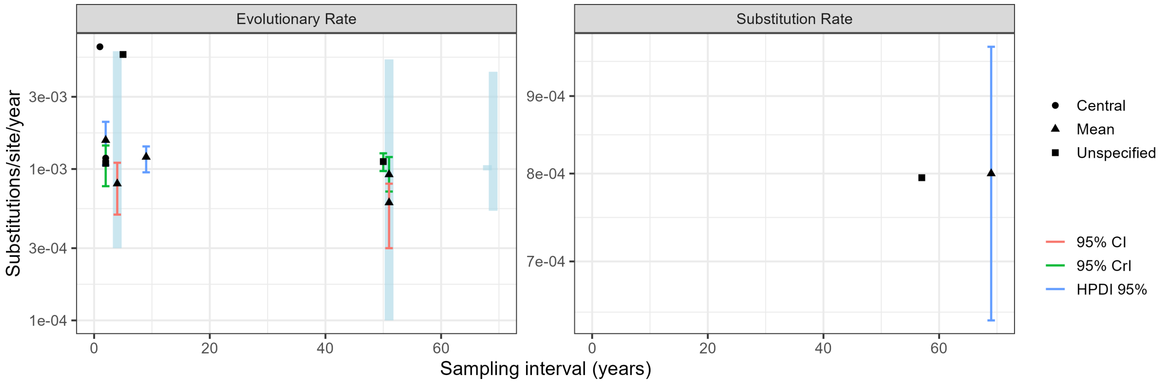

Figure B.12: Genomic information reported in studies with high QA scores. Points are central estimates (defined as either mean, median or unspecified central as estimated in the extracted paper), solid lines are confidence, credible, or high posterior density intervals, and shaded segments are ranges of central estimates across multiple groups. The sample sizes for each study shown in the figure are reported in Supplementary Table B.13.

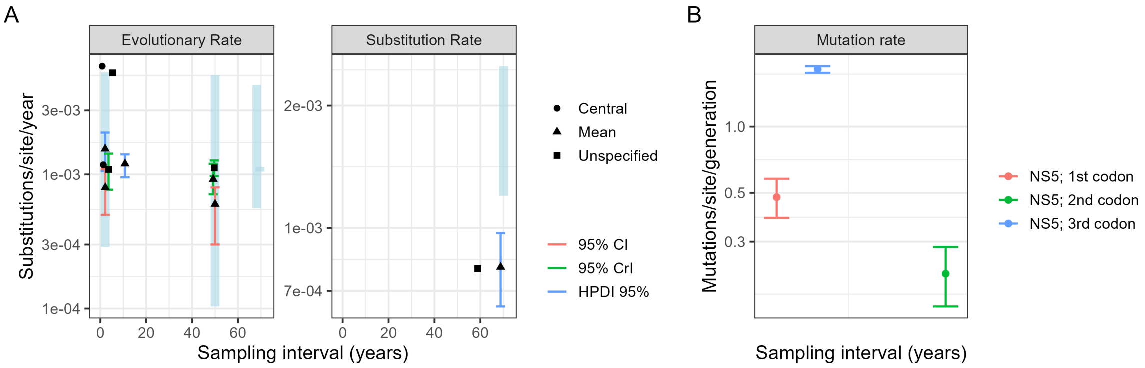

Figure B.13: Genomic information from all included studies. Points are central estimates (defined as either mean, median or unspecified central as estimated in the extracted paper), solid lines are confidence, credible, or high posterior density intervals, and shaded segments are ranges of central estimates across multiple groups. The sample sizes for each study shown in the figure are reported in Supplementary Table B.13.

B.3.5 Pregnancy loss proportion

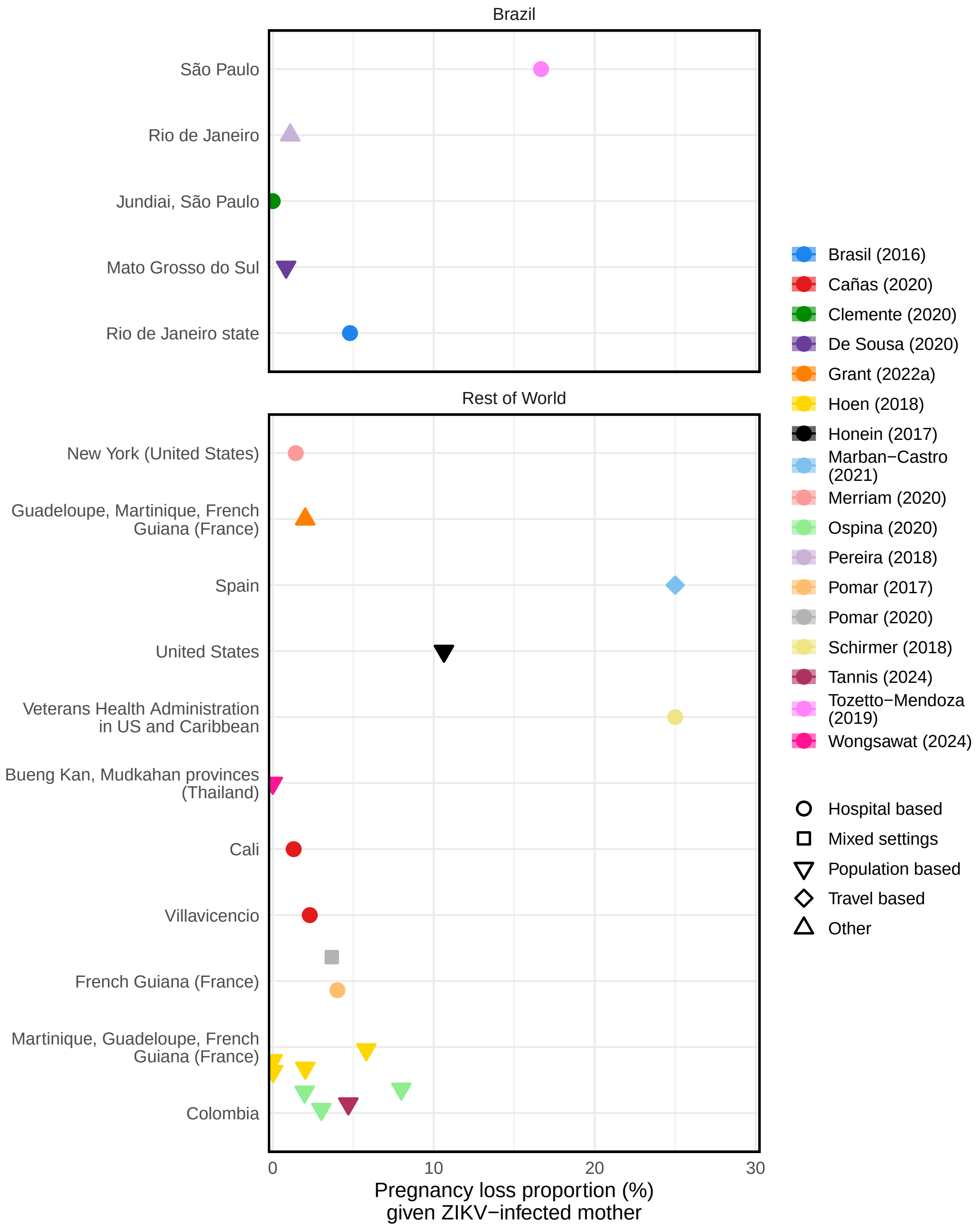

Figure B.14: Estimates of reported pregnancy loss probabilities. The top panel shows estimates from Brazil, while the bottom panel shows estimates from the rest of the world. Points are central estimates (defined as either mean, median or unspecified central as estimated in the extracted paper), solid lines are confidence or credible intervals, and shaded segments are ranges of central estimates across multiple groups. The sample sizes for each study shown in the figure are reported in Supplementary Table B.16.

B.3.6 Severity

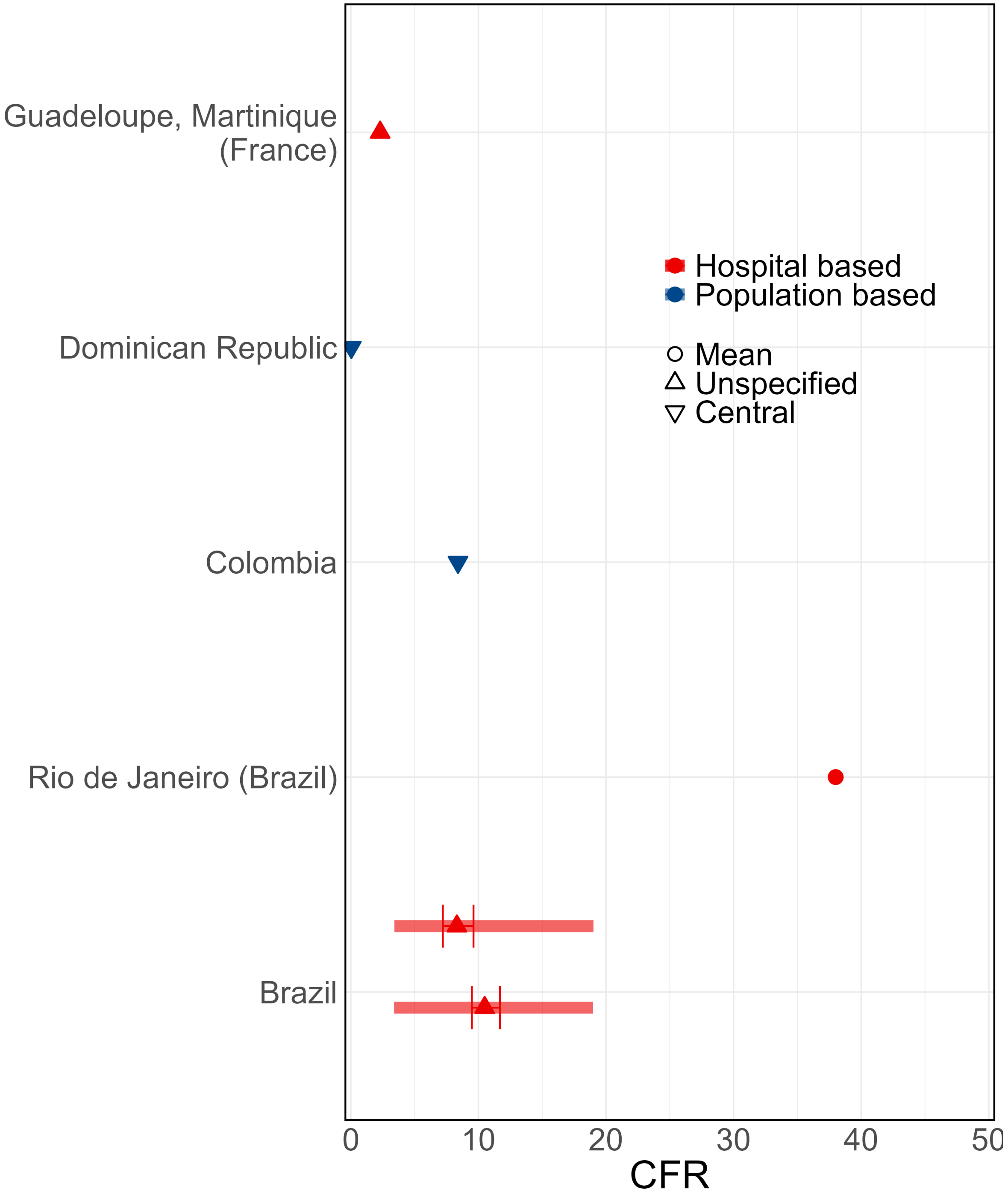

Figure B.15: Case fatality ratio estimates from all included studies, which all had high QA scores. Points are central estimates (defined as either mean, median or unspecified central as estimated in the extracted paper), solid lines are confidence or credible intervals, and shaded segments are ranges of central estimates across multiple groups. The sample sizes for each study shown in the figure are reported in Supplementary Table B.14.

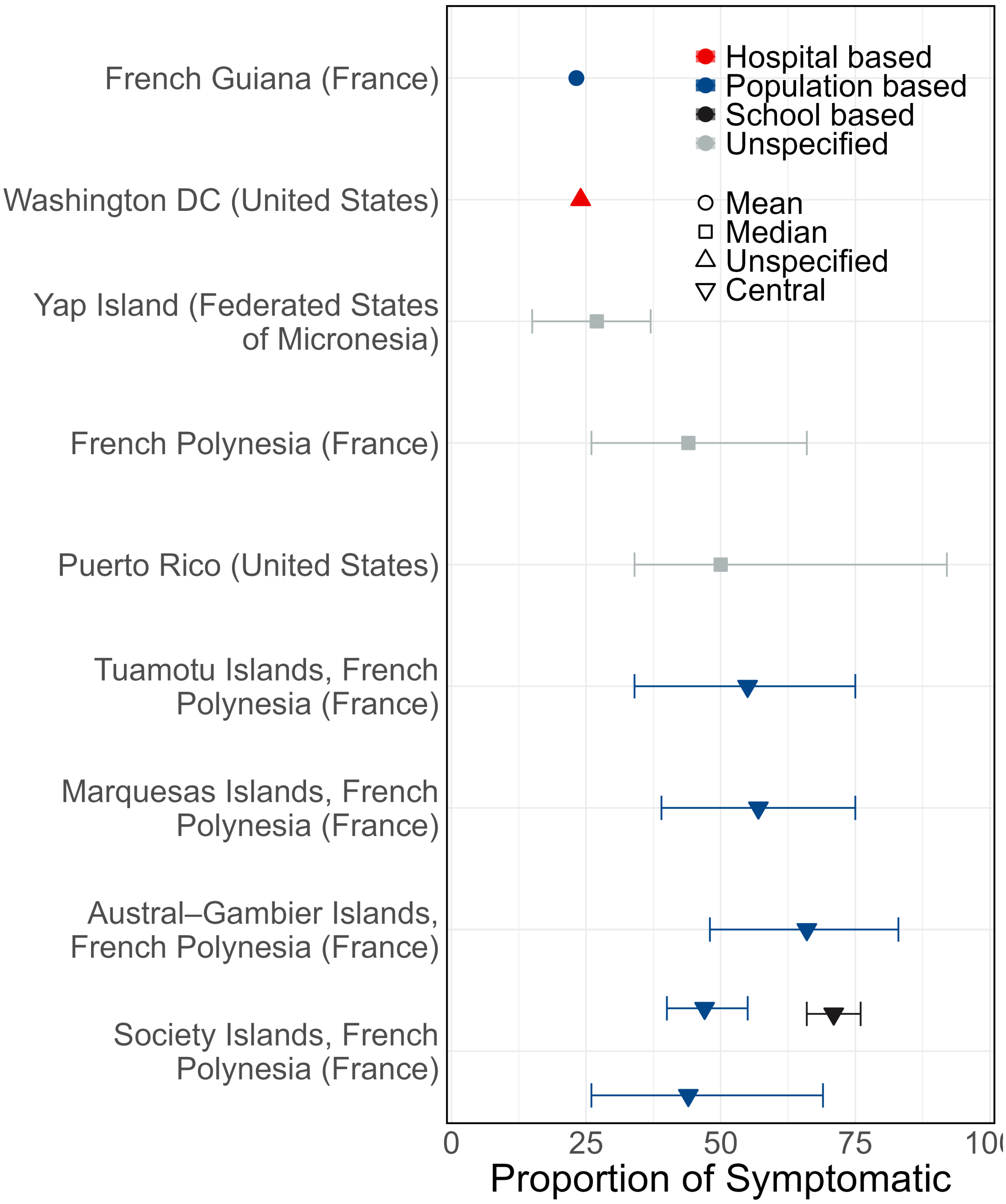

Figure B.16: Proportion of cases that are symptomatic from all included studies. Points are central estimates (defined as either mean, median or unspecified central as estimated in the extracted paper), solid lines are confidence or credible intervals, and shaded segments are ranges of central estimates across multiple groups. The sample sizes for each study shown in the figure are reported in Supplementary Table B.14.

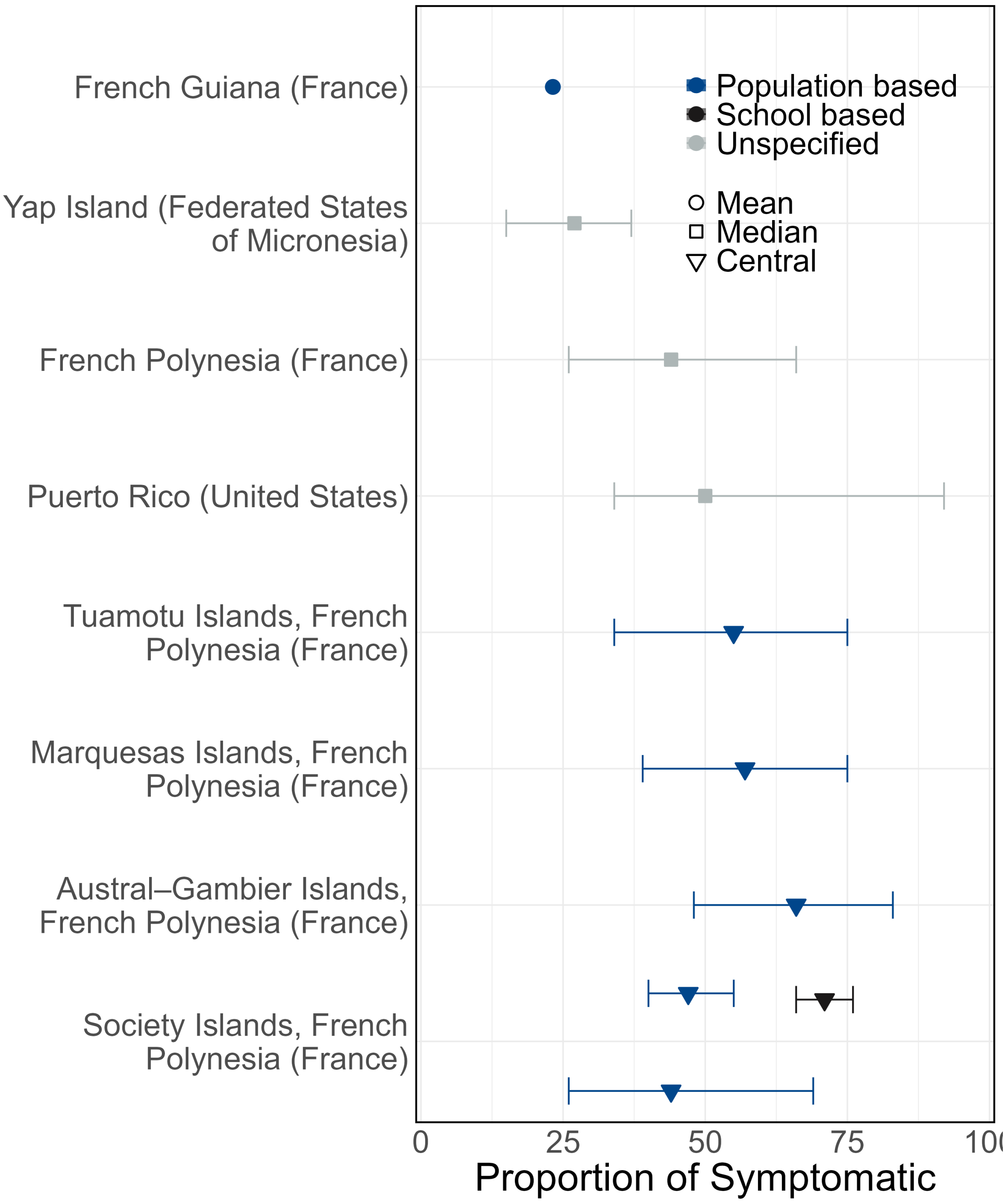

Figure B.17: Proportion of cases that are symptomatic from studies with high QA scores. Points are central estimates (defined as either mean, median or unspecified central as estimated in the extracted paper), solid lines are confidence or credible intervals, and shaded segments are ranges of central estimates across multiple groups. The sample sizes for each study shown in the figure are reported in Supplementary Table B.14.

B.3.7 Other transmission parameters

We considered estimates of the attack rate or final size of the epidemic. Of the 18 studies reporting estimates of the attack rate (Figures B.18 and B.19, Table B.12), we extracted a total of 51 estimates reported from Micronesia, Philippines, French Polynesia, Cabo Verde and several countries of Central and South America. The central estimates of the attack rate ranged from 0% in the Federal District of Mexico in 2015-2018 [9] to 94% estimated by a modelling study in French Polynesia in 2013-2014, [10] underscoring the variability in ZIKV transmission and burden across geographic regions.

In addition, we identified estimates of the growth rate (Figure B.20) and of the contribution of sexual transmission to overall ZIKV spread (Figures B.21 and B.22, Table B.12). Two studies in Colombia quantified the proportion of transmission attributable to sexual transmission, reporting estimates of 15.36% (95%CI 12.83-17.14)50 and 23% (95%CI 1-47%) [11]. We characterised growth rate estimates (n=4) (Supplementary Figure B.20, Table B.12) from 3 studies conducted in Brazil, Colombia, and Nicaragua, with corresponding doubling times spanning from 0.85 (95%CI 0.12-0.85) days in a paediatric cohort in Rio de Janeiro, Brazil [12] to 8.7 (95%CI 8.0-10.5) days in a hospital setting in Barranquilla, Colombia [13].

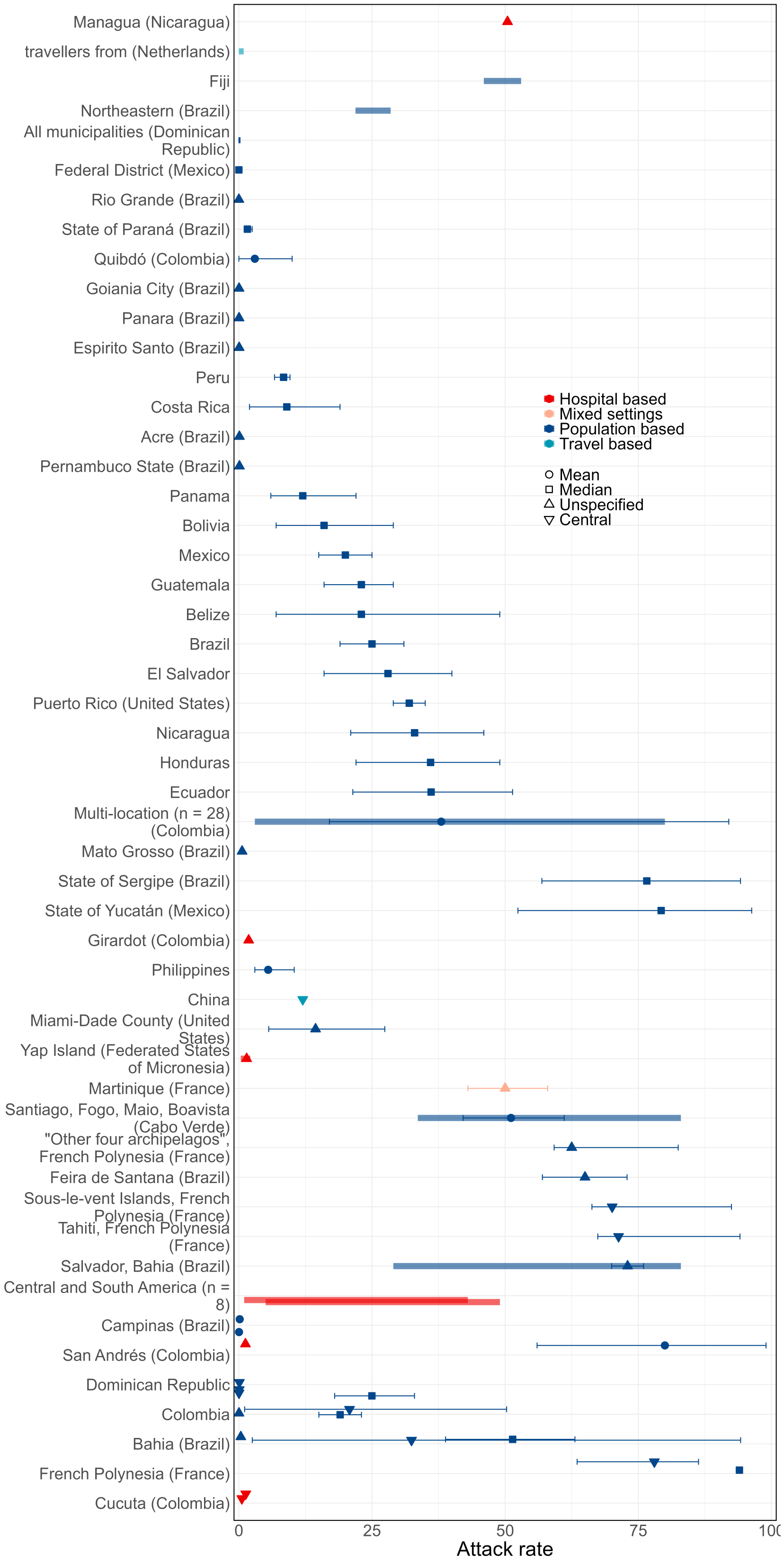

Figure B.18: Attack rate from all included studies. Points are central estimates (defined as either mean, median or unspecified central as estimated in the extracted paper), solid lines are confidence or credible intervals, and shaded segments are ranges of central estimates across multiple groups. The sample sizes for each study shown in the figure are reported in Supplementary Table B.12.

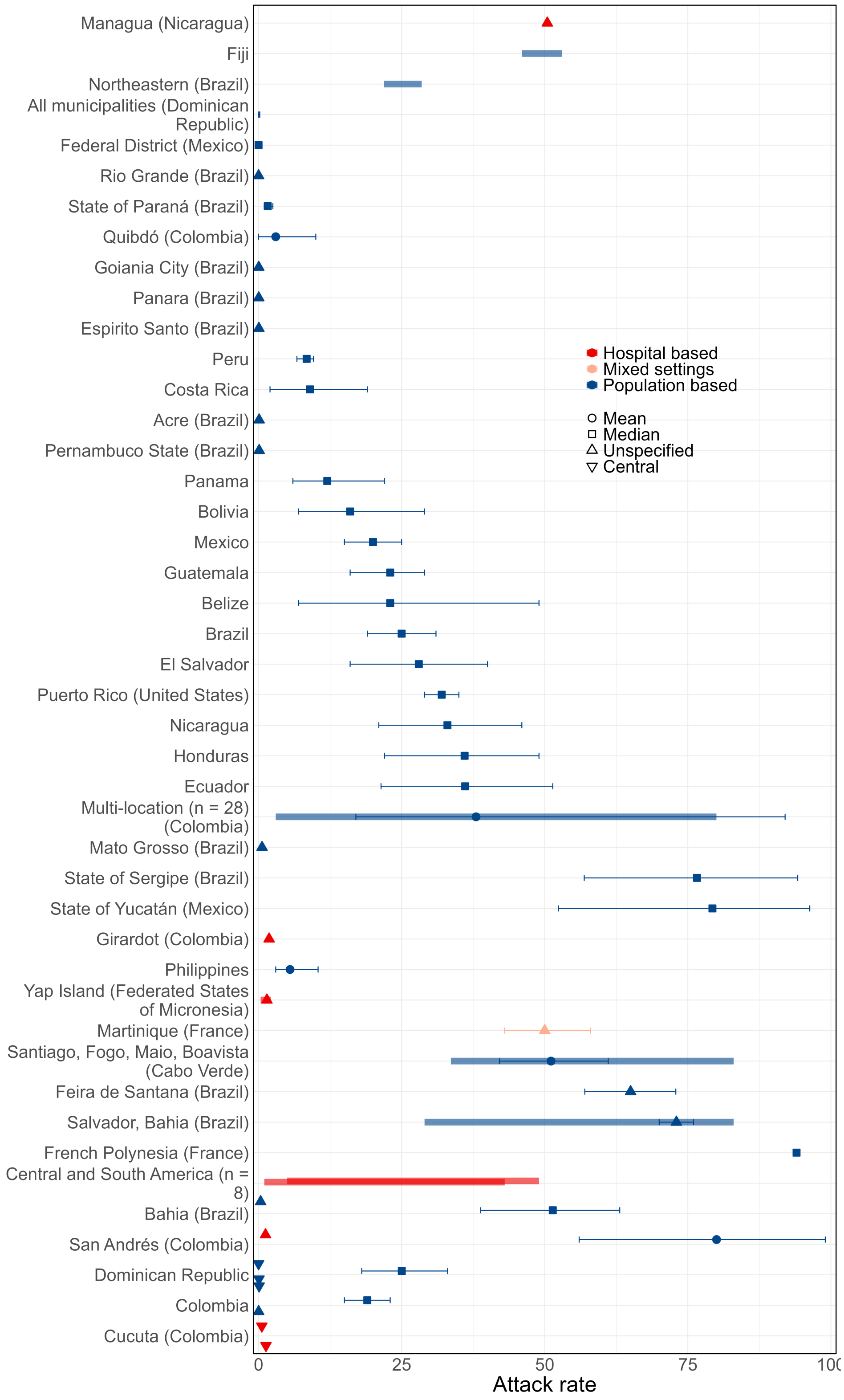

Figure B.19: Attack rate from studies with high QA scores. Points are central estimates (defined as either mean, median or unspecified central as estimated in the extracted paper), solid lines are confidence or credible intervals, and shaded segments are ranges of central estimates across multiple groups. The sample sizes for each study shown in the figure are reported in Supplementary Table B.12.

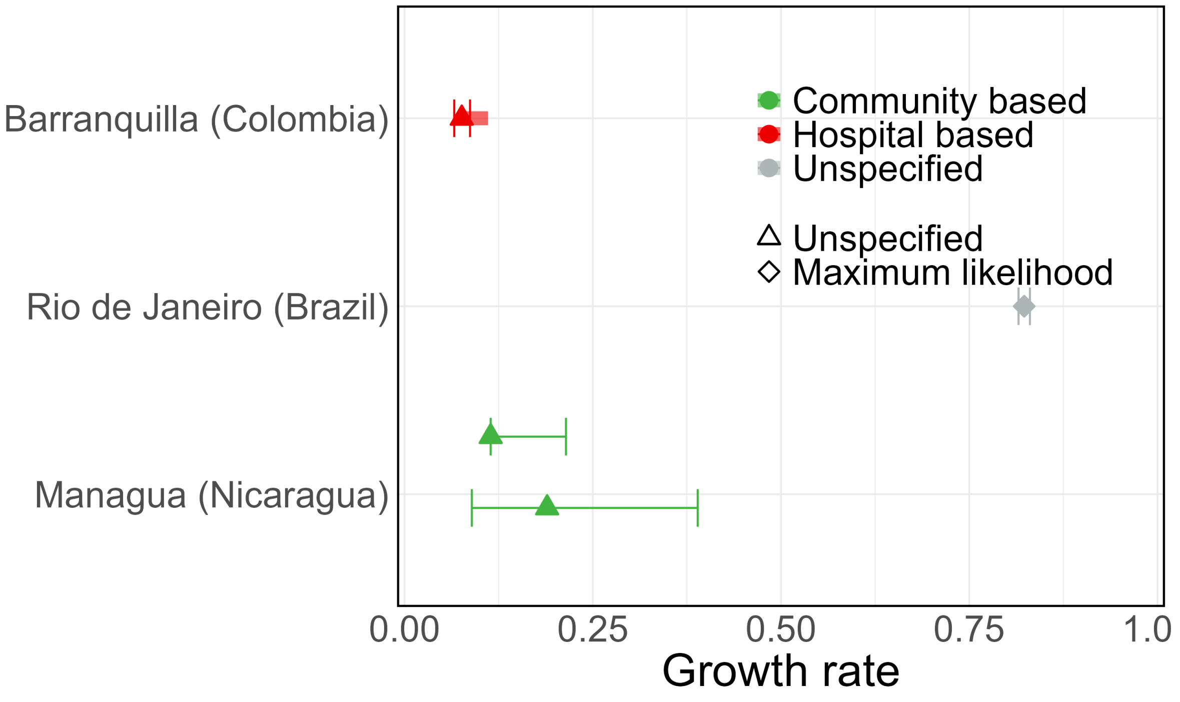

Figure B.20: Growth rate from all included studies, which all had high QA scores. Points are central estimates (defined as either mean, median or unspecified central as estimated in the extracted paper), solid lines are confidence or credible intervals, and shaded segments are ranges of central estimates across multiple groups. The sample sizes for each study shown in the figure are reported in Supplementary Table B.12.

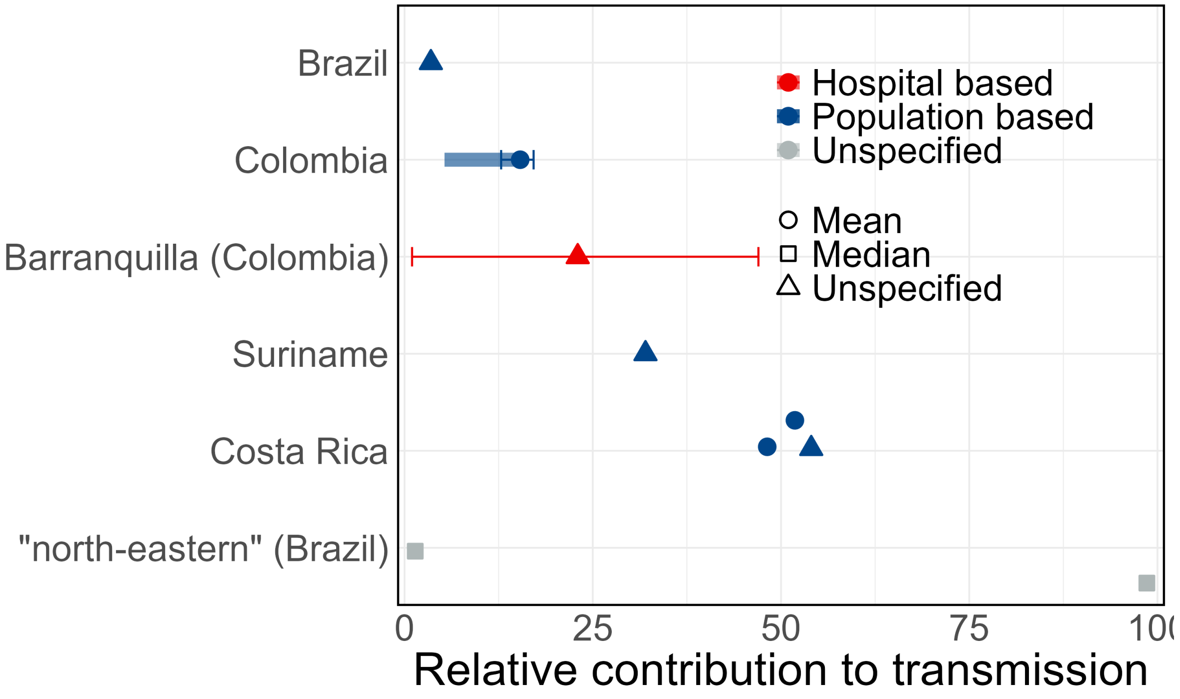

Figure B.21: Sexual relative contribution to transmission from all included studies. Points are central estimates (defined as either mean, median or unspecified central as estimated in the extracted paper), solid lines are confidence or credible intervals, and shaded segments are ranges of central estimates across multiple groups. The sample sizes for each study shown in the figure are reported in Supplementary Table B.12.

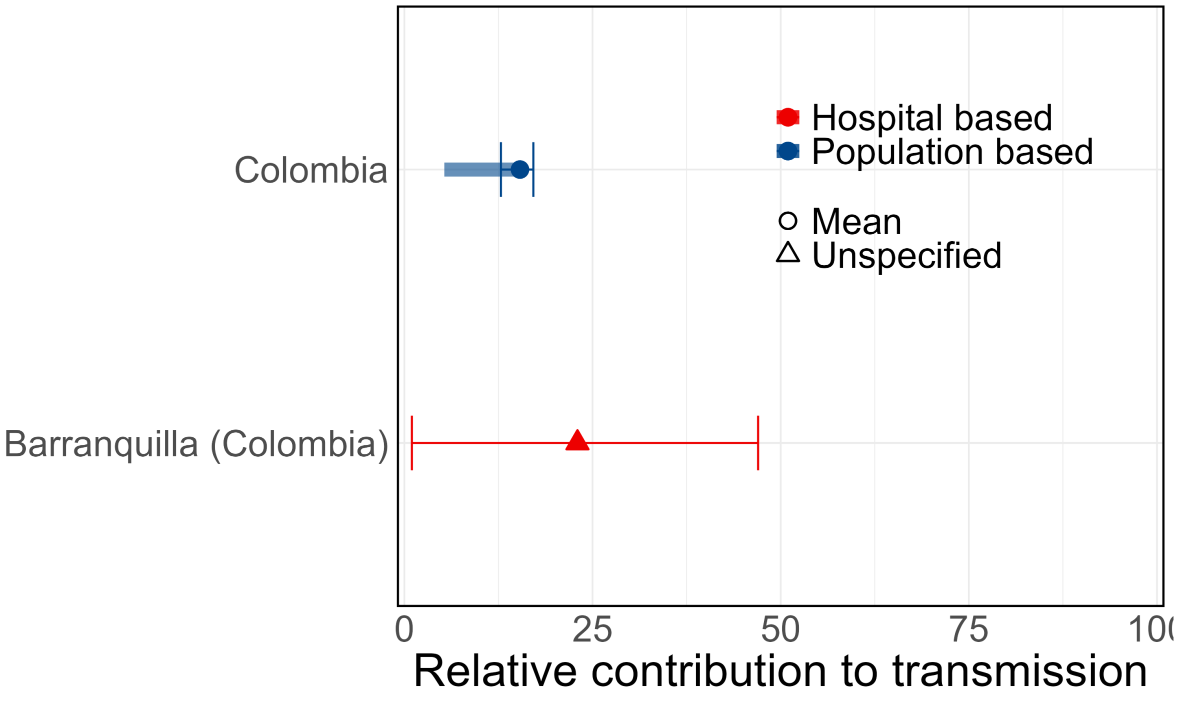

Figure B.22: Sexual relative contribution to transmission from studies with high QA scores. Points are central estimates (defined as either mean, median or unspecified central as estimated in the extracted paper), solid lines are confidence or credible intervals, and shaded segments are ranges of central estimates across multiple groups. The sample sizes for each study shown in the figure are reported in Supplementary Table B.12.

B.3.8 Risk factors

Ten unique risk factor types with 14 different Zika-related outcomes were reported in 137 studies (n=486 entries of 172 unique combinations of risk factor, outcome, statistical significance, and adjustment), of which 415 entries of 157 unique combinations were from 111 studies with quality assessments 50% or higher (Figures B.23 and B.24). ZIKV-related risk factors among the studies wiht high QA scores were most frequently investigated against serological outcomes (n=80) and ZIKV infection (n=65). The most commonly reported risk factors across all outcomes were age (n=87), sex (n=62), and infection during pregnancy (n=40). Most risk factors were reported as both significant and non-significant in different studies (B.11).

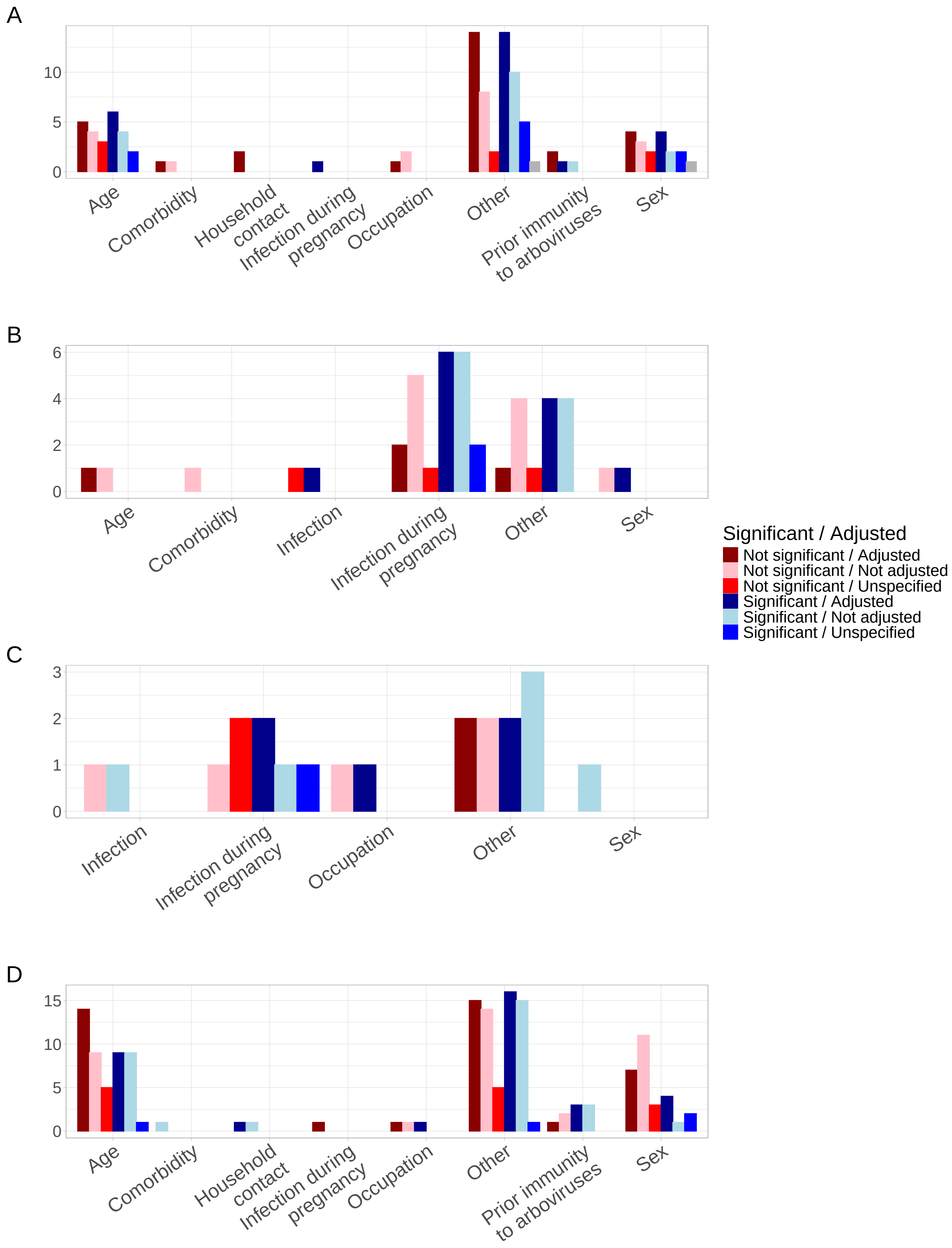

Figure B.23: Commonly reported outcomes and risk factors. A. Infection; B. Zika congenital syndrome; C. Microcephaly; D. Serology

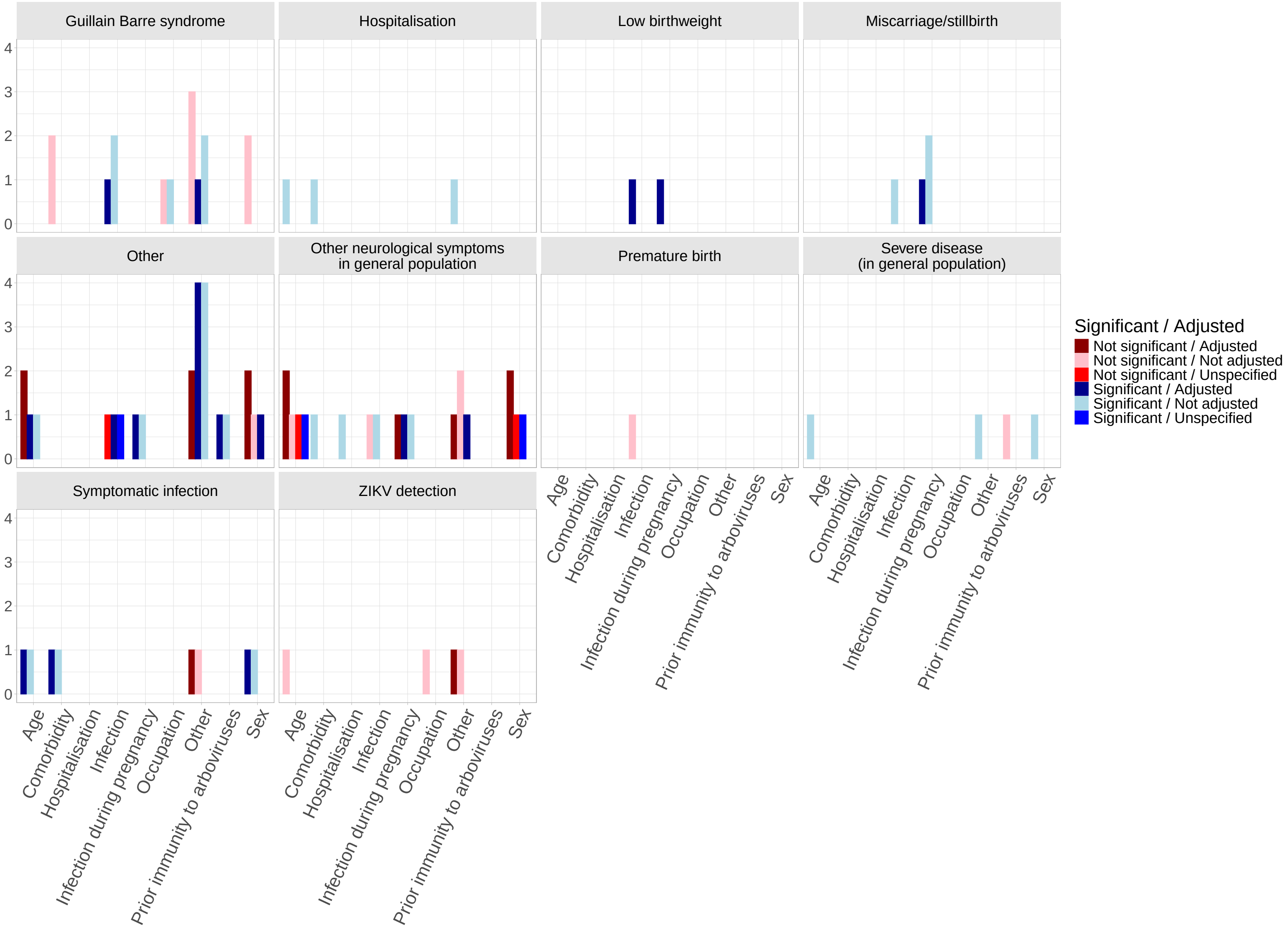

Figure B.24: Other reported outcomes and risk factors

B.4 Main figures with no QA filtering

Below, we present analogous versions of Figures 2-5 from the main text, but without filtering for studies with high QA scores.

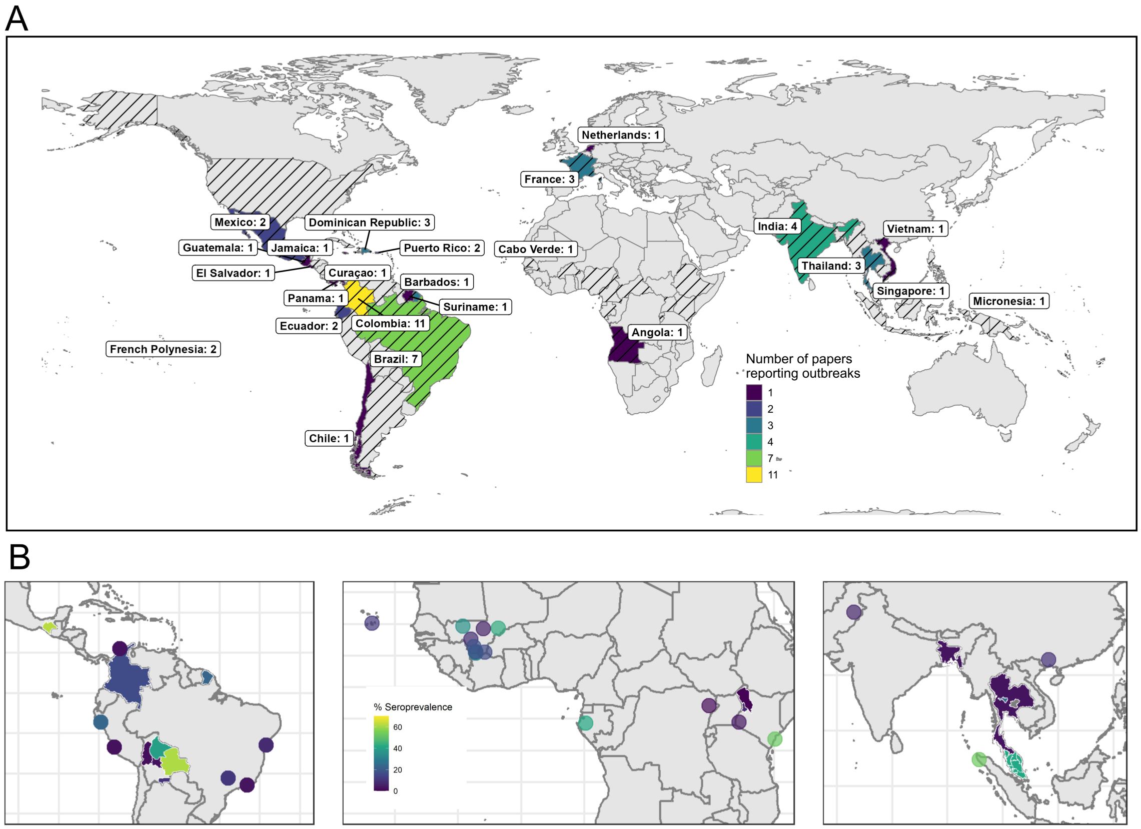

Figure B.25: ZIKV outbreak and seroprevalence mapping from all included studies: (A) Countries with papers reporting ZIKV outbreak information, coloured by number of publications. Outbreaks reported in France and Netherlands reflected outbreaks in overseas regions. Countries with black diagonal stripes indicate locations where ZIKV transmission has been reported by the WHO. (B) Geolocated areas or regions with ZIKV seroprevalence studies (using IgG assay, HAI/HI, MIA, NS1 BOB ELISA, IFA, capture ELISA and neutralisation assays) conducted in the general population in the Americas (left), Africa (centre) and Asia (right). Each dot represents a location-specific estimate, while shaded areas indicate estimates at the administrative unit level (region, province, district, or entire country). The map was created using administrative boundary data from the open-access resource GADM database of Global Administrative Areas (GADM database).

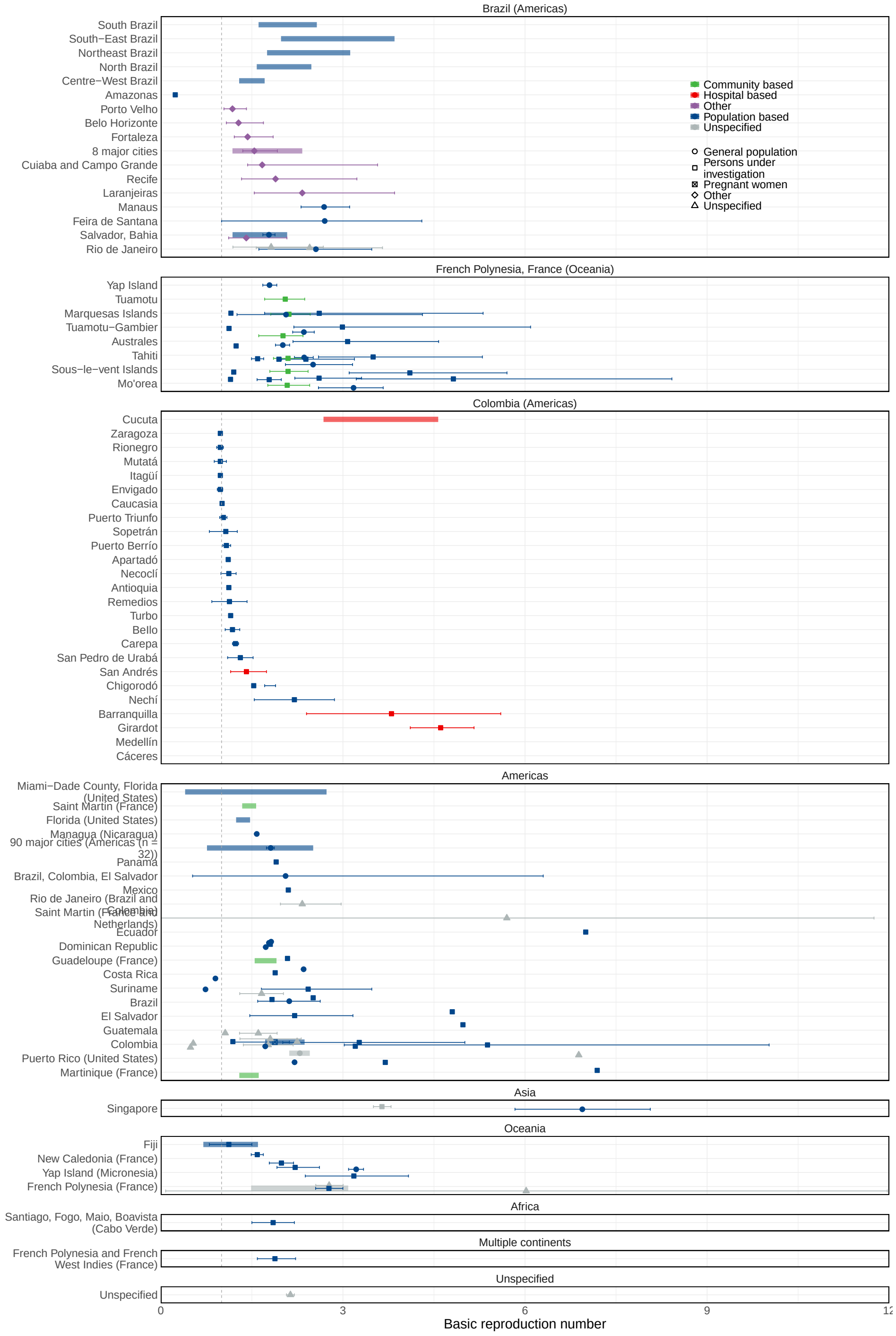

Figure B.26: Estimates by location, type of sample, and population group of the basic reproduction number of ZIKV. Points are central estimates (defined as either mean, median or unspecified central as estimated in the extracted paper), error bars are 95% confidence or credible intervals, and shaded bars are ranges of central estimates over disaggregated groups. When there were multiple estimates for the same location, points, error bars, and/or ranges were jittered. Note that the point estimates for Medellín (median 22.2) and Cáceres (median 56.38) are not shown because they are outside of the plot bounds. The grey vertical dotted line marks 1. The sample sizes for each study shown in the figure are reported in Supplementary Table B.12.

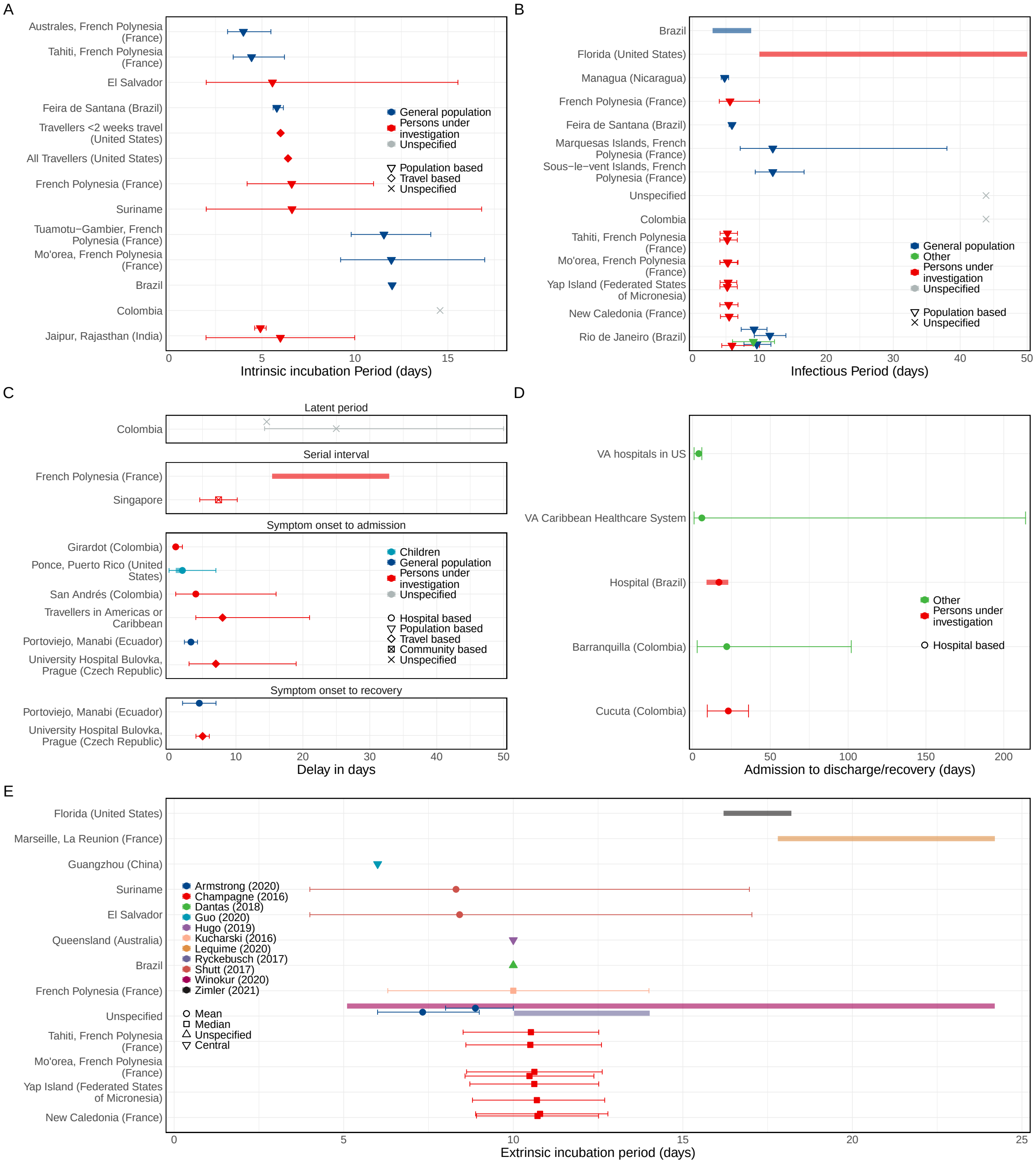

Figure B.27: Estimates by location and type of sample of the infectious period of ZIKV (A), incubation period (B), admission to discharge/recovery (C), and serial interval, symptom onset to admission, and symptom onset to recovery (D), and estimates by location and study of the extrinsic incubation period in the mosquito (E). Points are the central estimates reported in the studies (defined as either mean, median or unspecified central as estimated in the extracted paper), error bars are 95% confidence or credible intervals, and shaded bars are ranges of central estimates over disaggregated groups. The sample sizes for each study shown in the figure are reported in Supplementary Table B.9.

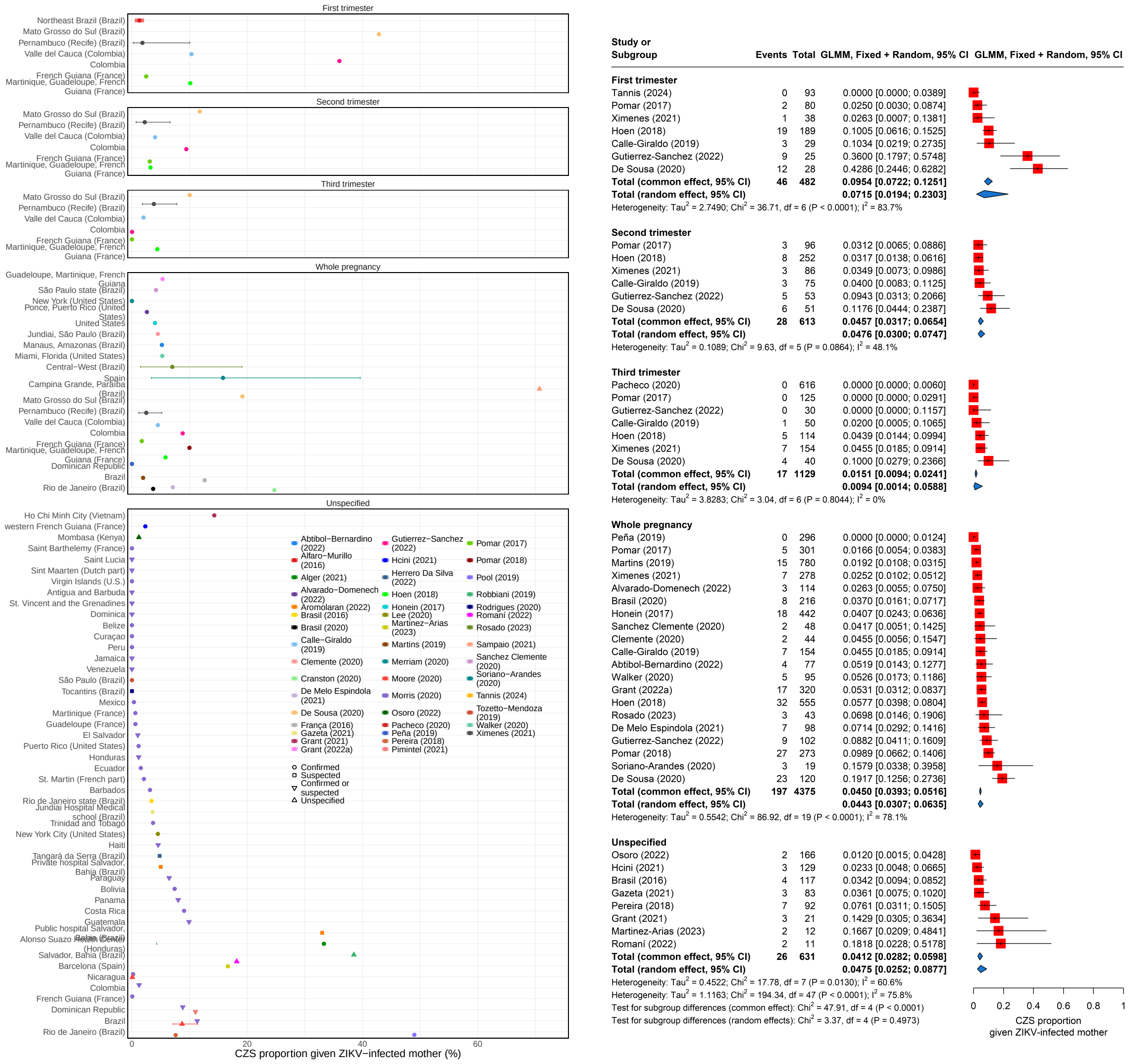

Figure B.28: (A) Estimates of reported CZS proportion. The top panel shows estimates from Brazil, while the bottom panel shows estimates from the rest of the world. Points are central estimates (defined as either mean, median or unspecified central as estimated in the extracted paper), solid lines are confidence or credible intervals, and shaded segments are ranges of central estimates across multiple groups. (B) Meta-analysis of CZS risk stratified by population sample type. We used generalised linear mixed-effect model (GLMM) estimates of CZS proportion. Red squares represent the observed study effect sizes and the solid black horizontal lines are confidence intervals. Blue diamonds represent pooled common and random effect estimates by trimester and overall. Common effect estimates assume that all aggregated data come from a single data-generating process with one common CZS proportion, and overall random effect estimates allow the CZS proportion to vary by study and give different weights to each study in the overall estimate. The "Events" column indicates the reported number of CZS cases. The vertical dashed line is the overall pooled estimate. The sample sizes for each study shown in the figure are reported in Supplementary Table B.16. Statistical analyses were performed using the Chi^2 test for heterogeneity, with two-sided significance. Between-study variance was estimated using Tau^2, and I^2 indicates the proportion of total variation due to heterogeneity rather than chance. Figures report the p-value for each group, rounding to < 0.0001 when values fall below this threshold in the meta-analysis.

B.5 Extended meta-analyses on CZS proportion and pregnancy loss proportion

In the main text, we presented our meta-analysis of CZS proportion by trimester. We also show the meta-analysis of the pregnancy loss proportion. We performed subgroup analyses by country (for Brazil), continent, and trimester for CZS proportion and by country (for Brazil) and continent for pregnancy loss proportion to explore the heterogeneity in estimates.

The CZS proportion funnel plot (Figure B.29) and the pregnancy loss proportion funnel plot (Figure B.30) are relatively symmetric around the overall pooled random effects estimates.

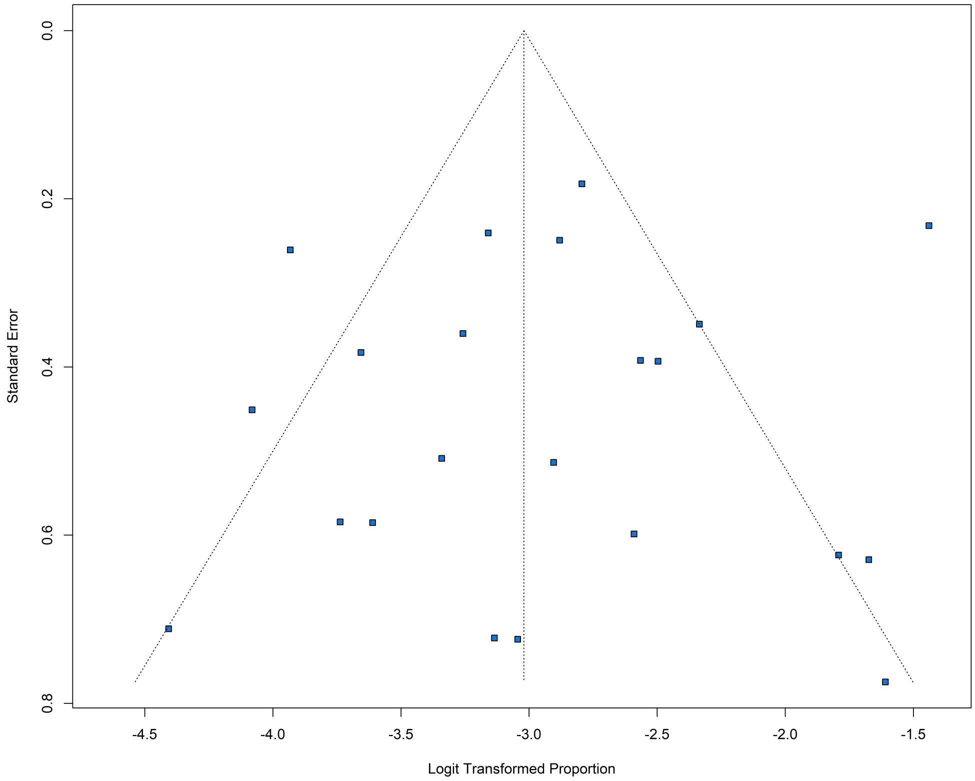

Figure B.29: Funnel plot for the CZS proportion meta-analysis. Blue squares represent individual study estimates.

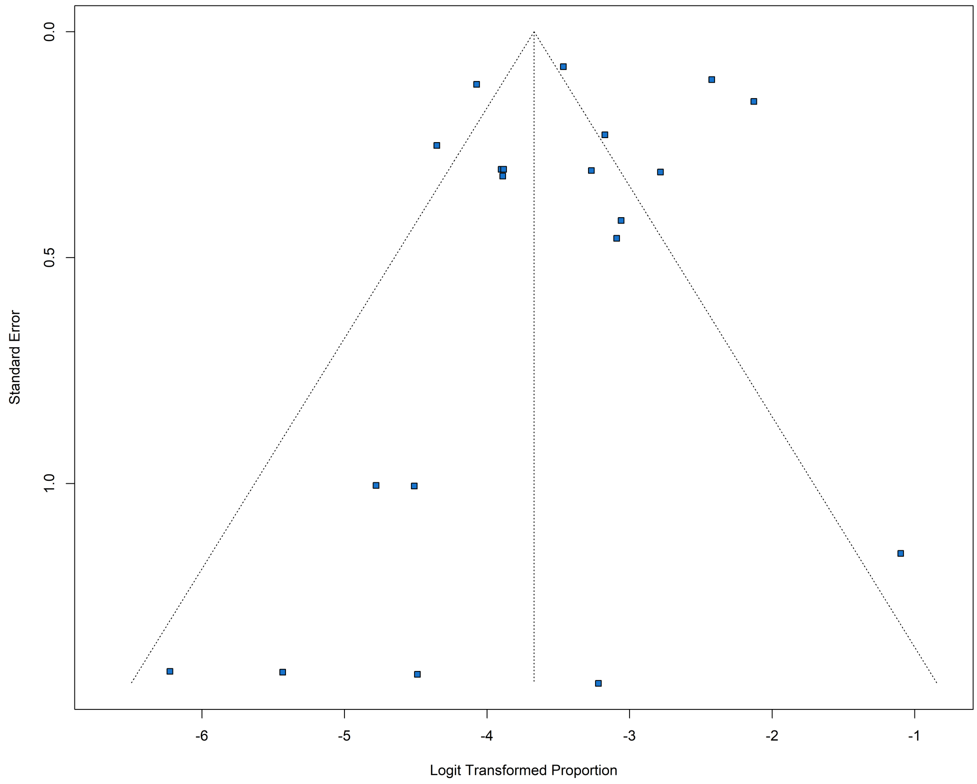

Figure B.30: Funnel plot for the pregnancy loss proportion meta-analysis. Blue squares represent individual study estimates.

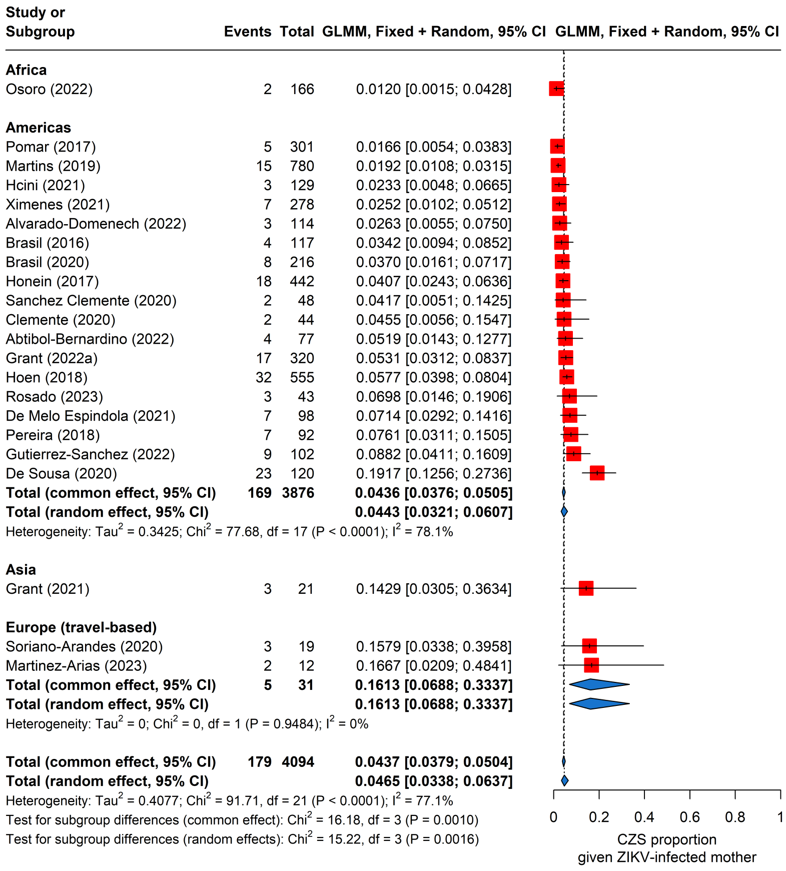

Figure B.31: CZS proportion meta-analysis stratified by continent. We used generalised linear mixed-effect model (GLMM) estimates of CZS proportion. Red squares represent the observed study effect sizes and the solid black horizontal lines are confidence intervals. Blue diamonds represent pooled common and random effect estimates by continent and overall. Common effect estimates assume that all aggregated data come from a single data-generating process with one common CZS proportion, and overall random effect estimates allow the CZS proportion to vary by study and give different weights to each study in the overall estimate. The "Events" column indicates the reported number of CZS cases. The vertical dashed line is the overall pooled estimate. The sample sizes for each study shown in the figure are reported in Supplementary Table B.16. Statistical analyses were performed using the Chi^2 test for heterogeneity, with two-sided significance. Between-study variance was estimated using Tau^2, and I^2 indicates the proportion of total variation due to heterogeneity rather than chance. Figures report the p-value for each group, rounding to < 0.0001 when values fall below this threshold in the meta-analysis.

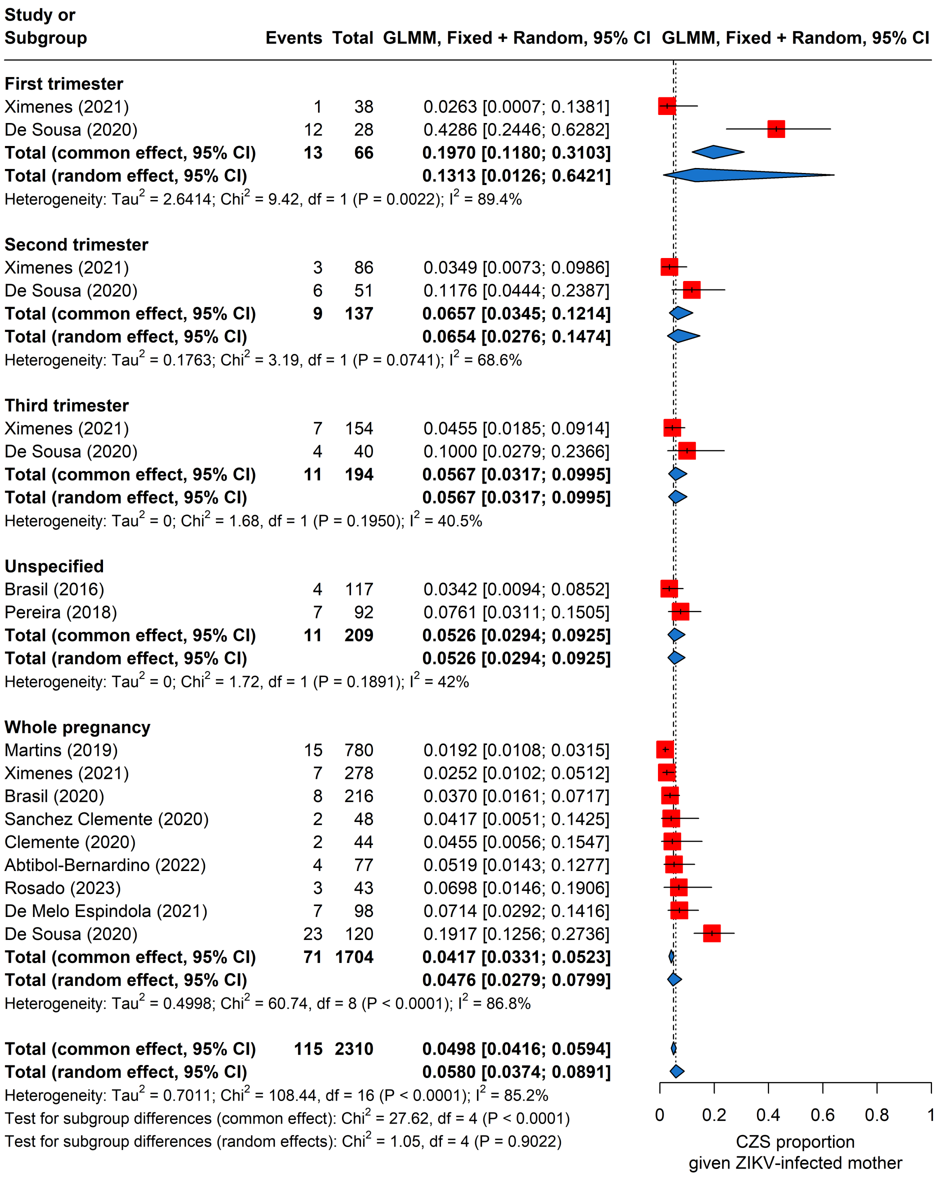

Figure B.32: CZS proportion meta-analysis of estimates from Brazil, stratified by trimester of ZIKV exposure. We used generalised linear mixed-effect model (GLMM) estimates of CZS proportion. Red squares represent the observed study effect sizes and the solid black horizontal lines are confidence intervals. Blue diamonds represent pooled common and random effect estimates by continent and overall. Common effect estimates assume that all aggregated data come from a single data-generating process with one common CZS proportion, and overall random effect estimates allow the CZS proportion to vary by study and give different weights to each study in the overall estimate. The "Events" column indicates the reported number of CZS cases. The vertical dashed line is the overall pooled estimate. The sample sizes for each study shown in the figure are reported in Supplementary Table B.16. Statistical analyses were performed using the Chi^2 test for heterogeneity, with two-sided significance. Between-study variance was estimated using Tau^2, and I^2 indicates the proportion of total variation due to heterogeneity rather than chance. Figures report the p-value for each group, rounding to < 0.0001 when values fall below this threshold in the meta-analysis.

Study	Events	Total	GLMM, Fixed + Random, 95% CI
Hoen (2018)	0	252	0.0000 [0.0000; 0.0145]
Hoen (2018)	0	114	0.0000 [0.0000; 0.0318]
De Sousa (2020)	1	120	0.0083 [0.0002; 0.0456]
Pereira (2018)	1	92	0.0109 [0.0003; 0.0591]
Clemente (2020)	0	44	0.0000 [0.0000; 0.0804]
Cañas (2020)	16	1259	0.0127 [0.0073; 0.0206]
Ospina (2020)	75	4481	0.0167 [0.0132; 0.0209]
Hoen (2018)	11	555	0.0198 [0.0099; 0.0352]
Cañas (2020)	10	499	0.0200 [0.0097; 0.0365]
Grant (2022a)	11	546	0.0201 [0.0101; 0.0358]
Ospina (2020)	172	5673	0.0303 [0.0260; 0.0351]
Pomar (2020)	11	300	0.0367 [0.0184; 0.0647]
Wongsawat (2024)	0	12	0.0000 [0.0000; 0.2646]
Pomar (2017)	20	498	0.0402 [0.0247; 0.0613]
Tannis (2024)	5	115	0.0435 [0.0143; 0.0985]
Brasil (2016)	6	134	0.0448 [0.0166; 0.0949]
Hoen (2018)	11	189	0.0582 [0.0294; 0.1017]
Ospina (2020)	97	1192	0.0814 [0.0665; 0.0984]
Honein (2017)	47	442	0.1063 [0.0792; 0.1389]
Marban-Castro (2021)	1	4	0.2500 [0.0063; 0.8059]
**Total (common effect, 95% CI)**	**495**	**16521**	**0.0300 [0.0275; 0.0327]**
**Total (random effect, 95% CI)**			**0.0248 [0.0162; 0.0378]**

Heterogeneity: $Tau^2 = 0.6492$; $Chi^2 = 213.43$, df = 19 ($P < 0.0001$); $I^2 = 91.1\%$

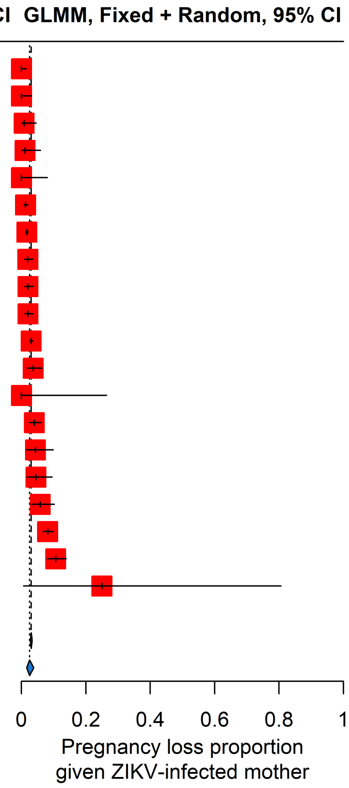

Figure B.33: Pregnancy loss proportion meta-analysis, non-stratified. We used generalised linear mixed-effect model (GLMM) estimates of pregnancy loss proportion. Red squares represent the observed study effect sizes and the solid black horizontal lines are confidence intervals. Blue diamonds represent overall common effect estimates, which assume that all aggregated data come from a single data-generating process with one common pregnancy loss proportion, and overall random effect estimates, which allow the pregnancy loss proportion to vary by study and give different weights to each study in the overall estimate. The "Events" column indicates the reported number of pregnancy losses. The vertical dashed line is the overall pooled estimate. The sample sizes for each study shown in the figure are reported in Supplementary Table B.16. Statistical analyses were performed using the Chi^2 test for heterogeneity, with two-sided significance. Between-study variance was estimated using Tau^2, and I^2 indicates the proportion of total variation due to heterogeneity rather than chance. Figures report the p-value for each group, rounding to < 0.0001 when values fall below this threshold in the meta-analysis.

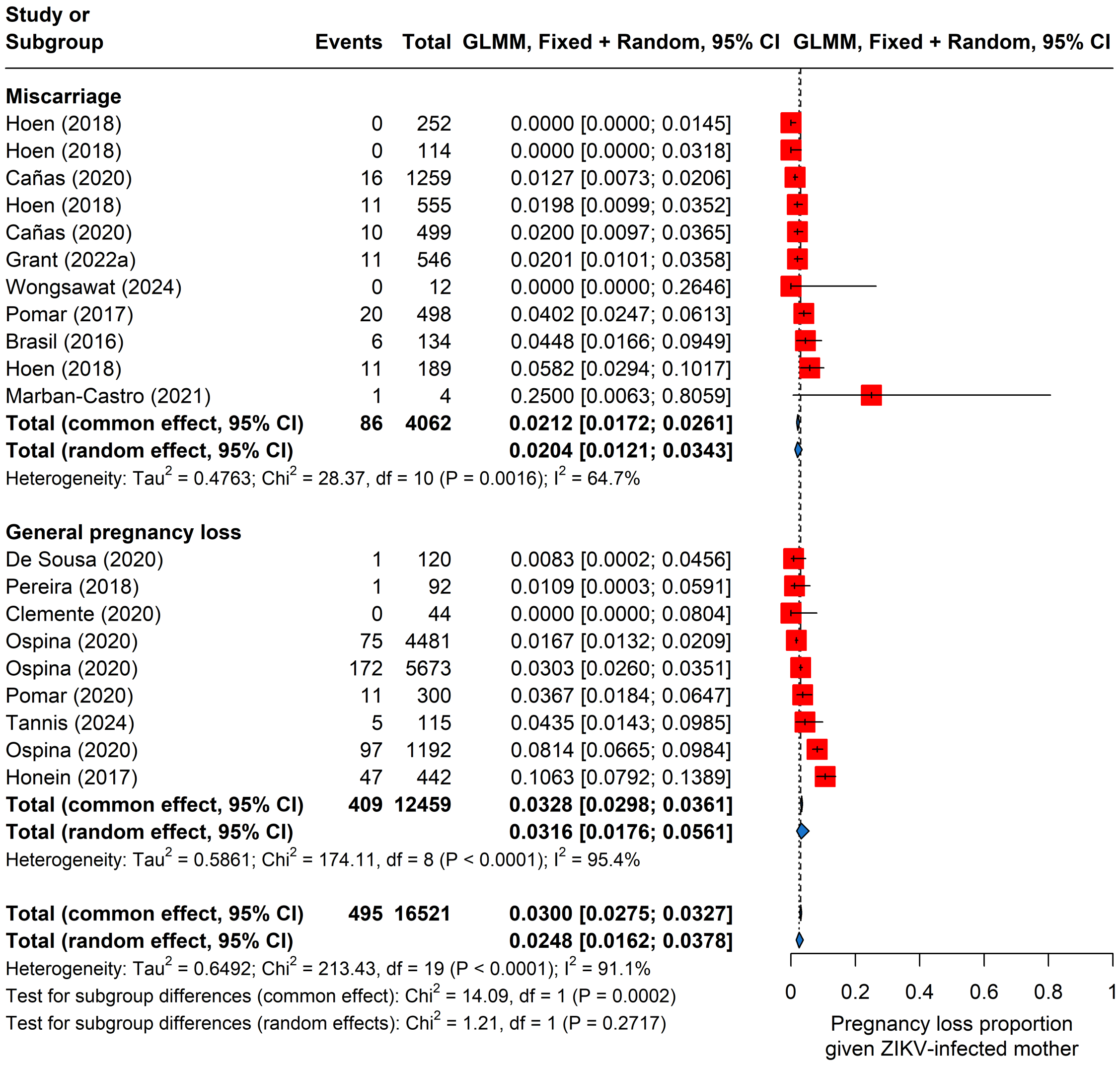

Figure B.34: Pregnancy loss proportion meta-analysis, stratified by pregnancy outcome. We used generalised linear mixed-effect model (GLMM) estimates of pregnancy loss proportion. Red squares represent the observed study effect sizes and the solid black horizontal lines are confidence intervals. Blue diamonds represent pooled common and random effect estimates by type of pregnancy loss and overall. Common effect estimates assume that all aggregated data come from a single data-generating process with one common pregnancy loss proportion, and overall random effect estimates allow the pregnancy loss proportion to vary by study and give different weights to each study in the overall estimate. The "Events" column indicates the reported number of pregnancy losses. The vertical dashed line is the overall pooled estimate. The sample sizes for each study shown in the figure are reported in Supplementary Table B.16. Statistical analyses were performed using the Chi^2 test for heterogeneity, with two-sided significance. Between-study variance was estimated using Tau^2, and I^2 indicates the proportion of total variation due to heterogeneity rather than chance. Figures report the p-value for each group, rounding to < 0.0001 when values fall below this threshold in the meta-analysis.

B.6 Meta-analyses on proportion of symptomatic individuals

In the main text, we presented the results of our meta-analysis of the proportion of symptomatic individuals. This analysis includes 7 estimates (from 2 papers) from overseas territories of France. In more details, 6 estimates come from a study in the general population and children of the French Polynesia (Society Islands, Tuamotu Islands, Marquesas islands and Austral-Gamber islands) and one from a study in pregnant women in French Guiana. The meta-analysis is shown in Figure B.35 and the funnel plot (Figure B.36) is relatively symmetric around the overall pooled random effects estimates.

Study	Events	Total	GLMM, Fixed + Random, 95% CI
Flamand (2017)	133	573	0.2321 [0.1981; 0.2689]
Aubry (2017)	8	18	0.4444 [0.2153; 0.6924]
Aubry (2017)	73	154	0.4740 [0.3931; 0.5560]
Aubry (2017)	12	22	0.5455 [0.3221; 0.7561]
Aubry (2017)	16	28	0.5714 [0.3718; 0.7554]
Aubry (2017)	19	29	0.6552 [0.4567; 0.8206]
Aubry (2017)	221	312	0.7083 [0.6545; 0.7582]
**Total (common effect, 95% CI)**	**482**	**1136**	**0.4243 [0.3958; 0.4533]**
**Total (random effect, 95% CI)**			**0.5120 [0.3800; 0.6423]**

Heterogeneity: $Tau^2 = 0.4284$; $Chi^2 = 183.74$, df = 6 ($P < 0.0001$); $I^2 = 96.7\%$

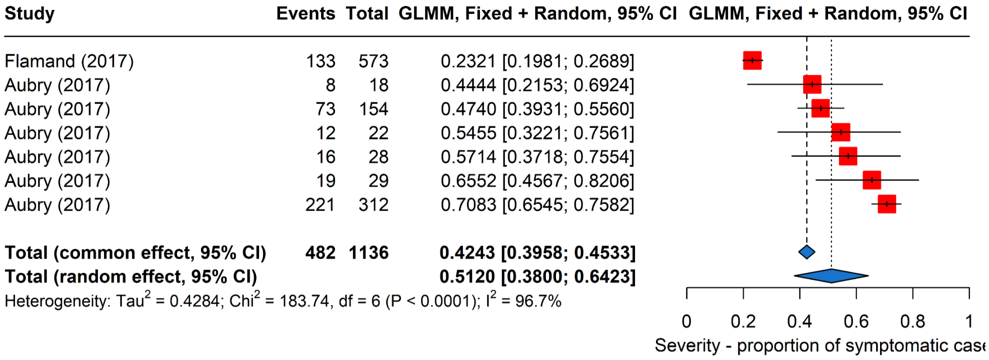

Figure B.35: Meta-analysis of the proportion of symptomatic individuals. We used generalised linear mixed-effect model (GLMM) estimates of proportion of symptomatic individuals. Red squares represent the observed study effect sizes and the solid black horizontal lines are confidence intervals. Blue diamonds represent pooled overall common effect estimates, which assume that all aggregated data come from a single data-generating process with one common proportion of symptomatic individuals, and overall random effect estimates, which allow the proportion of symptomatic individuals to vary by study and give different weights to each study in the overall estimate. The "Events" column indicates the reported number of symptomatic individuals. The vertical dashed line is the overall pooled estimate. The sample sizes for each study shown in the figure are reported in Supplementary Table B.16. Statistical analyses were performed using the Chi^2 test for heterogeneity, with two-sided significance. Between-study variance was estimated using Tau^2, and I^2 indicates the proportion of total variation due to heterogeneity rather than chance. Figures report the p-value for each group, rounding to < 0.0001 when values fall below this threshold in the meta-analysis.

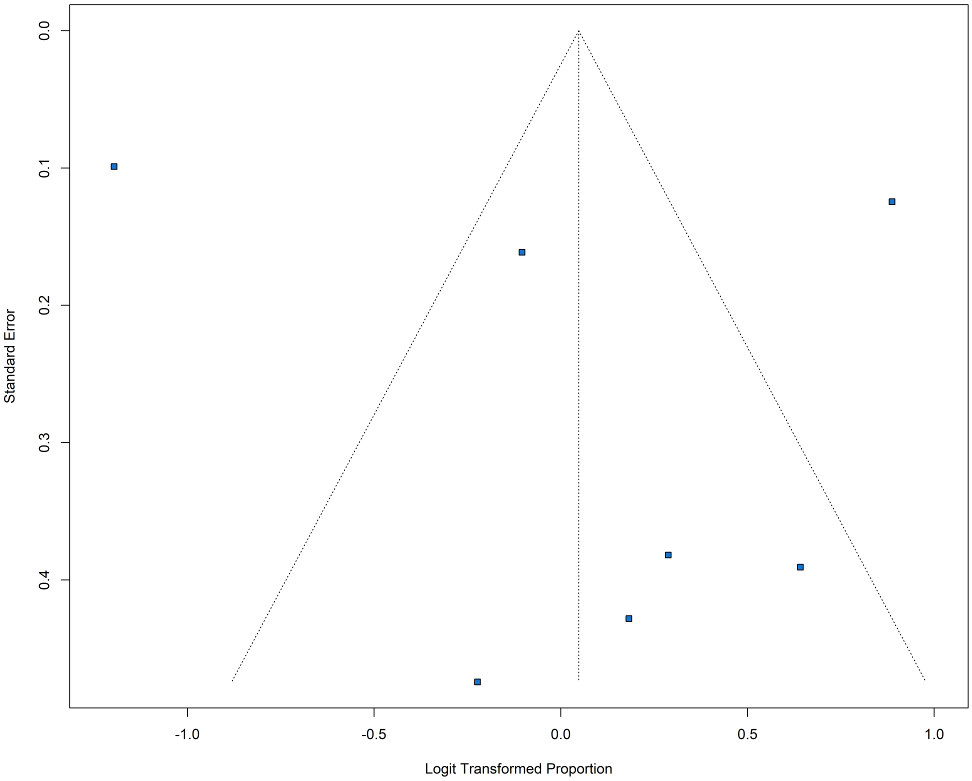

Figure B.36: Funnel plot for the or proportion of symptomatic individuals meta-analysis. Blue squares represent individual study estimates.

B.7 Tables of extracted parameters, models, and outbreaks

B.7.1 Models

Information on transmission models was extracted from 154 studies (Supplementary Tables B4 and B8, Figure B4), the majority of which were compartmental (n=120). Other published models included branching processes (n=6) and individual-based models (n=6). Most models accounted for vector-borne transmission (n=127), and some also included human-to-human sexual transmission (n=45). The most common compartmental type used was the SEIR-SEI model (n=47), with Susceptible–Exposed–Infectious–Recovered (SEIR) compartments for humans, and Susceptible–Exposed-Infectious (SEI) compartments for vectors. Notably, no modelling studies were published before the outbreak in Brazil in 2015, but there was a large surge in modelling publications in the following years, with 11 modelling studies published in 2024. Among the 154 studies reporting ZIKV transmission models, only 26 studies (17%) published the associated code.

Transmission Route(s)	Model Assumptions	Model Compartments	Fitted	Interventions	Source
**Branching Process**					
Unspecified	Unspecified		Fitted		Chowell (2016)
Unspecified	Unspecified		Fitted		Zhao (2019)
Unspecified			Fitted		Chowell (2021)
Mosquito-Human	Unspecified	SIR-SI	Theoretical	Behaviour Changes, Other	Soewono (2021)
Mosquito-Human	Heterogenity in transmission over time, Homogeneous mixing, Other	SIR	Fitted		Riou (2017)
Unspecified	Unspecified	SIR	Fitted		Dinh (2016)
Mosquito-Human	Homogeneous mixing	Other	Fitted	Treatment, Vaccination	Carmona (2020)
**Branching process;Compartmental - Stochastic**					
Mosquito-Human	Other	SEIR	Theoretical		Castro (2017)
**Compartmental - Deterministic**					
Mosquito-Human	Heterogenity in transmission between human and vector, Heterogenity in transmission over time	SEIR-SEI	Theoretical	Behaviour Changes, Pesticides/larvicides, Treatment, Vaccination, Wolbachia replacement, Wolbachia suppression	Ngonghala (2021)
Mosquito-Human	Heterogenity in transmission between human and vector, Heterogenity in transmission over time, Latent and incubation periods are same	SEIR-SEI	Theoretical		Huber (2018)
Mosquito-Human	Heterogenity in transmission over time, Homogeneous mixing	SEIR-SEI	Theoretical		Roy (2020)
Mosquito-Human	Homogeneous mixing	SEIR-SEI	Theoretical		Suantai (2023)
Mosquito-Human	Homogeneous mixing	SEIR-SEI	Theoretical		Kumar (2022)
Mosquito-Human	Homogeneous mixing	SEIR-SEI	Fitted		Prasad (2023)
Mosquito-Human	Homogeneous mixing	SEIR-SEI	Fitted		Kumar (2017)
Mosquito-Human	Homogeneous mixing	SEIR-SEI	Fitted		Dantas (2018)
Mosquito-Human	Homogeneous mixing	SEIR-SEI	Theoretical	Wolbachia replacement	Xue (2018)
Mosquito-Human	Homogeneous mixing, Latent and incubation periods are same	SEIR-SEI	Fitted	Other, Pesticides/larvicides, Treatment	Alzahrani (2021)
Mosquito-Human	Homogeneous mixing, Other	SEIR-SEI	Fitted		Funk (2016)
Mosquito-Human	Homogeneous mixing, Other	SEIR-SEI	Fitted	Insecticide-treated nets, Other, Quarantine, Mosquito Control	Yue (2023)
Mosquito-Human	Homogeneous mixing, Other	SEIR-SEI	Fitted		Rahman (2019)
Mosquito-Human	Other	SEIR-SEI	Fitted		Chen (2018)
Mosquito-Human	Other	SEIR-SEI	Theoretical		Yue (2022)
Mosquito-Human	Other	SEIR-SEI	Theoretical	Mosquito Control	Yue (2020)
Mosquito-Human	Unspecified	SEIR-SEI	Theoretical		Imran (2021)
Mosquito-Human	Unspecified	SEIR-SEI	Theoretical		Gao (2019)
Mosquito-Human		SEIR-SEI	Theoretical		Moreno (2017)
Mosquito-Human		SEIR-SEI	Theoretical	Other, Mosquito Control	Agusto (2017a)
Mosquito-Human		SEIR-SEI	Theoretical	Insecticide-treated nets, Treatment, Mosquito Control	Bonyah (2017)
Mosquito-Human		SEIR-SEI	Theoretical	Mosquito Control	Stone (2019)
Mosquito-Human		SEIR-SEI	Fitted		Caldwell (2021)
Mosquito-Human		SEIR-SEI	Theoretical		Nisar (2024)
Sexual, Mosquito-Human	Heterogenity in transmission between human and vector, Heterogenity in transmission between human groups	SEIR-SEI	Fitted	Behaviour Changes, Insecticide-treated nets, Mosquito Control	Kumar Biswas (2020)
Sexual, Mosquito-Human	Heterogenity in transmission between human and vector, Heterogenity in transmission between human groups	SEIR-SEI	Theoretical		Thaiprayoon (2022)

continued on next page

continued from previous page

Model Compartments	Transmission Route(s)	Model Assumptions	Fitted	Interventions	Source
Sexual, Mosquito-Human	Heterogenity in transmission between human and vector, Homogeneous mixing, Latent and incubation periods are same	SEIR-SEI	Theoretical		Towers (2016)
Sexual, Mosquito-Human	Homogeneous mixing	SEIR-SEI	Theoretical		Yamazaki (2019)
Sexual, Mosquito-Human	Homogeneous mixing	SEIR-SEI	Fitted	Behaviour Changes, Pesticides/larvicides, Vaccination	Valega-Mackenzie (2023)
Sexual, Mosquito-Human	Latent and incubation periods are same	SEIR-SEI	Theoretical	Indoor residual spraying, Insecticide-treated nets	Padmanabhan (2017b)
Sexual, Mosquito-Human	Other	SEIR-SEI	Theoretical	Behaviour Changes, Genetically modified mosquitoes, Indoor residual spraying, Mosquito Control, Wolbachia replacement	Bi (2020)
Sexual, Mosquito-Human	Unspecified	SEIR-SEI	Theoretical	Indoor residual spraying, Insecticide-treated nets, Treatment	Ali (2022b)
Sexual, Mosquito-Human	Unspecified	SEIR-SEI	Theoretical		Khan (2021)
Sexual, Mosquito-Human	Unspecified	SEIR-SEI	Fitted	Other, Vaccination, Mosquito Control	Burgess (2021)
Sexual, Mosquito-Human	Unspecified	SEIR-SEI	Fitted		Sow (2022)
Sexual, Mosquito-Human	Unspecified	SEIR-SEI	Theoretical	Vaccination	Hussain (2021)
Sexual, Mosquito-Human		SEIR-SEI	Fitted	Mosquito Control	Wang (2017)
Sexual, Mosquito-Human		SEIR-SEI	Fitted		Ibrahim (2021)
Sexual, Mosquito-Human		SEIR-SEI	Theoretical		Harvim (2019)
Human-Human, Sexual, Mosquito-Human	Other	SEIR-SEI	Theoretical		Hasan (2019)
Human-Human, Mosquito-Human	Heterogenity in transmission between human groups	SEIR-SEI	Theoretical	Behaviour Changes, Indoor residual spraying, Other, Vaccination	Okyere (2020)
Human-Human, Mosquito-Human	Homogeneous mixing	SEIR-SEI	Fitted		Sadeghieh (2021)
Human-Human, Mosquito-Human	Homogeneous mixing, Latent and incubation periods are same, Other	SEIR-SEI	Fitted		Kucharski (2016)
Human-Human, Mosquito-Human	Other	SEIR-SEI	Theoretical	Hospitals	Imran (2018)
Human-Human, Mosquito-Human	Unspecified	SEIR-SEI	Theoretical	Treatment, Vaccination	Jan (2023)
Human-Human, Mosquito-Human	Unspecified	SEIR-SEI	Theoretical		Bekiryazici (2022)
Human-Human, Mosquito-Human	Unspecified	SEIR-SEI	Fitted		Ali (2022a)
Human-Human, Mosquito-Human	Unspecified	SEIR-SEI	Theoretical	Behaviour Changes, Insecticide-treated nets, Other, Mosquito Control	Goswami (2021)
Human-Human, Mosquito-Human	Unspecified	SEIR-SEI	Theoretical		Veeresha (2022)
Mosquito-Human	Other	SEI-SI	Theoretical		Martin (2021)
Sexual, Mosquito-Human	Other	SEIR, SIR	Theoretical		Terefe (2018)
Human-Human, Mosquito-Human	Unspecified	SI-SI	Theoretical		Dharmalingam (2024)
Mosquito-Human	Homogeneous mixing	SIR-SEI	Theoretical	Vaccination	Massad (2019)
Human-Human, Mosquito-Human	Unspecified	SIR-SEI	Theoretical	Vaccination	Sharma (2021)
Mosquito-Human	Heterogenity in transmission over time, Other	SIR-SI	Theoretical		Al Najim (2024)
Mosquito-Human	Other	SIR-SI	Theoretical		Rakkiyappan (2019)
Mosquito-Human	Unspecified	SIR-SI	Theoretical	Behaviour Changes, Other	Soewono (2021)
Mosquito-Human	Unspecified	SIR-SI	Theoretical		Alfwzan (2023)
Mosquito-Human	Unspecified	SIR-SI	Theoretical		Alshehri (2022)
Mosquito-Human	Unspecified	SIR-SI	Theoretical		Alkahtani (2017)
Mosquito-Human		SIR-SI	Fitted		Mishra (2021)
Mosquito-Human		SIR-SI	Theoretical		Jiao (2021)
Sexual, Mosquito-Human	Heterogenity in transmission between human and vector, Heterogenity in transmission between human groups, Other	SIR-SI	Fitted	Treatment	Zhao (2020)
Sexual, Mosquito-Human	Other	SIR-SI	Fitted	Vaccination	Miyaoka (2019)
Sexual, Mosquito-Human	Unspecified	SIR-SI	Theoretical	Other	Angina (2022)
Human-Human, Mosquito-Human	Homogeneous mixing, Unspecified	SIR-SI	Theoretical		Binder (2019)
Human-Human, Mosquito-Human	Unspecified	SIR-SI	Theoretical	Behaviour Changes, Insecticide-treated nets, Other, Pesticides/larvicides	Ali (2021)
Mosquito-Human, Unspecified	Other	SIR-SI	Theoretical		Wang (2021)
Mosquito-Human	Homogeneous mixing	SIRS-SI	Theoretical	Other, Pesticides/larvicides	González-Parra (2020)
Sexual, Mosquito-Human	Homogeneous mixing	SIRS-SI	Fitted	Behaviour Changes, Insecticide-treated nets, Mosquito Control	Ukanwoke (2022)
Human-Human, Mosquito-Human	Other	SLIR-SLI	Theoretical		Van Wyk (2023)
Mosquito-Human	Homogeneous mixing	SIR	Fitted		O'driscoll (2021)
Mosquito-Human	Homogeneous mixing, Other	SIR	Fitted		Ospina (2017)
Human-Human	Homogeneous mixing	SIR	Fitted		Bastos (2018)
Human-Human	Homogeneous mixing	SIR	Fitted		Angulo (2018)
Human-Human		SIR	Fitted		Harris (2019)
Sexual, Mosquito-Human	Homogeneous mixing	SIR	Theoretical		Baca-Carrasco (2016)
Sexual, Mosquito-Human	Homogeneous mixing, Other	SIR	Theoretical		Baca-Carrasco (2016)
Human-Human, Mosquito-Human	Homogeneous mixing	SIR	Theoretical	Behaviour Changes, Pesticides/larvicides, Mosquito Control	Anggriani (2023)

continued on next page

continued from previous page

Model Compartments	Transmission Route(s)	Model Assumptions	Fitted	Interventions	Source
Mosquito-Human	Latent and incubation periods are same	SEIR	Theoretical		Zhao (2023)
Mosquito-Human		SEIR	Fitted		Netto (2017)
Sexual, Mosquito-Human	Heterogenity in transmission between human groups	SEIR	Fitted		De Barros (2019)
Human-Human, Mosquito-Human	Homogeneous mixing, Other	SEIR	Theoretical		Baca-Carrasco (2016)
Mosquito-Human	Other	Other SEIR-SEI	Theoretical		Ren (2024)
Mosquito-Human		Other SEIR-SEI	Theoretical		Wang (2023)
Sexual, Mosquito-Human	Homogeneous mixing	Other SEIR-SEI	Fitted	Mosquito Control	Gao (2016)
Sexual, Mosquito-Human	Other	Other SEIR-SEI	Theoretical	Behaviour Changes, Insecticide-treated nets, Pesticides/larvicides, Mosquito Control	Chaikham (2017)
Sexual, Mosquito-Human	Unspecified	Other SEIR-SEI	Fitted		Tonsing (2018)
Human-Human, Sexual, Mosquito-Human	Age	Other SEIR-SEI	Theoretical	Behaviour Changes, Insecticide-treated nets, Pesticides/larvicides, Treatment, Mosquito Control	Chaikham (2018)
Sexual, Mosquito-Human	Unspecified	SAIR-SEI	Theoretical	Behaviour Changes, Indoor residual spraying, Insecticide-treated nets, Treatment	Momoh (2018)
Mosquito-Human	Cross-immunity, Homogeneous mixing	Other	Theoretical	Behaviour Changes, Insecticide-treated nets, Other, Treatment, Mosquito Control	Bonyah (2019)
Mosquito-Human	Cross-immunity, Other	Other	Fitted		Henderson (2021)
Mosquito-Human	Heterogenity in transmission between human and vector, Heterogenity in transmission between human groups	Other	Theoretical		Muñoz (2017)
Mosquito-Human	Heterogenity in transmission between human groups, Other	Other	Fitted		Liang (2019)
Mosquito-Human	Homogeneous mixing	Other	Fitted		Aranda (2019)
Mosquito-Human	Homogeneous mixing	Other	Fitted	Other	Kumar (2023)
Mosquito-Human	Latent and incubation periods are same	Other	Theoretical		Zhang (2022)
Mosquito-Human	Other	Other	Theoretical		Fitzgibbon (2017)
Mosquito-Human	Other	Other	Theoretical	Behaviour Changes, Other, Pesticides/larvicides, Treatment, Mosquito Control	Djomegni (2021)
Mosquito-Human	Other	Other	Theoretical	Other, Vaccination	Kribs (2023)
Mosquito-Human	Other, Unspecified	Other	Theoretical		Caminade (2017)
Mosquito-Human	Unspecified	Other	Theoretical		Khan (2019b)
Mosquito-Human	Unspecified	Other	Theoretical	Behaviour Changes, Treatment, Mosquito Control	Manisha (2023)
Mosquito-Human		Other	Fitted		Villela (2017)
Mosquito-Human		Other	Fitted		O'Reilly (2018)
Mosquito-Human		Other	Theoretical		Han (2024)
Mosquito-Human		Other	Theoretical		Hajji (2024)
Mosquito-Human		Other	Theoretical		Wang (2024b)
Mosquito-Human		Other	Theoretical		Danbaba (2018)
Mosquito-Human		Other	Fitted		Omame (2023b)
Mosquito-Human		Other	Theoretical		Ndairou (2018)
Human-Human	Heterogenity in transmission between human groups	Other	Theoretical	Other, Quarantine	Scatà (2016)
Human-Human	Unspecified	Other	Fitted		Massad (2017)
Sexual, Mosquito-Human	Heterogenity in transmission between human and vector	Other	Theoretical	Genetically modified mosquitoes, Treatment, Mosquito Control	Atokolo (2022)
Sexual, Mosquito-Human	Heterogenity in transmission between human and vector, Other	Other	Theoretical		Begum (2021)
Sexual, Mosquito-Human	Heterogenity in transmission between human and vector, Other	Other	Theoretical		Farman (2020)
Sexual, Mosquito-Human	Heterogenity in transmission between human and vector, Other	Other	Theoretical		Rezapour (2020)
Sexual, Mosquito-Human	Heterogenity in transmission between human groups	Other	Theoretical		Villela (2017)
Sexual, Mosquito-Human	Heterogenity in transmission between human groups, Homogeneous mixing	Other	Theoretical		Cruz-Pacheco (2019)
Sexual, Mosquito-Human	Homogeneous mixing	Other	Theoretical	Behaviour Changes, Indoor residual spraying, Insecticide-treated nets, Other, Pesticides/larvicides, Mosquito Control	Wang (2019)
Sexual, Mosquito-Human	Homogeneous mixing	Other	Fitted		Dénes (2019)
Sexual, Mosquito-Human	Homogeneous mixing	Other	Fitted	Pesticides/larvicides, Mosquito Control, Wolbachia replacement	Wang (2024a)

continued on next page

continued from previous page

Model Compartments	Transmission Route(s)	Model Assumptions	Fitted	Interventions	Source
Sexual, Mosquito-Human	Homogeneous mixing, Unspecified	Other	Fitted		Li (2019)
Sexual, Mosquito-Human	Latent and incubation periods are same	Other	Fitted	Mechanical removal of breeding sites , Pesticides/larvicides	Agudelo (2022)
Sexual, Mosquito-Human	Other	Other	Fitted	Behaviour Changes	Luo (2021)
Sexual, Mosquito-Human	Unspecified	Other	Theoretical		Brauer (2016)
Sexual, Mosquito-Human	Unspecified	Other	Theoretical	Behaviour Changes, Other, Treatment	Wattanasirikosone (2021)
Sexual, Mosquito-Human	Unspecified	Other	Fitted	Behaviour Changes, Other, Mosquito Control	Zhu (2022)
Sexual, Mosquito-Human		Other	Theoretical		Agusto (2017b)
Human-Human, Sexual, Mosquito-Human	Heterogenity in transmission between human and vector, Homogeneous mixing, Other	Other	Fitted		Yuan (2021)
Human-Human, Sexual, Mosquito-Human		Other	Fitted	Behaviour Changes, Mosquito Control	Huo (2023)
Human-Human, Mosquito-Human	Heterogenity in transmission between human groups, Homogeneous mixing	Other	Fitted		Tuncer (2018)
Human-Human, Mosquito-Human	Homogeneous mixing, Other	Other	Fitted	Other	Sanchez (2019)
Mosquito-Human, Unspecified	Cross-immunity, Other	Other	Fitted	Vaccination	Omame (2023a)
Unspecified	Cross-immunity, Other	Other	Theoretical	Other	Deolia (2024)
Unspecified	Unspecified	Other	Fitted		Zafar (2024)
Mosquito-Human	Homogeneous mixing		Theoretical		Amaku (2018)
Mosquito-Human	Unspecified		Theoretical		Maamar (2024)
Sexual	Cross-immunity		Fitted		Tang (2019)
Sexual, Mosquito-Human	Heterogenity in transmission between human groups, Other		Theoretical	Other, Pesticides/larvicides	Olawoyin (2018)
Sexual, Mosquito-Human			Theoretical	Mosquito Control	Padmanabhan (2017a)
Human-Human, Mosquito-Human	Unspecified		Theoretical		Rezapour (2023)
**Compartmental - Stochastic**					
Mosquito-Human	Cross-immunity, Homogeneous mixing, Other	SEIR-SEI	Fitted		Shutt (2017)
Mosquito-Human	Heterogenity in transmission between human and vector, Homogeneous mixing	SEIR-SEI	Fitted		Lourenco (2017)
Mosquito-Human	Heterogenity in transmission between human groups, Other	SEIR-SEI	Fitted		Perrotta (2022)
Mosquito-Human	Homogeneous mixing	SEIR-SEI	Fitted		Morrison (2020)
Mosquito-Human	Homogeneous mixing	SEIR-SEI	Theoretical	Behaviour Changes, Vaccination, Mosquito Control	Mina (2020)
Mosquito-Human	Other	SEIR-SEI	Theoretical		Tramonte (2019)
Mosquito-Human	Unspecified	SEIR-SEI	Theoretical		Gokila (2021)
Sexual, Mosquito-Human	Heterogenity in transmission between human groups, Other	SEIR-SEI	Fitted	Vaccination	Durham (2018)
Sexual, Mosquito-Human	Other	SEIR-SEI	Theoretical		He (2020)
Sexual, Mosquito-Human	Unspecified	SEIR-SEI	Theoretical	Insecticide-treated nets, Other, Treatment	Shao (2023)
Human-Human, Mosquito-Human	Other	SEIR-SEI	Theoretical		Sun (2018)
Human-Human, Mosquito-Human	Unspecified	SEIR-SEI	Theoretical		Bekiryazici (2022)
Mosquito-Human	Homogeneous mixing	SI-SI	Theoretical		Maity (2024)
Human-Human, Mosquito-Human	Heterogenity in transmission between human groups	SI-SI	Theoretical		Bekiryazici (2023)
Mosquito-Human	Homogeneous mixing, Latent and incubation periods are same	SIR	Fitted		Perkins (2016)
Unspecified	Unspecified	SIR	Fitted		Quandelacy (2021)
Mosquito-Human	Heterogenity in transmission over time, Other	SEIR	Fitted		Nguyen-Van-Yen (2021)
Human-Human		SEIR	Theoretical		Wardle (2024)
Mosquito-Human	Cross-immunity, Homogeneous mixing	Other	Fitted		Hirata (2023)
Mosquito-Human	Homogeneous mixing	Other	Theoretical		Zhu (2024)
Sexual, Mosquito-Human	Other	Other	Theoretical	Wolbachia suppression	Xue (2021)
Human-Human, Mosquito-Human	Heterogenity in transmission between human and vector, Homogeneous mixing	Other	Theoretical	Treatment	Algehyne (2022)
Mosquito-Human	Cross-immunity, Heterogenity in transmission between human groups, Other		Fitted		Champagne (2016)
**Compartmental - Unspecified**					
Mosquito-Human	Heterogenity in transmission between human and vector, Heterogenity in transmission over time, Homogeneous mixing, Latent and incubation periods are same	SEIR-SEI	Fitted	Pesticides/larvicides, Mosquito Control	Suparit (2018)
Mosquito-Human		SEIR-SEI	Theoretical		Manore (2017)
Mosquito-Human		SEIR-SEI	Fitted	Vaccination	Bartsch (2019)

continued on next page

continued from previous page

Model Compartments	Transmission Route(s)	Model Assumptions	Fitted	Interventions	Source
Human-Human, Mosquito-Human	Unspecified	SEIR-SEI	Fitted		Ibrahim (2023)
Mosquito-Human	Heterogenity in transmission between human and vector, Other	SIR	Fitted		Siraj (2017)
Mosquito-Human	Heterogenity in transmission between human groups, Homogeneous mixing, Other	Other	Fitted	Mosquito Control	Sasmal (2018)
Mosquito-Human		Other	Fitted		Fuller (2017)
Sexual, Mosquito-Human	Heterogenity in transmission between human groups, Other	Other	Theoretical		Maxian (2017)
Sexual, Mosquito-Human	Homogeneous mixing	Other	Theoretical	Mosquito Control	Saad-Roy (2018)
Sexual, Mosquito-Human	Other	Other	Theoretical	Vaccination	Valega-Mackenzie (2018)
Sexual, Mosquito-Human	Other, Heterogenity in transmission between human groups	Other	Theoretical		Saad-Roy (2016)
Human-Human, Mosquito-Human		Other	Theoretical	Insecticide-treated nets, Treatment, Mosquito Control	Khan (2019a)
Mosquito-Human	Heterogenity in transmission between human and vector, Homogeneous mixing		Fitted	Behaviour Changes, Treatment, Mosquito Control	Biswas (2024)
Sexual, Mosquito-Human			Fitted		Srivastav (2019)
**Compartmental;Other - Deterministic**					
Mosquito-Human	Other	SIR	Fitted		Claypool (2022)
**Compartmental;Other - Stochastic**					
Mosquito-Human		SIR	Fitted		Riou (2018)
**Compartmental;Other - Unspecified**					
Mosquito-Human	Unspecified	SEIR-SEI	Theoretical		Sabir (2023)
Unspecified	Other	SIR	Fitted		Rotejanaprasert (2019)
**Individual based - Stochastic**					
Sexual, Mosquito-Human	Age	SEIR-SEI	Theoretical	Behaviour Changes, Indoor residual spraying, Pesticides/larvicides, Mosquito Control	Moghadas (2017)
Sexual, Mosquito-Human	Heterogenity in transmission between human groups	SEIR-SEI	Theoretical		Ferdousi (2019)
Unspecified	Age, Heterogenity in transmission between human groups	SIR	Fitted	Vaccination	Counotte (2019)
Mosquito-Human	Heterogenity in transmission between human groups, Other	SEIR	Theoretical	Behaviour Changes	Ajelli (2017)
**Individual based;Compartmental - Deterministic**					
Mosquito-Human		SEIR-SEI	Fitted	Other, Mosquito Control	Gwalani (2018)
**Individual based;Compartmental - Stochastic**					
Mosquito-Human		SEIR-SEI	Fitted		Zhang (2017)
Mosquito-Human	Cross-immunity	SEIR	Fitted		Pinotti (2024)
Mosquito-Human		Other	Fitted		Marini (2017)
**Other - Deterministic**					
Mosquito-Human	Homogeneous mixing		Fitted		Rocklov (2016)
Mosquito-Human	Unspecified		Fitted	Other	Guzzetta (2016)
Mosquito-Human			Theoretical		Chen (2024)
Human-Human			Fitted		Ogden (2017)
Human-Human			Fitted		Hsieh (2017)
Sexual, Mosquito-Human			Fitted		Rojas (2016)
Unspecified	Unspecified		Fitted		Picinini Freitas (2024)
Mosquito-Human	Homogeneous mixing	Other	Theoretical		Rocklov (2016)
Unspecified	Unspecified		Theoretical		Sabir (2024)
**Other - Stochastic**					
Mosquito-Human	Other		Fitted		Aguiar (2018)
Mosquito-Human			Fitted		Nisar (2024)
Sexual	Heterogenity in transmission between human groups		Theoretical		Allard (2017a)
Sexual	Heterogenity in transmission between human groups		Theoretical		Allard (2017b)
Unspecified	Heterogenity in transmission over time, Other		Fitted		Charniga (2021b)
Unspecified	Other		Fitted		Gardner (2018)
**Other - Unspecified**					
Mosquito-Human			Fitted		Solimini (2018)
Human-Human			Fitted		Cousien (2019)
Unspecified	Other		Fitted		Bogoch (2016)
Unspecified	Unspecified		Fitted		Sebrango-Rodriguez (2017)
Unspecified			Fitted		Watts (2017)
Unspecified			Fitted		Fajardo (2016)
Unspecified			Fitted		Li (2021)
Unspecified			Theoretical		Farman (2022)
Unspecified			Fitted		Carreto (2022)
Unspecified			Fitted		Weinstein (2020)
Unspecified			Fitted		Zhang (2021)
Sexual, Mosquito-Human	Heterogenity in transmission between human and vector	Other	Theoretical		Wang (2022a)

continued on next page

continued from previous page

Model Compartments	Transmission Route(s)	Model Assumptions	Fitted	Interventions	Source
Unspecified		Other	Theoretical		Wang (2022b)
**Unspecified**					
Unspecified			Fitted		Omame (2023b)
**Unspecified - Stochastic**					
Unspecified	Unspecified	Other	Theoretical	Mechanical removal of breeding sites , Other	Madewell (2019)

Table B.7: Overview of extracted transmission models.

B.7.2 Outbreaks

Outbreak location	Outbreak dates	Suspected cases	Confirmed cases	Mode of case detection	Female cases	Male cases	References
Entire Country	26 Dec 2015 - Dec 2016	454	147	Molecular	115	32	Ryan (2018)
Entire Country	09 Aug 2015 - 02 Apr 2016		65726	Confirmed + Suspected	44133	21592	Pacheco (2020)
**Angola**							
	Dec 2016	54	4	Molecular	3	1	Hill (2019)
**Brazil**							
Manaus	Nov 2015 - Sep 2018		6987	Unspecified			Giovanetti (2020)
Salvador, Bahia	May 2015 - Aug 2017	434	78	Unspecified			Bandeira (2020)
Tocantins	Jul 2015 - Dec 2016	10775	1825	Molecular	1553	682	Rodrigues (2020)
Rio De Janeiro	Jan 2015 - 15 Apr 2016	25213		Symptoms			Villela (2017)
Rio De Janeiro	Feb 2015 - May 2016		1717	Molecular			Fuller (2017)
Minas Gerais State	Dec 2015 - Jul 2017		1723	Molecular	1578		Iani (2021)
Rio De Janeiro	2015 - 2016	39331		Unspecified			Raymundo (2021)
**Cabo Verde**							
Santiago, Fogo, Maio, Boavista	05 Oct 2015 - 29 May 2016	7580		Symptoms			Lourenco (2018)
Santiago	05 Oct 2015 - 29 May 2016	4937		Symptoms			Lourenco (2018)
Fogo	05 Oct 2015 - 29 May 2016	1458		Symptoms			Lourenco (2018)
**Chile**							
Easter Island	Jan 2014 - 30 May 2014	89	51	Molecular	33	18	Tognarelli (2016)
**Colombia**							
	Aug 2015 - 2016	18364		Unspecified			Sasmal (2018)
Cúcuta	22 Nov 2015 - 02 Apr 2016	4287		Symptoms			Sebrango-Rodriguez (2017)
Nieva	22 Nov 2015 - 02 Apr 2016	1940		Symptoms			Sebrango-Rodriguez (2017)
Arauca	2015 - 2017	788		Confirmed + Suspected			Charniga (2022)
Armenia	2015 - 2017	189		Confirmed + Suspected			Charniga (2022)
Barranquilla	2015 - 2017	4665		Confirmed + Suspected			Charniga (2022)
Bucaramanga	2015 - 2017	4322		Confirmed + Suspected			Charniga (2022)
Cali	2015 - 2017	16279		Confirmed + Suspected			Charniga (2022)
Cartagena	2015 - 2017	1021		Confirmed + Suspected			Charniga (2022)
Cúcuta	2015 - 2017	6485		Confirmed + Suspected			Charniga (2022)
Florencia	2015 - 2017	663		Confirmed + Suspected			Charniga (2022)
Ibagué	2015 - 2017	4076		Confirmed + Suspected			Charniga (2022)
Inírida	2015 - 2017	12		Confirmed + Suspected			Charniga (2022)
Leticia	2015 - 2017	278		Confirmed + Suspected			Charniga (2022)
Medellín	2015 - 2017	549		Confirmed + Suspected			Charniga (2022)
Mitú	2015 - 2017	17		Confirmed + Suspected			Charniga (2022)
Mocoa	2015 - 2017	57		Confirmed + Suspected			Charniga (2022)
Montería	2015 - 2017	1785		Confirmed + Suspected			Charniga (2022)
Neiva	2015 - 2017	3409		Confirmed + Suspected			Charniga (2022)
Pereira	2015 - 2017	463		Confirmed + Suspected			Charniga (2022)
Popayán	2015 - 2017	51		Confirmed + Suspected			Charniga (2022)
Puerto Carreño	2015 - 2017	17		Confirmed + Suspected			Charniga (2022)
Quibdó	2015 - 2017	14		Confirmed + Suspected			Charniga (2022)
Riohacha	2015 - 2017	279		Confirmed + Suspected			Charniga (2022)
San Andrés	2015 - 2017	1109		Confirmed + Suspected			Charniga (2022)
San José Del Guaviare	2015 - 2017	154		Confirmed + Suspected			Charniga (2022)
Santa Marta	2015 - 2017	1913		Confirmed + Suspected			Charniga (2022)
Sincelejo	2015 - 2017	856		Confirmed + Suspected			Charniga (2022)
Valledupar	2015 - 2017	788		Confirmed + Suspected			Charniga (2022)
Villavicencio	2015 - 2017	2377		Confirmed + Suspected			Charniga (2022)
Yopal	2015 - 2017	2121		Confirmed + Suspected			Charniga (2022)
	2015 - 2016	40741		Confirmed + Suspected			Flórez-Lozano (2020)
Girardot	19 Sep 2015 - 22 Jan 2016	1936	32	Molecular	1138		Rojas (2016)
Bucaramanga	13 Dec 2015 - 10 Sep 2016	3651		Symptoms			Sebrango-Rodriguez (2017)
Cali	13 Dec 2015 - 10 Sep 2016	12220		Symptoms			Sebrango-Rodriguez (2017)
	10 Oct 2015 - 24 Jun 2017	108087	9802	Molecular	70478	35977	Méndez (2017)
San Andres	06 Sep 2015 - 30 Jan 2016	928	52	Molecular	589		Rojas (2016)

continued on next page

continued from previous page

Outbreak location	Outbreak dates	Suspected cases	Confirmed cases	Mode of case detection	Female cases	Male cases	References
Barranquilla	01 Oct 2015 - 31 Dec 2015		1470	Symptoms	860	610	Towers (2016)
15 Unspecified Admin Regions	01 Jan 2016 - 13 Mar 2016		1593	Unspecified			Rocklov (2016)
Antioquia: Bajo Cauca: Caceres	01 Jan 2016 - 11 Apr 2016	45		Symptoms			Ospina (2017)
Antioquia: Bajo Cauca: Nechi	01 Jan 2016 - 11 Apr 2016	29		Symptoms			Ospina (2017)
Antioquia: Bajo Cauca: Caucasia	01 Jan 2016 - 11 Apr 2016	59		Symptoms			Ospina (2017)
Antioquia: Bajo Cauca: Zaragoza	01 Jan 2016 - 11 Apr 2016	49		Symptoms			Ospina (2017)
Antioquia: Valle Aburra: Medellin	01 Jan 2016 - 11 Apr 2016	351		Symptoms			Ospina (2017)
Antioquia: Valle Aburra: Bello	01 Jan 2016 - 11 Apr 2016	32		Symptoms			Ospina (2017)
Antioquia: Valle Aburra: Envigado	01 Jan 2016 - 11 Apr 2016	21		Symptoms			Ospina (2017)
Antioquia: Valle Aburra: Itagui	01 Jan 2016 - 11 Apr 2016	40		Symptoms			Ospina (2017)
Antioquia: Valle Aburra: Uraba Chigorodo	01 Jan 2016 - 11 Apr 2016	164		Symptoms			Ospina (2017)
Antioquia: Valle Aburra: San Pedro De Uraba	01 Jan 2016 - 11 Apr 2016	23		Symptoms			Ospina (2017)
Antioquia: Valle Aburra: Carepa	01 Jan 2016 - 11 Apr 2016	102		Symptoms			Ospina (2017)
Antioquia: Valle Aburra: Turbo	01 Jan 2016 - 11 Apr 2016	228		Symptoms			Ospina (2017)
Antioquia: Valle Aburra: Necocli	01 Jan 2016 - 11 Apr 2016	36		Symptoms			Ospina (2017)
Antioquia: Valle Aburra: Apartado	01 Jan 2016 - 11 Apr 2016	315		Symptoms			Ospina (2017)
Antioquia: Valle Aburra: Mutata	01 Jan 2016 - 11 Apr 2016	19		Symptoms			Ospina (2017)
Antioquia: Nordeste: Remedios	01 Jan 2016 - 11 Apr 2016	18		Symptoms			Ospina (2017)
Antioquia: Magdalena Medio: Puerto Berrio	01 Jan 2016 - 11 Apr 2016	61		Symptoms			Ospina (2017)
Antioquia: Magdalena Medio: Puerto Triunfo	01 Jan 2016 - 11 Apr 2016	27		Symptoms			Ospina (2017)
Antioquia: Magdalena Medio: Occidente Sospetran	01 Jan 2016 - 11 Apr 2016	17		Symptoms			Ospina (2017)
Antioquia: Magdalena Medio: Oriente Rionegro	01 Jan 2016 - 11 Apr 2016	18		Symptoms			Ospina (2017)
Florencia	01 Jan 2015 - 24 Nov 2018		611	Symptoms			Bonilla-Aldana (2020)
San Vicente Del Caguan	01 Jan 2015 - 24 Nov 2018		103	Symptoms			Bonilla-Aldana (2020)
San Jose Del Fragua	01 Jan 2015 - 24 Nov 2018		83	Symptoms			Bonilla-Aldana (2020)
El Doncello	01 Jan 2015 - 24 Nov 2018		75	Symptoms			Bonilla-Aldana (2020)
El Paujil	01 Jan 2015 - 24 Nov 2018		65	Symptoms			Bonilla-Aldana (2020)
Montanita	01 Jan 2015 - 24 Nov 2018		29	Symptoms			Bonilla-Aldana (2020)
Belen De Los Andaquies	01 Jan 2015 - 24 Nov 2018		26	Symptoms			Bonilla-Aldana (2020)
Solita	01 Jan 2015 - 24 Nov 2018		13	Symptoms			Bonilla-Aldana (2020)
Cartagena Del Chaira	01 Jan 2015 - 24 Nov 2018		12	Symptoms			Bonilla-Aldana (2020)
Morelia	01 Jan 2015 - 24 Nov 2018		9	Symptoms			Bonilla-Aldana (2020)
Milan	01 Jan 2015 - 24 Nov 2018		7	Symptoms			Bonilla-Aldana (2020)
Albania	01 Jan 2015 - 24 Nov 2018		5	Symptoms			Bonilla-Aldana (2020)
Valparaiso	01 Jan 2015 - 24 Nov 2018		5	Symptoms			Bonilla-Aldana (2020)
Solano	01 Jan 2015 - 24 Nov 2018		4	Symptoms			Bonilla-Aldana (2020)
Curillo	01 Jan 2015 - 24 Nov 2018		2	Symptoms			Bonilla-Aldana (2020)
**Curaçao**							
	16 Dec 2015 - 26 Apr 2017	2820	781	Molecular	574	207	Lim (2019)
**Dominican Republic**							
	30 Jan 2016 - 27 Feb 2016	573		Unspecified			Rocklov (2016)
	2015 - 2016	5161		Unspecified			Petrone (2021)
	01 Jan 2016 - 31 Dec 2016	5235		Unspecified			Bowman (2018)
**Ecuador**							
Portoviejo, Manabi	01 Jan 2016 - 31 Aug 2016	467	148	Molecular	107	41	Fors (2018)
2 Unspecified Admin Regions	01 Jan 2016 - 13 Mar 2016	96		Unspecified			Rocklov (2016)
**El Salvador**							
11 Unspecified Admin Regions	01 Jan 2016 - 13 Mar 2016	5618		Unspecified			Rocklov (2016)
**Federated States of Micronesia**							
Yap Island	01 Apr 2007 - 31 Jul 2007	185	108	Confirmed + Suspected	66		Duffy (2009)
**France**							
Austral Islands, French Polynesia	Oct 2013 - Mar 2014	1208		Unspecified			Riou (2017)
Mo'orea, French Polynesia	Oct 2013 - Mar 2014	1235		Unspecified			Riou (2017)

continued on next page

continued from previous page

Outbreak location	Outbreak dates	Suspected cases	Confirmed cases	Mode of case detection	Female cases	Male cases	References
Marquesas Islands, French Polynesia	Oct 2013 - Mar 2014	994		Unspecified			Riou (2017)
Sous-Le-Vent Islands, French Polynesia	Oct 2013 - Mar 2014	3912		Unspecified			Riou (2017)
Tahiti, French Polynesias	Oct 2013 - Mar 2014	21406		Unspecified			Riou (2017)
Tuamotus, French Polynesia	Oct 2013 - Mar 2014	1211		Unspecified			Riou (2017)
Guadeloupe	Jan 2016 - Oct 2016	30454		Unspecified			Riou (2017)
Martinique	Jan 2016 - Oct 2016	37295		Unspecified			Riou (2017)
Guadeloupe	Jan 2016 - 25 Feb 2016	389	35	Molecular			Daudens-Vaysse (2016)
Saint-Martin	Feb 2016 - 25 Feb 2016	58	11	Molecular			Daudens-Vaysse (2016)
French Guiana	Dec 2015 - 25 Feb 2016	1030		Unspecified			Daudens-Vaysse (2016)
Guadeloupe	29 Jan 2016 - 10 Mar 2016	717		Unspecified			Rocklov (2016)
Martinique	24 Nov 2015 - 25 Feb 2016	7600	203	Unspecified	142	61	Daudens-Vaysse (2016)
Martinique	21 Dec 2015 - 21 Jan 2016		102	Unspecified			Rocklov (2016)
Tahiti, French Polynesias	11 Oct 2013 - 28 Mar 2014	4966	128	Molecular			Kucharski (2016)
Sous-Le-Vent Islands, French Polynesia	11 Oct 2013 - 28 Mar 2014	1131	166	Molecular			Kucharski (2016)
Mo'orea, French Polynesia	11 Oct 2013 - 28 Mar 2014	440	22	Molecular			Kucharski (2016)
Tuamotu-Gambier, French Polynesia	11 Oct 2013 - 28 Mar 2014	612	9	Molecular			Kucharski (2016)
Marquesas Islands, French Polynesia	11 Oct 2013 - 28 Mar 2014	455	21	Molecular			Kucharski (2016)
Austral Islands, French Polynesia	11 Oct 2013 - 28 Mar 2014	733	36	Molecular			Kucharski (2016)
**France and the Netherlands**							
Saint Martin	Jan 2016 - Oct 2016	2519		Unspecified			Riou (2017)
**Guatemala**							
8 Unspecified Admin Regions	16 Nov 2015 - 15 Feb 2016			Unspecified			Rocklov (2016)
**India**							
Kerala, Punjab, Rajasthan, Uttar Pradesh, Jharkhand	May 2021 - Oct 2021		67	Molecular			Yadav (2022)
Tamil Nadu*	Jun 2017			Unspecified			Khan (2022)
Maharashtra*	31 Jul 2021			Unspecified			Khan (2022)
Madhya Pradesh*	30 Oct 2018			Unspecified			Khan (2022)
Uttar Pradesh*	23 Oct 2021			Unspecified			Khan (2022)
Rajasthan*	21 Sep 2018			Unspecified			Khan (2022)
Jaipur, Rajasthan	2018 - 16 Nov 2018	1925	153	Molecular			Sharma (2019)
Shastri Nagar And Surrounding Area Jaipur,	2018 - 16 Nov 2018	1925	153	Molecular			Sharma (2019)
Rajasthan	15 Sep 2018 - 22 Nov 2018		159	Molecular	104	55	Malhotra (2020)
Kerala*	08 Jul 2021			Unspecified			Khan (2022)
**Jamaica**							
	30 Jan 2016 - 30 Jul 2016	4567	72	Unspecified			Webster-Kerr (2016)
**Mexico**							
Chiapas State	30 Nov 2015 - 18 Dec 2015	119	25	Molecular			Guerbois (2016)
1 Unspecified Admin Region	01 Jan 2016 - 13 Mar 2016		129	Unspecified			Rocklov (2016)
**Netherlands**							
Bonaire	17 Oct 2016 - 26 Apr 2017	382	112	Molecular	81	31	Lim (2019)
**Panama**							
1 Unspecified Admin Region	01 Jan 2016 - 13 Mar 2016		49	Unspecified			Rocklov (2016)
**Singapore**							
	21 Aug 2016 - 30 Nov 2016		455	Molecular	192	263	Singapore Zika Study Group (2017)
**Suriname**							
	02 Oct 2015 - 23 Aug 2016		791	Molecular	553	238	Codrington (2018)
Brokopondo	02 Oct 2015 - 23 Aug 2016		1	Molecular			Codrington (2018)
Commewijne	02 Oct 2015 - 23 Aug 2016		19	Molecular			Codrington (2018)
Coronie	02 Oct 2015 - 23 Aug 2016		5	Molecular			Codrington (2018)
Marowoijne	02 Oct 2015 - 23 Aug 2016		4	Molecular			Codrington (2018)
Nickerie	02 Oct 2015 - 23 Aug 2016		39	Molecular			Codrington (2018)
Para	02 Oct 2015 - 23 Aug 2016		6	Molecular			Codrington (2018)
Paramaribo	02 Oct 2015 - 23 Aug 2016		358	Molecular			Codrington (2018)
Saramacca	02 Oct 2015 - 23 Aug 2016		11	Molecular			Codrington (2018)
Sipaliwini	02 Oct 2015 - 23 Aug 2016		4	Molecular			Codrington (2018)
Wanica	02 Oct 2015 - 23 Aug 2016		121	Molecular			Codrington (2018)
**Thailand**							
	Jan 2016 - Dec 2017	1717	368	Molecular	224		Ruchusatsawat (2019)
Samut Songkhram	2016 - 2018		2300	Symptoms			Sirinam (2022)
Ratchaburi, Bangkok	2006 - 2009		28	Molecular	15	13	Sriburin (2021)
**United States**							
Puerto Rico	26 Nov 2015 - 24 Feb 2016		198	Unspecified			Rocklov (2016)

continued on next page

continued from previous page

Outbreak location	Outbreak dates	Suspected cases	Confirmed cases	Mode of case detection	Female cases	Male cases	References
Wynwood Neighborhood, Miami-Dade County, Florida	26 Jun 2016 - 05 Aug 2016		21	Unspecified			Marini (2017)
Hidalgo County, Texas*	2016 - 2018			Unspecified			Hinojosa (2020)
Miami-Dade	07 Jul 2016 - 28 Dec 2016		256	Molecular			Philips (2019)
St. Croix, St. John, St. Thomas	03 Jan 2016 - 24 Jan 2018		1031	Molecular		680	Browne (2022)
St. Croix	03 Jan 2016 - 24 Jan 2018		257	Molecular			Browne (2022)
St. John	03 Jan 2016 - 24 Jan 2018		89	Molecular			Browne (2022)
St. Thomas	03 Jan 2016 - 24 Jan 2018		685	Molecular			Browne (2022)
Puerto Rico	01 Nov 2015 - 31 Dec 2016	39717	36390	Unspecified			Sharp (2020)
New York City	01 Jan 2016 - 30 Jun 2017	355	725	Molecular	519		Mcgibbon (2018)
Puerto Rico	01 Jan 2015 - 24 Nov 2018		21	Symptoms			Bonilla-Aldana (2020)
**Vietnam**							
Southern Vietnam	2016 - 2016	2190	214	Molecular			Phan (2019)

Table B.8: Overview of extracted outbreaks. Starred locations indicate outbreaks with reported dates but with no case numbers reported, which are included for completeness.

B.7.3 Delays

Parameter value	Parameter type	Uncertainty	Disaggregation	Sample size	Location	Dates	Population sample type	Population group	Reference
**Admission To Critical Care/ICU To Discharge From Critical Care/Icu**									
4 days	Median	Range: 1-30		20	VA Caribbean Healthcare System	01 Dec 2015 - 31 Oct 2016	Hospital	Other	Schirmer (2018)
**Admission To Discharge From Care**									
4 days	Median	Range: 1-6		3	VA hospitals in US	01 Dec 2015 - 31 Oct 2016	Hospital	Other	Schirmer (2018)
6 days	Median	Range: 1-214		91	VA Caribbean Healthcare System	01 Dec 2015 - 31 Oct 2016	Hospital	Other	Schirmer (2018)
17 days	Median			41	Hospital (Brazil)	Nov 2015 - Apr 2016	Hospital	Persons Under Investigation	Ferreira (2020)
22 days	Median	Range: 3-102		47	Barranquilla (Colombia)	01 Oct 2015 - 02 Apr 2016	Hospital	Other	Salinas (2017)
23 days	Median	IQR: 9.5-36		6117	Cucuta (Colombia)	29 Jun 2015 - 30 Jul 2016	Hospital	Persons Under Investigation	Anaya (2017)
**Diagnosis/Test Result To Death**									
39 days	Median	Range: 3-104		19	VA Caribbean Healthcare System	01 Dec 2015 - 31 Oct 2016	Hospital	Other	Schirmer (2018)
**Diagnosis/Test Result To Onset Of Neurologic Symptoms**									
7 days	Median	IQR: 2-14.5		29	Cucuta (Colombia)	29 Jun 2015 - 30 Jul 2016	Hospital	Other	Anaya (2017)
**Exposure/Infection To Recovery/Non-Infectiousness**									
9 days	Median	IQR: 8-10			Marseille, La Reunion (France)	Unspecified	Unspecified	Unspecified	Lequime (2020)
**Exposure/Infection To Recovery/Non-Infectiousness (Inverse Parameter)**									
0.125 per day	Unspecified				El Salvador	Oct 2015 - Apr 2016	Population	General Population	Kumar (2017)
**Extrinsic Incubation Period**									
6 days	Central				Guangzhou (China)	Unspecified			Guo (2020)
7.33 days	Mean	95% CrI: 6-9			Unspecified	Unspecified	Unspecified	Other	Armstrong (2020)
8.311 days	Mean	95% CrI: 4-16.962			Suriname	2015 - 22 Jun 2016	Population	Persons Under Investigation	Shutt (2017)
8.417 days	Mean	95% CrI: 4-17.035			El Salvador	2015 - 22 Jun 2016	Population	Persons Under Investigation	Shutt (2017)
8.88 days	Mean	95% CrI: 8-10			Unspecified	Unspecified	Unspecified	Other	Armstrong (2020)
10 days	Median	95% CrI: 6.3-14			French Polynesia (France)	Unspecified	Population	Persons Under Investigation	Kucharski (2016)
10 days	Central				Queensland (Australia)	Apr 2016			Hugo (2019)
10.5 days	Median	95% CrI: 8.6-12.4			Mo'orea, French Polynesia (France)	Oct 2013 - Apr 2014	Population	Persons Under Investigation	Champagne (2016)
10.5 days	Median	95% CrI: 8.6-12.6			Tahiti, French Polynesia (France)	Oct 2013 - Apr 2014	Population	Persons Under Investigation	Champagne (2016)
10.5 days	Median	95% CrI: 8.5-12.5			Tahiti, French Polynesia (France)	Oct 2013 - Apr 2014	Population	Persons Under Investigation	Champagne (2016)
10.6 days	Median	95% CrI: 8.7-12.5			Yap Island (Federated States of Micronesia)	01 Apr 2007 - 29 Jul 2007	Population	Persons Under Investigation	Champagne (2016)
10.6 days	Median	95% CrI: 8.6-12.6			Mo'orea, French Polynesia (France)	Oct 2013 - Apr 2014	Population	Persons Under Investigation	Champagne (2016)

continued on next page

continued from previous page

Parameter value	Parameter type	Uncertainty	Disaggregation	Sample size	Location	Dates	Population sample type	Population group	Reference
10.7 days	Median	95% CrI: 8.9-12.5			New Caledonia (France)	12 Nov 2013 - Aug 2014	Population	Persons Under Investigation	Champagne (2016)
10.7 days	Median	95% CrI: 8.8-12.7			Yap Island (Federated States of Micronesia)	01 Apr 2007 - 29 Jul 2007	Population	Persons Under Investigation	Champagne (2016)
10.8 days	Median	95% CrI: 8.9-12.8			New Caledonia (France)	12 Nov 2013 - Aug 2014	Population	Persons Under Investigation	Champagne (2016)
14 days	Central				Queensland (Australia)	Apr 2016			Hugo (2019)
10 - 14 days					Unspecified	Unspecified			Ryckebusch (2017)
5.1 - 24.2 days			Other		Unspecified	Unspecified			Winokur (2020)
17.8 - 24.2 days	Mean		Level of Exposure		Marseille, La Reunion (France)	Unspecified	Unspecified	Unspecified	Lequime (2020)
16.2 - 18.2 days			Other		Florida (United States)	Unspecified			Zimler (2021)
**Extrinsic Incubation Period (EIP)**									
9.7 - 25.6 E10	Central		Other		Unspecified	Unspecified	Other	Other	Blagrove (2020)
**Extrinsic Incubation Period (Inverse Parameter)**									
0.1 per day	Unspecified				Brazil	Jan 2016 - Dec 2016	Population	General Population	Dantas (2018)
0.004	Maximum likelihood	Other: 0.001-0.014			Colombia	2015 - 2016	Unspecified	Unspecified	Tonsing (2018)
**First Detection Of Anti-ZIKV IgM To Last Detection Of Anti-Zikv Igm**									
124 days	Mean	95% CI: 139-109		59	United States	13 Jul 2016 - 19 Sep 2017	Hospital	Persons Under Investigation	El Sahly (2019)
**First Infection In Country To First Reporting In Country**									
12 - 35 weeks	Median				Unspecified	Jan 2014 - Dec 2017	Population	General Population	Yamamoto (2019)
**Generation Time**									
2.5 weeks	Mean	SD: 0.7			Guadeloupe, Martinique, Saint-Martin (France)	2015 - 2017			Riou (2018)
**Incubation Period**									
4.9 days	Mean	95% CI: 4.59-5.21		111	Jaipur, Rajasthan (India)	22 Sep 2018 - 16 Nov 2018	Population	Persons Under Investigation	Sharma (2019)
5.563 days	Mean	95% CrI: 2-15.54		11825	El Salvador	2015 - 22 Jun 2016	Population	Persons Under Investigation	Shutt (2017)
5.8 days	Median	95% CI: 5.6-6.15		1946	Feira de Santana (Brazil)	01 Feb 2015 - 30 Apr 2017	Population	General Population	Lourenco (2017)
6 days	Mean	95% CI: 5.2-6.8		79	Travellers ¡2 weeks travel (United States)	01 Jan 2015 - 23 Jun 2016	Travel	Persons Under Investigation	Krow-Lucal (2017)
6 days	Median	Range: 2-10		111	Jaipur, Rajasthan (India)	22 Sep 2018 - 16 Nov 2018	Population	Persons Under Investigation	Sharma (2019)
6.4 days	Mean	95% CI: 5.7-7		197	All Travellers (United States)	01 Jan 2015 - 23 Jun 2016	Travel	Persons Under Investigation	Krow-Lucal (2017)
6.6 days	Median	95% CrI: 4.2-11			French Polynesia (France)	11 Oct 2013 - 28 Mar 2014	Population	Persons Under Investigation	Kucharski (2016)
6.614 days	Mean	95% CrI: 2-16.814		3042	Suriname	2015 - 22 Jun 2016	Population	Persons Under Investigation	Shutt (2017)
**Incubation Period (Inverse Parameter)**									
0.25 days	Central	95% CI: 0.183-0.317			Australes, French Polynesia (France)	Oct 2013 - Mar 2014	Population	General Population	Rahman (2019)

continued on next page

continued from previous page

Parameter value	Parameter type	Uncertainty	Disaggregation	Sample size	Location	Dates	Population sample type	Population group	Reference
0.225 days	Central	95% CI: 0.161-0.29			Tahiti, French Polynesia (France)	Oct 2013 - Mar 2014	Population	General Population	Rahman (2019)
0.086 days	Central	95% CI: 0.071-0.102			Tuamotu-Gambier, French Polynesia (France)	Oct 2013 - Mar 2014	Population	General Population	Rahman (2019)
0.084 days	Central	95% CI: 0.059-0.108			Mo'orea, French Polynesia (France)	Oct 2013 - Mar 2014	Population	General Population	Rahman (2019)
0.083 per day	Unspecified				Brazil	Jan 2016 - Dec 2016	Population	General Population	Dantas (2018)
0.069 per day	Unspecified				Colombia	2016 - 2016	Unspecified	Unspecified	Alzahrani (2021)
**Infectious Period**									
4.8 days	Median	95% CrI: 4.3-5.4			Managua (Nicaragua)	2016 - 2016	Population	General Population	Counotte (2019)
5.2 days	Median	95% CrI: 4.1-6.7			Tahiti, French Polynesia (France)	Oct 2013 - Apr 2014	Population	Persons Under Investigation	Champagne (2016)
5.2 days	Median	95% CrI: 4.1-6.7			Tahiti, French Polynesia (France)	Oct 2013 - Apr 2014	Population	Persons Under Investigation	Champagne (2016)
5.2 days	Median	95% CrI: 4.1-6.7			Yap Island (Federated States of Micronesia)	01 Apr 2007 - 29 Jul 2007	Population	Persons Under Investigation	Champagne (2016)
5.2 days	Median	95% CrI: 4.1-6.8			Mo'orea, French Polynesia (France)	Oct 2013 - Apr 2014	Population	Persons Under Investigation	Champagne (2016)
5.3 days	Median	95% CrI: 4.1-6.6			Yap Island (Federated States of Micronesia)	01 Apr 2007 - 29 Jul 2007	Population	Persons Under Investigation	Champagne (2016)
5.3 days	Median	95% CrI: 4.1-6.7			Mo'orea, French Polynesia (France)	Oct 2013 - Apr 2014	Population	Persons Under Investigation	Champagne (2016)
5.4 days	Median	95% CrI: 4.1-6.8			New Caledonia (France)	12 Nov 2013 - Aug 2014	Population	Persons Under Investigation	Champagne (2016)
5.5 days	Median	95% CrI: 4.2-6.8			New Caledonia (France)	12 Nov 2013 - Aug 2014	Population	Persons Under Investigation	Champagne (2016)
5.6 days	Median	95% CrI: 4-10			French Polynesia (France)	Unspecified	Population	Persons Under Investigation	Kucharski (2016)
5.9 days	Median	95% CI: 5.47-6.14		1946	Feira de Santana (Brazil)	01 Feb 2015 - 30 Apr 2017	Population	General Population	Lourenco (2017)
9.073 days	Mean	HPDI 95%: 5.956-12.232			Rio de Janeiro (Brazil)	2015 - 2016	Population	Other	Bastos (2018)
9.207 days	Mean	HPDI 95%: 7.283-11.134			Rio de Janeiro (Brazil)	2015 - 2016	Population	General Population	Bastos (2018)
9.599 days	Mean	95% CI: 7.673-11.701	Sex		Rio de Janeiro (Brazil)	2015 - 2016	Population	General Population	Bastos (2018)
11.568 days	Mean	HPDI 95%: 9.246-13.963			Rio de Janeiro (Brazil)	2015 - 2016	Population	General Population	Bastos (2018)
**Infectious Period (Inverse Parameter)**									
0.17 per day	Median	95% CrI: 0.1-0.23			Rio de Janeiro (Brazil)	2016 - 2016	Population	Persons Under Investigation	De Barros (2019)
0.083 days	Central	95% CI: 0.06-0.107			Sous-le-vent Islands, French Polynesia (France)	Oct 2013 - Mar 2014	Population	General Population	Rahman (2019)
0.083 days	Central	95% CI: 0.026-0.14			Marquesas Islands, French Polynesia (France)	Oct 2013 - Mar 2014	Population	General Population	Rahman (2019)
0.023 per day	Unspecified				Colombia	2016 - 2016	Unspecified	Unspecified	Alzahrani (2021)
0.023 per day	Unspecified				Unspecified	Unspecified	Unspecified	Unspecified	Ali (2022a)
0.02 - 0.1 per day	Unspecified		Method;Other		Florida (United States)	19 Jul 2016 - 29 Sep 2016	Population	Persons Under Investigation	Tuncer (2018)
0.114 - 0.333 per day	Unspecified		Method		Brazil	Jan 2016 - Dec 2016	Population	General Population	Dantas (2018)
**Latent Period (Inverse Parameter)**									

continued on next page

continued from previous page

Parameter value	Parameter type	Uncertainty	Disaggregation	Sample size	Location	Dates	Population sample type	Population group	Reference
0.069 per day	Unspecified				Colombia	Unspecified	Unspecified	Unspecified	Ali (2022a)
0.04	Maximum likelihood	Other: 0.02-0.07			Colombia	2015 - 2016	Unspecified	Unspecified	Tonsing (2018)
**NAAT-Detectable Infection To Seroconversion (IgM)**									
7.7 days	Mean	95% CI: 6.1-9.2		25	Florida, Puerto Rico, Texas, Nevada (United States)	06 Jul 2016 - 07 Mar 2017	Population	Persons Under Investigation	Stone (2020)
**NAAT-Detectable Infection To Seroreversion (IgM)**									
237 days	Mean	95% CI: 128.7-459.5		25	Florida, Puerto Rico, Texas, Nevada (United States)	06 Jul 2016 - 07 Mar 2017	Population	Persons Under Investigation	Stone (2020)
**Onset Of Antecedent Symptoms To Onset Of Neurologic Symptoms**									
6 days	Median	Range: 0-55		47	Barranquilla (Colombia)	01 Oct 2015 - 02 Apr 2016	Hospital	Other	Salinas (2017)
**Onset Of Neurologic Symptoms To Admission**									
9 days	Median	Range: 0-56		47	Barranquilla (Colombia)	01 Oct 2015 - 02 Apr 2016	Hospital	Other	Salinas (2017)
**Onset Of Neurologic Symptoms To Nadir Of Neurologic Symptoms**									
6 days	Median	IQR: 4-9		50	Salvador, Bahia (Brazil)	01 Jan 2015 - 31 Aug 2015	Hospital	Other	Styczynski (2017)
**Onset Of Previous Illness To Onset Of Neurologic Symptoms**									
8 days	Median	IQR: 5-15		50	Salvador, Bahia (Brazil)	01 Jan 2015 - 31 Aug 2015	Hospital	Other	Styczynski (2017)
**Onset To Admission**									
1 days	Median	IQR: 1-2		1936	Girardot (Colombia)	19 Oct 2015 - 22 Jan 2016	Hospital	Persons Under Investigation	Rojas (2016)
2 days	Median	95% CI: 0-7	Age	351	Ponce, Puerto Rico (United States)	2012 - 31 Dec 2016	Hospital	Children	Read (2018)
3.3 days	Mean	SD: 0.98		467	Portoviejo, Manabi (Ecuador)	01 Jan 2016 - 31 Aug 2016	Hospital	General Population	Fors (2018)
4 days	Median	IQR: 1-16		928	San Andrés (Colombia)	06 Sep 2015 - 30 Jan 2016	Hospital	Persons Under Investigation	Rojas (2016)
7 days	Median	IQR: 3-19		19	University Hospital Bulovka, Prague (Czech Republic)	Jan 2004 - Dec 2019	Travel	Persons Under Investigation	Trojanek (2023)
8 days	Median	IQR: 4-21		335	Cases acquired in travellers in Americas or Caribbean (Brazil)	01 Mar 2016 - 31 Dec 2019	Travel	Persons Under Investigation	Angelo (2020)
**Onset To Implementation Of Vector Control Measures**									
13 days	Median	Range: 4-58		625	Mainland, travel-related (France)	01 Jan 2016 - 15 Jul 2016	Population	Persons Under Investigation	Septfons (2016)
**Onset To Negative PCR Test In Saliva**									
3.1 days	Mean	SD: 1.5		51	Managua (Nicaragua)	31 Aug 2016 - 21 Oct 2016	Household	Household Contacts Of Survivors	Burger-Calderon (2018)
**Onset To Negative PCR Test In Serum/Plasma**									
2.56 days	Mean	SD: 1.53		25	Managua (Nicaragua)	31 Aug 2016 - 21 Oct 2016	Household	Household Contacts Of Survivors	Burger-Calderon (2018)

continued on next page

continued from previous page

Parameter value	Parameter type	Uncertainty	Disaggregation	Sample size	Location	Dates	Population sample type	Population group	Reference
**Onset To Negative PCR Test In Urine**									
5.94 days	Mean	SD: 3.8		86	Managua (Nicaragua)	31 Aug 2016 - 21 Oct 2016	Household	Household Contacts Of Survivors	Burger-Calderon (2018)
**Onset To Recovery/Non-Infectiousness**									
4.5 days	Mean	Range: 1.8		467	Portoviejo, Manabi (Ecuador)	01 Jan 2016 - 31 Aug 2016	Hospital	General Population	Fors (2018)
5 days	Median	IQR: 4-6		19	University Hospital Bulovka, Prague (Czech Republic)	Jan 2004 - Dec 2019	Travel	Persons Under Investigation	Trojanek (2023)
**Onset To Return To Area With Active Vectors**									
2 days	Median	Range: -7-10		625	Mainland, travel-related (France)	01 Jan 2016 - 15 Jul 2016	Population	Persons Under Investigation	Septfons (2016)
**Onset To Test Result**									
3.5 days	Median	IQR: 3-5		90	Bangkok, Samut Prakan, Samut Sakhon, Ratchaburi, Chon Buri (Thailand)	Mar 2020 - Mar 2023	Hospital	Persons Under Investigation	Khongwichit (2023)
14 days	Other			10	Recife and Manaus (Brazil)	Unspecified	Hospital	Persons Under Investigation	Batto-Menezes (2019)
**Onset To ZIKV IgG Detection**									
17 days	Median	IQR: 12-26		30	Padova and Pavia, travel-related (Italy)	Jan 2016 - Jan 2017	Hospital	Persons Under Investigation	Barzon (2018)
**Onset To ZIKV IgM Detection**									
8 days	Median	IQR: 5-15		30	Padova and Pavia, travel-related (Italy)	Jan 2016 - Jan 2017	Hospital	Persons Under Investigation	Barzon (2018)
**Onset To ZIKV RNA Clearance From Plasma**									
11.5 days	Median	IQR: 6-64		30	Padova and Pavia, travel-related (Italy)	Jan 2016 - Jan 2017	Hospital	Persons Under Investigation	Barzon (2018)
**Onset To ZIKV RNA Clearance From Saliva**									
14 days	Median	IQR: 8-31		26	Padova and Pavia, travel-related (Italy)	Jan 2016 - Jan 2017	Hospital	Persons Under Investigation	Barzon (2018)
**Onset To ZIKV RNA Clearance From Semen**									
25 days	Median	IQR: 14-29		10	Padova and Pavia, travel-related (Italy)	Jan 2016 - Jan 2017	Hospital	Persons Under Investigation	Barzon (2018)
**Onset To ZIKV RNA Clearance From Urine**									
24 days	Median	IQR: 17-34		29	Padova and Pavia, travel-related (Italy)	Jan 2016 - Jan 2017	Hospital	Persons Under Investigation	Barzon (2018)
**Recovery/Non-Infectiousness To Susceptibility (Inverse Parameter)**									
0.054 per day	Unspecified				Colombia	2016 - 2016	Unspecified	Unspecified	Alzahrani (2021)
0.054 per day	Unspecified				Unspecified	Unspecified	Unspecified	Unspecified	Ali (2022a)
**Reporting To Intervention**									
5 days	Median	Range: 2-38		625	Mainland, travel-related (France)	01 Jan 2016 - 15 Jul 2016	Population	Persons Under Investigation	Septfons (2016)
**Serial Interval**									
7.4 days	Mean	95% CI: 4.59-10.2		10	Singapore	Unspecified	Community	Persons Under Investigation	Singapore Zika Study Group (2017)
2.2 - 4.7 weeks	Mean		Region		French Polynesia (France)	Oct 2013 - Oct 2016	Population	Persons Under Investigation	Riou (2017)

Table B.9: Overview of extracted delay parameters.

B.7.4 Seroprevalence

Seroprev- alence type	Parameter value	Uncert- ainty	Disag- gregation	Num- erator	Denom- inator	Sample size	PRNT on ELISA + tests	Location	Dates	Population sample type	Population group	Reference
**Argentina**												
IgM				5	73	104			Apr 2016 - Mar 2017	Other	Persons Under Investigation	Tellechea (2018)
**Austria**												
IgG	0.1 %			1	1001	1001		Tyrol	Aug 2016 - Nov 2016	Population	Blood Donors	Borena (2017)
IgM	0.3 %			3	1001	1001		Tyrol	Aug 2016 - Nov 2016	Population	Blood Donors	Borena (2017)
PRNT	0 %			0	4	1001	TRUE	Tyrol	Aug 2016 - Nov 2016	Population	Blood Donors	Borena (2017)
**Bangladesh**												
IgG	17 %			17	92	92			2014 - 2014	Population	Persons Under Investigation	Geurtsvankessel (2018)
IgG	28 %			15	52	52			2015 - 2015	Population	Persons Under Investigation	Geurtsvankessel (2018)
IgM	%			1	418	418			2011 - 2015	Population	Persons Under Investigation	Geurtsvankessel (2018)
IgM	%			3	418	418			2011 - 2015	Population	General Population	Geurtsvankessel (2018)
PRNT	3.1 %		Time	12	16	418	TRUE		2011 - 2015	Population	General Population	Geurtsvankessel (2018)
PRNT	4.3 %		Time	5	15	418	TRUE		2011 - 2015	Population	Persons Under Investigation	Geurtsvankessel (2018)
**Bolivia**												
IgG	1.9 %		Age, Sex	3	162	162		La Paz	Mar 2017 - Apr 2017	Population	General Population	Villarroel (2018)
IgG	2.6 %		Age, Sex	4	152	152		Cochabamba	Mar 2017 - Apr 2017	Population	General Population	Villarroel (2018)
IgG	9.7 %		Age, Sex	19	196	196		Tarija	Mar 2017 - Apr 2017	Population	General Population	Villarroel (2018)
IgG	61 %		Age, Sex	122	200	200		Santa Cruz	Mar 2017 - Apr 2017	Population	General Population	Villarroel (2018)
IgG	61.9 %		Age, Sex	65	105	105		Beni	Dec 2016 - Dec 2016	Population	General Population	Villarroel (2018)
PRNT	0 %	95% CI: 0-0	Age, Sex	0	4	4		La Paz	Mar 2017 - Apr 2017	Population	General Population	Villarroel (2018)
PRNT	0 %	95% CI: 0-0	Age, Sex	0	7	7		Cochabamba	Mar 2017 - Apr 2017	Population	General Population	Villarroel (2018)
PRNT	0.5 %	95% CI: 0-1.5	Age, Sex	1	22	22		Tarija	Mar 2017 - Apr 2017	Population	General Population	Villarroel (2018)
PRNT	21.5 %	95% CI: 16-27	Age, Sex	43	134	134		Santa Cruz	Mar 2017 - Apr 2017	Population	General Population	Villarroel (2018)
PRNT	39 %	95% CI: 30-48	Age, Sex	41	70	70		Beni	Mar 2017 - Apr 2017	Population	General Population	Villarroel (2018)
**Brazil**												
HAI/HI	27.7 - 36.6 %		Age		298	298		Manaus (Amazonas state)	Jan 2014 - Dec 2015	Other	Other	Salgado (2021)
HAI/HI	52.6 %		Age	235	447	447		Manaus	Jan 2015 - Dec 2015	Community	Other	Salgado (2023)
IgG	0 %			1	132	132		Recife	2016 - 2017	Population	Children	Alves (2020)
IgG	0.55 %			1	182	182		Rio Grande do Sul	05 Dec 2016 - 06 Jan 2017	Hospital	Blood Donors	Diefenbach (2019)
IgG	0 - 3.4 %		Age		135	135		Diamantina	2018 - 2019	Population	Pregnant Women	Santos (2023)
IgG	3 %			63	2749	2749		Rio de Janeiro	Jul 2018 - Oct 2018	Population	General Population	Perisse (2020)
IgG	4.15 %			7	140	140		São Paulo	May 2016 - May 2018	Population	Blood Donors	Lira (2022)
IgG	6.8 %			6	88	88		Maranhao state	2016 - 2018	Hospital	Persons Under Investigation	Ribeiro (2020)

continued on next page

continued from previous page

Seroprev- alence type	Parameter value	Uncert- ainty	Disag- gregation	Num- erator	Denom- inator	Sample size	PRNT on ELISA + tests	Location	Dates	Population sample type	Population group	Reference
IgG	7 %	95% CI: 4-10			249	249		Salvador, Bahia	Oct 2014 - Oct 2014	Population	General Population	Rodriguez-Barraquer (2019)
IgG	7.25 %				262	262		Recife	Jan 2016 - Dec 2016	Population	Pregnant Women	Castanha (2019)
IgG	8 %	95% CI: 6-10			675	675		Salvador, Bahia	Mar 2015 - Mar 2015	Population	General Population	Rodriguez-Barraquer (2019)
IgG	8.3 %			20	240	240		Pernambuco State	Dec 2015 - Jun 2017	Hospital	Pregnant Women	Ximenes (2019)
IgG	0 - 21.4 %							Ribeirão Preto, São Paulo State	Jul 2010 - May 2017	Population	General Population	Slavov (2020)
IgG	15 %	95% CI: 4-29			751	751		Porto Velho	Jul 2016 - Jun 2019	Other	Other	Botosso (2023)
IgG	15 %		Other, Time	11	72	72		São Paulo state	Jan 2016 - Sep 2016	Hospital	Persons Under Investigation	Tozetto-Mendoza (2019)
IgG	17.3 %			34	196	196		Maranhao state	Apr 2017 - Jun 2018	Hospital	Pregnant Women	Branco (2021)
IgG	19 %			43	227	227		Salvador, Bahia	01 Jan 2015 - 31 Jan 2017	Hospital	Pregnant Women	Aromolaran (2022)
IgG	19 %	IQR: 16-22			227	227		Salvador, Bahia	01 Jan 2015 - 31 Jan 2017	Hospital	Pregnant Women	Aromolaran (2022)
IgG	22 %	95% CI: 7-41			817	817		Belo Horizonte	Jul 2016 - Jun 2019	Other	Other	Botosso (2023)
IgG	29 %	95% CI: 11-52			494	494		Salvador, Bahia	Jul 2016 - Jun 2019	Other	Other	Botosso (2023)
IgG	30 %	95% CI: 17-46			345	345		Fortaleza	Jul 2016 - Jun 2019	Other	Other	Botosso (2023)
IgG	33.87 %			63	186	186		Manaus	May 2021 - Oct 2021	Population	Other	Neto (2024)
IgG	35 %	95% CI: 26-48	Region		5108	5108		Belo Horizonte, Salvador, Laranjeiras, Fortaleza, Recife, Cuiaba and Campo Grande, Porto Velho	Jul 2016 - Jun 2019	Other	Other	Botosso (2023)
IgG	0.1 - 74.7 %		Age, Method, Other, Sex		2070	2070		Recife	Aug 2018 - Feb 2019	Household	General Population	Braga (2023)
IgG	38.6 %	95% CI: 31.3-47		56	144	144		University Hospital Professor Edgard Santos (UHPES) in Salvador de Bahia	Aug 2017 - Feb 2018	Hospital	Other	Moreira-Soto (2020)
IgG	40 %	95% CI: 30-72			1017	1017		Cuiaba and Campo Grande	Jul 2016 - Jun 2019	Other	Other	Botosso (2023)
IgG	43.95 %			98	223	223		Salvador, Bahia	May 2021 - Oct 2021	Population	Other	Neto (2024)
IgG	46.7 %			163	349	349		Rio de Janeiro	Jul 2017 - Dec 2017	Hospital	Pregnant Women	Wittlin (2022)
IgG	47 %	95% CI: 25-69			1110	1110		Recife	Jul 2016 - Jun 2019	Other	Other	Botosso (2023)
IgG				216	404	404		Juazeiro do Norte	Jun 2018 - Dec 2018	Community	General Population	De Almeida Barreto (2020)
IgG	15 - 94 %		Time						Jan 2016 - Sep 2016	Hospital	Persons Under Investigation	Tozetto-Mendoza (2019)
IgG	57 %	95% CI: 35-74			574	574		Laranjeiras	Jul 2016 - Jun 2019	Other	Other	Botosso (2023)
IgG	57 %			172	302	302		Salvador, Bahia	Sep 2017 - Nov 2017	Hospital	Other	Bastos Filho (2023)

continued on next page

continued from previous page

Seroprev- alence type	Parameter value	Uncert- ainty	Disag- gregation	Num- erator	Denom- inator	Sample size	PRNT on ELISA + tests	Location	Dates	Population sample type	Population group	Reference
IgG	59 %	95% CI: 50.7-66.7		85	144	144		University Hospital Professor Edgard Santos (UHPES) in Salvador de Bahia	Feb 2016 - May 2016	Hospital	Other	Moreira-Soto (2020)
IgG			Age, Other	393	642	642		Salvador, Bahia	Oct 2015 - Oct 2015	Population	Other	Rodriguez-Barraquer (2019)
IgG	61.3 %	95% CI: 52.9-69.7		81	132	132		Recife	2016 - 2017	Population	Pregnant Women	Alves (2020)
IgG	63 %	95% CI: 60-65	Age, Region		1453	1453		Salvador, Bahia	Oct 2015 - Oct 2015	Population	General Population	Rodriguez-Barraquer (2019)
IgG	63.3 %	95% CI: 59.4-66.8	Other, Time	401	633	633		Salvador, Bahia	25 Nov 2015 - 28 May 2016	Mixed	Mixed Groups	Netto (2017)
IgG	64 %	IQR: 61-67			188	188		Salvador, Bahia	01 Jan 2015 - 31 Jan 2017	Hospital	Pregnant Women	Aromolaran (2022)
IgG	68 %			127	188	188		Salvador, Bahia	01 Jan 2015 - 31 Jan 2017	Hospital	Pregnant Women	Aromolaran (2022)
IgG	72.3 %			73	101	101		Salvador, Bahia	Jan 2016 - Dec 2016	Population	Pregnant Women	Duarte (2020)
IgG	74.7 %			71	95	95		Salvador, Bahia	18 Jan 2016 - 16 Dec 2016	Hospital	Other	Oliveira (2020)
IgG	80.6 %	95% CI: 76.4-84.1	Age, Sex	323	401	425		Pernambuco State	Apr 2017 - Dec 2017	Household	Mixed Groups	Magalhaes (2021)
IgG	80.9 %			17	21	21		Salvador, Bahia	18 Jan 2016 - 16 Dec 2016	Hospital	Other	Oliveira (2020)
IgG	86.7 %			78	90	90		Maranhao state	2016 - 2018	Hospital	Persons Under Investigation	Ribeiro (2020)
IgG	88.6 %			62	70	70		Bahia	Mar 2015 - Feb 2016	Hospital	Children	Venturi (2019)
IgG	96.7 %			87	90	90		Bahia	Mar 2015 - Feb 2016	Hospital	Pregnant Women	Venturi (2019)
IgG and IgM				54	214	219		Rio de Janeiro	Jan 2015 - Aug 2016	Hospital	Pregnant Women	João (2018)
IgM	0 %			0	132	132		Recife	2016 - 2017	Population	Pregnant Women	Alves (2020)
IgM	0 %			0	223	223		Salvador, Bahia	May 2021 - Oct 2021	Population	Other	Neto (2024)
IgM	0 %			0	186	186		Manaus	May 2021 - Oct 2021	Population	Other	Neto (2024)
IgM	1.3 %		Other, Time	8	633	633		Salvador, Bahia	25 Nov 2015 - 28 May 2016	Mixed	Mixed Groups	Netto (2017)
IgM	1.7 %			6	349	349		Rio de Janeiro	Jun 2017 - Dec 2017	Hospital	Pregnant Women	Wittlin (2022)
IgM				9	404	404		Juazeiro do Norte	Jun 2018 - Dec 2018	Community	General Population	De Almeida Barreto (2020)
IgM	2.4 %			1	41	1289		Fortaleza	Feb 2018 - Dec 2018	Community	Other	Frota (2023)
IgM	2.85 %			4	140	140		São Paulo	May 2016 - May 2018	Population	Blood Donors	Lira (2022)
IgM	3 %			6	196	196		Maranhao state	Apr 2017 - Jun 2018	Hospital	Pregnant Women	Branco (2021)
IgM	4.2 %			4	95	95		Salvador, Bahia	18 Jan 2016 - 16 Dec 2016	Hospital	Other	Oliveira (2020)
IgM	5.6 %				262	262		Recife	Jan 2016 - Dec 2016	Population	Pregnant Women	Castanha (2019)
IgM				1	16	16		Northeast Brazil	Dec 2014 - Feb 2017	Hospital	Persons Under Investigation	Leonhard (2021)

continued on next page

continued from previous page

Seroprev- alence type	Parameter value	Uncert- ainty	Disag- gregation	Num- erator	Denom- inator	Sample size	PRNT on ELISA + tests	Location	Dates	Population sample type	Population group	Reference
IgM	7.1 %			5	70	70		Bahia	Mar 2015 - Feb 2016	Hospital	Children	Venturi (2019)
IgM	10.2 %			71	694	694		Pernambuco State	Dec 2015 - Jun 2017	Hospital	Pregnant Women	Ximenes (2019)
IgM				3	15	15		Northeast Brazil	Dec 2014 - Feb 2017	Hospital	Persons Under Investigation	Leonhard (2021)
IgM				9	43	43		Central-West	Jan 2017 - Apr 2019	Hospital	Pregnant Women	Rosado (2023)
IgM	23.8 %			5	21	21		Salvador, Bahia	18 Jan 2016 - 16 Dec 2016	Hospital	Other	Oliveira (2020)
IgM				17	68	68		Northeast Brazil	Dec 2014 - Feb 2017	Hospital	Persons Under Investigation	Leonhard (2021)
IgM	36 %			30	84	84		Salvador, Bahia	01 Jan 2015 - 31 Aug 2015	Hospital	Other	Styczynski (2017)
IgM	37 %			41	112	112		Rio de Janeiro	2015 - 2016	Population	Persons Under Investigation	Brasil (2020)
IgM	41 %			17	41	41		Salvador, Bahia	01 Jan 2015 - 31 Aug 2015	Hospital	Other	Styczynski (2017)
IgM	44.4 %			40	90	90		Bahia	Mar 2015 - Feb 2016	Hospital	Pregnant Women	Venturi (2019)
IgM	6 - 86 %		Time					São Paulo state	Jan 2016 - Sep 2016	Hospital	Persons Under Investigation	Tozetto-Mendoza (2019)
IgM				19	41	41		Hospital da Restauração, Recife, Pernambuco,	Nov 2015 - Apr 2016	Hospital	Persons Under Investigation	Ferreira (2020)
IgM	84.8 %			39	48	48		Salvador, Bahia	Jan 2015 - Dec 2016	Community	Pregnant Women	Aguilar Ticona (2021)
PRNT	1 %	95% CI: 0-4			101	101		Salvador, Bahia	Mar 2015 - Mar 2015	Population	General Population	Rodriguez-Barraquer (2019)
PRNT	0 - 16.9 %						TRUE	Ribeirão Preto, São Paulo State	Jul 2010 - May 2017	Population	General Population	Slavov (2020)
PRNT				5	43	43		Central-West	Jan 2017 - Apr 2019	Hospital	Pregnant Women	Rosado (2023)
PRNT	20.5 %			49	239	239			Jun 2011 - Mar 2012	Other	Children	Zambrano (2021)
PRNT	30 %			24	85	85	TRUE	University Hospital Professor Edgard Santos (UHPES) in Salvador de Bahia	Feb 2016 - May 2016	Hospital	Other	Moreira-Soto (2020)
PRNT	40.54 %			15	37	262	TRUE	Recife	Jan 2016 - Dec 2016	Population	Pregnant Women	Castanha (2019)
PRNT	42.5 %			40	94	94		Maranhao state	2016 - 2018	Hospital	Persons Under Investigation	Ribeiro (2020)
PRNT	55.8 %			324	581	581		Pernambuco State	Dec 2015 - Jun 2017	Hospital	Pregnant Women	Ximenes (2019)
PRNT	61.2 %			287	469	469		Salvador (Hospital Geral Roberto Santos)	Oct 2015	Population	Pregnant Women	Nery (2021)
PRNT	62.5 %		Other, Time	187	299	299		Salvador, Bahia	25 Nov 2015 - 28 May 2016	Mixed	Mixed Groups	Netto (2017)
PRNT	65.7 %	95% CI: 61-70.1	Age, Sex	272	414	425		Pernambuco State	Apr 2017 - Dec 2017	Household	Mixed Groups	Magalhaes (2021)
PRNT	76 %			64	84	84		Salvador, Bahia	01 Jan 2015 - 31 Aug 2015	Hospital	Other	Styczynski (2017)

continued on next page

continued from previous page

Seroprev- alence type	Parameter value	Uncert- ainty	Disag- gregation	Num- erator	Denom- inator	Sample size	PRNT on ELISA + tests	Location	Dates	Population sample type	Population group	Reference
PRNT	84.8 %			39	46	70		Bahia	Mar 2015 - Feb 2016	Hospital	Children	Venturi (2019)
PRNT	88 %			36	41	41		Salvador, Bahia	01 Jan 2015 - 31 Aug 2015	Hospital	Other	Styczynski (2017)
PRNT	90 %			81	90	90		Bahia	Mar 2015 - Feb 2016	Hospital	Pregnant Women	Venturi (2019)
**Burkina Faso**												
IgG	45.7 %	95% CI: 41.39-50.08		229	501	501		Ouagadougou, Bobo-Dioulasso	2020 - 2020	Population	Blood Donors	Tinto (2022)
PRNT	22.75 %	95% CI: 19.29-26.62		114	229	501	TRUE	Ouagadougou, Bobo-Dioulasso	2020 - 2020	Population	Blood Donors	Tinto (2022)
**Cabo Verde**												
IgG	10.9 %			47	431	431		Santiago Island: Praia	24 Aug 2014 - 04 Nov 2014	Population	General Population	Ward (2022)
IgG				311	1226	1226			Oct 2015 - 2016	Population	Persons Under Investigation	Faye (2020)
IgM				15	64	64			05 Oct 2015 - 29 May 2016	Population	Persons Under Investigation	Lourenco (2018)
PRNT				311	311	1226	TRUE		Oct 2015 - 2016	Population	Persons Under Investigation	Faye (2020)
**Cambodia**												
PRNT	4			2	50	770		Kampong Speu	Jul 2018 - Aug 2018	Other	Children	Odio (2024)
**Cameroon**												
PRNT	2 %			3	150	150		Maroua	Aug 2015 - Oct 2015	Other	Blood Donors	Gake (2017)
PRNT	2			3	149	149		Ngaoundere	Aug 2015 - Oct 2015	Other	Blood Donors	Gake (2017)
PRNT	3.3			9	272	272		Yaounde	Aug 2015 - Oct 2015	Other	Blood Donors	Gake (2017)
PRNT	4.8 %			9	186	186		Garoua	Aug 2015 - Oct 2015	Other	Blood Donors	Gake (2017)
PRNT	7.6			12	157	157		Bertoua	Aug 2015 - Oct 2015	Other	Blood Donors	Gake (2017)
PRNT	10			17	170	170		Douala	Aug 2015 - Oct 2015	Other	Blood Donors	Gake (2017)
**China**												
IgG	9.5 %			26	273	273		Nanning City, Guanxi	Mar 2019 - Mar 2019	Population	General Population	Zhou (2020)
IgM	0 %			0	150	150		Affiliated Hospital of Jining Medical University	Oct 2013 - Jun 2017	Hospital	Persons Under Investigation	Hao (2019)
IgM	1.8 %			5	273	273		Nanning City, Guanxi	Mar 2019 - Mar 2019	Population	General Population	Zhou (2020)
PRNT	65.4 %			17	26	273	TRUE	Nanning City, Guanxi	Mar 2019 - Mar 2019	Population	General Population	Zhou (2020)
**Colombia**												
IgG	0 - 5.35 %	95% CI: 0-16.74	Time		390	390		Barranquilla	06 May 2015 - 31 Aug 2015	Population	General Population	Bayona-Pacheco (2019)
IgG	64.9 %		Method	213	328	328		Santander	Unspecified	School	Mixed Groups	Cardenas (2020)
IgG	86.8 %			99	114	115		Risaralda	22 Nov 2017 - 05 Jun 2019	Hospital	Pregnant Women	Cardona-Ospina (2022)
IgG	89 %			80	90	90		Cereté: Córdoba state	01 May 2016 - 31 May 2016	Hospital	Pregnant Women	Marbán-Castro (2020)

continued on next page

continued from previous page

Seroprev- alence type	Parameter value	Uncert- ainty	Disag- gregation	Num- erator	Denom- inator	Sample size	PRNT on ELISA + tests	Location	Dates	Population sample type	Population group	Reference
IgG	100 %			29	29	29		Cucuta	29 Jun 2015 - 30 Jul 2016	Hospital	Other	Anaya (2017)
IgM	0.8 %			9	1080	1108			Feb 2017 - Mar 2019	Population	Children	Tannis (2024)
IgM	1 %			1	90	90		Cereté: Córdoba state	01 May 2016 - 31 May 2016	Hospital	Pregnant Women	Marbán-Castro (2020)
IgM	4.3 %			1519	66	1519			Feb 2017 - Mar 2019	Population	Pregnant Women	Tannis (2024)
IgM	7 %			22	301	301			01 Dec 2015 - 31 Dec 2019	Hospital	Children	Castro-Trujillo (2024)
NS1 BOB ELISA	1 %				703	703			2013 - 2014	Population	General Population	Nascimento (2019)
NS1 BOB ELISA	32 %				703	703			2017 - 2018	Population	General Population	Nascimento (2019)
PRNT	2.63 %			3	114	115	TRUE	Risaralda	22 Nov 2016 - 05 Jun 2019	Other	Pregnant Women	Cardona-Ospina (2022)
PRNT	3 %				703	703	TRUE		2013 - 2014	Population	General Population	Nascimento (2019)
PRNT	16 %				703	703	TRUE		2017 - 2018	Population	General Population	Nascimento (2019)
PRNT	47.6 %			339	712	712			Jun 2011 - Mar 2012	Other	Children	Zambrano (2021)
**Czech Republic**												
IgG	42.1 %			8	19	19		University Hospital Bulovka, Prague	Jan 2004 - Dec 2019	Travel	Persons Under Investigation	Trojanek (2023)
IgM	63.1 %			12	19	19		University Hospital Bulovka, Prague	Jan 2004 - Dec 2019	Travel	Persons Under Investigation	Trojanek (2023)
PRNT	84.2 %			16	19	19		University Hospital Bulovka, Prague	Jan 2004 - Dec 2019	Travel	Persons Under Investigation	Trojanek (2023)
**Dominican Republic**												
IgM	3.2 %			6	189	189			2016	Travel	Other	Voss (2020)
PRNT	3.2 %			6	6	189	TRUE		2016	Travel	Other	Voss (2020)
**DRC**												
IgG	3.5 %			34	978	978			2013 - 2014	Population	Children	Wilcox (2018)
PRNT	0.1 %	95% CI: 0-0.5		1	31		TRUE		2013 - 2014	Population	Children	Wilcox (2018)
**Ecuador**												
NS1 BOB ELISA	29 %	95% CI: 25-33	Age, Region, Sex		1126	1126		Borbón, Maldonado, Timbiré, Santa Maria, Santo Domingo, Colon Eloy	Aug 2019 - Oct 2019	Community	General Population	Andrade (2024)
NS1 BOB ELISA	42 %	95% CI: 39-45	Age, Other, Region, Sex		1192	1192		Borbón, Maldonado, Timbiré, Santa Maria, Santo Domingo, Colon Eloy	Jul 2018 - Oct 2018	Community	General Population	Andrade (2024)
**El Salvador**												
IgM	22.7 %			45	198	198		Sonsonate	Mar 2022 - Sep 2022	Population	Pregnant Women	Lynn (2024)
**Ethiopia**												

continued on next page

continued from previous page

Seroprev-alence type	Parameter value	Uncert-ainty	Disag- gregation	Num-erator	Denom-inator	Sample size	PRNT on ELISA + tests	Location	Dates	Population sample type	Population group	Reference
IgG				65	1645	1645			May 2014 - Jul 2014	Community	General Population	Tsegaye (2018)
IgG	27.3 %	95% CI: 20.7-35.1		41	150	150		Itang special district, Lare district	Oct 2018 - Jun 2019	Community	General Population	Asebe (2021)
PRNT	0.4 %		Region	7	65	1645	TRUE		May 2014 - Jul 2014	Community	General Population	Tsegaye (2018)
**Federated States of Micronesia**												
IgM	74 %		Sex	414	557	557		Yap Island	01 Apr 2007 - 31 Jul 2007	Household	General Population	Duffy (2009)
**Fiji**												
MIA	6.7 %	95% CI: 4.5-9.4		30	451	778		Central division	Sep 2013 - Nov 2013	Population	Persons Under Investigation	Kama (2019)
MIA	9 %	95% CI: 5.8-13		24	268	778		Western division	Sep 2013 - Nov 2013	Population	Persons Under Investigation	Kama (2019)
MIA	11.9 %	95% CI: 4.9-22.9		7	59	778		Northern division	Sep 2013 - Nov 2013	Population	Persons Under Investigation	Kama (2019)
MIA	6.1 - 25.2 %		Age, Sex, Time						Sep 2013 - Nov 2015	Population	Persons Under Investigation	Kama (2019)
MIA	21.9 %	95% CI: 17.9-26.8		73	333	333		Central division	Oct 2015 - Nov 2015	Population	Persons Under Investigation	Kama (2019)
**France**												
IgG	0.8 %		Level of Exposure	5	593	593		French Polynesia	Jul 2011 - Oct 2013	Population	Mixed Groups	Aubry (2015)
IgG	22 %	95% CI: 16-28		154	700	700		Society Islands, French Polynesia	Sep 2015 - Nov 2015	Population	General Population	Aubry (2017)
IgG	26 %			25	98	98		Papeete, Tahiti, French Polynesia	Nov 2013 - Feb 2014	Hospital	General Population	Cao-Lormeau (2016)
IgG				51	179	179		Guadeloupe	Apr 2016 - Mar 2017	Population	Pregnant Women	Prisant (2019)
IgG	37 %	95% CI: 26-47		18	49	49		Society Islands, French Polynesia	Feb 2014 - Mar 2014	Population	General Population	Aubry (2017)
IgG	45 %	95% CI: 38-52		22	49	49		Tuamotu Islands, French Polynesia	Feb 2014 - Mar 2014	Population	General Population	Aubry (2017)
IgG	57 %	95% CI: 47-68		28	49	49		Marquesas Islands, French Polynesia	Feb 2014 - Mar 2014	Population	General Population	Aubry (2017)
IgG	59 %	95% CI: 39-80		29	49	49		Austral–Gambier Islands, French Polynesia	Feb 2014 - Mar 2014	Population	General Population	Aubry (2017)
IgG	66 %	95% CI: 60-71		312	476	476		Society Islands, French Polynesia	May 2014 - Jun 2014	School	Children	Aubry (2017)
IgM	17 %			17	98	98		Papeete, Tahiti, French Polynesia	Nov 2013 - Feb 2014	Hospital	General Population	Cao-Lormeau (2016)
IgM	23.3 %	95% CI: 0-62	Region		2697	2697		22 munici-palities of French Guiana	Jun 2017 - Oct 2017	Population	General Population	Flamand (2019)
IgM	26.1 %			76	291	291		French Guiana	01 Jan 2015 - 15 Jul 2016	Mixed	Children	Pomar (2020)
MIA	0 - 45.6 %		Region		2697	2697		French Guiana	Jun 2017 - Oct 2017	Population	General Population	Bailly (2021)

continued on next page

continued from previous page

Seroprev- alence type	Parameter value	Uncert- ainty	Disag- gregation	Num- erator	Denom- inator	Sample size	PRNT on ELISA + tests	Location	Dates	Population sample type	Population group	Reference
PRNT	56 %			54	98	98		Papeete, Tahiti, French Polynesia	Nov 2013 - Feb 2014	Hospital	General Population	Cao-Lormeau (2016)
PRNT	76 %			78	102	102		Tahiti, Mo'orea, French Polynesia	01 Jun 2013 - 31 Aug 2014	Hospital	Pregnant Women	Subissi (2018)
PRNT	95 %			20	21	21		Tahiti, Mo'orea, French Polynesia	01 Jun 2013 - 31 Aug 2014	Hospital	Pregnant Women	Subissi (2018)
**Gabon**												
IgG	29.1 - 55 %		Age, Sex, Time		462	462		Lambarene	Nov 2014 - Jan 2017	Population	General Population	Ushijima (2021)
**Germany**												
IgG	1.2 %			1	81	81		Germany travelling to Asia	Jan 2017 - Jan 2018	Travel	Persons Under Investigation	Dammermann (2023)
IgM	1.2 %			1	81	81		Germany travelling to Asia	Jan 2017 - Jan 2018	Travel	Persons Under Investigation	Dammermann (2023)
**Ghana**												
IgG			Age, Sex	3	160	160		Greater Accra Regional Hospital	Dec 2016 - Nov 2017	Hospital		Ankrah (2019)
IgM			Age, Sex	30	160	160		Greater Accra Regional Hospital	Dec 2016 - Nov 2017	Hospital		Ankrah (2019)
**Guatemala**												
NS1 BOB ELISA	10 %			20	196	196		Coastal lowlands of SW Guatemala	Oct 2015 - Nov 2015	Population	Children	Lamb (2022)
NS1 BOB ELISA	37 %			69	186	186		Coastal lowlands of SW Guatemala	Jan 2016 - Feb 2016	Population	Children	Lamb (2022)
PRNT	0 - 14 %		Method		196	196		Coastal lowlands of SW Guatemala	Oct 2015 - Nov 2015	Population	Children	Lamb (2022)
PRNT	19 - 40 %		Method		186	186		Coastal lowlands of SW Guatemala	Jan 2016 - Feb 2016	Population	Children	Lamb (2022)
**Guinea**												
IgM	14.7 %	95% CI: 8.8-22.4	Time	17	116	116		Faranah Prefecture Hospital	May 2018 - Jul 2021	Hospital	Persons Under Investigation	Bayandin (2023)
**Guyana**												
IgG	9.82 %							Cayenne, Kourou, Saint Laurent	Jan 2016 - Dec 2016	Hospital	Pregnant Women	Hallet (2020)
IgG			Other	837	6654	6654		Cayenne, Kourou, Saint Laurent	Jan 2016 - Dec 2016	Hospital	Pregnant Women	Hallet (2020)
IgG	16.5 %							Cayenne, Kourou, Saint Laurent	Jan 2016 - Dec 2016	Hospital	Pregnant Women	Hallet (2020)
**Honduras**												
IgM	1.7 - 66.3 %		Method		60	60		Tegucigalpa	Jul 2016 - Dec 2016	Hospital	Children	Alger (2024)

continued on next page

continued from previous page

Seroprev- alence type	Parameter value	Uncert- ainty	Disag- gregation	Num- erator	Denom- inator	Sample size	PRNT on ELISA + tests	Location	Dates	Population sample type	Population group	Reference
NS1 BOB ELISA	1 %				223	223			2013 - 2014	Population	General Population	Nascimento (2019)
NS1 BOB ELISA	57 %				223	223			2017 - 2018	Population	General Population	Nascimento (2019)
PRNT	2 %				223	223	TRUE		2013 - 2014	Population	General Population	Nascimento (2019)
PRNT	23 %				223	223	TRUE		2017 - 2018	Population	General Population	Nascimento (2019)
PRNT	79.3 %			180	227	227			Jun 2011 - Mar 2012	Other	Children	Zambrano (2021)
**Hungary**												
IFA				1	219	219			2017 - 2017	Travel	General Population	Nagy (2019)
IFA				1	188	188			2018 - 2018	Travel	General Population	Nagy (2019)
IFA				5	196	196			2016 - 2016	Travel	General Population	Nagy (2019)
**India**												
IgG	6.66 %			5	75	75		Bhopal region	Unspecified	Unspecified	Blood Donors	Nema (2024)
IgG	15.38 %			10	75	75		Bhopal region	Unspecified	Unspecified	Blood Donors	Nema (2024)
PRNT				4	29	29			May 2021 - Oct 2021	Unspecified	Persons Under Investigation	Yadav (2022)
**Indonesia**												
IgM	0			0	45	45			Oct 2016 - Apr 2017	Population	Children	Putri (2020)
IgM	0			0	113	113			Oct 2016 - Apr 2017	Population	Children	Putri (2020)
PRNT	2			1	46	46			Oct 2016 - Apr 2017	Population	Children	Putri (2020)
PRNT	2			2	110	110			Oct 2016 - Apr 2017	Population	Children	Putri (2020)
PRNT	9.1 %	95% CI: 3.95-11.01	Other, Region	60	662	662		Aceh, North Sumatra, West Sumatra, Jambi, Lampung, Banten, DKI Jakarta, West Java, Central Java, East Java, Bali, East Kalimantan, South Sulawesi, Southeast Sulawesi	Oct 2014 - Nov 2014	Population	Children	Tedjo Sasmono (2018)
PRNT	13 %		Sex	9	71	71		Dasan Geres and Kelayu, Lombok	Unspecified	Community	Other	Olson (1983)
PRNT	6 - 100		Other		116	116		Aceh Jaya	2017 - 2017	Population	General Population	Harapan (2022)
**Iran**												
IgG	0 %			0	494	494		Hormozgan province	Sep 2016 - Jun 2017	Mixed	Mixed Groups	Ziyaeyan (2018)
**Italy**												
IgM	1.3 %			2	156	156		Bari	Mar 2015 - Jun 2017	Travel	Persons Under Investigation	Loconsole (2018)
IgM	16.7 %			5	30	30			Nov 2015 - Nov 2022	Travel	Persons Under Investigation	Merakou (2023)
IgM	21.4 %			104	487	497			Nov 2015 - Nov 2022	Travel	Persons Under Investigation	Merakou (2023)
IgM	57.1 %			4	7	7			Nov 2015 - Nov 2022	Travel	Persons Under Investigation	Merakou (2023)
IgM	77.3 %			34	44	44			Nov 2015 - Nov 2022	Travel	Persons Under Investigation	Merakou (2023)

continued on next page

continued from previous page

Seroprev- alence type	Parameter value	Uncert- ainty	Disag- gregation	Num- erator	Denom- inator	Sample size	PRNT on ELISA + tests	Location	Dates	Population sample type	Population group	Reference
PRNT	16 %			95	594	594			Nov 2015 - Nov 2022	Travel	Persons Under Investigation	Merakou (2023)
PRNT	86 %			38	44	44			Nov 2015 - Nov 2022	Travel	Persons Under Investigation	Merakou (2023)
**Kenya**												
HAI/HI	1.3 %		Age, Other	14	1042	1042		Kitui District	Nov 1966 - Apr 1968	Population	General Population	Geser (1970)
HAI/HI	3.3 %		Age, Other	27	822	822		Central Nyanza	Nov 1966 - Apr 1968	Population	General Population	Geser (1970)
HAI/HI	52 %		Age, Other	434	834	834		Malindi District	Nov 1966 - Apr 1968	Population	General Population	Geser (1970)
IgG	0 %			0	135	135		Eldoret	Jan 2013 - Feb 2013	Population	Other	Kisuya (2019)
IgG	0 %			0	96	96		Nairobi	Aug 2013 - Sep 2013	Population	Other	Kisuya (2019)
IgG	0.9 %			5	577	577		Eldoret, Kisumu, Nairobi	Mar 2009 - Jan 2014	Population	Mixed Groups	Kisuya (2019)
IgG	1.4 %			3	212	212		Nairobi	Mar 2009 - Nov 2012	Population	Other	Kisuya (2019)
IgG	1.5 %			2	134	134		Kisumu	Dec 2013 - Jan 2014	Population	Other	Kisuya (2019)
IgM	7.2 %			166	2293	1916		Mombasa	Oct 2017 - Jul 2019	Hospital	Pregnant Women	Osoro (2022)
PRNT	0 %			0	25	25		Prigan, Turkana	Aug 2017 - Aug 2017	Population	General Population	Chepkorir (2019)
PRNT	0 %			0	61	61		Kare Edome, Turkana	Aug 2017 - Aug 2017	Population	General Population	Chepkorir (2019)
PRNT	0 %			0	69	69		Nayanaesanyati, Turkana	Aug 2017 - Aug 2017	Population	General Population	Chepkorir (2019)
PRNT	0 %			0	56	56		Old Gunner, Turkana	Aug 2017 - Aug 2017	Population	General Population	Chepkorir (2019)
PRNT	0 %			0	139	139		Lowarengak, Turkana	Aug 2017 - Aug 2017	Population	General Population	Chepkorir (2019)
PRNT				0	11	11	TRUE	Eldoret, Kisumu, Nairobi	Mar 2009 - Jan 2014	Population	Persons Under Investigation	Kisuya (2019)
PRNT	0.24 %		Age, Region	1	413	413		Turkana	Aug 2017 - Aug 2017	Population	General Population	Chepkorir (2019)
PRNT	1.59 %			1	63	63		Elelea, Turkana	Aug 2017 - Aug 2017	Population	General Population	Chepkorir (2019)
PRNT	1.67 %			1	60	60		Longarkau, West Pokot	Feb 2016 - Feb 2016	Population	General Population	Chepkorir (2019)
PRNT				3	144	1916	TRUE	Mombasa	Oct 2017 - Jul 2019	Hospital	Pregnant Women	Osoro (2022)
PRNT	3.85 %			2	52	52		Pkotong, West Pokot	Feb 2016 - Feb 2016	Population	General Population	Chepkorir (2019)
PRNT	4.11 %			3	73	73		Shaba, West Pokot	Feb 2016 - Feb 2016	Population	General Population	Chepkorir (2019)
PRNT	7.11 %		Age, Region	33	464	464		West Pokot	Feb 2016 - Feb 2016	Population	General Population	Chepkorir (2019)
PRNT	8 %			8	100	100		Kokipei, West Pokot	Feb 2016 - Feb 2016	Population	General Population	Chepkorir (2019)
PRNT	9.09 %			6	66	66		Sangakai, West Pokot	Feb 2016 - Feb 2016	Population	General Population	Chepkorir (2019)
PRNT	11.5 %			13	113	113		Kanyerus, West Pokot	Feb 2016 - Feb 2016	Population	General Population	Chepkorir (2019)
**Laos**												
IgG	17.3 %			62	359	359		Vientiane capital	2003 - 2004	Population	Blood Donors	Pastorino (2019)
IgG	27.9 %			192	687	687		Vientiane capital	2015 - 2015	Population	Blood Donors	Pastorino (2019)
PRNT	3.9 - 10.1 %	95% CI:	Age, Sex, Time				TRUE	Vientiane capital	2003 - 2015	Population	Blood Donors	Pastorino (2019)
**Madagascar**												

continued on next page

continued from previous page

Seroprev- alence type	Parameter value	Uncert- ainty	Disag- gregation	Num- erator	Denom- inator	Sample size	PRNT on ELISA + tests	Location	Dates	Population sample type	Population group	Reference
IgG	4.9 %		Age, Region, Sex, Time		1036	1036			Jan 2018 - Jun 2021	Hospital	Other	Rakotomalala (2023)
**Malaysia**												
IgG	0 %			0	22	22		Paya Pelong	Unspecified	Community	General Population	Khor (2020)
IgG				15	418	418		Sabah	2018 - 2018	Hospital	Blood Donors	Tun (2022)
IgG	4 %			4	101	101		Sangwai	Unspecified	Community	General Population	Khor (2020)
IgG	4.5 %			3	67	67		Jekjok	Unspecified	Community	General Population	Khor (2020)
IgG	5.3 %			1	19	19		Pasir Intan	Unspecified	Community	General Population	Khor (2020)
IgG	5.9 %			1	17	17		Bukit Payung	Unspecified	Community	General Population	Khor (2020)
IgG	6.9 %			6	87	87		Tumboh Hangat	Unspecified	Community	General Population	Khor (2020)
IgG	7.7 %			3	39	39		Semanggar	Unspecified	Community	General Population	Khor (2020)
IgG				36	400	400		Sabah	2017 - 2017	Hospital	Persons Under Investigation	Tun (2022)
IgG	9.4 %			12	90	90		Paya Sendayan	Unspecified	Community	General Population	Khor (2020)
IgG				58	498	498			Unspecified	Community	General Population	Khor (2020)
IgG	13.2 %		Region, Sex	115	872	872			Unspecified	Community	General Population	Khor (2020)
IgG				57	374	374			Unspecified	Community	General Population	Khor (2020)
IgG	17.3 %			14	81	81		Lubuk Legong	Unspecified	Community	General Population	Khor (2020)
IgG	17.4 %			8	46	46		Ulu Kelaka	Unspecified	Community	General Population	Khor (2020)
IgG	21.3 %			10	47	47		Paya Lebar	Unspecified	Community	General Population	Khor (2020)
IgG	22 %			20	91	91		Sungai Perah	Unspecified	Community	General Population	Khor (2020)
IgG	22.5 %			8	45	45		Donglai Baru	Unspecified	Community	General Population	Khor (2020)
IgG	22.5 %			27	120	120		Dusun Kubur	Unspecified	Community	General Population	Khor (2020)
IgG	41.7 %			244	585	585		"Peninsula"	Unspecified	Population	General Population	Khor (2024)
IgM				16	418	418		Sabah	2018 - 2018	Hospital	Blood Donors	Tun (2022)
IgM				38	400	400		Sabah	2017 - 2017	Hospital	Persons Under Investigation	Tun (2022)
NS1 BOB ELISA	7.6 %	95% CI: 6.1-9.3	Age, Other, Sex	82	1085	1085		Kuala Lumpur	2012 - 2017	Mixed	Mixed Groups	Sam (2019)
PRNT	0.5 %	95% CI: 0.13-1.8		2	38	38	TRUE	Sabah	2017 - 2017	Hospital	Persons Under Investigation	Tun (2022)
PRNT	1.4 %	95% CI: 0.65-3		6	16	26	TRUE	Sabah	2018 - 2018	Hospital	Blood Donors	Tun (2022)
PRNT	1.4 %	95% CI: 0.65-3		6	16	26	TRUE	Sabah	2018 - 2018	Hospital	Blood Donors	Tun (2022)
PRNT	3.3 %	95% CI: 2.4-4.6		36	82	1085	TRUE	Kuala Lumpur	2012 - 2017	Mixed	Mixed Groups	Sam (2019)
PRNT	6 %	95% CI: 4-8		24	38	38	TRUE	Sabah	2017 - 2017	Hospital	Persons Under Investigation	Tun (2022)
**Mali**												
IgG	6.2 %			4	65	65		Niono	Oct 2016 - Nov 2016	Population	General Population	Diarra (2020)
IgG	8.5 %			11	129	129		Bamako	Oct 2016 - Nov 2016	Population	General Population	Diarra (2020)
IgG	10.5 %			67	637	637		Bamako	2013 - 2013	Population	General Population	Diarra (2020)
IgG	16.2 %			22	136	136		Kadiolo	Oct 2016 - Nov 2016	Population	General Population	Diarra (2020)

continued on next page

continued from previous page

Seroprev- alence type	Parameter value	Uncert- ainty	Disag- gregation	Num- erator	Denom- inator	Sample size	PRNT on ELISA + tests	Location	Dates	Population sample type	Population group	Reference
IgG	17.3 %			22	127	127		Bougouni	Oct 2016 - Nov 2016	Population	General Population	Diarra (2020)
IgG	17.5 %			7	40	40		Kita	Oct 2016 - Nov 2016	Population	General Population	Diarra (2020)
IgG	19 %	95% CI: 13.6-24.4		38	200	200		Soromba, Sibirila, Bougouni, Sikasso	Feb 2015 - Feb 2015	Population	General Population	Bane (2024)
IgG	24.5 %	95% CI: 18.5-30.5		49	200	200		Banzana, Sibirila, Bougouni, Sikasso	Feb 2015 - Feb 2015	Population	General Population	Bane (2024)
IgG	34 %	95% CI: 27.4-40.6		68	200	200		Bamba, Sibirila, Bougouni, Sikasso	Feb 2015 - Feb 2015	Population	General Population	Bane (2024)
IgG	35.8 %			39	109	109		Diema	Oct 2016 - Nov 2016	Population	General Population	Diarra (2020)
IgG	43.3 %			81	187	187		Bandiagara	Oct 2016 - Nov 2016	Population	General Population	Diarra (2020)
PRNT	0.3 %	95% CI: 0.01-1.9	Age, Sex					Bamako	Aug 2007 - Oct 2007			Marchi (2020)
PRNT	0.4 %	95% CI: 0.01-2.42	Age, Sex					Bamako	Nov 2011 - Apr 2012			Marchi (2020)
PRNT	3.1 %			2	8	65	TRUE	Niono	Oct 2016 - Nov 2016	Population	General Population	Diarra (2020)
PRNT	5.4 %			7	11	129	TRUE	Bamako	Oct 2016 - Nov 2016	Population	General Population	Diarra (2020)
PRNT	7.4 %			47	85	637	TRUE	Bamako	2013 - 2013	Population	General Population	Diarra (2020)
PRNT	10.3 %			14	23	136	TRUE	Kadiolo	Oct 2016 - Nov 2016	Population	General Population	Diarra (2020)
PRNT	11.8 %			15	25	127	TRUE	Bougouni	Oct 2016 - Nov 2016	Population	General Population	Diarra (2020)
PRNT	15 %			6	12	40	TRUE	Kita	Oct 2016 - Nov 2016	Population	General Population	Diarra (2020)
PRNT	15.5 %			29	105	187	TRUE	Bandiagara	Oct 2016 - Nov 2016	Population	General Population	Diarra (2020)
PRNT	20.2 %			22	53	109	TRUE	Diema	Oct 2016 - Nov 2016	Population	General Population	Diarra (2020)
**Mexico**												
IgG	5.5 %				126	126		Oaxaca	Dec 2017 - Feb 2018	Hospital	Children	Porras-Garcia (2024)
IgG	28.6 %			36	126	126		Oaxaca	Dec 2017 - Feb 2018	Hospital	Pregnant Women	Porras-Garcia (2024)
IgG	2.4 - 27.1		Age	286	768	768		Merida	2020 - 2020	Hospital	Persons Under Investigation	Earnest (2024)
IgG	62 %		Age	85	136	136		Chiapas	Feb 2019 - Aug 2019	Hospital	Pregnant Women	Eligio-García (2020)
IgM	8 %		Other, Symptoms	9	113	113		Monterrey City	01 Aug 2017 - 30 Jun 2018	Hospital	Persons Under Investigation	Gongora-Rivera (2020)
IgM	12 %		Other, Symptoms	3	26	26		Monterrey City	01 Aug 2017 - 30 Jun 2018	Hospital	Persons Under Investigation	Gongora-Rivera (2020)
IgM	31 %			39	126	126		Oaxaca	Dec 2017 - Feb 2018	Hospital	Pregnant Women	Porras-Garcia (2024)
NS1 BOB ELISA	0 %				266	266			2013 - 2014	Population	General Population	Nascimento (2019)
NS1 BOB ELISA	31 %				266	266			2017 - 2018	Population	General Population	Nascimento (2019)
PRNT				2	263	48		Guerrero	May 2019 - Nov 2019	Hospital	Other	Nunez-Avellaneda (2021)
PRNT				6	263	60		Guerrero	May 2019 - Nov 2019	Hospital	Other	Nunez-Avellaneda (2021)
PRNT	3 %				266	266	TRUE		2013 - 2014	Population	General Population	Nascimento (2019)

continued on next page

continued from previous page

Seroprev- alence type	Parameter value	Uncert- ainty	Disag- gregation	Num- erator	Denom- inator	Sample size	PRNT on ELISA + tests	Location	Dates	Population sample type	Population group	Reference
PRNT	7 %				266	266	TRUE		2017 - 2018	Population	General Population	Nascimento (2019)
PRNT	38.3 %			103	269	269			Jun 2011 - Mar 2012	Other	Children	Zambrano (2021)
PRNT	62.1 %			74	119	119		Chiapas State	30 Nov 2015 - 18 Dec 2015	Population	General Population	Guerbois (2016)
PRNT				9	13	13		Oaxaca	Dec 2017 - Feb 2018	Hospital	Children	Porras-Garcia (2024)
**Mozambique**												
IgM	2.5 %	95% CI: 8.5-17.4		5	193	193		South Region	2009 - 2015	Population	Other	Chelene (2019)
IgM	4.7 %	95% CI: 3.9-9		17	356	356		Central Region	2009 - 2015	Population	Other	Chelene (2019)
IgM	4.9 %	95% CI: 3.5-6.6	Age, Sex, Time	42	850	850			2009 - 2015	Population	Other	Chelene (2019)
IgM	6.6 %	95% CI: 10.2-17		20	301	301		North Region	2009 - 2015	Population	Other	Chelene (2019)
**Myanmar**												
IgM	31.3 %			89	284	284		Yangon	2018 - 2018	Population	General Population	Tun (2020a)
**Netherlands**												
IgG	0.6 %	95% CI: 0.1-2.1		2	326	326			Mar 2014 - Oct 2017	Travel	General Population	Overbosch (2023)
IgG	14 %			18	129	129			Mar 2014 - Oct 2017	Travel	General Population	Overbosch (2023)
**New Zealand**												
IgG	58.3 %		Age, Sex	35	60	60		Aitutaki	Jan 2017 - Apr 2017	Population	General Population	Saretzki (2024)
IgG	67.8 %		Age, Sex	141	208	208		Rarotonga, Cook Islands	Jan 2017 - Apr 2017	Population	General Population	Saretzki (2024)
**Nicaragua**												
IgG	66.4 %			89	134	134		León	05 Feb 2017	Hospital	Pregnant Women	Zepeda (2023)
IgG	80.4 %			82	102	102		León	05 Feb 2017	Hospital	Pregnant Women	Zepeda (2023)
IgG	94 %			176	187	187		León	Feb 2017 - Jul 2017	Hospital	Pregnant Women	Collins (2020)
IgM			Age, Sex, Symptoms	23	142	142		Managua	31 Aug 2016 - 21 Oct 2016	Household	Household Contacts Of Survivors	Burger-Calderon (2018)
NS1 BOB ELISA	36.1 %	95% CI: 34.5-37.8	Age, Other, Region, Sex					District II, Managua	Feb 2017 - Jul 2017	Household	Children	Zambrana (2018)
NS1 BOB ELISA	45.8 %	95% CI: 43.4-48.3	Age, Sex					District II, Managua	Feb 2017 - Jul 2017	Household	General Population	Zambrana (2018)
NS1 BOB ELISA	56.4 %	95% CI: 53.1-59.6	Age, Other, Region, Sex					District II, Managua	Feb 2017 - Jul 2017	Household	Other	Zambrana (2018)
PRNT	61 %			107	176	176	TRUE	León	Feb 2017 - Jul 2017	Hospital	Pregnant Women	Collins (2020)
**Nigeria**												
HAI/HI	56 %		Age	150	267			Kainji Lake	1980 - 1980			Adekolu-John (1983)
IgG	0.4 %		Symptoms	4	1006	1006		Jos	01 Apr 2019 - 31 Jan 2022	Hospital	Pregnant Women	Ogwuche (2023)
IgG	3.6 %		Sex, Symptoms	17	468	468		Plateau, Nasarawa	Jan 2016 - Dec 2016	Community	General Population	Mathe (2018)
IgG	12 %	95% CI: 9.9-14.4		12	100	100		Cross River State	17 Mar 2017 - 30 May 2017	Population	Persons Under Investigation	Otu (2020)
IgG	18.3 %			55	300	300		Baru-Dokp Teaching Hospital, Kaduna, Kaduna State	Dec 2020 - Nov 2021	Hospital	Mixed Groups	Mac (2023a)

continued on next page

continued from previous page

Seroprev- alence type	Parameter value	Uncert- ainty	Disag- gregation	Num- erator	Denom- inator	Sample size	PRNT on ELISA + tests	Location	Dates	Population sample type	Population group	Reference
IgG	18.6 %		Age, Other, Region, Sex	82	419	871		Nasarawa	Dec 2020 - Nov 2021	Hospital	Mixed Groups	Peter Asaga (2023)
IgG	18.7 %		Age, Other, Region, Sex	56	300	871		Kaduna	Dec 2020 - Nov 2021	Hospital	Mixed Groups	Peter Asaga (2023)
IgG	18.9 %			79	419	419		Federal Medical Centre, Keffi, Nasarawa State.	Dec 2020 - Nov 2021	Hospital	Mixed Groups	Mac (2023a)
IgG	19.2 %		Age, Other, Region, Sex	167	871	871		Abia State University Teaching Hospital, Aba, Abia state, Federal Medical Centre, Keffi, Nasarawa State, Baru-Dokp Teaching Hospital, Kaduna, Kaduna State	Dec 2020 - Nov 2021	Hospital	Mixed Groups	Mac (2023a)
IgG	19.2 %	95% CI: 0.16-0.21	Age, Other, Region, Sex, Symptoms	167	871	871		3 states in Nigeria Nigeria, Abia State in Southern Nigeria, and Kaduna	Dec 2020 - Nov 2021	Hospital	Mixed Groups	Mac (2023a)
State in Northern Nigeria	Dec 2020 - Nov 2021	Hospital	General Population	Mac (2023b)	871	871		3 states in Nigeria Nigeria, Abia State in Southern Nigeria, and Kaduna	Dec 2020 - Nov 2021	Hospital	Mixed Groups	Mac (2023a)
IgG	21.7 %			33	152	152		Abia State University Teaching Hospital, Aba, Abia state	Dec 2020 - Nov 2021	Hospital	Mixed Groups	Mac (2023a)
IgG	22.4 %		Age, Other, Region, Sex	34	152	871		Abia	Dec 2020 - Nov 2021	Hospital	Mixed Groups	Peter Asaga (2023)
IgG	0 - 62.7 %		Age, Other, Region, Sex		871	871		Abia, Kaduna, Nasarawa	Dec 2020 - Nov 2021	Hospital	Mixed Groups	Peter Asaga (2023)
IgG	85 %			170	200	200		Borno state	Apr 2018 - Aug 2018	Population	Persons Under Investigation	Baba (2023)
IgG and IgM	20 %	95% CI: 11.9-30.2		20	100	100		Cross River State	17 Mar 2017 - 30 May 2017	Population	Persons Under Investigation	Otu (2020)
IgM	1.4 %		Symptoms	14	1006	1006		Jos	01 Apr 2019 - 31 Jan 2022	Hospital	Pregnant Women	Ogwuche (2023)
IgM	6.2 %		Sex, Symptoms	29	468	468		Plateau, Nasarawa	Jan 2016 - Dec 2016	Community	General Population	Mathe (2018)
IgM	10 %	95% CI: 3.8-20.1		10	100	100		Cross River State	17 Mar 2017 - 30 May 2017	Population	Persons Under Investigation	Otu (2020)
IgM	12.1 %		Other	60	496	496		Adamawa, Bauchi, and Borno states	Apr 2018 - Aug 2018	Population	Persons Under Investigation	Baba (2023)

continued on next page

continued from previous page

Seroprev-alence type	Parameter value	Uncert-ainty	Disag- gregation	Num-erator	Denom-inator	Sample size	PRNT on ELISA + tests	Location	Dates	Population sample type	Population group	Reference
IgM	14.5 %			29	96	200		Borno state	Apr 2018 - Aug 2018	Population	Persons Under Investigation	Baba (2023)
IgM	15.5 %			31	200	200		Adamawa state	Apr 2018 - Aug 2018	Population	Persons Under Investigation	Baba (2023)
IgM	22 %			30	137	200		Borno state	Mar 2018 - Apr 2018	Hospital	Persons Under Investigation	Oderinde (2020)
IgM	25 %			51	200	200		Borno state	Mar 2018 - Apr 2018	Hospital	Persons Under Investigation	Oderinde (2020)
IgM	29.4 %			53	180	180		Zaria	Unspecified	Hospital	Pregnant Women	Adekola (2023)
PRNT	3 %			6	200	200		Adamawa state	Apr 2018 - Aug 2018	Population	Persons Under Investigation	Baba (2023)
PRNT	9.4 %			16	170	200		Borno state	Apr 2018 - Aug 2018	Population	Persons Under Investigation	Baba (2023)
PRNT	12 %			24	200	200		Borno state	Apr 2018 - Aug 2018	Population	Persons Under Investigation	Baba (2023)
PRNT	13.1 %		Other	65	496	496		Adamawa, Bauchi, and Borno states	Apr 2018 - Aug 2018	Population	Persons Under Investigation	Baba (2023)
PRNT	30 %			6	20	20		Igbo-Ora	May 1975 - Oct 1975	Population	Other	Fagbami (1977)
PRNT	36.5 %			35	96	96		Bauchi state	Apr 2018 - Aug 2018	Population	Persons Under Investigation	Baba (2023)
**Pakistan**												
IgG	6.48 %			47	725	725		Faisalabad	2019 - 2019	Population		Chen (2024)
PRNT	0.69 %			5	47	725	TRUE	Faisalabad	2019 - 2019	Population	General Population	Chen (2024)
PRNT	6.48 %			47	47	725	TRUE	Faisalabad	2019 - 2019	Population	General Population	Chen (2024)
**Panama**												
MIA	30.3 %			121	400	400			2015 - 2016	Population	Persons Under Investigation	Eskildsen (2020)
**Papua New Guinea**												
IgG	66 %			87	132	132		Wewak	Apr 2019 - Apr 2019	Other	Other	Grant (2022b)
IgG	69.7 %			53	76	76		Manus Island	Apr 2019 - Apr 2019	Other	Other	Grant (2022b)
IgM	9 %			7	76	76		Manus Island	Apr 2019 - Apr 2019	Other	Other	Grant (2022b)
IgM	9.1 %			12	132	132		Wewak	Apr 2019 - Apr 2019	Other	Other	Grant (2022b)
PRNT	63.2 %			48	55	76	TRUE	Manus Island	Apr 2019 - Apr 2019	Other	Other	Grant (2022b)
PRNT	65.9 %			87	93	132	TRUE	Wewak	Apr 2019 - Apr 2019	Other	Other	Grant (2022b)
**Paraguay**												
IgG	31.4 %			49	156	156		Central department and Asuncion	Jan 2018 - May 2018	Hospital	Other	Rojas (2019)
**Peru**												
IgG	2.5 %				200	200		Chincha Baja	Mar 2019 - May 2019	Population	General Population	Cachay (2021)
IgG	23.5 %				200	200		Pueblo Nuevo	Mar 2019 - May 2019	Population	General Population	Cachay (2021)
**Philippines**												
Biotinylated-												
EDIII antigen												
capture ELISA	18 %			98	547	547		Balamban and Bogo City in Cebu Province	2017 - 2017	Other	Children	Adams (2021)

continued on next page

continued from previous page

Seroprev- alence type	Parameter value	Uncert- ainty	Disag- gregation	Num- erator	Denom- inator	Sample size	PRNT on ELISA + tests	Location	Dates	Population sample type	Population group	Reference
IgG	23 %			118	508				2016 - 2016	Hospital	Persons Under Investigation	Biggs (2020)
IgG	31.5 %			314	997	997			2016 - 2016	Population	Persons Under Investigation	Biggs (2021)
IgM	0 - 1 %				508	508			2016 - 2016	Hospital	Persons Under Investigation	Biggs (2020)
**Republic of the Congo**												
IgG	5.7 %				386	386		Brazzaville, Pointe-Noire, Ewo, Gamboma, Oyo, Owando	Mar 2011 - Jul 2011	Other	Blood Donors	Nurtop (2020)
PRNT	1.1 %						TRUE	Brazzaville	Mar 2011 - Jul 2011	Other	Blood Donors	Nurtop (2020)
PRNT	1.8 %				386	386	TRUE	Brazzaville, Pointe-Noire, Ewo, Gamboma, Oyo, Owando	Mar 2011 - Jul 2011	Other	Blood Donors	Nurtop (2020)
PRNT	3.2 %						TRUE	Pointe-Noir	Mar 2011 - Jul 2011	Other	Blood Donors	Nurtop (2020)
**Rwanda**												
IgG	1.4 %			12	874	874		4 provinces and Kigali	2015 - 2015	Population	General Population	Seruyange (2018)
**Saudi Arabia**												
IgG				0	52	52		Najran city	Nov 2016 - Jul 2017	Hospital	Other	Alayed (2018)
IgG	1 %			2	217	217		Abha	2019 - 2020	Hospital	Pregnant Women	Harish (2023)
IgG				52	410	410		Najran city	Nov 2016 - Jul 2017	Hospital	Pregnant Women	Alayed (2018)
IgM				0	52	52		Najran city	Nov 2016 - Jul 2017	Hospital	Other	Alayed (2018)
IgM	1.8 %			4	217	217		Abha	2019 - 2020	Hospital	Pregnant Women	Harish (2023)
IgM				24	410	410		Najran city	Nov 2016 - Jul 2017	Hospital	Pregnant Women	Alayed (2018)
**Senegal**												
IgM				9	13845	13845		Kedougou	Jul 2009 - Mar 2013	Hospital	Persons Under Investigation	Sow (2016)
PRNT	13.4 %	95% CI: 9.65-17.9	Age, Sex					Niakhar	Aug 2007 - Oct 2007			Marchi (2020)
PRNT	13.7 %	95% CI: 9.4-19.14	Age, Sex					Niakhar	Nov 2011 - Apr 2012			Marchi (2020)
**Solomon Islands**												
IgG			Age	11	1021				Apr 2018 - Nov 2018	Community	General Population	Russell (2022)
**Spain**												
IgG	52.7 %			214	406	406		Barcelona	Jan 2016 - Apr 2019	Population	Pregnant Women	Martinez-Arias (2023)
IgM	0.5 %			2	406	406		Barcelona	Jan 2016 - Apr 2019	Population	Pregnant Women	Martinez-Arias (2023)
IgM	7.8 %			47	602	602		Madrid	Jan 2016 - Jan 2017	Hospital	Persons Under Investigation	Crespillo-Andujar (2020)
PRNT	4.8 %			10	209	209	TRUE	Barcelona	Jan 2016 - Apr 2019	Population	Pregnant Women	Martinez-Arias (2023)
PRNT	84 %			126	150	150		Hospital Universitari Vall d'Hebron Barcelona, Catalonia	May 2016 - Dec 2021	Mixed	Pregnant Women	Romaní (2022)

continued on next page

continued from previous page

Seroprev- alence type	Parameter value	Uncert- ainty	Disag- gregation	Num- erator	Denom- inator	Sample size	PRNT on ELISA + tests	Location	Dates	Population sample type	Population group	Reference
**Sri Lanka**												
IgG	34.5 %			102	295			Kandy	2017 - 2019	Hospital	Persons Under Investigation	Tun (2023)
IgG	42.6 %			128	300			Negombo	2018 - 2019	Hospital	Persons Under Investigation	Tun (2023)
IgM	1.4 %			4	295			Kandy	2017 - 2019	Hospital	Persons Under Investigation	Tun (2023)
IgM	9 %			17	300			Negombo	2018 - 2019	Hospital	Persons Under Investigation	Tun (2023)
**Sudan**												
IgG	62.7 %	95% CI: 59.4-66.1	Age, Region, Sex	530	845	845			2012 - 2012	Community	Other	Soghaier (2018)
PRNT				1	530	530	TRUE		2012 - 2012	Community	Other	Soghaier (2018)
PRNT	3.2 %			3	92	92			Dec 2012 - Jan 2013	Population	Other	Adam (2024)
PRNT	21.7 %			23	106	106			Dec 2012 - Jan 2013	Population	Other	Adam (2024)
**Suriname**												
IgG	49.7 %	95% CI: 41.9-57.5	Age, Other, Sex, Symptoms	77	155	155		Kwamalasamutu	Jan 2017 - Feb 2017	Household	General Population	Langerak (2019)
IgG	68.4 %	95% CI: 63.8-72.6	Age, Other, Sex, Symptoms	290	424	424		Paramaribo	Jan 2017 - Feb 2017	Hospital	Persons Under Investigation	Langerak (2019)
IgG	83.2 %	95% CI: 77.3-87.9	Age, Other, Sex, Symptoms	159	191	191		Laduani	Jan 2017 - Feb 2017	Hospital	Persons Under Investigation	Langerak (2019)
PRNT	24.5 %	95% CI: 18.4-31.9	Age, Other, Sex, Symptoms	38	155	155		Kwamalasamutu	Jan 2017 - Feb 2017	Household	General Population	Langerak (2019)
PRNT	36.7 %	95% CI: 30.1-43.7	Age, Other, Sex, Symptoms	70	191	191		Laduani	Jan 2017 - Feb 2017	Hospital	Persons Under Investigation	Langerak (2019)
PRNT	38.2 %	95% CI: 33.7-42.9	Age, Other, Sex, Symptoms	162	424	424		Paramaribo	Jan 2017 - Feb 2017	Hospital	Persons Under Investigation	Langerak (2019)
**Sweden**												
IgG	0 %			0	215	215		Gothenburg	2015 - 2015	Population	General Population	Seruyange (2018)
**Taiwan**												
IgG	4.2 %			9	212	212		Tainan City	2015 - 2015	Travel	General Population	Chien (2019)
IgG	38.8 %		Age, Other, Sex		600	600			Jun 2017 - Aug 2017	Travel	Other	Perng (2019)
IgM	0 %			0	212	212		Tainan City	2015 - 2015	Travel	General Population	Chien (2019)
IgM	3 %		Age, Other, Sex	18	600	600			Jun 2017 - Aug 2017	Travel	Other	Perng (2019)
PRNT	11.11 %			1	9	212	TRUE	Tainan City	2015 - 2015	Travel	General Population	Chien (2019)
**Tanzania**												
IgG	2.5 %							Kondoa	Apr 2018 - Nov 2018	Population	Mixed Groups	Mwanyika (2021)
IgG	3.9 %							Buhigwe	Apr 2018 - Nov 2018	Population	Mixed Groups	Mwanyika (2021)
IgG	5 %							Kinondoni	Apr 2018 - Nov 2018	Population	Mixed Groups	Mwanyika (2021)
IgG	5.8 %							Kilindi	Apr 2018 - Nov 2018	Population	Mixed Groups	Mwanyika (2021)
IgG	5.9 %			59	989	1818		Buhigwe, Kalambo, Kilindi, Kinondoni, Kondoa, Kyela, Mvomero and Ukerewe districts	Apr 2018 - Nov 2018	Population	Mixed Groups	Mwanyika (2021)

continued on next page

continued from previous page

Seroprev- alence type	Parameter value	Uncert- ainty	Disag- gregation	Num- erator	Denom- inator	Sample size	PRNT on ELISA + tests	Location	Dates	Population sample type	Population group	Reference
IgG	6.8 %				1818	1818		Buhigwe, Kalambo, Kilindi, Kinondoni, Kondoa, Kyela, Mvomero and Ukerewe districts	Apr 2018 - Nov 2018	Population	Mixed Groups	Mwanyika (2021)
IgG	6.1 - 7.6 %		Age		1818	1818		Buhigwe, Kalambo, Kilindi, Kinondoni, Kondoa, Kyela, Mvomero and Ukerewe districts	Apr 2018 - Nov 2018	Population	Mixed Groups	Mwanyika (2021)
IgG	7.5 %			62	829	1818		Buhigwe, Kalambo, Kilindi, Kinondoni, Kondoa, Kyela, Mvomero and Ukerewe districts	Apr 2018 - Nov 2018	Population	Mixed Groups	Mwanyika (2021)
IgG	7.7 %							Kalambo	Apr 2018 - Nov 2018	Population	Mixed Groups	Mwanyika (2021)
IgG	8.7 %							Kyela	Apr 2018 - Nov 2018	Population	Mixed Groups	Mwanyika (2021)
IgG	10.6 %							Mvomero	Apr 2018 - Nov 2018	Population	Mixed Groups	Mwanyika (2021)
IgG	10.6 %							Ukerewe	Apr 2018 - Nov 2018	Population	Mixed Groups	Mwanyika (2021)
**Thailand**												
IgG	11 %			8	73	73		Samut Songkhram	Apr 2017 - Apr 2018	Population	General Population	Sirinam (2022)
IgG	13.4 %			13	97	97		Samut Songkhram	Apr 2017 - Apr 2018	Population	General Population	Sirinam (2022)
IgG	15.1 %		Age, Time	53	350	350		Samut Songkhram	Apr 2017 - Apr 2018	Population	General Population	Sirinam (2022)
IgG	17.8 %			32	180	180		Samut Songkhram	Apr 2017 - Apr 2018	Population	General Population	Sirinam (2022)
IgG	16.5 - 27.4 %	95% CI: 13-33.4	Other, Sex, Time		1648	1648			Dec 1997 - Dec 2017	Population	General Population	Harapan (2022)
IgG	29 %			50	174	174			1997 - 2015	Other	Pregnant Women	Ngo-Giang-Huong (2021)
IgG	30.4 %			290	955	955		Ratchaburi, Bangkok	2006 - 2009	School	Children	Sriburin (2021)
IgG	30.77 %		Age, Other, Time	200	650	650		Siriraj Hospital	May 2019 - Oct 2019	Hospital	Pregnant Women	Phatihattakorn (2021)
IgG	83.6 %		Age, Sex	51	61	61		Nakhon Ratchasima	Apr 2016 - Jun 2016	Population	General Population	Hattakam (2021)
IgM	0 %			0	174	174			1997 - 2015	Other	Pregnant Women	Ngo-Giang-Huong (2021)
PRNT	2.8 %				254	254			Dec 2017 - Feb 2020	Population	General Population	Kitro (2024)
PRNT	8.14 %			21	258	258	TRUE	Siriraj Hospital	May 2019 - Oct 2019	Hospital	Pregnant Women	Phatihattakorn (2021)
PRNT	12.8 %	95% CI: 9.7-16.5						Narathiwat	Jul 2018 - Mar 2019	Household	Pregnant Women	Densathaporn (2020)
PRNT	17 %				147	147			2011 - 2012	Population	General Population	Yamanaka (2021)
PRNT	21.6 %				51	51		Phuket	2011 - 2012	Population	General Population	Yamanaka (2021)
PRNT	23.5 %				34	34		Nakhon Sawan	2011 - 2012	Population	General Population	Yamanaka (2021)

continued on next page

continued from previous page

Seroprev- alence type	Parameter value	Uncert- ainty	Disag- gregation	Num- erator	Denom- inator	Sample size	PRNT on ELISA + tests	Location	Dates	Population sample type	Population group	Reference
PRNT	24.3 %	95% CI: 20.1-28.8						Surat Thani	Jul 2018 - Mar 2019	Household	Pregnant Women	Densathaporn (2020)
PRNT	29.7 %	95% CI: 23.3-26		385	499	499		Narathiwat	Mar 2018 - Sep 2018	Household	Mixed Groups	Densathaporn (2020)
PRNT	43.7 %	95% CI: 35.9-51.6		377	504	504		Surat Thani	Mar 2018 - Sep 2018	Household	Mixed Groups	Densathaporn (2020)
PRNT	44.3 %		Age, Sex	27	61	61		Nakhon Ratchasima	Apr 2016 - Jun 2016	Population	General Population	Hattakam (2021)
Western blot				2	21	21			Unspecified	Hospital	Persons Under Investigation	Wikan (2016)
**The Gambia**												
PRNT	3.7 %	95% CI: 1.87-6.55	Age, Sex					Basse Santa Su	Aug 2007 - Oct 2007			Marchi (2020)
PRNT	7.1 %	95% CI: 4.03-11.45	Age, Sex					Basse Santa Su	Nov 2011 - Apr 2012			Marchi (2020)
**Uganda**												
HAI/HI	6 %		Region	8	132	132		Karamoja District	May 1984 - May 1984	Community	General Population	Rodhain (1989)
IgM				5	384	384		Nkonkonjeru	Feb 2014 - Oct 2017	Hospital	Other	Kayiwa (2018)
PRNT				3	5	384	TRUE	Nkonkonjeru	Feb 2014 - Oct 2017	Hospital	Other	Kayiwa (2018)
**United Kingdom**												
IgM	19 %			31	161	161			01 Jan 2016 - 31 Dec 2017	Travel	Persons Under Investigation	Petridou (2019)
**United States**												
IgG				4	18	18		Hawaii	2009 - 2012	Other	Persons Under Investigation	Kumar (2016)
IgG	31.4 %	95% CI: 30-32.9	Age, Occupation, Other, Sex	1268	4035	4090		Ponce, Puerto Rico	Apr 2018 - May 2019	Household	General Population	Adams (2022)
IgM	2.18 %			277	12374	12374		Hidalgo County, Texas	2016 - 2018	Hospital	Mixed Groups	Hinojosa (2020)
IgM	5 %	95% CI: 2.5-10		8	141	141			Jan 2016 - Aug 2016	Travel	Pregnant Women	Rao (2017)
IgM				1	18	18		Hawaii	2009 - 2012	Other	Persons Under Investigation	Kumar (2016)
IgM	7 %			22	295	295		New York	01 Jan 2016 - 30 Jun 2017	Hospital	Persons Under Investigation	Conners (2018)
IgM	7 %			36	547	547		Dallas, Texas	14 Mar 2016 - 01 Oct 2016	Travel	Pregnant Women	Adhikari (2017)
IgM	12.5 %				713	713		Ponce, Puerto Rico	2018 - 2019	Community	Children	Adams (2023)
IgM	13 %			16	141	141			2016 - 2017	Hospital	Children	Lindsey (2020)
IgM	16 %		Other	5	31	31		Congenital Zika Program at Children's National (CZPCN) in Washington DC	Jan 2016 - Jun 2018	Hospital	Children	Mulkey (2021)
IgM				79	367	367		San Juan, Puerto Rico	16 Sep 2016 - 27 Oct 2016	Contact	Other	Lozier (2018)
IgM	22 %			22	329	329		New York City	Jan 2016 - Dec 2017	Hospital	Persons Under Investigation	Lee (2020)
IgM	30.9 %	95% CI: 26.3-35.9		112	362	362		Puerto Rico: Ponce, San Juan, Guayama	May 2016 - Jul 2017	Household	Household Contacts Of Survivors	Rosenberg (2019)

continued on next page

continued from previous page

Seroprev- alence type	Parameter value	Uncert- ainty	Disag- gregation	Num- erator	Denom- inator	Sample size	PRNT on ELISA + tests	Location	Dates	Population sample type	Population group	Reference
IgM				110	339	339		Puerto Rico	Apr 2016 - Dec 2016	Population	Persons Under Investigation	Williamson (2020)
IgM	37 %	95% CI: 29-44		56	153	153			Unspecified	Population	Persons Under Investigation	Hills (2021)
IgM	44 %		Other	27	61	61		Congenital Zika Program at Children's National (CZPCN) in Washington DC	Jan 2016 - Jun 2018	Hospital	Pregnant Women	Mulkey (2021)
IgM	63 %			19	30	30		Miami-Dade County, Florida	Unspecified	Other	Persons Under Investigation	Griffin (2019)
NS1 BOB ELISA	1 %				91	91		Puerto Rico	2013 - 2014	Population	General Population	Nascimento (2019)
NS1 BOB ELISA	20 %				91	91		Puerto Rico	2017 - 2018	Population	General Population	Nascimento (2019)
PRNT	0 %			0	5	5	TRUE	Congenital Zika Program at Children's National (CZPCN) in Washington DC	Jan 2016 - Jun 2018	Hospital	Children	Mulkey (2021)
PRNT	0.2 %	95% CI: 0.01-1.1			494	494		Puerto Rico	01 Jan 2015 - 01 Jun 2015	Other	Other	Pollett (2022)
PRNT				1	141	141			Jan 2016 - Aug 2016	Travel	Pregnant Women	Rao (2017)
PRNT	1 %				91	91	TRUE	Puerto Rico	2013 - 2014	Population	General Population	Nascimento (2019)
PRNT				16	969	183		Veterans Health Administration in US and Caribbean	01 Dec 2015 - 31 Oct 2016	Hospital	Other	Schirmer (2018)
PRNT	5 %				91	91	TRUE	Puerto Rico	2017 - 2018	Population	General Population	Nascimento (2019)
PRNT	5.3 %			29	36	547	TRUE	Dallas, Texas	14 Mar 2016 - 01 Oct 2016	Travel	Pregnant Women	Adhikari (2017)
PRNT	8 %			2	26	26	TRUE	Congenital Zika Program at Children's National (CZPCN) in Washington DC	Jan 2016 - Jun 2018	Hospital	Pregnant Women	Mulkey (2021)
PRNT	18.8 %			44	234	234	TRUE	Hidalgo County, Texas	2016 - 2018	Hospital	Mixed Groups	Hinojosa (2020)
PRNT	25 %			23	92	92		Puerto Rico	Jun 2011 - Mar 2012	Other	Children	Zambrano (2021)
PRNT	33.2 %			237	713	713		Ponce, Puerto Rico	2018 - 2019	Community	Children	Adams (2023)
**Vanuatu**												
IgG	51.8 %		Age, Sex	102	197	197		Espiritu Santo	Aug 2016 - Jan 2017	Population	General Population	Saretzki (2024)
**Vietnam**												
IgG	2.3 %				176	176		Ho Chi Minh City	Jan 2008 - Dec 2008	Hospital	Pregnant Women	Chui (2023)
IgG				1	21	21		Ho Chi Minh City	Mar 2016 - Nov 2017	Hospital	Children	Grant (2021)

continued on next page

continued from previous page

Seroprev- alence type	Parameter value	Uncert- ainty	Disag- gregation	Num- erator	Denom- inator	Sample size	PRNT on ELISA + tests	Location	Dates	Population sample type	Population group	Reference
IgG				18	20	20		Ho Chi Minh City	Mar 2016 - Nov 2017	Hospital	Pregnant Women	Grant (2021)
IgM	1 %			21	2013	2013		Nha Trang	jul 2017 - Sep 2018	Hospital	Children	Tun (2020b)
IgM	10.3 %		Age, Other	83	801	801		Krong Buk	Jan 2017 - Jul 2018	Community	Mixed Groups	Nguyen (2020)
PRNT	0.5 %			11	21	2013	TRUE	Nha Trang	Jul 2017 - Sep 2018	Hospital	Children	Tun (2020b)
PRNT	1.1 %		Age, Other	9	83	801	TRUE	Krong Buk	Jan 2017 - Jul 2018	Community	Mixed Groups	Nguyen (2020)
**Zambia**												
IgG	10.8	95% CI: 6.9-15.7	Age, Occupation, Other, Sex	23	214	214		Lukanga swamps, Central Province of Zambia	Oct 2016 - Nov 2016	Community	Other	Chisenga (2020)

Table B.10: Overview of extracted seroprevalence parameters.

B.7.5 Risk factors

Risk factor name	Statistically significant	Adjusted	Sample size	Location	Dates	Population sample type	Population group	Reference
**Infection**								
Age	Significant	Not Adjusted		Guadeloupe, Martinique (France)	06 Jan 2016	Hospital	Persons Under Investigation	Lannuzel (2019)
Age	Significant	Unspecified		Managua (Nicaragua)	2016 - 2016	Population	General Population	Counotte (2019)
Age, Other	Significant	Adjusted	362	Puerto Rico: Ponce, San Juan, Guayama (United States)	May 2016 - Jul 2017	Household	Household Contacts Of Survivors	Rosenberg (2019)
Age, Other	Significant	Adjusted	805	Narathiwat and Surat Thani (Thailand)	Mar 2018 - Mar 2019	Household	Mixed Groups	Densathaporn (2020)
Age, Other	Significant	Not Adjusted	1717	Thailand	Jan 2016 - Dec 2017	Population	General Population	Ruchusatsawat (2019)
Age, Other, Sex	Significant	Adjusted	4090	Ponce, Puerto Rico (United States)	Apr 2018 - May 2019	Household	General Population	Adams (2022)
Age, Other, Sex	Significant	Not Adjusted	467	Portoviejo, Manabi (Ecuador)	01 Jan 2016 - 31 Aug 2016	Hospital	General Population	Fors (2018)
Age, Other, Sex	Significant	Unspecified	106033	Colombia	Aug 2015 - Jun 2017	Population	Persons Under Investigation	Charniga (2021a)
Age, Prior immunity to arboviruses, Sex	Significant	Not Adjusted	3296	Managua (Nicaragua)	01 Jan 2016 - 28 Feb 2017	Community	Children	Gordon (2019)
Age, Sex	Significant	Adjusted	1936	Girardot (Colombia)	19 Oct 2015 - 22 Jan 2016	Hospital	Persons Under Investigation	Rojas (2016)
Age, Sex	Significant	Adjusted	928	San Andrés (Colombia)	06 Sep 2015 - 30 Jan 2016	Hospital	Persons Under Investigation	Rojas (2016)
Age, Sex	Significant	Adjusted	3296	Managua (Nicaragua)	01 Jan 2016 - 28 Feb 2017	Community	Children	Gordon (2019)
Infection during pregnancy, Prior immunity to arboviruses	Significant	Adjusted	225	León (Nicaragua)	Jan 2016 - Aug 2017	Hospital	Persons Under Investigation	Bowman (2021)
Other	Significant			Fiji	Sep 2013 - Nov 2015	Population	Persons Under Investigation	Kama (2019)
Other	Significant	Adjusted	367	San Juan, Puerto Rico (United States)	16 Sep 2016 - 27 Oct 2016	Contact	Other	Lozier (2018)
Other	Significant	Adjusted		Multi-country: Americas (n = 19), Europe (n = 8), Asia (n = 1), Oceania (n = 4)	2015 - 2016	Unspecified	Unspecified	Nah (2016)
Other	Significant	Adjusted	142	Managua (Nicaragua)	31 Aug 2016 - 21 Oct 2016	Household	Household Contacts Of Survivors	Burger-Calderon (2018)
Other	Significant	Adjusted		Colombia	Sep 2015 - Jul 2016	Population	General Population	Kellemen (2021)

continued on next page

continued from previous page

Risk factor name	Statistically significant	Adjusted	Sample size	Location	Dates	Population sample type	Population group	Reference
Other	Significant	Adjusted		Rio de Janeiro (Brazil)	2015 - 2016	Population	General Population	Queiroz (2021)
Other	Significant	Adjusted		Unspecified	2016			Weinstein (2020)
Other	Significant	Adjusted	602	Madrid (Spain)	Jan 2016 - Jan 2017	Hospital	Persons Under Investigation	Crespillo-Andujar (2020)
Other	Significant	Adjusted	7029	Barranquilla (Colombia)	2014 - 2016	Population	General Population	Mchale (2019)
Other	Significant	Adjusted	12823	Mexico	Jan 2012 - Dec 2020	Population	Persons Under Investigation	Bukhari (2023)
Other	Significant	Adjusted	3493	Cayenne, Kourou, Saint Laurent (Guyana)	Jan 2016 - Dec 2016	Hospital	Pregnant Women	Hallet (2020)
Other	Significant	Adjusted	739	St. Croix, St. John, St. Thomas (Virgin Islands (U.S.))	03 Jan 2016 - 24 Jan 2018	Population	Persons Under Investigation	Browne (2022)
Other	Significant	Adjusted	1031	St. Thomas (Virgin Islands (U.S.))	03 Jan 2016 - 24 Jan 2018	Population	Persons Under Investigation	Browne (2022)
Other	Significant	Adjusted	1031	St. Croix (Virgin Islands (U.S.))	03 Jan 2016 - 24 Jan 2018	Population	Persons Under Investigation	Browne (2022)
Other	Significant	Adjusted	1031	St. John (Virgin Islands (U.S.))	03 Jan 2016 - 24 Jan 2018	Population	Persons Under Investigation	Browne (2022)
Other	Significant	Adjusted	865	Unspecified	Jan 2016 - Jul 2019	Other	Pregnant Women	Vouga (2021)
Other	Significant	Not Adjusted	362	Puerto Rico: Ponce, San Juan, Guayama (United States)	May 2016 - Jul 2017	Household	Household Contacts Of Survivors	Rosenberg (2019)
Other	Significant	Not Adjusted	367	San Juan, Puerto Rico (United States)	16 Sep 2016 - 27 Oct 2016	Contact	Other	Lozier (2018)
Other	Significant	Not Adjusted	4533	Multi-country: Americas (n = 21)	15 Aug 2015 - 11 Jun 2016	Population	Persons Under Investigation	Ogden (2017)
Other	Significant	Not Adjusted		Manaus (Brazil)	Unspecified	Population	General Population	Giovanetti (2020)
Other	Significant	Not Adjusted	382	Goiania (Brazil)	01 Jan 2016 - 31 Dec 2017	Population	Pregnant Women	Rosado (2022)
Other	Significant	Not Adjusted	144	Recife (Brazil)	Dec 2015 - Apr 2017	Community	Pregnant Women	Lobkowicz (2021)
Other	Significant	Not Adjusted	3493	Cayenne, Kourou, Saint Laurent (Guyana)	Jan 2016 - Dec 2016	Hospital	Pregnant Women	Hallet (2020)
Other	Significant	Not Adjusted	189	Dominican Republic	2016	Travel	Other	Voss (2020)

continued on next page

continued from previous page

Risk factor name	Statistically significant	Adjusted	Sample size	Location	Dates	Population sample type	Population group	Reference
Other	Significant	Not Adjusted	751	Bangkok, Samut Prakan, Samut Sakhon, Ratchaburi, Chon Buri (Thailand)	Mar 2020 - Mar 2023	Hospital	Persons Under Investigation	Khongwichit (2023)
Other	Significant	Unspecified		Rio de Janeiro (Brazil)	Feb 2015 - May 2016	Population	Persons Under Investigation	Fuller (2017)
Other	Significant	Unspecified	270	Miami-Dade County, Florida (United States)	Jul 2016 - Dec 2016	Community	General Population	Ajelli (2017)
Other	Significant	Unspecified	1289	Fortaleza (Brazil)	Feb 2018 - Dec 2018	Community	Other	Frota (2023)
Other, Sex	Significant	Unspecified	21468	Puerto Rico (United States)	03 Apr 2016 - 12 Aug 2016	Community	Other	Chevalier (2017)
Age	Not Significant	Not Adjusted	3493	Cayenne, Kourou, Saint Laurent (Guyana)	Jan 2016 - Dec 2016	Hospital	Pregnant Women	Hallet (2020)
Age	Not Significant	Unspecified	21468	Puerto Rico (United States)	03 Apr 2016 - 12 Aug 2016	Community	Other	Chevalier (2017)
Age, Comorbidity, Other, Sex	Not Significant	Adjusted	367	San Juan, Puerto Rico (United States)	16 Sep 2016 - 27 Oct 2016	Contact	Other	Lozier (2018)
Age, Comorbidity, Other, Sex	Not Significant	Not Adjusted	367	San Juan, Puerto Rico (United States)	16 Sep 2016 - 27 Oct 2016	Contact	Other	Lozier (2018)
Age, Household contact, Other, Sex	Not Significant	Adjusted	142	Managua (Nicaragua)	31 Aug 2016 - 21 Oct 2016	Household	Household Contacts Of Survivors	Burger-Calderon (2018)
Age, Occupation, Other	Not Significant	Adjusted		Buhigwe (Tanzania)	Apr 2018 - Nov 2018	Population	Mixed Groups	Mwanyika (2021)
Age, Occupation, Other	Not Significant	Not Adjusted		Buhigwe (Tanzania)	Apr 2018 - Nov 2018	Population	Mixed Groups	Mwanyika (2021)
Age, Occupation, Other, Sex	Not Significant	Not Adjusted	362	Puerto Rico: Ponce, San Juan, Guayama (United States)	May 2016 - Jul 2017	Household	Household Contacts Of Survivors	Rosenberg (2019)
Age, Other	Not Significant	Adjusted	3493	Cayenne, Kourou, Saint Laurent (Guyana)	Jan 2016 - Dec 2016	Hospital	Pregnant Women	Hallet (2020)
Age, Other	Not Significant	Not Adjusted	547	Dallas, Texas (United States)	14 Mar 2016 - 01 Oct 2016	Travel	Pregnant Women	Adhikari (2017)
Age, Other	Not Significant	Not Adjusted	135	Diamantina (Brazil)	2018 - 2019	Population	Pregnant Women	Santos (2023)
Age, Other, Sex	Not Significant	Adjusted	225	León (Nicaragua)	Jan 2016 - Aug 2017	Hospital	Persons Under Investigation	Bowman (2021)

continued on next page

continued from previous page

Risk factor name	Statistically significant	Adjusted	Sample size	Location	Dates	Population sample type	Population group	Reference
Age, Other, Sex	Not Significant	Unspecified	751	Bangkok, Samut Prakan, Samut Sakhon, Ratchaburi, Chon Buri (Thailand)	Mar 2020 - Mar 2023	Hospital	Persons Under Investigation	Khongwichit (2023)
Age, Prior immunity to arboviruses	Not Significant	Adjusted	2749	Rio de Janeiro (Brazil)	Jul 2018 - Oct 2018	Population	General Population	Perisse (2020)
Age, Sex	Not Significant	Unspecified	106033	Colombia	Aug 2015 - Jun 2017	Population	Persons Under Investigation	Charniga (2021a)
Household contact, Other	Not Significant	Adjusted	805	Narathiwat and Surat Thani (Thailand)	Mar 2018 - Mar 2019	Household	Mixed Groups	Densathaporn (2020)
Other	Not Significant			Fiji	Sep 2013 - Nov 2015	Population	Persons Under Investigation	Kama (2019)
Other	Not Significant	Adjusted	362	Puerto Rico: Ponce, San Juan, Guayama (United States)	May 2016 - Jul 2017	Household	Household Contacts Of Survivors	Rosenberg (2019)
Other	Not Significant	Adjusted		Multi-country: Americas (n = 19), Europe (n = 8), Asia (n = 1), Oceania (n = 4)	2015 - 2016	Unspecified	Unspecified	Nah (2016)
Other	Not Significant	Adjusted		Colombia	Sep 2015 - Jul 2016	Population	General Population	Kellemen (2021)
Other	Not Significant	Adjusted		Rio de Janeiro (Brazil)	2015 - 2016	Population	General Population	Queiroz (2021)
Other	Not Significant	Adjusted		Centre Hospitalier de l'Ouest Guyanais (CHOG, Saint-Laurent-du-Maroni, in French Guiana) (France)	Jan 2015 - Jul 2016	Hospital	Pregnant Women	Pomar (2021)
Other	Not Significant	Adjusted	12823	Mexico	Jan 2012 - Dec 2020	Population	Persons Under Investigation	Bukhari (2023)
Other	Not Significant	Adjusted	4090	Ponce, Puerto Rico (United States)	Apr 2018 - May 2019	Household	General Population	Adams (2022)
Other	Not Significant	Adjusted	1031	St. Thomas (Virgin Islands (U.S.))	03 Jan 2016 - 24 Jan 2018	Population	Persons Under Investigation	Browne (2022)
Other	Not Significant	Adjusted	1031	St. Croix (Virgin Islands (U.S.))	03 Jan 2016 - 24 Jan 2018	Population	Persons Under Investigation	Browne (2022)
Other	Not Significant	Adjusted	1031	St. John (Virgin Islands (U.S.))	03 Jan 2016 - 24 Jan 2018	Population	Persons Under Investigation	Browne (2022)

continued on next page

continued from previous page

Risk factor name	Statistically significant	Adjusted	Sample size	Location	Dates	Population sample type	Population group	Reference
Other	Not Significant	Not Adjusted	2659	Managua (Nicaragua)	01 Jan 2016 - 28 Feb 2017	Community	Children	Gordon (2019)
Other	Not Significant	Not Adjusted		Centre Hospitalier de l'Ouest Guyanais (CHOG, Saint-Laurent-du-Maroni, in French Guiana) (France)	Jan 2015 - Jul 2016	Hospital	Pregnant Women	Pomar (2021)
Other	Not Significant	Not Adjusted	189	Dominican Republic	2016	Travel	Other	Voss (2020)
Other	Not Significant	Not Adjusted		Guadeloupe, Martinique (France)	06 Jan 2016	Hospital	Persons Under Investigation	Lannuzel (2019)
Other, Prior immunity to arboviruses	Not Significant	Adjusted	2659	Managua (Nicaragua)	01 Jan 2016 - 28 Feb 2017	Community	Children	Gordon (2019)
Other, Sex	Not Significant	Adjusted	7029	Barranquilla (Colombia)	2014 - 2016	Population	General Population	Mchale (2019)
Other, Sex	Not Significant	Not Adjusted	1717	Thailand	Jan 2016 - Dec 2017	Population	General Population	Ruchusatsawat (2019)
Other	Unspecified	Unspecified	10595	Australia	Jul 2013 - Jun 2014	Other	Blood Donors	Coghlan (2018)
Sex	Unspecified	Unspecified		United States	2016 - 2017	Mixed	Mixed Groups	Major (2021)
**Serology**								
Age	Significant	Adjusted	845	Sudan	2012 - 2012	Community	Other	Soghaier (2018)
Age	Significant	Not Adjusted		District II, Managua (Nicaragua)	Feb 2017 - Jul 2017	Household	Other	Zambrana (2018)
Age	Significant	Not Adjusted	1430	Mali	2013 - Nov 2016	Population	General Population	Diarra (2020)
Age	Significant	Unspecified	801	Krong Buk (Vietnam)	Jan 2017 - Jul 2018	Community	Mixed Groups	Nguyen (2020)
Age, Comorbidity, Other	Significant	Not Adjusted	871	Abia State University Teaching Hospital, Aba, Abia state, Federal Medical Centre, Keffi, Nasarawa State, Baru-Dokp Teaching Hospital, Kaduna, Kaduna State (Nigeria)	Dec 2020 - Nov 2021	Hospital	Mixed Groups	Mac (2023a)
Age, Other	Significant	Adjusted	1295	Thailand	Dec 1997 - Dec 2017	Population	Pregnant Women	Harapan (2022)
Age, Other	Significant	Adjusted	469	Salvador (Hospital Geral Roberto Santos)	Oct 2015	Population	Pregnant Women	Nery (2021)
Age, Other	Significant	Adjusted		Coastal lowlands of SW Guatemala (Guatemala)	2015 - 2016	Population	Children	Lamb (2022)

continued on next page

continued from previous page

Risk factor name	Statistically significant	Adjusted	Sample size	Location	Dates	Population sample type	Population group	Reference
Age, Other	Significant	Adjusted	1192	Borbón, Maldonado, Timbiré, Santa Maria, Santo Domingo, Colon Eloy (Ecuador)	Jul 2018 - Oct 2018	Community	General Population	Andrade (2024)
Age, Other	Significant	Not Adjusted	501	Ouagadougou, Bobo-Dioulasso (Burkina Faso)	2020 - 2020	Population	Blood Donors	Tinto (2022)
Age, Other	Significant	Not Adjusted	1295	Thailand	Dec 1997 - Dec 2017	Population	Pregnant Women	Harapan (2022)
Age, Other	Significant	Not Adjusted	469	Salvador (Hospital Geral Roberto Santos)	Oct 2015	Population	Pregnant Women	Nery (2021)
Age, Other	Significant	Not Adjusted	455	Unspecified	Mar 2014 - Oct 2017	Travel	General Population	Overbosch (2023)
Age, Other	Significant	Not Adjusted	585	"Peninsula" (Malaysia)	Unspecified	Population	General Population	Khor (2024)
Age, Other	Significant	Not Adjusted	1192	Borbón, Maldonado, Timbiré, Santa Maria, Santo Domingo, Colon Eloy (Ecuador)	Jul 2018 - Oct 2018	Community	General Population	Andrade (2024)
Age, Other	Significant	Unspecified		Malaysia	Unspecified	Community	General Population	Khor (2020)
Age, Other, Prior immunity to arboviruses	Significant	Adjusted		Recife (Brazil)	Aug 2018 - Feb 2019	Household	General Population	Braga (2023)
Age, Other, Prior immunity to arboviruses	Significant	Adjusted	1126	Borbón, Maldonado, Timbiré, Santa Maria, Santo Domingo, Colon Eloy (Ecuador)	Aug 2019 - Oct 2019	Community	General Population	Andrade (2024)
Age, Other, Prior immunity to arboviruses	Significant	Not Adjusted		Recife (Brazil)	Aug 2018 - Feb 2019	Household	General Population	Braga (2023)
Age, Other, Sex	Significant	Not Adjusted		District II, Managua (Nicaragua)	Feb 2017 - Jul 2017	Household	Children	Zambrana (2018)
Age, Sex	Significant	Adjusted		District II, Managua (Nicaragua)	Feb 2017 - Jul 2017	Household	Other	Zambrana (2018)
Age, Sex	Significant	Adjusted	1085	Kuala Lumpur (Malaysia)	2012 - 2017	Mixed	Mixed Groups	Sam (2019)
Household contact, Other	Significant	Adjusted	425	Pernambuco State (Brazil)	Apr 2017 - Dec 2017	Household	Mixed Groups	Magalhaes (2021)
Household contact, Other	Significant	Not Adjusted	425	Pernambuco State (Brazil)	Apr 2017 - Dec 2017	Household	Mixed Groups	Magalhaes (2021)
Infection	Significant	Unspecified	328	Santander (Colombia)	Unspecified	School	Mixed Groups	Cardenas (2020)
Occupation, Other	Significant	Adjusted	1084	Maroua, Garoua, Ngaoundere, Douala, Yaounde, Bertoua (Cameroon)	Aug 2015 - Oct 2015	Other	Blood Donors	Gake (2017)

continued on next page

continued from previous page

Risk factor name	Statistically significant	Adjusted	Sample size	Location	Dates	Population sample type	Population group	Reference
Occupation, Other, Sex	Significant	Adjusted	150	Itang special district, Lare district (Ethiopia)	Oct 2018 - Jun 2019	Community	General Population	Asebe (2021)
Other	Significant	Adjusted	100	Cross River State (Nigeria)	17 Mar 2017 - 30 May 2017	Population	Persons Under Investigation	Otu (2020)
Other	Significant	Adjusted	359	Salvador, Bahia (Brazil)	Aug 2015 - Jul 2016	Population	Persons Under Investigation	Ticona (2021)
Other	Significant	Adjusted	94	Maranhao state (Brazil)	2016 - 2018	Hospital	Persons Under Investigation	Ribeiro (2020)
Other	Significant	Adjusted	33	West Pokot (Kenya)	Feb 2016 - Feb 2016	Population	General Population	Chepkorir (2019)
Other	Significant	Adjusted	2697	French Guiana (France)	Jun 2017 - Oct 2017	Population	General Population	Bailly (2021)
Other	Significant	Adjusted	496	Adamawa, Bauchi, and Borno states (Nigeria)	Apr 2018 - Aug 2018	Population	Persons Under Investigation	Baba (2023)
Other	Significant	Adjusted	871	Abia, Kaduna, Nasarawa (Nigeria)	Dec 2020 - Nov 2021	Hospital	Mixed Groups	Peter Asaga (2023)
Other	Significant	Not Adjusted	642	Salvador, Bahia (Brazil)	Oct 2015 - Oct 2015	Population	Other	Rodriguez-Barraquer (2019)
Other	Significant	Not Adjusted	410	Saudi Arabia	Nov 2016 - Jul 2017	Hospital	Pregnant Women	Alayed (2018)
Other	Significant	Not Adjusted	2697	22 municipalities of French Guiana (France)	Jun 2017 - Oct 2017	Population	General Population	Flamand (2019)
Other	Significant	Not Adjusted	359	Salvador, Bahia (Brazil)	Aug 2015 - Jul 2016	Population	Persons Under Investigation	Ticona (2021)
Other	Significant	Not Adjusted	469	Salvador (Hospital Geral Roberto Santos)	Oct 2015	Population	Pregnant Women	Nery (2021)
Other	Significant	Not Adjusted		Salvador, Bahia (Brazil)	01 Jan 2015 - 31 Jan 2017	Hospital	Pregnant Women	Aromolaran (2022)
Other	Significant	Not Adjusted	496	Adamawa, Bauchi, and Borno states (Nigeria)	Apr 2018 - Aug 2018	Population	Persons Under Investigation	Baba (2023)
Other	Significant	Unspecified	1289	Fortaleza (Brazil)	Feb 2018 - Dec 2018	Community	Other	Frota (2023)
Other, Prior immunity to arboviruses	Significant	Not Adjusted	431	Santiago Island: Praia (Cabo Verde)	24 Aug 2014 - 04 Nov 2014	Population	General Population	Ward (2022)
Other, Sex	Significant	Adjusted		District II, Managua (Nicaragua)	Feb 2017 - Jul 2017	Household	Children	Zambrana (2018)
Other, Sex	Significant	Not Adjusted	468	Plateau, Nasarawa (Nigeria)	Jan 2016 - Dec 2016	Community	General Population	Mathe (2018)
Other, Sex	Significant	Unspecified	557	Yap Island (Federated States of Micronesia)	01 Apr 2007 - 31 Jul 2007	Household	General Population	Duffy (2009)
Prior immunity to arboviruses	Significant	Adjusted	642	Salvador, Bahia (Brazil)	Oct 2015 - Oct 2015	Population	Other	Rodriguez-Barraquer (2019)

continued on next page

continued from previous page

Risk factor name	Statistically significant	Adjusted	Sample size	Location	Dates	Population sample type	Population group	Reference
Prior immunity to arboviruses	Significant	Not Adjusted	642	Salvador, Bahia (Brazil)	Oct 2015 - Oct 2015	Population	Other	Rodriguez-Barraquer (2019)
Sex	Significant	Unspecified	153	United States	Unspecified	Population	Persons Under Investigation	Hills (2021)
Age	Not Significant	Adjusted	642	Salvador, Bahia (Brazil)	Oct 2015 - Oct 2015	Population	Other	Rodriguez-Barraquer (2019)
Age, Infection during pregnancy, Other, Sex	Not Significant	Adjusted	871	Abia, Kaduna, Nasarawa (Nigeria)	Dec 2020 - Nov 2021	Hospital	Mixed Groups	Peter Asaga (2023)
Age, Occupation, Other, Prior immunity to arboviruses, Sex	Not Significant	Adjusted	33	West Pokot (Kenya)	Feb 2016 - Feb 2016	Population	General Population	Chepkorir (2019)
Age, Other	Not Significant	Adjusted		District II, Managua (Nicaragua)	Feb 2017 - Jul 2017	Household	Children	Zambrana (2018)
Age, Other	Not Significant	Adjusted		District II, Managua (Nicaragua)	Feb 2017 - Jul 2017	Household	Other	Zambrana (2018)
Age, Other	Not Significant	Adjusted	1295	Thailand	Dec 1997 - Dec 2017	Population	Pregnant Women	Harapan (2022)
Age, Other	Not Significant	Adjusted		Recife (Brazil)	Aug 2018 - Feb 2019	Household	General Population	Braga (2023)
Age, Other	Not Significant	Adjusted	150	Itang special district, Lare district (Ethiopia)	Oct 2018 - Jun 2019	Community	General Population	Asebe (2021)
Age, Other	Not Significant	Adjusted	1192	Borbón, Maldonado, Timbiré, Santa Maria, Santo Domingo, Colon Eloy (Ecuador)	Jul 2018 - Oct 2018	Community	General Population	Andrade (2024)
Age, Other	Not Significant	Not Adjusted	1295	Thailand	Dec 1997 - Dec 2017	Population	Pregnant Women	Harapan (2022)
Age, Other	Not Significant	Unspecified	557	Yap Island (Federated States of Micronesia)	01 Apr 2007 - 31 Jul 2007	Household	General Population	Duffy (2009)
Age, Other	Not Significant	Unspecified	153	United States	Unspecified	Population	Persons Under Investigation	Hills (2021)
Age, Other	Not Significant	Unspecified	650	Siriraj Hospital (Thailand)	May 2019 - Oct 2019	Hospital	Pregnant Women	Phatihattakorn (2021)
Age, Other, Prior immunity to arboviruses, Sex	Not Significant	Not Adjusted		Recife (Brazil)	Aug 2018 - Feb 2019	Household	General Population	Braga (2023)
Age, Other, Sex	Not Significant	Adjusted	845	Sudan	2012 - 2012	Community	Other	Soghaier (2018)
Age, Other, Sex	Not Significant	Adjusted	100	Cross River State (Nigeria)	17 Mar 2017 - 30 May 2017	Population	Persons Under Investigation	Otu (2020)
Age, Other, Sex	Not Significant	Adjusted	2697	French Guiana (France)	Jun 2017 - Oct 2017	Population	General Population	Bailly (2021)
Age, Other, Sex	Not Significant	Adjusted	496	Adamawa, Bauchi, and Borno states (Nigeria)	Apr 2018 - Aug 2018	Population	Persons Under Investigation	Baba (2023)

continued on next page

continued from previous page

Risk factor name	Statistically significant	Adjusted	Sample size	Location	Dates	Population sample type	Population group	Reference
Age, Other, Sex	Not Significant	Adjusted	1126	Borbón, Maldonado, Timbiré, Santa Maria, Santo Domingo, Colon Eloy (Ecuador)	Aug 2019 - Oct 2019	Community	General Population	Andrade (2024)
Age, Other, Sex	Not Significant	Not Adjusted		District II, Managua (Nicaragua)	Feb 2017 - Jul 2017	Household	Other	Zambrana (2018)
Age, Other, Sex	Not Significant	Not Adjusted	642	Salvador, Bahia (Brazil)	Oct 2015 - Oct 2015	Population	Other	Rodriguez-Barraquer (2019)
Age, Other, Sex	Not Significant	Not Adjusted	2697	22 municipalities of French Guiana (France)	Jun 2017 - Oct 2017	Population	General Population	Flamand (2019)
Age, Other, Sex	Not Significant	Not Adjusted	431	Santiago Island: Praia (Cabo Verde)	24 Aug 2014 - 04 Nov 2014	Population	General Population	Ward (2022)
Age, Other, Sex	Not Significant	Not Adjusted	496	Adamawa, Bauchi, and Borno states (Nigeria)	Apr 2018 - Aug 2018	Population	Persons Under Investigation	Baba (2023)
Age, Other, Sex	Not Significant	Not Adjusted	1192	Borbón, Maldonado, Timbiré, Santa Maria, Santo Domingo, Colon Eloy (Ecuador)	Jul 2018 - Oct 2018	Community	General Population	Andrade (2024)
Age, Other, Sex	Not Significant	Unspecified	328	Santander (Colombia)	Unspecified	School	Mixed Groups	Cardenas (2020)
Age, Other, Sex	Not Significant	Unspecified	850	Mozambique	2009 - 2015	Population	Other	Chelene (2019)
Age, Prior immunity to arboviruses	Not Significant	Not Adjusted	642	Salvador, Bahia (Brazil)	Oct 2015 - Oct 2015	Population	Other	Rodriguez-Barraquer (2019)
Age, Sex	Not Significant	Unspecified	814	Beni, Santa Cruz, Tarija, Cochabamba, La Paz (Bolivia)	Dec 2016 - Apr 2017	Population	General Population	Villarroel (2018)
Comorbidity, Prior immunity to arboviruses, Sex	Not Significant	Not Adjusted	468	Plateau, Nasarawa (Nigeria)	Jan 2016 - Dec 2016	Community	General Population	Mathe (2018)
Occupation, Other	Not Significant	Not Adjusted	254	Thailand	Dec 2017 - Feb 2020	Population	General Population	Kitro (2024)
Other	Not Significant	Adjusted	1085	Kuala Lumpur (Malaysia)	2012 - 2017	Mixed	Mixed Groups	Sam (2019)
Other	Not Significant	Adjusted	359	Salvador, Bahia (Brazil)	Aug 2015 - Jul 2016	Population	Persons Under Investigation	Ticona (2021)
Other	Not Significant	Not Adjusted		District II, Managua (Nicaragua)	Feb 2017 - Jul 2017	Household	Children	Zambrana (2018)
Other	Not Significant	Not Adjusted	359	Salvador, Bahia (Brazil)	Aug 2015 - Jul 2016	Population	Persons Under Investigation	Ticona (2021)
Other	Not Significant	Not Adjusted	469	Salvador (Hospital Geral Roberto Santos)	Oct 2015	Population	Pregnant Women	Nery (2021)

continued on next page

continued from previous page

Risk factor name	Statistically significant	Adjusted	Sample size	Location	Dates	Population sample type	Population group	Reference
Other, Sex	Not Significant	Not Adjusted	871	Abia State University Teaching Hospital, Aba, Abia state, Federal Medical Centre, Keffi, Nasarawa State, Baru-Dokp Teaching Hospital, Kaduna, Kaduna State (Nigeria)	Dec 2020 - Nov 2021	Hospital	Mixed Groups	Mac (2023a)
Other, Sex	Not Significant	Not Adjusted		Coastal lowlands of SW Guatemala (Guatemala)	2015 - 2016	Population	Children	Lamb (2022)
Other, Sex	Not Significant	Not Adjusted	455	Unspecified	Mar 2014 - Oct 2017	Travel	General Population	Overbosch (2023)
Other, Sex	Not Significant	Unspecified	801	Krong Buk (Vietnam)	Jan 2017 - Jul 2018	Community	Mixed Groups	Nguyen (2020)
Sex	Not Significant	Not Adjusted		Vientiane capital (Laos)	2003 - 2015	Population	Blood Donors	Pastorino (2019)
Sex	Not Significant	Not Adjusted	501	Ouagadougou, Bobo-Dioulasso (Burkina Faso)	2020 - 2020	Population	Blood Donors	Tinto (2022)
Sex	Not Significant	Not Adjusted	1430	Mali	2013 - Nov 2016	Population	General Population	Diarra (2020)
Sex	Not Significant	Not Adjusted	585	"Peninsula" (Malaysia)	Unspecified	Population	General Population	Khor (2024)
**Guillain Barre syndrome**								
Infection	Significant	Not Adjusted	281	9 unspecified states (Mexico)	01 Jul 2016 - 30 Jun 2018	Hospital	Persons Under Investigation	Grijalva (2020)
Infection, Other	Significant	Adjusted	40	Barranquilla (Colombia)	01 Oct 2015 - 02 Apr 2016	Hospital	Other	Salinas (2017)
Infection, Other	Significant	Not Adjusted	41	Salvador, Bahia (Brazil)	01 Jan 2015 - 31 Aug 2015	Hospital	Other	Styczynski (2017)
Occupation, Other	Significant	Not Adjusted	29	Cucuta (Colombia)	29 Jun 2015 - 30 Jul 2016	Hospital	Other	Anaya (2017)
Other	Significant	Unspecified	191	Monterrey City (Mexico)	01 Aug 2017 - 30 Jun 2018	Hospital	Persons Under Investigation	Gongora-Rivera (2020)
Comorbidity, Occupation, Other, Sex	Not Significant	Not Adjusted	29	Cucuta (Colombia)	29 Jun 2015 - 30 Jul 2016	Hospital	Other	Anaya (2017)
Comorbidity, Other, Sex	Not Significant	Not Adjusted	41	Salvador, Bahia (Brazil)	01 Jan 2015 - 31 Aug 2015	Hospital	Other	Styczynski (2017)
Infection	Not Significant	Unspecified	191	Monterrey City (Mexico)	01 Aug 2017 - 30 Jun 2018	Hospital	Persons Under Investigation	Gongora-Rivera (2020)
Other	Not Significant	Not Adjusted		Bangladesh	2011 - 2015	Population	Mixed Groups	Geurtsvankessel (2018)
**Hospitalisation**								

continued on next page

continued from previous page

Risk factor name	Statistically significant	Adjusted	Sample size	Location	Dates	Population sample type	Population group	Reference
Age, Comorbidity, Other	Significant	Not Adjusted	94	Veterans Health Administration in US and Caribbean	01 Dec 2015 - 31 Oct 2016	Hospital	Other	Schirmer (2018)
**Low birthweight**								
Infection, Infection during pregnancy	Significant	Adjusted	291	French Guiana (France)	01 Jan 2015 - 15 Jul 2016	Mixed	Children	Pomar (2020)
Infection during pregnancy	Not Significant	Not Adjusted	745	Manaus (Brazil)	2015 - 2016	Community	Pregnant Women	De Fatima Redivo (2020)
**Microcephaly**								
Infection during pregnancy	Significant	Not Adjusted		French Polynesia (France)	Sep 2013 - Jul 2015	Population	Pregnant Women	Cauchemez (2016)
Infection during pregnancy	Significant	Not Adjusted	109	Tangará da Serra (Brazil)	01 Jan 2016 - 31 Dec 2016	Other	Children	Herrero Da Silva (2022)
Infection during pregnancy	Significant	Not Adjusted	745	Manaus (Brazil)	2015 - 2016	Community	Pregnant Women	De Fatima Redivo (2020)
Infection during pregnancy	Significant	Unspecified	108	Maranhao state (Brazil)	Mar 2015 - Sep 2018	Contact	Pregnant Women	Mendes (2020)
Infection during pregnancy, Occupation	Significant	Adjusted	64	Aracaju city in Sergipe (Brazil)	01 Sep 2015 - 05 Jan 2016	Hospital	Children	Santa Rita (2017)
Infection during pregnancy, Other	Significant	Adjusted	3603823	Brazil	01 Jan 2015 - 23 May 2017	Population	Children	Brady (2019)
Infection, Other	Significant	Adjusted	191	Paraíba (Brazil)	Jan 2015 - Dec 2016	Community	Children	Bezerra (2023)
Infection, Other, Sex	Significant	Not Adjusted	264	Recife (Brazil)	15 Jan 2016 - 30 Nov 2016	Hospital	Children	De Araujo (2018)
Other	Significant	Adjusted	163	Rio de Janeiro (Brazil)	Feb 2016 - Sep 2017	Other	Children	Power (2020)
Other	Significant	Not Adjusted	555	Martinique, Guadeloupe, French Guiana (France)	Mar 2016 - 27 Apr 2017	Population	Pregnant Women	Hoen (2018)
Other	Significant	Not Adjusted	163	Rio de Janeiro (Brazil)	Feb 2016 - Sep 2017	Other	Children	Power (2020)
Infection	Not Significant	Not Adjusted	98	Rio de Janeiro (Brazil)	2016 - 2016	Other	Children	De Melo Espindola (2021)
Infection during pregnancy	Not Significant	Not Adjusted	334	Dallas, Texas (United States)	14 Mar 2016 - 01 Oct 2016	Hospital	Children	Adhikari (2017)
Infection during pregnancy	Not Significant	Unspecified		Unspecified	Unspecified			Pomar (2017)
Infection during pregnancy	Not Significant	Unspecified	18	Hawaii (United States)	2009 - 2012	Other	Persons Under Investigation	Kumar (2016)
Occupation, Other	Not Significant	Not Adjusted	163	Rio de Janeiro (Brazil)	Feb 2016 - Sep 2017	Other	Children	Power (2020)
Other	Not Significant	Adjusted	3603828	Brazil	01 Jan 2015 - 23 May 2017	Population	Children	Brady (2019)
Other	Not Significant	Adjusted	163	Rio de Janeiro (Brazil)	Feb 2016 - Sep 2017	Other	Children	Power (2020)
Other	Not Significant	Not Adjusted	31	Goiania (Brazil)	01 Jan 2016 - 31 Dec 2017	Population	Pregnant Women	Rosado (2022)
**Miscarriage/stillbirth**								

continued on next page

continued from previous page

Risk factor name	Statistically significant	Adjusted	Sample size	Location	Dates	Population sample type	Population group	Reference
Infection during pregnancy	Significant	Adjusted	511	Ribeirão Preto (Brazil)	2015 - 2016	Population	Children	Coutinho (2021)
Infection during pregnancy	Significant	Adjusted		Centre Hospitalier de l'Ouest Guyanais (CHOG, Saint-Laurent-du-Maroni, in French Guiana) (France)	Jan 2015 - Jul 2016	Hospital	Pregnant Women	Pomar (2021)
Infection during pregnancy	Significant	Not Adjusted	511	Ribeirão Preto (Brazil)	2015 - 2016	Population	Children	Coutinho (2021)
Infection during pregnancy	Significant	Not Adjusted		Centre Hospitalier de l'Ouest Guyanais (CHOG, Saint-Laurent-du-Maroni, in French Guiana) (France)	Jan 2015 - Jul 2016	Hospital	Pregnant Women	Pomar (2021)
Infection, Infection during pregnancy	Significant	Not Adjusted	625	French Guiana (France)	01 Jan 2015 - 15 Jul 2016	Mixed	Children	Pomar (2020)
Infection during pregnancy	Not Significant	Not Adjusted		Centre Hospitalier de l'Ouest Guyanais (CHOG, Saint-Laurent-du-Maroni, in French Guiana) (France)	Jan 2015 - Jul 2016	Hospital	Pregnant Women	Pomar (2021)
Infection during pregnancy	Not Significant	Not Adjusted	762	Manaus (Brazil)	2015 - 2016	Community	Pregnant Women	De Fatima Redivo (2020)
**Other**								
Age, Other, Prior immunity to arboviruses	Significant	Not Adjusted	3893	Managua (Nicaragua)	01 Jan 2016 - 28 Feb 2017	Community	Children	Gordon (2019)
Age, Other, Prior immunity to arboviruses, Sex	Significant	Adjusted	3893	Managua (Nicaragua)	01 Jan 2016 - 28 Feb 2017	Community	Children	Gordon (2019)
Infection	Significant	Unspecified	59	United States	13 Jul 2016 - 19 Sep 2017	Hospital	Persons Under Investigation	El Sahly (2019)
Infection during pregnancy	Significant	Not Adjusted	109	Tangará da Serra (Brazil)	01 Jan 2016 - 31 Dec 2016	Other	Children	Herrero Da Silva (2022)
Infection during pregnancy	Significant	Not Adjusted	102	Colombia	Oct 2015 - Jan 2017	Hospital	Pregnant Women	Gutierrez-Sanchez (2022)
Infection, Infection during pregnancy	Significant	Adjusted	625	French Guiana (France)	01 Jan 2015 - 15 Jul 2016	Mixed	Children	Pomar (2020)
Other	Significant	Adjusted	163	Rio de Janeiro (Brazil)	Feb 2016 - Sep 2017	Other	Children	Power (2020)
Other	Significant	Adjusted	39331	Rio de Janeiro (Brazil)	2015 - 2016	Population	General Population	Raymundo (2021)
Other	Significant	Adjusted		Brazil	01 Aug 2015 - 31 Dec 2019	Population	Other	Pescarini (2022)

continued on next page

continued from previous page

Risk factor name	Statistically significant	Adjusted	Sample size	Location	Dates	Population sample type	Population group	Reference
Other	Significant	Adjusted	200	Chincha Baja and Pueblo Nuevo (Peru)	Mar 2019 - May 2019	Population	General Population	Cachay (2021)
Other	Significant	Not Adjusted	523	Brazil	19 Nov 2015 - 27 Feb 2016	Population	Pregnant Women	França (2016)
Other	Significant	Not Adjusted	602	Brazil	19 Nov 2015 - 27 Feb 2016	Population	Pregnant Women	França (2016)
Other	Significant	Not Adjusted		Queensland (Australia)	Apr 2016			Hugo (2019)
Other	Significant	Not Adjusted		Jundiai, Parana (Brazil)	2015 - 2016	Hospital	Mixed Groups	De Moraes (2022)
Age, Other, Sex	Not Significant	Adjusted	3893	Managua (Nicaragua)	01 Jan 2016 - 28 Feb 2017	Community	Children	Gordon (2019)
Age, Sex	Not Significant	Adjusted		Arauca, Armenia, Barranquilla, Bucara-manga, Cali, Cartagena, Cúcuta, Florencia, Ibagué, Inírida, Leticia, Medellín, Mitú, Mocoa, Montería, Neiva, Pereira, Popayán, Puerto Carreño, Quibdó, Riohacha, San Andrés, San José del Guaviare, Santa Marta, Sincelejo, Valledupar, Villavicencio, Yopal (Colombia)	2015 - 2017	Population	General Population	Charniga (2022)
Infection	Not Significant	Unspecified	59	United States	13 Jul 2016 - 19 Sep 2017	Hospital	Persons Under Investigation	El Sahly (2019)
Infection during pregnancy	Not Significant	Adjusted	83	Jundiai Hospital Medical school (Brazil)	01 Mar 2016 - 30 Jun 2019	Hospital	Pregnant Women	Gazeta (2021)
Infection during pregnancy	Not Significant	Not Adjusted	745	Manaus (Brazil)	2015 - 2016	Community	Pregnant Women	De Fatima Redivo (2020)
Other	Not Significant	Adjusted	163	Rio de Janeiro (Brazil)	Feb 2016 - Sep 2017	Other	Children	Power (2020)
Other	Not Significant	Not Adjusted		Jundiai, Parana (Brazil)	2015 - 2016	Hospital	Mixed Groups	De Moraes (2022)
Sex	Not Significant	Not Adjusted	3893	Managua (Nicaragua)	01 Jan 2016 - 28 Feb 2017	Community	Children	Gordon (2019)

continued on next page

continued from previous page

Risk factor name	Statistically significant	Adjusted	Sample size	Location	Dates	Population sample type	Population group	Reference
Other	Unspecified	Not Adjusted	5108	Belo Horizonte, Salvador, Laranjeiras, Fortaleza, Recife, Cuiaba and Campo Grande, Porto Velho (Brazil)	Jul 2016 - Jun 2019	Other	Other	Botosso (2023)
**Other neurological symptoms in general population**								
Age, Sex	Significant	Unspecified	418	Colombia	Jul 2015 - Oct 2017	Population	Other	Charniga (2021a)
Comorbidity, Hospitalisation	Significant	Not Adjusted	46	Veterans Health Administration in US and Caribbean	01 Dec 2015 - 31 Oct 2016	Hospital	Other	Schirmer (2018)
Infection	Significant	Not Adjusted		Guadeloupe, Martinique (France)	06 Jan 2016	Hospital	Persons Under Investigation	Lannuzel (2019)
Infection during pregnancy	Significant	Adjusted		Centre Hospitalier de l'Ouest Guyanais (CHOG, Saint-Laurent-du-Maroni, in French Guiana) (France)	Jan 2015 - Jul 2016	Hospital	Pregnant Women	Pomar (2021)
Infection during pregnancy	Significant	Not Adjusted	129	León (Nicaragua)	Feb 2017 - Jul 2017	Other	Children	Stringer (2021)
Infection during pregnancy	Significant	Not Adjusted		Centre Hospitalier de l'Ouest Guyanais (CHOG, Saint-Laurent-du-Maroni, in French Guiana) (France)	Jan 2015 - Jul 2016	Hospital	Pregnant Women	Pomar (2021)
Infection during pregnancy, Other	Significant	Adjusted	129	León (Nicaragua)	Feb 2017 - Jul 2017	Other	Children	Stringer (2021)
Age, Other	Not Significant	Not Adjusted	129	León (Nicaragua)	Feb 2017 - Jul 2017	Other	Children	Stringer (2021)
Age, Other, Sex	Not Significant	Adjusted	129	León (Nicaragua)	Feb 2017 - Jul 2017	Other	Children	Stringer (2021)

continued on next page

continued from previous page

Risk factor name	Statistically significant	Adjusted	Sample size	Location	Dates	Population sample type	Population group	Reference
Age, Sex	Not Significant	Adjusted		Arauca, Armenia, Barranquilla, Bucaramanga, Cali, Cartagena, Cúcuta, Florencia, Ibagué, Inírida, Leticia, Medellín, Mitú, Mocoa, Montería, Neiva, Pereira, Popayán, Puerto Carreño, Quibdó, Riohacha, San Andrés, San José del Guaviare, Santa Marta, Sincelejo, Valledupar, Villavicencio, Yopal (Colombia)	2015 - 2017	Population	General Population	Charniga (2022)
Age, Sex	Not Significant	Unspecified	418	Colombia	Jul 2015 - Oct 2017	Population	Other	Charniga (2021a)
Infection	Not Significant	Not Adjusted	98	Rio de Janeiro (Brazil)	2016 - 2016	Other	Children	De Melo Espindola (2021)
Infection during pregnancy	Not Significant	Adjusted	152	Tegucigalpa (Honduras)	Jul 2016 - Dec 2016	Hospital	Children	Alger (2024)
Infection during pregnancy	Not Significant	Not Adjusted		Centre Hospitalier de l'Ouest Guyanais (CHOG, Saint-Laurent-du-Maroni, in French Guiana) (France)	Jan 2015 - Jul 2016	Hospital	Pregnant Women	Pomar (2021)
Other	Not Significant	Adjusted		Centre Hospitalier de l'Ouest Guyanais (CHOG, Saint-Laurent-du-Maroni, in French Guiana) (France)	Jan 2015 - Jul 2016	Hospital	Pregnant Women	Pomar (2021)
Other	Not Significant	Not Adjusted		Centre Hospitalier de l'Ouest Guyanais (CHOG, Saint-Laurent-du-Maroni, in French Guiana) (France)	Jan 2015 - Jul 2016	Hospital	Pregnant Women	Pomar (2021)

continued on next page

continued from previous page

Risk factor name	Statistically significant	Adjusted	Sample size	Location	Dates	Population sample type	Population group	Reference
Other	Not Significant	Not Adjusted		Guadeloupe, Martinique (France)	06 Jan 2016	Hospital	Persons Under Investigation	Lannuzel (2019)
**Premature birth**								
Infection during pregnancy	Significant	Not Adjusted	745	Manaus (Brazil)	2015 - 2016	Community	Pregnant Women	De Fatima Redivo (2020)
Infection	Not Significant	Not Adjusted	98	Rio de Janeiro (Brazil)	2016 - 2016	Other	Children	De Melo Espindola (2021)
Infection	Not Significant	Not Adjusted	250	New York (United States)	2016 - 2016	Population	Pregnant Women	Cooper (2019)
**Severe disease (in general population)**								
Age, Other, Sex	Significant	Not Adjusted	10319	Mexico	Jan 2012 - Mar 2020	Population	Persons Under Investigation	Ananth (2020)
Prior immunity to arboviruses	Not Significant	Not Adjusted	997	Philippines	2016 - 2016	Population	Persons Under Investigation	Biggs (2021)
**Symptomatic infection**								
Age, Comorbidity, Sex	Significant	Adjusted	114	San Juan, Puerto Rico (United States)	16 Sep 2016 - 27 Oct 2016	Contact	Other	Lozier (2018)
Age, Comorbidity, Sex	Significant	Not Adjusted	114	San Juan, Puerto Rico (United States)	16 Sep 2016 - 27 Oct 2016	Contact	Other	Lozier (2018)
Other	Not Significant	Adjusted	114	San Juan, Puerto Rico (United States)	16 Sep 2016 - 27 Oct 2016	Contact	Other	Lozier (2018)
Other	Not Significant	Not Adjusted	114	San Juan, Puerto Rico (United States)	16 Sep 2016 - 27 Oct 2016	Contact	Other	Lozier (2018)
**Zika congenital syndrome/other birth defects**								
Infection during pregnancy	Significant		50	Rio de Janeiro and Manaus (Brazil)	Sep 2015 - Jun 2016	Population	Pregnant Women	Damasceno (2020)
Infection during pregnancy	Significant	Adjusted	21	Tahiti, Mo'orea, French Polynesia (France)	01 Jun 2013 - 31 Aug 2014	Hospital	Children	Subissi (2018)
Infection during pregnancy	Significant	Adjusted	219	Rio de Janeiro (Brazil)	Jan 2015 - Aug 2016	Hospital	Pregnant Women	João (2018)
Infection during pregnancy	Significant	Adjusted	511	Ribeirão Preto (Brazil)	2015 - 2016	Population	Children	Coutinho (2021)
Infection during pregnancy	Significant	Adjusted	291	French Guiana (France)	01 Jan 2015 - 15 Jul 2016	Mixed	Children	Pomar (2020)
Infection during pregnancy	Significant	Adjusted	129	western French Guiana (France)	Jan 2016 - Sep 2016	Hospital	Mixed Groups	Hcini (2021)
Infection during pregnancy	Significant	Adjusted	83	Jundiai Hospital Medical school (Brazil)	01 Mar 2016 - 30 Jun 2019	Hospital	Pregnant Women	Gazeta (2021)
Infection during pregnancy	Significant	Not Adjusted	511	Ribeirão Preto (Brazil)	2015 - 2016	Population	Children	Coutinho (2021)

continued on next page

continued from previous page

Risk factor name	Statistically significant	Adjusted	Sample size	Location	Dates	Population sample type	Population group	Reference
Infection during pregnancy	Significant	Not Adjusted	557	Jundiai, São Paulo (Brazil)	01 Mar 2016 - 23 Aug 2017	Hospital	Children	Clemente (2020)
Infection during pregnancy	Significant	Not Adjusted	129	western French Guiana (France)	Jan 2016 - Sep 2016	Hospital	Mixed Groups	Hcini (2021)
Infection during pregnancy	Significant	Not Adjusted	42	Rio de Janeiro (Brazil)	Apr 2015 - Oct 2017	Hospital	Children	Lima (2019)
Infection during pregnancy	Significant	Not Adjusted	94	Rio de Janeiro (Brazil)	01 Mar 2016 - 30 Jun 2017	Hospital	Pregnant Women	Pool (2019)
Infection during pregnancy	Significant	Unspecified	108	Maranhao state (Brazil)	Mar 2015 - Sep 2018	Contact	Pregnant Women	Mendes (2020)
Infection during pregnancy, Other	Significant	Adjusted	3603828	Brazil	01 Jan 2015 - 23 May 2017	Population	Children	Brady (2019)
Infection during pregnancy, Other	Significant	Not Adjusted	865	Unspecified	Jan 2016 - Jul 2019	Other	Pregnant Women	Vouga (2021)
Infection, Infection during pregnancy, Other	Significant	Adjusted	174	Ceará (Brazil)	Oct 2015 - Jun 2017	Population	Children	Rocha (2019)
Infection, Infection during pregnancy, Other	Significant	Unspecified	157	Paraíba (Brazil)	Unspecified	Other	Persons Under Investigation	Krow-Lucal (2018)
Other	Significant	Adjusted	143	Rio de Janeiro (Brazil)	Dec 2015	Other	Children	Azamor (2021)
Other	Significant	Not Adjusted	555	Martinique, Guadeloupe, French Guiana (France)	Mar 2016 - 27 Apr 2017	Population	Pregnant Women	Hoen (2018)
Other	Significant	Not Adjusted	120	Mato Grosso do Sul (Brazil)	Nov 2017 - Oct 2018	Population	Persons Under Investigation	De Sousa (2020)
Other	Significant	Not Adjusted		Salvador, Bahia (Brazil)	01 Jan 2015 - 31 Jan 2017	Hospital	Mixed Groups	Aromolaran (2022)
Other, Sex	Significant	Adjusted	11366686	Brazil	01 Jan 2015 - 31 Dec 2018	Population	Children	Paixão (2022)
Age	Not Significant	Adjusted	11366686	Brazil	01 Jan 2015 - 31 Dec 2018	Population	Children	Paixão (2022)
Age, Other	Not Significant	Not Adjusted	42	Rio de Janeiro (Brazil)	Apr 2015 - Oct 2017	Hospital	Children	Lima (2019)
Comorbidity, Other	Not Significant	Not Adjusted	120	Mato Grosso do Sul (Brazil)	Nov 2017 - Oct 2018	Population	Persons Under Investigation	De Sousa (2020)
Infection during pregnancy	Not Significant	Adjusted	174	Thailand	1997 - 2015	Other	Pregnant Women	Ngo-Giang-Huong (2021)
Infection during pregnancy	Not Significant	Adjusted	376	Pernambuco (Recife) (Brazil)	Dec 2015 - Jun 2017	Hospital	Pregnant Women	Ximenes (2021)
Infection during pregnancy	Not Significant	Not Adjusted	1236	Mombasa (Kenya)	Oct 2017 - Jul 2019	Hospital	Children	Osoro (2022)
Infection during pregnancy	Not Significant	Not Adjusted	102	Colombia	Oct 2015 - Jan 2017	Hospital	Pregnant Women	Gutierrez-Sanchez (2022)
Infection during pregnancy	Not Significant	Not Adjusted	557	Jundiai, São Paulo (Brazil)	01 Mar 2016 - 23 Aug 2017	Hospital	Children	Clemente (2020)
Infection during pregnancy	Not Significant	Not Adjusted	70	New York (United States)	Mar 2016 - Apr 2016	Hospital	Pregnant Women	Merriam (2020)

continued on next page

continued from previous page

Risk factor name	Statistically significant	Adjusted	Sample size	Location	Dates	Population sample type	Population group	Reference
Infection during pregnancy, Other, Sex	Not Significant	Not Adjusted	80	Santo Domingo, Santiago (Dominican Republic)	2016 - 2019	Hospital	Children	Pimintel (2021)
Infection, Infection during pregnancy, Other	Not Significant	Unspecified	157	Paraíba (Brazil)	Unspecified	Other	Persons Under Investigation	Krow-Lucal (2018)
Infection, Infection during pregnancy, Other	Not Significant	Unspecified	194	León (Nicaragua)	Feb 2017 - Jul 2017	Hospital	Children	Collins (2020)
Other	Not Significant	Adjusted	143	Rio de Janeiro (Brazil)	Dec 2015	Other	Children	Azamor (2021)
Other	Not Significant	Not Adjusted	143	Rio de Janeiro (Brazil)	Dec 2015	Other	Children	Azamor (2021)
**ZIKV detection**								
Age, Occupation, Other	Not Significant	Not Adjusted	315	University Hospital of the Faculty of Medicine of Jundiaí, Jundiaí, São-Paulo (Brazil)	Mar 2016 - Aug 2017	Hospital	Pregnant Women	Pires (2021)
Other	Not Significant	Adjusted	315	University Hospital of the Faculty of Medicine of Jundiaí, Jundiaí, São-Paulo (Brazil)	Mar 2016 - Aug 2017	Hospital	Pregnant Women	Pires (2021)

Table B.11: Overview of extracted risk factors. Levels of statistical significance may be different between studies.

B.7.6 Transmission

Parameter value	Uncertainty	Disaggr-egation	Method for R estimation	Sample size	Location	Dates	Population sample type	Population group	Reference
**Attack Rate**									
0	95% CrI: 0-0				Federal District (Mexico)	28 Nov 2015 - 05 Nov 2018	Population	General Population	Moore (2020)
0.007	95% CI: 595-772	Method		11247972	Rio Grande (Brazil)	Oct 2015 - May 2016	Population	Persons Under Investigation	Zhao (2019)
0.016	95% CrI: 0.01-0.025				State of Paraná (Brazil)	Apr 2015 - 31 Dec 2018	Population	General Population	Moore (2020)
0.03	95% CrI: 0-0.1				Quibdó (Colombia)	2015 - 2017	Population	General Population	Charniga (2022)
0.034	95% CI: 2040-2578	Method		6610681	Goiania City (Brazil)	Oct 2015 - May 2016	Population	Persons Under Investigation	Zhao (2019)
0.036	95% CI: 3610-4382	Method		11163018	Panara (Brazil)	Oct 2015 - May 2016	Population	Persons Under Investigation	Zhao (2019)
0.05 %	95% CI: 0.01-0.29			1962	Campinas (Brazil)	Mar 2015 - Mar 2015	Population	Blood Donors	Benites (2019)
0.054	95% CI: 1671-2364	Method		3929911	Espirito Santo (Brazil)	Oct 2015 - May 2016	Population	Persons Under Investigation	Zhao (2019)
0.084	95% CrI: 0.067-0.096				Peru	01 Jan 2016 - 15 Sep 2018	Population	General Population	Moore (2020)
0.09	95% CrI: 0.02-0.19				Costa Rica	01 Jan 2016 - 31 Dec 2017	Population	General Population	Moore (2020)
0.097	95% CI: 772-925	Method		803513	Acre (Brazil)	Oct 2015 - May 2016	Population	Persons Under Investigation	Zhao (2019)
0.106	95% CI: 9320-10721	Method		9345173	Pernambuco State (Brazil)	Oct 2015 - May 2016	Population	Persons Under Investigation	Zhao (2019)
0.12	95% CrI: 0.06-0.22				Panama	22 Nov 2015 - 10 Nov 2018	Population	General Population	Moore (2020)
0.16	95% CrI: 0.07-0.29				Bolivia	01 Jan 2016 - 30 Jun 2018	Population	General Population	Moore (2020)
0.17 %	95% CI: 0.06-0.5			1775	Campinas (Brazil)	Mar 2016 - Mar 2016	Population	Blood Donors	Benites (2019)
0.19	95% CrI: 0.15-0.23				Colombia	09 Aug 2015 - 31 Dec 2017	Population	General Population	Moore (2020)
0.2	95% CrI: 0.15-0.25				Mexico	28 Nov 2015 - 05 Nov 2018	Population	General Population	Moore (2020)
0.23	95% CrI: 0.07-0.49				Belize	01 Jan 2016 - 31 Dec 2017	Population	General Population	Moore (2020)
0.23	95% CrI: 0.16-0.29				Guatemala	29 Nov 2015 - 20 Oct 2018	Population	General Population	Moore (2020)
0.25	95% CrI: 0.19-0.31				Brazil	Apr 2015 - 31 Dec 2018	Population	General Population	Moore (2020)
0.25	95% CrI: 0.18-0.33				Dominican Republic	01 Jan 2016 - 25 Mar 2018	Population	General Population	Moore (2020)
0.28	95% CrI: 0.16-0.4				El Salvador	22 Nov 2015 - 16 Dec 2017	Population	General Population	Moore (2020)
0.32	95% CrI: 0.29-0.35				Puerto Rico (United States)	01 Jan 2007 - 07 Jan 2017	Population	General Population	Moore (2020)
0.33	95% CrI: 0.21-0.46				Nicaragua	27 Jan 2016 - 31 Dec 2017	Population	General Population	Moore (2020)
0.36	95% CrI: 0.22-0.49				Honduras	13 Dec 2015 - 04 Jan 2018	Population	General Population	Moore (2020)
0.361	95% CrI: 0.214-0.514				Ecuador	20 Dec 2015 - 23 Oct 2018	Population	General Population	Moore (2020)
0.365	95% CI: 50249-59773	Method		15203934	Bahia (Brazil)	Oct 2015 - May 2016	Population	Persons Under Investigation	Zhao (2019)

continued on next page

continued from previous page

Parameter value	Uncertainty	Disaggr-egation	Method for R estimation	Sample size	Location	Dates	Population sample type	Population group	Reference
0.38	95% CrI: 0.17-0.92	Region			Arauca, Armenia, Barranquilla, Bucaramanga, Cali, Cartagena, Cúcuta, Florencia, Ibagué, Inírida, Leticia, Medellín, Mitú, Mocoa, Montería, Neiva, Pereira, Popayán, Puerto Carreño, Quibdó, Riohacha, San Andrés, San José del Guaviare, Santa Marta, Sincelejo, Valledupar, Villavicencio, Yopal (Colombia)	2015 - 2017	Population	General Population	Charniga (2022)
0.514	95% CrI: 0.388-0.631				Bahia (Brazil)	Apr 2015 - 31 Dec 2018	Population	General Population	Moore (2020)
0.606	95% CI: 12901-19791	Method		3265486	Mato Grosso (Brazil)	Oct 2015 - May 2016	Population	Persons Under Investigation	Zhao (2019)
0.766	95% CrI: 0.569-0.942				State of Sergipe (Brazil)	Apr 2015 - 31 Dec 2018	Population	General Population	Moore (2020)
0.793	95% CrI: 0.524-0.963				State of Yucatán (Mexico)	28 Nov 2015 - 05 Nov 2018	Population	General Population	Moore (2020)
0.8	95% CrI: 0.56-0.99				San Andrés (Colombia)	2015 - 2017	Population	General Population	Charniga (2022)
1.213 %		Age, Sex		928	San Andrés (Colombia)	06 Sep 2015 - 30 Jan 2016	Hospital	Persons Under Investigation	Rojas (2016)
1.843 %		Age, Sex		1936	Girardot (Colombia)	19 Oct 2015 - 22 Jan 2016	Hospital	Persons Under Investigation	Rojas (2016)
2.7 per 10k		Age			Dominican Republic	Jan 2016 - Dec 2016	Population	General Population	Bowman (2018)
5.2 per 10k				5235	Dominican Republic	Jan 2016 - Dec 2016	Population	General Population	Bowman (2018)
5.5 %	95% CI: 3-10.4			997	Philippines	2016 - 2016	Population	General Population	Biggs (2021)
7.6 per 10k		Age			Dominican Republic	Jan 2016 - Dec 2016	Population	General Population	Bowman (2018)
12 %				34	China	Feb 2016 - Feb 2016	Travel	Other	Sun (2017)
14.4 %	95% CI: 5.6-27.4				Wynwood Neighborhood, Miami-Dade County, Florida (United States)	01 Jun 2016 - 05 Aug 2016	Population	General Population	Marini (2017)
14.6 per 10k		Age, Region, Sex		185	Yap Island (Federated States of Micronesia)	01 Apr 2007 - 31 Jul 2007	Hospital	Persons Under Investigation	Duffy (2009)
20 per 100k		Age, Method, Other, Region, Sex		48747574	Colombia	10 Oct 2015 - 24 Jun 2017	Population	General Population	Méndez (2017)
20.8 %	95% CI: 1.1-50.3				Colombia	Aug 2015 - May 2016	Population	General Population	He (2017)
32.4 %	95% CI: 2.5-94.2				Bahia (Brazil)	Feb 2015 - Feb 2016	Population	General Population	He (2017)
50 %	95% CrI: 43-58				Martinique (France)	2015 - 2016	Mixed	Mixed Groups	Cousien (2019)
%				1110	Managua (Nicaragua)	Jan 2016 - Feb 2017	Hospital	Persons Under Investigation	Burger-Calderon (2020)
51.1	Other: 42.1-61.1	Method		7580	Santiago, Fogo, Maio, Boavista (Cabo Verde)	05 Oct 2015 - 29 May 2016	Population	Children	Lourenco (2018)

continued on next page

continued from previous page

Parameter value	Uncertainty	Disaggr-egation	Method for R estimation	Sample size	Location	Dates	Population sample type	Population group	Reference
62.5 %	95% CI: 59.2-82.5				"Other four archipelagos", French Polynesia (France)	Aug 2013 - May 2014	Population	General Population	He (2017)
65 %	95% CI: 57-72.9				Feira de Santana (Brazil)	01 Feb 2015 - 30 Apr 2017	Population	General Population	Lourenco (2017)
70.1 %	95% CI: 66.3-92.5				Sous-le-vent Islands, French Polynesia (France)	Aug 2013 - May 2014	Population	General Population	He (2017)
71.3 %	95% CI: 67.4-94.1				Tahiti, French Polynesia (France)	Aug 2013 - May 2014	Population	General Population	He (2017)
73 %	95% CI: 70-76	Region		1453	Salvador, Bahia (Brazil)	2015 - 2015	Population	General Population	Rodriguez-Barraquer (2019)
78 %	95% CI: 63.5-86.3	Region			French Polynesia (France)	Aug 2013 - May 2014	Population	General Population	He (2017)
94 %			Compartmental Model		French Polynesia (France)	11 Oct 2013 - 28 Mar 2014	Population	Persons Under Investigation	Kucharski (2016)
552 per 100k		Age			Cucuta (Colombia)	29 Jun 2015 - 30 Jul 2016	Hospital	Persons Under Investigation	Anaya (2017)
1306 per 100k		Age			Cucuta (Colombia)	29 Jun 2015 - 30 Jul 2016	Hospital	Persons Under Investigation	Anaya (2017)
1 - 43 %		Region			Multi-country: Central and South America (n = 8)	Jan 2014 - 01 Feb 2016	Hospital	Persons Under Investigation	Zhang (2017)
5 - 49 %		Region			Multi-country: Central and South America (n = 8)	01 Feb 2016 - 28 Feb 2017	Hospital	Persons Under Investigation	Zhang (2017)
0.897 - 21.16 per 10k	95% CI: 0.897-21.548	Region			All municipalities (Dominican Republic)	2014 - 2016	Population	General Population	Kingston (2022)
0.219 - 0.285		Method			Northeastern (Brazil)	Jan 2015 - Nov 2016	Population	Persons Under Investigation	He (2020)
46 - 53 %		Method			Fiji	May 2013 - Nov 2017	Population	Persons Under Investigation	Henderson (2021)
0 - 0.9 %		Method, Other		481	Netherlands	Mar 2014 - Oct 2017	Travel	General Population	Overbosch (2023)
**Basic Reproduction Number**									
0.237		Method, Other	Compartmental Model		Amazonas (Brazil)	06 Feb 2021 - 30 Apr 2021	Population	Persons Under Investigation	Omame (2023a)
0.494			Unspecified		Colombia	2015 - 2015	Unspecified	Unspecified	Zafar (2024)
0.548			Compartmental Model		Colombia	2016 - 2016	Unspecified	Unspecified	Alzahrani (2021)
0.737			Compartmental Model		Suriname	Unspecified	Population	General Population	Dénes (2019)
0.924			Compartmental Model		Costa Rica	2016 - 2018	Population	General Population	Dénes (2019)
0.98	95% CI: 0.93-1.02		Compartmental Model	1841	Envigado (Colombia)	01 Jan 2016 - 11 Apr 2016	Population	Persons Under Investigation	Ospina (2017)
0.98			Compartmental Model	1841	Itagüí (Colombia)	01 Jan 2016 - 11 Apr 2016	Population	Persons Under Investigation	Ospina (2017)
0.98	95% CI: 0.88-1.08		Compartmental Model	1841	Mutatá (Colombia)	01 Jan 2016 - 11 Apr 2016	Population	Persons Under Investigation	Ospina (2017)
0.98	95% CI: 0.92-1.03		Compartmental Model	1841	Rionegro (Colombia)	01 Jan 2016 - 11 Apr 2016	Population	Persons Under Investigation	Ospina (2017)
0.98			Compartmental Model	1841	Zaragoza (Colombia)	01 Jan 2016 - 11 Apr 2016	Population	Persons Under Investigation	Ospina (2017)
1.009	95% CI: 0.99-1.02		Compartmental Model	1841	Caucasia (Colombia)	01 Jan 2016 - 11 Apr 2016	Population	Persons Under Investigation	Ospina (2017)
1.03	95% CI: 0.97-1.09		Compartmental Model	1841	Puerto Triunfo (Colombia)	01 Jan 2016 - 11 Apr 2016	Population	Persons Under Investigation	Ospina (2017)

continued on next page

continued from previous page

Parameter value	Uncertainty	Disaggr-egation	Method for R estimation	Sample size	Location	Dates	Population sample type	Population group	Reference
1.05	95% CI: 1.03-1.08		Other		Guatemala	Unspecified			Hsieh (2017)
1.07	95% CI: 0.8-1.26		Compartmental Model	1841	Sopetrán (Colombia)	01 Jan 2016 - 11 Apr 2016	Population	Persons Under Investigation	Ospina (2017)
1.08	95% CI: 1.02-1.15		Compartmental Model	1841	Puerto Berrío (Colombia)	01 Jan 2016 - 11 Apr 2016	Population	Persons Under Investigation	Ospina (2017)
1.11	95% CI: 1.09-1.13		Compartmental Model	1841	Apartadó (Colombia)	01 Jan 2016 - 11 Apr 2016	Population	Persons Under Investigation	Ospina (2017)
1.117			Other		Tuamotu-Gambier, French Polynesia (France)	2013 - 2014	Population	Persons Under Investigation	Prasad (2023)
1.12		Region	Compartmental Model	1841	Antioquia (Colombia)	01 Jan 2016 - 11 Apr 2016	Population	Persons Under Investigation	Ospina (2017)
1.12	95% CI: 0.99-1.24		Compartmental Model	1841	Necoclí (Colombia)	01 Jan 2016 - 11 Apr 2016	Population	Persons Under Investigation	Ospina (2017)
1.12	95% CrI: 0.8-1.5	Method, Time	Compartmental Model		Fiji	2013 - 2018	Population	Persons Under Investigation	Henderson (2021)
1.13	95% CI: 0.84-1.42		Compartmental Model	1841	Remedios (Colombia)	01 Jan 2016 - 11 Apr 2016	Population	Persons Under Investigation	Ospina (2017)
1.142			Other		Marquesas Islands, French Polynesia (France)	2013 - 2014	Population	Persons Under Investigation	Prasad (2023)
1.15	95% CI: 1.12-1.17		Compartmental Model	1841	Turbo (Colombia)	01 Jan 2016 - 11 Apr 2016	Population	Persons Under Investigation	Ospina (2017)
1.151			Other		Mo'orea, French Polynesia (France)	2013 - 2014	Population	Persons Under Investigation	Prasad (2023)
1.172			Other		Colombia	2016 - 2016	Population	Persons Under Investigation	Prasad (2023)
1.18	95% CI: 1.06-1.3		Compartmental Model	1841	Bello (Colombia)	01 Jan 2016 - 11 Apr 2016	Population	Persons Under Investigation	Ospina (2017)
1.18	95% CI: 1.04-1.41		Other	751	Porto Velho (Brazil)	Jul 2016 - Jun 2019	Other	Other	Botosso (2023)
1.185			Other		Sous-le-vent Islands, French Polynesia (France)	2013 - 2014	Population	Persons Under Investigation	Prasad (2023)
1.225			Other		Australes, French Polynesia (France)	2013 - 2014	Population	Persons Under Investigation	Prasad (2023)
1.23	95% CI: 1.18-1.28		Compartmental Model	1841	Carepa (Colombia)	01 Jan 2016 - 11 Apr 2016	Population	Persons Under Investigation	Ospina (2017)
1.28	95% CI: 1.08-1.69		Other	817	Belo Horizonte (Brazil)	Jul 2016 - Jun 2016	Other	Other	Botosso (2023)
1.31	95% CI: 1.1-1.52		Compartmental Model	1841	San Pedro de Urabá (Colombia)	01 Jan 2016 - 11 Apr 2016	Population	Persons Under Investigation	Ospina (2017)
1.41	95% CI: 1.15-1.74		Other	928	San Andrés (Colombia)	06 Sep 2015 - 30 Jan 2016	Hospital	Persons Under Investigation	Rojas (2016)
1.41	95% CI: 1.12-2.08		Other	494	Salvador, Bahia (Brazil)	Jul 2016 - Jun 2019	Other	Other	Botosso (2023)
1.43	95% CI: 1.21-1.85		Other	345	Fortaleza (Brazil)	Jul 2016 - Jun 2019	Other	Other	Botosso (2023)
1.53	95% CI: 1.71-1.89		Compartmental Model	1841	Chigorodó (Colombia)	01 Jan 2016 - 11 Apr 2016	Population	Persons Under Investigation	Ospina (2017)
1.54	95% CI: 1.35-1.92	Region	Other	5108	Belo Horizonte, Salvador, Laranjeiras, Fortaleza, Recife, Cuiaba and Campo Grande, Porto Velho (Brazil)	Jul 2016 - Jun 2019	Other	Other	Botosso (2023)
1.58	95% CrI: 1.56-1.59		Next Generation Matrix		Managua (Nicaragua)	2016 - 2016	Population	General Population	Counotte (2019)
1.59	95% CI: 1.28-1.9	Time	Other		Guatemala	2015 - 2016			Hsieh (2017)
1.6	95% CrI: 1.5-1.7		Next Generation Matrix		Tahiti, French Polynesia (France)	Oct 2013 - Apr 2014	Population	Persons Under Investigation	Champagne (2016)

continued on next page

continued from previous page

Parameter value	Uncertainty	Disaggr-egation	Method for R estimation	Sample size	Location	Dates	Population sample type	Population group	Reference
1.6	95% CrI: 1.5-1.7		Next Generation Matrix		New Caledonia (France)	12 Nov 2013 - Aug 2014	Population	Persons Under Investigation	Champagne (2016)
1.67	95% CI: 1.43-3.57		Other	1017	Cuiaba and Campo Grande (Brazil)	Jul 2016 - Jun 2019	Other	Other	Botosso (2023)
1.68	95% CI: 1.32-2.04		Other		Suriname	2015 - 2016			Hsieh (2017)
1.72	95% CI: 1.69-1.75	Age	Growth Rate		Dominican Republic	Jan 2016 - Dec 2016	Population	General Population	Bowman (2018)
1.727			Next Generation Matrix		Colombia	2015 - Dec 2016	Population	General Population	Valega-Mackenzie (2023)
1.75	95% CI: 1.34-2.16	Time	Other		Colombia	2015 - 2016			Hsieh (2017)
1.79	95% CI: 1.29-2.3		Other		Colombia	Unspecified			Hsieh (2017)
1.79	Other: 1.68-1.91		Other		Yap Island, French Polynesia (France)	2007 - 2007	Population	General Population	Gao (2016)
1.8	95% CI: 1.7-1.9	Region	Other	1453	Salvador, Bahia (Brazil)	2015 - 2015	Population	General Population	Rodriguez-Barraquer (2019)
1.8			Other	573	Dominican Republic	30 Jan 2016 - 27 Feb 2016	Population	Persons Under Investigation	Rocklov (2016)
1.8	95% CrI: 1.6-2		Next Generation Matrix		Mo'orea, French Polynesia (France)	Oct 2013 - Apr 2014	Population	Persons Under Investigation	Champagne (2016)
1.8	95% CI: 1.78-1.82		Growth Rate	5235	Dominican Republic	Jan 2016 - Dec 2016	Population	General Population	Bowman (2018)
1.81	95% CrI: 1.74-1.87	Other, Region	Compartmental Model		90 major cities across LAC (Multi-country: Americas (n = 32))	2015 - 2017	Population	General Population	O'Reilly (2018)
1.816			Compartmental Model		Brazil	25 Mar 2016 - 14 Apr 2018	Population	Persons Under Investigation	Zhao (2020)
1.82	95% CI: 1.19-2.68		Growth Rate		Rio de Janeiro (Brazil)	2012			Villela (2017)
1.84	95% CI: 1.82-1.87	Age	Growth Rate		Dominican Republic	Jan 2016 - Dec 2016	Population	General Population	Bowman (2018)
1.85	95% CI: 1.5-2.2		Growth Rate	7580	Santiago, Fogo, Maio, Boavista (Cabo Verde)	05 Oct 2015 - 29 May 2016	Population	Persons Under Investigation	Lourenco (2018)
1.866			Other		Costa Rica	2016 - 2016	Population	Persons Under Investigation	Prasad (2023)
1.88	95% CrI: 1.59-2.22	Region	Branching Process	1e+05	French Polynesia and French West Indies (France)	Oct 2013 - Oct 2016	Population	Persons Under Investigation	Riou (2017)
1.89	95% CI: 1.21-2.13	Method	Compartmental Model	18364	Colombia	2015 - 2016	Population	Pregnant Women	Sasmal (2018)
1.89	95% CI: 1.33-3.23		Other	1110	Recife (Brazil)	Jul 2016 - Jun 2019	Other	Other	Botosso (2023)
1.9			Other	49	Panama	01 Jan 2016 - 13 Mar 2016	Population	Persons Under Investigation	Rocklov (2016)
1.947			Other		Tahiti, French Polynesia (France)	2013 - 2014	Population	Persons Under Investigation	Prasad (2023)
2	95% CrI: 1.8-2.2		Next Generation Matrix		New Caledonia (France)	12 Nov 2013 - Aug 2014	Population	Persons Under Investigation	Champagne (2016)
2.027	95% CI: 1.907-2.14				Australes, French Polynesia (France)	Oct 2013 - Mar 2014	Population	General Population	Rahman (2019)
2.03	95% CrI: 1.63-2.36		Other		Australes, French Polynesia (France)	01 Dec 2015 - 30 Mar 2017	Community	Persons Under Investigation	Riou (2018)
2.05	95% CrI: 1.71-2.37		Other		Tuamotu, French Polynesia (France)	20 Oct 2015 - 02 Mar 2017	Community	Persons Under Investigation	Riou (2018)

continued on next page

continued from previous page

Parameter value	Uncertainty	Disaggr-egation	Method for R estimation	Sample size	Location	Dates	Population sample type	Population group	Reference
2.055	95% CI: 0.523-6.3		Compartmental Model		Multi-country: Brazil, Colombia, El Salvador	Apr 2015 - 27 Feb 2016	Population	General Population	Gao (2016)
2.069	95% CI: 1.261-4.316				Marquesas Islands, French Polynesia (France)	Oct 2013 - Mar 2014	Population	General Population	Rahman (2019)
2.1			Other	717	Guadeloupe (France)	29 Jan 2016 - 10 Mar 2016	Population	Persons Under Investigation	Rocklov (2016)
2.1			Other	129	Mexico	01 Jan 2016 - 13 Mar 2016	Population	Persons Under Investigation	Rocklov (2016)
2.1	95% CrI: 1.78-2.47		Other		Mo'orea, French Polynesia (France)	27 Oct 2015 - 09 Mar 2017	Community	Persons Under Investigation	Riou (2018)
2.1	95% CrI: 1.8-2.43		Other		Sous-le-vent Islands, French Polynesia (France)	27 Oct 2015 - 23 Mar 2017	Community	Persons Under Investigation	Riou (2018)
2.1	95% CrI: 1.86-2.35		Other		Tahiti, French Polynesia (France)	13 Oct 2015 - 16 Mar 2017	Community	Persons Under Investigation	Riou (2018)
2.12	95% CrI: 1.82-2.47		Other		Marquesas Islands, French Polynesia (France)	27 Oct 2015 - 23 Mar 2017	Community	Persons Under Investigation	Riou (2018)
2.13	95% CI: 1.61-2.64		Next Generation Matrix		Brazil	Jan 2015 - Sep 2016	Population	General Population	Wang (2024a)
2.135	95% CI: 2.07-2.2		Next Generation Matrix		Unspecified	01 Oct 2013 - 30 Apr 2014			Gwalani (2018)
2.2	95% CI: 1.54-2.86		Compartmental Model	1841	Nechí (Colombia)	01 Jan 2016 - 11 Apr 2016	Population	Persons Under Investigation	Ospina (2017)
2.2	95% CrI: 1.9-2.6		Next Generation Matrix		Yap Island (Federated States of Micronesia)	01 Apr 2007 - 29 Jul 2007	Population	Persons Under Investigation	Champagne (2016)
2.205	95% CrI: 1.466-3.166		Next Generation Matrix		El Salvador	2015 - 22 Jun 2016	Population	Persons Under Investigation	Shutt (2017)
2.227			Next Generation Matrix		Puerto Rico (United States)	Jan 2016 - Dec 2017	Population	General Population	Valega-Mackenzie (2023)
2.23		Other	Next Generation Matrix		Colombia	Unspecified	Unspecified	Unspecified	Li (2019)
2.28			Compartmental Model	3681000	Puerto Rico (United States)	Dec 2015 - Dec 2016	Unspecified	General Population	Biswas (2024)
2.33	95% CI: 1.97-2.97	Other, Region	Growth Rate		Rio de Janeiro (Multi-country: Brazil and Colombia)	18 Oct 2015			Villela (2017)
2.33	95% CI: 1.54-3.85		Other	574	Laranjeiras (Brazil)	Jul 2016 - Jun 2019	Other	Other	Botosso (2023)
2.337	95% CI: 2.152-2.508				Tuamotu-Gambier, French Polynesia (France)	Oct 2013 - Mar 2014	Population	General Population	Rahman (2019)
2.338	95% CI: 2.179-2.488				Tahiti, French Polynesia (France)	Oct 2013 - Mar 2014	Population	General Population	Rahman (2019)
2.37	95% CI: 2.35-2.39		Next Generation Matrix		Costa Rica	12 Apr 2016 - 24 Aug 2016	Population	General Population	Luo (2021)
2.4	95% CrI: 2-3.2		Next Generation Matrix		Tahiti, French Polynesia (France)	Oct 2013 - Apr 2014	Population	Persons Under Investigation	Champagne (2016)
2.422	95% CrI: 1.653-3.47		Next Generation Matrix		Suriname	2015 - 22 Jun 2016	Population	Persons Under Investigation	Shutt (2017)
2.45	95% CI: 1.57-3.65		Growth Rate		Rio de Janeiro (Brazil)	2002			Villela (2017)
2.498	95% CI: 2.042-3.148				Sous-le-vent Islands, French Polynesia (France)	Oct 2013 - Mar 2014	Population	General Population	Rahman (2019)
2.502			Next Generation Matrix		Brazil	06 Feb 2016 - 02 Jun 2016	Population	Persons Under Investigation	Wang (2017)
2.53	95% CI: 1.59-3.45		Compartmental Model		Rio de Janeiro (Brazil)	2015 - 2016	Population	General Population	Bastos (2018)

continued on next page

continued from previous page

Parameter value	Uncertainty	Disaggr-egation	Method for R estimation	Sample size	Location	Dates	Population sample type	Population group	Reference
2.6	95% CrI: 1.7-5.3		Compartmental Model		Marquesas Islands, French Polynesia (France)	11 Oct 2013 - 28 Mar 2014	Population	Persons Under Investigation	Kucharski (2016)
2.6	95% CrI: 2.2-3.3		Next Generation Matrix		Mo'orea, French Polynesia (France)	Oct 2013 - Apr 2014	Population	Persons Under Investigation	Champagne (2016)
2.69	95% CrI: 2.31-3.11		Growth Rate		Manaus (Brazil)	Nov 2015	Population	General Population	Giovanetti (2020)
2.7	Range: 1-4.3		Unspecified	1946	Feira de Santana (Brazil)	01 Feb 2015 - 30 Apr 2017	Population	General Population	Lourenco (2017)
2.75	95% CI: 2.53-2.98				French Polynesia (France)	2013	Unspecified	Unspecified	Sun (2018)
2.75	95% CI: 2.53-2.98		Compartmental Model		French Polynesia (France)	2013 - 2013	Population	Persons Under Investigation	Perrotta (2022)
3	95% CrI: 2.2-6.1		Compartmental Model		Tuamotu-Gambier, French Polynesia (France)	11 Oct 2013 - 28 Mar 2014	Population	Persons Under Investigation	Kucharski (2016)
3.1	95% CrI: 2.2-4.6		Compartmental Model		Australes, French Polynesia (France)	11 Oct 2013 - 28 Mar 2014	Population	Persons Under Investigation	Kucharski (2016)
3.188	95% CI: 2.61-3.676				Mo'orea, French Polynesia (France)	Oct 2013 - Mar 2014	Population	General Population	Rahman (2019)
3.199	95% CI: 3.073-3.32				Yap Island (Federated States of Micronesia)	Oct 2013 - Mar 2014	Population	General Population	Rahman (2019)
3.2			Other	1593	Colombia	01 Jan 2016 - 13 Mar 2016	Population	Persons Under Investigation	Rocklov (2016)
3.2	95% CrI: 2.4-4.1		Next Generation Matrix		Yap Island (Federated States of Micronesia)	01 Apr 2007 - 29 Jul 2007	Population	Persons Under Investigation	Champagne (2016)
3.26	Range: 2-5		Other		Colombia	31 May 2015 - 16 Apr 2016	Population	Persons Under Investigation	Majumder (2016)
3.5	95% CrI: 2.6-5.3		Compartmental Model		Tahiti, French Polynesia (France)	11 Oct 2013 - 28 Mar 2014	Population	Persons Under Investigation	Kucharski (2016)
3.62	95% CI: 3.48-3.77	Time	Other		Singapore	31 Jul 2016 - 01 Sep 2016		Persons Under Investigation	Singapore Zika Study Group (2017)
3.7			Other	198	Puerto Rico (United States)	26 Nov 2015 - 24 Feb 2016	Population	Persons Under Investigation	Rocklov (2016)
3.8	95% CI: 2.4-5.6		Other	359	Barranquilla (Colombia)	01 Oct 2015 - Nov 2015	Hospital	Persons Under Investigation	Towers (2016)
4.1	95% CrI: 3.1-5.7		Compartmental Model		Sous-le-vent Islands, French Polynesia (France)	11 Oct 2013 - 28 Mar 2014	Population	Persons Under Investigation	Kucharski (2016)
4.61	95% CI: 4.11-5.16		Other	1936	Girardot (Colombia)	19 Oct 2015 - 22 Jan 2016	Hospital	Persons Under Investigation	Rojas (2016)
4.8			Other	5618	El Salvador	01 Jan 2016 - 13 Mar 2016	Population	Persons Under Investigation	Rocklov (2016)
4.8	95% CrI: 3.2-8.4		Compartmental Model		Mo'orea, French Polynesia (France)	11 Oct 2013 - 28 Mar 2014	Population	Persons Under Investigation	Kucharski (2016)
5			Other	386	Guatemala	16 Nov 2015 - 15 Feb 2016	Population	Persons Under Investigation	Rocklov (2016)
5.36	Range: 3-10		Other		Colombia	22 Aug 2015 - 16 Apr 2016	Population	Persons Under Investigation	Majumder (2016)
5.7	95% CI: 0-11.75		Other		Saint Martin (Multi-country: France and Netherlands)	2015 - 2016			Hsieh (2017)
6	95% CI: 0.06-11.95	Region	Other		French Polynesia (France)	2013 - 2014			Hsieh (2017)
6.89	95% CI: 0-16.24		Other		Puerto Rico (United States)	2015 - 2016			Hsieh (2017)
6.93	95% CI: 5.82-8.05		Compartmental Model		Singapore (Singapore)	07 Aug 2016 - 31 Aug 2016	Population	General Population	Zhu (2022)

continued on next page

continued from previous page

Parameter value	Uncertainty	Disaggr-egation	Method for R estimation	Sample size	Location	Dates	Population sample type	Population group	Reference
7			Other	96	Ecuador	01 Jan 2016 - 13 Mar 2016	Population	Persons Under Investigation	Rocklov (2016)
7.2			Other	102	Martinique (France)	21 Dec 2015 - 21 Jan 2016	Population	Persons Under Investigation	Rocklov (2016)
22.2			Compartmental Model	1841	Medellín (Colombia)	01 Jan 2016 - 11 Apr 2016	Population	Persons Under Investigation	Ospina (2017)
56.38			Compartmental Model	1841	Cáceres (Colombia)	01 Jan 2016 - 11 Apr 2016	Population	Persons Under Investigation	Ospina (2017)
1.24 - 1.47		Method	Compartmental Model		Florida (United States)	19 Jul 2016 - 29 Sep 2016	Population	Persons Under Investigation	Tuncer (2018)
1.54 - 1.9		Method, Time	Other		Guadeloupe (France)	24 Jan 2015 - 29 Jan 2017	Community	Persons Under Investigation	Riou (2018)
1.3 - 1.62		Method, Time	Other		Martinique (France)	20 Dec 2015 - 05 Feb 2017	Community	Persons Under Investigation	Riou (2018)
1.34 - 1.57		Method, Time	Other		Saint Martin (France)	24 Jan 2015 - 19 Feb 2017	Community	Persons Under Investigation	Riou (2018)
0.4 - 2.73			Compartmental Model		Wynwood Neighborhood, Miami-Dade County, Florida (United States)	26 Jan 2016 - 05 Aug 2016	Population	General Population	Marini (2017)
2.8 - 12.5		Other	Growth Rate	108	Yap Island (Federated States of Micronesia)	2007 - 2007	Unspecified	Unspecified	Nishiura (2016)
1.5 - 3.1		Other	Growth Rate	8581	French Polynesia (France)	2013 - 2014	Unspecified	Unspecified	Nishiura (2016)
1.29 - 1.71		Method	Branching Process	44825	Centre-West Brazil (Brazil)	01 Jan 2015 - 19 Nov 2016	Population	Persons Under Investigation	Faria (2017)
1.58 - 2.48		Method	Branching Process	22373	North Brazil (Brazil)	01 Jan 2015 - 19 Nov 2016	Population	Persons Under Investigation	Faria (2017)
1.75 - 3.12		Method, Time	Branching Process	122779	Northeast Brazil (Brazil)	01 Jan 2015 - 19 Nov 2016	Population	Persons Under Investigation	Faria (2017)
1.98 - 3.85		Method	Branching Process	112689	South-East Brazil (Brazil)	01 Jan 2015 - 19 Nov 2016	Population	Persons Under Investigation	Faria (2017)
1.61 - 2.57		Method	Branching Process	4944	South Brazil (Brazil)	01 Jan 2015 - 19 Nov 2016	Population	Persons Under Investigation	Faria (2017)
2.68 - 4.57		Method	Growth Rate	6117	Cucuta (Colombia)	29 Jun 2015 - 30 Jul 2016	Hospital	Persons Under Investigation	Anaya (2017)
**Basic Reproduction Number - Sexual**									
0.005			Next Generation Matrix		Puerto Rico (United States)	Jan 2016 - Dec 2017	Population	General Population	Valega-Mackenzie (2023)
0.054			Next Generation Matrix		Brazil	06 Feb 2016 - 02 Jun 2016	Population	Persons Under Investigation	Wang (2017)
0.136	95% CI: 0.009-0.521		Compartmental Model		Multi-country: Brazil, Colombia, El Salvador	Apr 2015 - 27 Feb 2016	Population	General Population	Gao (2016)
0.19	95% CI: 0.05-0.48	Region	Ross-Macdonald Formula		Texas (United States)	Jan 2016 - Jan 2017	Population	General Population	Fox (2019)
0.2			Compartmental Model		Brazil	25 Mar 2016 - 14 Apr 2018	Population	Persons Under Investigation	Zhao (2020)
0.42	95% CI: 0.29-0.64	Method	Compartmental Model	18364	Colombia	2015 - 2016	Population	Pregnant Women	Sasmal (2018)
0.624			Next Generation Matrix		Colombia	2015 - Dec 2016	Population	General Population	Valega-Mackenzie (2023)
0.881			Compartmental Model		Colombia	2016 - 2016	Population	General Population	Aranda (2019)
1.318			Compartmental Model		Brazil	2015 - 2015	Population	General Population	Liang (2019)
1.519	95% CI: 1.508-1.531		Unspecified	10234	Costa Rica	Jan 2016 - Dec 2017	Population	General Population	Sanchez (2019)

continued on next page

continued from previous page

Parameter value	Uncertainty	Disaggr-egation	Method for R estimation	Sample size	Location	Dates	Population sample type	Population group	Reference
1.66			Next Generation Matrix		Itabuna (Brazil)	2016 - 2016	Population	General Population	Hirata (2023)
1.667	95% CrI: 0.147-4.85		Next Generation Matrix		Suriname	2015 - 22 Jun 2016	Population	Persons Under Investigation	Shutt (2017)
1.8			Next Generation Matrix		Cuiaba (Brazil)	2016 - 2016	Population	General Population	Hirata (2023)
1.87			Next Generation Matrix		Rio de Janeiro (Brazil)	2016 - 2016	Population	General Population	Hirata (2023)
1.99	95% CI: 1.97-2.01		Next Generation Matrix		Costa Rica	12 Apr 2016 - 24 Aug 2016	Population	General Population	Luo (2021)
2.1	95% CI: 1.8-2.5		Unspecified		Salvador, Bahia (Brazil)	Jan 2015 - 28 May 2016	Population	General Population	Netto (2017)
2.2	95% CI: 1.1-3.8		Branching Process	330	Reynosa (Mexico)	2017 - 2017	Population	General Population	Olson (2020)
2.459	95% CrI: 0.152-7.402		Next Generation Matrix		El Salvador	2015 - 22 Jun 2016	Population	Persons Under Investigation	Shutt (2017)
2.76			Next Generation Matrix		Goiania (Brazil)	2016 - 2016	Population	General Population	Hirata (2023)
4.554			Next Generation Matrix		El Salvador	Oct 2015 - Apr 2016	Population	General Population	Kumar (2017)
7.6	95% CI: 4.8-14	Method	Next Generation Matrix	108	Yap Main Islands (Federated States of Micronesia)	Apr 2007 - Jul 2007	Population	Persons Under Investigation	Funk (2016)
0 - 8.31		Region	Other	377525	Multi-country: Americas (n = 21)	15 Aug 2015 - 11 Jun 2016	Population	Persons Under Investigation	Ogden (2017)
0.5 - 2.5			Unspecified		Multi-country: Brazil, Colombia, French Polynesia	Aug 2013 - Jun 2016	Population	General Population	He (2017)
1.15 - 2.75		Other	Ross-Macdonald Formula		Manacapuru (Brazil)	Feb 2014 - Feb 2015			Abad-Franch (2017)
0 - 4			Unspecified		Global	2015 - 2016	Mixed	General Population	Sun (2020)
**Basic Reproduction Number - Vector-Borne**									
1.04	95% CI: 1.03-1.05		Next Generation Matrix		Costa Rica	12 Apr 2016 - 24 Aug 2016	Population	General Population	Luo (2021)
1.103			Next Generation Matrix		Colombia	2015 - Dec 2016	Population	General Population	Valega-Mackenzie (2023)
1.51	95% CI: 1.23-1.87	Method	Compartmental Model	18364	Colombia	2015 - 2016	Population	Pregnant Women	Sasmal (2018)
1.713			Compartmental Model		Brazil	25 Mar 2016 - 14 Apr 2018	Population	Persons Under Investigation	Zhao (2020)
1.96	95% CI: 0.45-6.227		Compartmental Model		Multi-country: Brazil, Colombia, El Salvador	Apr 2015 - 27 Feb 2016	Population	General Population	Gao (2016)
2.04	95% CI: 1.53-2.55		Next Generation Matrix		Brazil	Jan 2015 - Sep 2016	Population	General Population	Wang (2024a)
2.222			Next Generation Matrix		Puerto Rico (United States)	Jan 2016 - Dec 2017	Population	General Population	Valega-Mackenzie (2023)
2.475			Next Generation Matrix		Brazil	06 Feb 2016 - 02 Jun 2016	Population	Persons Under Investigation	Wang (2017)
2.96	95% CrI: 2.58-3.39		Ross-Macdonald Formula		Unspecified	Unspecified	Unspecified	Other	Armstrong (2020)
4.05	95% CrI: 3.22-5.17		Ross-Macdonald Formula		Unspecified	Unspecified	Unspecified	Other	Armstrong (2020)
4.274	95% CrI: 0.166-12.941		Next Generation Matrix		El Salvador	2015 - 22 Jun 2016	Population	Persons Under Investigation	Shutt (2017)
6.093	95% CrI: 0.6-15.738		Next Generation Matrix		Suriname	2015 - 22 Jun 2016	Population	Persons Under Investigation	Shutt (2017)
**Effective Reproduction Number**									
0.16	95% CI: 0.13-0.19		Branching Process		Florida (United States)	01 May 2016 - 23 Sep 2016	Population	General Population	Dinh (2016)

continued on next page

continued from previous page

Parameter value	Uncertainty	Disaggr-egation	Method for R estimation	Sample size	Location	Dates	Population sample type	Population group	Reference
0.24	95% CI: 0.07-0.41		Compartmental Model		Singapore	04 Sep 2016 - 30 Nov 2016	Population	General Population	Zhu (2022)
0.62	Range: 0.25-1.06		Unspecified		Feira de Santana (Brazil)	2017 - 30 Apr 2017	Population	General Population	Lourenco (2017)
1.22	95% CI: 1.19-1.24	Time	Other		Singapore	01 Sep 2016 - 24 Nov 2016		Persons Under Investigation	Singapore Zika Study Group (2017)
1.45			Compartmental Model		Suriname	Unspecified	Population	General Population	Dénes (2019)
1.47			Compartmental Model		Costa Rica	2016 - 2018	Population	General Population	Dénes (2019)
1.54	95% CI: 1.43-1.65	Method	Branching Process	11247972	Rio Grande (Brazil)	Oct 2015 - May 2016	Population	Persons Under Investigation	Zhao (2019)
1.63	Range: 1-2		Other		Colombia	31 May 2015 - 16 Apr 2016	Population	Persons Under Investigation	Majumder (2016)
1.63	95% CI: 1.58-1.68	Method	Branching Process	15203934	Bahia (Brazil)	Oct 2015 - May 2016	Population	Persons Under Investigation	Zhao (2019)
1.67	95% CI: 1.56-1.79	Method	Branching Process	3265486	Mato Grosso (Brazil)	Oct 2015 - May 2016	Population	Persons Under Investigation	Zhao (2019)
1.85	95% CI: 1.75-1.95	Method	Branching Process	9345173	Pernambuco State (Brazil)	Oct 2015 - May 2016	Population	Persons Under Investigation	Zhao (2019)
1.96	Range: 1-3		Other		Colombia	22 Aug 2015 - 16 Apr 2016	Population	Persons Under Investigation	Majumder (2016)
2.13	95% CI: 2.07-2.19	Method	Branching Process	803513	Acre (Brazil)	Oct 2015 - May 2016	Population	Persons Under Investigation	Zhao (2019)
2.2		Time	Next Generation Matrix		Northeastern (Brazil)	Jan 2015 - Nov 2016	Population	Persons Under Investigation	He (2020)
2.82	95% CI: 2.66-2.99	Method	Branching Process	11163018	Panara (Brazil)	Oct 2015 - May 2016	Population	Persons Under Investigation	Zhao (2019)
3.05	95% CI: 2.73-3.41	Method	Branching Process	3929911	Espirito Santo (Brazil)	Oct 2015 - May 2016	Population	Persons Under Investigation	Zhao (2019)
3.07	95% CI: 2.92-3.24	Method	Branching Process	6610681	Goiania City (Brazil)	Oct 2015 - May 2016	Population	Persons Under Investigation	Zhao (2019)
0.7 - 2.2		Time	Branching Process		Salvador, Bahia (Brazil)	2015 - 2015	Population	General Population	Rodriguez-Barraquer (2019)
0.882 - 0.941		Method	Compartmental Model		Brazil	25 Mar 2016 - 14 Apr 2018	Population	Persons Under Investigation	Zhao (2020)
0 - 3.1		Time	Compartmental Model	3681000	Puerto Rico (United States)	Dec 2015 - Dec 2016	Unspecified	General Population	Biswas (2024)
**Effective Reproduction Number - Sexual**									
0.05			Compartmental Model		Brazil	25 Mar 2016 - 14 Apr 2018	Population	Persons Under Investigation	Zhao (2020)
3.3	95% CI: 1.3-5.3		Growth Rate		Managua (Nicaragua)	Jun 2016 - Jun 2016	Community	Children	Gordon (2019)
3.4	95% CI: 2.4-4.7		Growth Rate		Managua (Nicaragua)	Jun 2016 - Jul 2016	Community	Children	Gordon (2019)
1.6 - 10.3		Method, Time	Other		Antioquia (Colombia)	14 Oct 2015 - 10 Apr 2016	Population	General Population	Chowell (2016)
0.85 - 1.71			Next Generation Matrix		"north-eastern" (Brazil)	Jan 2015 - Sep 2016	Unspecified	Unspecified	Yuan (2021)
**Effective Reproduction Number - Vector-Borne**									
0.857			Compartmental Model		Brazil	25 Mar 2016 - 14 Apr 2018	Population	Persons Under Investigation	Zhao (2020)
**Growth Rate (R)**									
0.076 per day	95% CI: 0.066-0.087			359	Barranquilla (Colombia)	01 Oct 2015 - Nov 2015	Hospital	Persons Under Investigation	Towers (2016)
0.1	95% CI: 0.1-0.2				Managua (Nicaragua)	Jun 2016 - Jul 2016	Community	Children	Gordon (2019)

continued on next page

continued from previous page

Parameter value	Uncertainty	Disaggr-egation	Method for R estimation	Sample size	Location	Dates	Population sample type	Population group	Reference
0.2	95% CI: 0.1-0.4				Managua (Nicaragua)	Jun 2016 - Jul 2016	Community	Children	Gordon (2019)
0.823 per week	SD: 0.053		Growth Rate		Rio de Janeiro (Brazil)	18 Oct 2015			Villela (2017)
**Sexual Transmission Contribution**									
1.4 %					"north-eastern" (Brazil)	Jan 2015 - Sep 2016	Unspecified	Unspecified	Yuan (2021)
3.5 %					Brazil	Jan 2015 - Sep 2016	Population	General Population	Wang (2024a)
15.36 %	95% CI: 12.83-17.14	Method	Compartmental Model	18364	Colombia	2015 - 2016	Population	Pregnant Women	Sasmal (2018)
23 %	95% CI: 1-47			359	Barranquilla (Colombia)	01 Oct 2015 - Nov 2015	Hospital	Persons Under Investigation	Towers (2016)
32 %					Suriname	Unspecified	Population	General Population	Dénes (2019)
48.15 %					Costa Rica	12 Apr 2016 - 24 Aug 2016	Population	General Population	Luo (2021)
54 %					Costa Rica	2016 - 2018	Population	General Population	Dénes (2019)
**Vector Transmission Contribution**									
51.85 %					Costa Rica	12 Apr 2016 - 24 Aug 2016	Population	General Population	Luo (2021)
98.6 %					"north-eastern" (Brazil)	Jan 2015 - Sep 2016	Unspecified	Unspecified	Yuan (2021)

Table B.12: Overview of extracted transmission parameters.

B.7.7 Genomic parameters

Parameter value	Uncertainty	Genome site	Sample size	Dates	Population sample type	Population group	Reference
**Evolutionary Rate**							
0.6 s/s/y 10^{-3}	95% CI: 0.3-0.8		40	1966 - 2017	Unspecified	Unspecified	Barzilai (2019)
0.8 s/s/y 10^{-3}	95% CI: 0.5-1.1		40	2013 - 2017	Unspecified	Unspecified	Barzilai (2019)
1.18 s/s/y 10^{-3}			238	Jun 2017 - Jun 2019	Population	Persons Under Investigation	Da Costa Castilho (2024)
0.98 - 1.06 s/s/y 10^{-3}		near-complete genome		1947 - Dec 2015			Faria (2016)
8.61 s/s/y 10^{-4}	HPDI 95%: 6.34-11.1	Whole genome sequence	88	Aug 2018	Unspecified	Unspecified	Liu (2019)
8.65 s/s/y 10^{-4}		Whole genome sequence	360	Unspecified	Unspecified	Unspecified	Black (2019)
9.2 s/s/y 10^{-4}	95% CrI: 7.1-12	Whole genome sequence	65	1966 - Aug 2017			Delatorre (2017)
1.09 s/s/y 10^{-3}	95% CrI: 0.77-1.43	Whole genome sequence	59	15 Dec 2015 - Mar 2017	Population	General Population	Giovanetti (2020)
1.12 s/s/y 10^{-3}	95% CrI: 0.97-1.27	Whole genome sequence	254	1966 - 01 Mar 2016	Unspecified	Persons Under Investigation	Faria (2017)
1.2 s/s/y 10^{-3}	HPDI 95%: 0.951-1.41	Whole genome sequence	39	2007 - 2016	Unspecified	Unspecified	Fajardo (2016)
1.55 s/s/y 10^{-3}	HPDI 95%: 1.06-2.05	Whole genome sequence	49	Dec 2014 - Oct 2016			Aldunate (2017)
1.57 s/s/y 10^{-3}		Whole genome sequence	139	2016			Boskova (2018)
1.76 s/s/y 10^{-3}		Whole genome sequence	67	2016			Boskova (2018)
5.71 s/s/y 10^{-3}		Whole genome sequence	269	2013 - Aug 2018	Unspecified	Unspecified	Grubaugh (2019)
6.42 s/s/y 10^{-3}		Whole genome sequence	23	Jun 2016 - 11 Oct 2016			Boskova (2018)
0.53 - 4.39 s/s/y 10^{-3}		Whole genome sequence	58	1947 - 2016			Yokoyama (2017)
**Mutation Rate**							
0.37 Mutations/site/generation (mu)	HPDI 95%: 0.298-0.448	NS5, 1st codon	108	1947 - Oct 2017	Population	Persons Under Investigation	Shao (2020)
0.16 Mutations/site/generation (mu)	HPDI 95%: 0.113-0.211	NS5, 2nd codon	108	1947 - Oct 2017	Population	Persons Under Investigation	Shao (2020)
2.471 Mutations/site/generation (mu)	HPDI 95%: 2.382-2.555	NS5, 3rd codon	108	1947 - Oct 2017	Population	Persons Under Investigation	Shao (2020)
**Substitution Rate**							
12 - 25 Mutations/year			33	1947 - 06 Feb 2016	Unspecified	Unspecified	Logan (2016)
7.95 s/s/y 10^{-4}		Whole genome sequence	214	1966 - 2023	Mixed	Persons Under Investigation	Khongwichit (2023)
8 s/s/y 10^{-4}	HPDI 95%: 6.4-9.7	Whole genome sequence	135	1947 - 2016	Unspecified	Unspecified	Ebranati (2019)
1.114 s/s/y 10^{-3}	HPDI 95%: 0.993-1.254	Whole genome sequence	182	01 Mar 2017	Mixed	Unspecified	Le Hingrat (2019)

Table B.13: Overview of extracted genomic parameters.

B.7.8 Severity

Parameter value	Uncertainty	Disaggr-egation	Naive or Adjusted	Numerator	Denominator	Population country	Dates	Population sample type	Population group	Reference
**Case fatality ratio**										
8.3 %	95% CI: 7.2-9.6		Naive	171	2063	Brazil	10 Nov 2015 - 15 Oct 2016	Hospital	Other	Cunha (2017)
10.5 %	95% CI: 9.5-11.7		Unspecified	313	2974	Brazil	10 Nov 2015 - 15 Oct 2016	Hospital	Other	Cunha (2017)
38 %			Naive	6	16	Rio de Janeiro (Brazil)	Jan 2016 - Oct 2016	Hospital	Persons Under Investigation	Pereira Jr (2019)
8.4 %			Unspecified	35	418	Colombia	Jul 2015 - Oct 2017	Population	Persons Under Investigation	Charniga (2021a)
0.04 %			Unspecified		5161	Dominican Republic	2016 - 2016	Population	General Population	Petrone (2021)
%				2	87	Guadeloupe, Martinique (France)	06 Jan 2016	Hospital	Persons Under Investigation	Lannuzel (2019)
**Proportion of symptomatic cases**										
27 %	95% CrI: 15-37	Method				Yap Island (Federated States of Micronesia)	2007 - 2007	Unspecified	Unspecified	Mitchell (2019)
23.2 %				133	573	French Guiana (France)	01 Feb 2016 - 01 Jun 2016	Population	Pregnant Women	Flamand (2017)
44 %	95% CrI: 26-66	Method				French Polynesia (France)	2013 - 2014	Unspecified	Unspecified	Mitchell (2019)
44 %	95% CI: 26-69		Naive	8	18	Society Islands, French Polynesia (France)	Feb 2014 - Mar 2014	Population	General Population	Aubry (2017)
47 %	95% CI: 40-55		Naive	73	154	Society Islands, French Polynesia (France)	Sep 2015 - Nov 2015	Population	General Population	Aubry (2017)
55 %	95% CI: 34-75		Naive	12	22	Tuamotu Islands, French Polynesia (France)	Feb 2014 - Mar 2014	Population	General Population	Aubry (2017)
57 %	95% CI: 39-75		Naive	16	28	Marquesas Islands, French Polynesia (France)	Feb 2014 - Mar 2014	Population	General Population	Aubry (2017)
66 %	95% CI: 48-83		Naive	19	29	Austral–Gambier Islands, French Polynesia (France)	Feb 2014 - Mar 2014	Population	General Population	Aubry (2017)
71 %	95% CI: 66-76		Naive	221	312	Society Islands, French Polynesia (France)	May 2014 - Jun 2014	School	Children	Aubry (2017)
24 %			Naive	7	29	Congenital Zika Program at Children's National (CZPCN) in Washington DC (United States)	Jan 2016 - Jun 2018	Hospital	Pregnant Women	Mulkey (2021)
50 %	95% CrI: 34-92	Method				Puerto Rico (United States)	2016 - 2016	Unspecified	Unspecified	Mitchell (2019)

Table B.14: Overview of extracted severity parameters.

B.7.9 Relative contributions

Parameter value	Uncertainty	Population country	Dates	Population sample type	Population group	Reference
**Relative contribution - sexual**						
1.4 %		"north-eastern" (Brazil)	Jan 2015 - Sep 2016	Unspecified	Unspecified	Yuan (2021)
3.5 %		Brazil	Jan 2015 - Sep 2016	Population	General Population	Wang (2024a)
15.36 %	95% CI: 12.83-17.14	Colombia	2015 - 2016	Population	Pregnant Women	Sasmal (2018)
23 %	95% CI: 1-47	Barranquilla (Colombia)	01 Oct 2015 - Nov 2015	Hospital	Persons Under Investigation	Towers (2016)
48.15 %		Costa Rica	12 Apr 2016 - 24 Aug 2016	Population	General Population	Luo (2021)
54 %		Costa Rica	2016 - 2018	Population	General Population	Dénes (2019)
32 %		Suriname	Unspecified	Population	General Population	Dénes (2019)
**Relative contribution - vector-borne**						
98.6 %		"north-eastern" (Brazil)	Jan 2015 - Sep 2016	Unspecified	Unspecified	Yuan (2021)
51.85 %		Costa Rica	12 Apr 2016 - 24 Aug 2016	Population	General Population	Luo (2021)

Table B.15: Overview of extracted relative contribution parameters.

B.7.10 Adverse birth outcomes

Parameter value	Uncertainty	Naive or Adjusted	Numerator	Denom-inator	Population country	Dates	Population sample type	Reference
**Zika congenital syndrome (microcephaly) proportion**								
0 %		Unspecified	0	16	Antigua and Barbuda	May 2015 - Jan 2018	Population	Morris (2020)
3.13 %		Unspecified	1	32	Barbados	May 2015 - Jan 2018	Population	Morris (2020)
0 %		Unspecified	0	1	Belize	May 2015 - Jan 2018	Population	Morris (2020)
7.41 %		Unspecified	14	189	Bolivia	May 2015 - Jan 2018	Population	Morris (2020)
0 %		Naive	0	6	São Paulo (Brazil)	01 Jan 2016 - Sep 2016	Hospital	Tozetto-Mendoza (2019)
0.006 %		Unspecified			Tocantins (Brazil)	Jul 2015 - Dec 2016	Hospital	Rodrigues (2020)
0.49 - 2.1 %		Unspecified			Northeast Brazil (Brazil)	15 Nov 2015 - 02 Apr 2016	Other	Alfaro-Murillo (2016)
1.8 %	95% CI: 0.2-10	Naive	1	38	Pernambuco (Recife) (Brazil)	Dec 2015 - Jun 2017	Hospital	Ximenes (2021)
%		Unspecified	15	780	Brazil	2016 - 2016	Hospital	Martins (2019)
2.2 %	95% CI: 0.7-6.6	Naive	3	86	Pernambuco (Recife) (Brazil)	Dec 2015 - Jun 2017	Hospital	Ximenes (2021)
2.5 %	95% CI: 1.2-5.2	Naive	7	278	Pernambuco (Recife) (Brazil)	Dec 2015 - Jun 2017	Hospital	Ximenes (2021)
3.4 %		Unspecified	4	117	Rio de Janeiro state (Brazil)	01 Jan 2016 - 31 Jul 2016	Hospital	Brasil (2016)
3.6 %		Naive	3	83	Jundiai Hospital Medical school (Brazil)	01 Mar 2016 - 30 Jun 2019	Hospital	Gazeta (2021)
3.7 %		Unspecified	8	216	Rio de Janeiro (Brazil)	Dec 2015 - Dec 2016	Hospital	Brasil (2020)
3.8 %	95% CI: 1.8-7.8	Naive	7	154	Pernambuco (Recife) (Brazil)	Dec 2015 - Jun 2017	Hospital	Ximenes (2021)
%		Unspecified	2	48	São Paulo state (Brazil)	01 Mar 2016 - 23 Aug 2017	Hospital	Sanchez Clemente (2020)
4.5 %		Unspecified	2	44	Jundiai, São Paulo (Brazil)	01 Mar 2016 - 23 Aug 2017	Hospital	Clemente (2020)
4.8 %		Unspecified	5	109	Tangará da Serra (Brazil)	01 Jan 2016 - 31 Dec 2016	Other	Herrero Da Silva (2022)
5 %		Naive	2	43	Private hospital Salvador, Bahia (Brazil)	01 Jan 2015 - 31 Jan 2017	Hospital	Aromolaran (2022)
5.2 %		Unspecified	4	77	Manaus, Amazonas (Brazil)	Mar 2016 - Jun 2018	Hospital	Abtibol-Bernardino (2022)
7 %	95% CI: 1.5-19.1	Unspecified	3	43	Central-West (Brazil)	Jan 2017 - Apr 2019	Hospital	Rosado (2023)
7.1 %		Unspecified	7	98	Rio de Janeiro (Brazil)	2016 - 2016	Other	De Melo Espindola (2021)
%		Naive	7	92	Rio de Janeiro (Brazil)	01 Sep 2015 - 31 May 2016	Other	Pereira (2018)
8.7 %	95% CrI: 7.13-11.39	Unspecified			Brazil	11 Aug 2015 - 31 Dec 2018	Population	Moore (2020)
%		Naive	4	40	Mato Grosso do Sul (Brazil)	Nov 2017 - Oct 2018	Population	De Sousa (2020)
11.33 %		Unspecified	2952	26066	Brazil	May 2015 - Jan 2018	Population	Morris (2020)
%		Naive	6	51	Mato Grosso do Sul (Brazil)	Nov 2017 - Oct 2018	Population	De Sousa (2020)

continued on next page

continued from previous page

Parameter value	Uncertainty	Naive or Adjusted	Numerator	Denom-inator	Population country	Dates	Population sample type	Reference
%		Unspecified	76	602	Brazil	19 Nov 2015 - 27 Feb 2016	Population	França (2016)
%		Naive	23	120	Mato Grosso do Sul (Brazil)	Nov 2017 - Oct 2018	Population	De Sousa (2020)
24.7 %		Naive	53	219	Rio de Janeiro (Brazil)	Sep 2015 - Jun 2017	Hospital	Cranston (2020)
33 %		Naive	42	127	Public hospital Salvador, Bahia (Brazil)	01 Jan 2015 - 31 Jan 2017	Hospital	Aromolaran (2022)
38.5 %		Unspecified	104	104	Salvador, Bahia (Brazil)	Nov 2015 - Feb 2016	Hospital	Robbiani (2019)
%		Naive	12	28	Mato Grosso do Sul (Brazil)	Nov 2017 - Oct 2018	Population	De Sousa (2020)
49 %		Naive	54	110	Rio de Janeiro (Brazil)	01 Mar 2016 - 30 Jun 2017	Hospital	Pool (2019)
70.7 %		Naive	53	75	Campina Grande, Paraíba (Brazil)	Mar 2016 - Dec 2018	Hospital	Sampaio (2021)
%		Unspecified	0	30	Colombia	Oct 2015 - Jan 2017	Hospital	Gutierrez-Sanchez (2022)
0 %		Naive	0	616	Colombia	09 Aug 2015 - 02 Apr 2016	Other	Pacheco (2020)
0 %		Unspecified	0	93	Colombia	Feb 2017 - Mar 2019	Population	Tannis (2024)
1.24 %		Unspecified	248	19993	Colombia	May 2015 - Jan 2018	Population	Morris (2020)
%		Unspecified	1	50	Valle del Cauca (Colombia)	Feb 2018 - Apr 2020	Population	Calle-Giraldo (2019)
%		Unspecified	3	75	Valle del Cauca (Colombia)	Jan 2017 - Mar 2019	Population	Calle-Giraldo (2019)
4.5 %		Naive	7	154	Valle del Cauca (Colombia)	Nov 2015 - Jan 2017	Population	Calle-Giraldo (2019)
8.8 %		Unspecified	9	102	Colombia	Oct 2015 - Jan 2017	Hospital	Gutierrez-Sanchez (2022)
%		Unspecified	5	53	Colombia	Oct 2015 - Jan 2017	Hospital	Gutierrez-Sanchez (2022)
%		Unspecified	3	29	Valle del Cauca (Colombia)	Dec 2016 - Feb 2018	Population	Calle-Giraldo (2019)
%		Unspecified	9	25	Colombia	Oct 2015 - Jan 2017	Hospital	Gutierrez-Sanchez (2022)
9.05 %		Unspecified	19	210	Costa Rica	May 2015 - Jan 2018	Population	Morris (2020)
0 %		Unspecified	0	30	Curaçao	May 2015 - Jan 2018	Population	Morris (2020)
0 %		Unspecified	0	13	Dominica	May 2015 - Jan 2018	Population	Morris (2020)
0 %		Unspecified	0	296	Dominican Republic	Jan 2016 - Apr 2017	Population	Peña (2019)
8.8 %		Unspecified	85	966	Dominican Republic	May 2015 - Jan 2018	Population	Morris (2020)
11 %		Unspecified	87	800	Dominican Republic	2016	Hospital	Pimintel (2021)
1.54 %		Unspecified	14	912	Ecuador	May 2015 - Jan 2018	Population	Morris (2020)
1.02 %		Unspecified	4	391	El Salvador	May 2015 - Jan 2018	Population	Morris (2020)
0 %		Naive	0	125	French Guiana (France)	15 Jul 2016 - 01 Jan 2016	Hospital	Pomar (2017)
0 %		Unspecified	0	11	Saint Barthelemy (France)	May 2015 - Jan 2018	Population	Morris (2020)
0.05 %		Unspecified	1	2211	French Guiana (France)	May 2015 - Jan 2018	Population	Morris (2020)
0.6 %		Unspecified	5	830	Martinique (France)	May 2015 - Jan 2018	Population	Morris (2020)

continued on next page

continued from previous page

Parameter value	Uncertainty	Naive or Adjusted	Numerator	Denom-inator	Population country	Dates	Population sample type	Reference
0.61 %		Unspecified	5	815	Guadeloupe (France)	May 2015 - Jan 2018	Population	Morris (2020)
1.7 %		Naive	5	301	French Guiana (France)	15 Jul 2016 - 01 Jan 2016	Hospital	Pomar (2017)
%		Naive	3	129	western French Guiana (France)	Jan 2016 - Sep 2016	Hospital	Hcini (2021)
2.5 %		Naive	2	80	French Guiana (France)	15 Jul 2016 - 01 Jan 2016	Hospital	Pomar (2017)
3.1 %		Naive	3	96	French Guiana (France)	15 Jul 2016 - 01 Jan 2016	Hospital	Pomar (2017)
3.2 %		Unspecified	8	252	Martinique, Guadeloupe, French Guiana (France)	Mar 2016 - 27 Apr 2017	Population	Hoen (2018)
4.4 %		Unspecified	5	114	Martinique, Guadeloupe, French Guiana (France)	Mar 2016 - 27 Apr 2017	Population	Hoen (2018)
%		Naive	17	320	Guadeloupe, Martinique, French Guiana (France)	02 Mar 2016 - 24 Nov 2016	Other	Grant (2022a)
5.8 %		Unspecified	32	555	Martinique, Guadeloupe, French Guiana (France)	Mar 2016 - 27 Apr 2017	Population	Hoen (2018)
10 %		Naive	27	273	French Guiana (France)	Jan 2016 - Jul 2016	Hospital	Pomar (2018)
10.1 %		Unspecified	19	189	Martinique, Guadeloupe, French Guiana (France)	Mar 2016 - 27 Apr 2017	Population	Hoen (2018)
9.9 %		Unspecified	140	1414	Guatemala	May 2015 - Jan 2018	Population	Morris (2020)
4.55 %		Unspecified	1	22	Haiti	May 2015 - Jan 2018	Population	Morris (2020)
1.17 %		Unspecified	8	681	Honduras	May 2015 - Jan 2018	Population	Morris (2020)
33.3 %	95% CI: 4.3-77.7	Unspecified	2	6	Alonso Suazo Health Center (Honduras)	Jul 2016 - 31 Dec 2016	Hospital	Alger (2021)
0 %		Unspecified	0	712	Jamaica	May 2015 - Jan 2018	Population	Morris (2020)
%		Naive	2	166	Mombasa (Kenya)	Oct 2017 - Jul 2019	Hospital	Osoro (2022)
0.35 %		Unspecified	20	5667	Mexico	May 2015 - Jan 2018	Population	Morris (2020)
0.07 %	95% CrI: 0.01-0.19	Unspecified			Nicaragua	27 Jan 2016 - 21 Nov 2016	Population	Moore (2020)
0.18 %		Unspecified	2	1117	Nicaragua	May 2015 - Jan 2018	Population	Morris (2020)
8.02 %		Unspecified	17	212	Panama	May 2015 - Jan 2018	Population	Morris (2020)
6.45 %		Unspecified	2	31	Paraguay	May 2015 - Jan 2018	Population	Morris (2020)
0 %		Unspecified	0	279	Peru	May 2015 - Jan 2018	Population	Morris (2020)
0 %		Unspecified	0	10	Sint Maarten (Dutch part)	May 2015 - Jan 2018	Population	Morris (2020)
15.8 %	95% CI: 3.4-39.6	Naive	3	19	Spain	Jan 2016 - Feb 2019	Travel	Soriano-Arandes (2020)
%		Naive	2	12	Barcelona (Spain)	Jan 2016 - Apr 2019	Population	Martinez-Arias (2023)
		Unspecified	2	11	Barcelona (Spain)	May 2016 - Dec 2021	Other	Romaní (2022)

continued on next page

continued from previous page

Parameter value	Uncertainty	Naive or Adjusted	Numerator	Denom- inator	Population country	Dates	Population sample type	Reference
2.08 %		Unspecified	1	48	St. Martin (French part)	May 2015 - Jan 2018	Population	Morris (2020)
0 %		Unspecified	0	3	St. Vincent and the Grenadines	May 2015 - Jan 2018	Population	Morris (2020)
3.67 %		Unspecified	17	463	Trinidad and Tobago	May 2015 - Jan 2018	Population	Morris (2020)
		Unspecified	0	70	New York (United States)	Mar 2016 - Apr 2016	Hospital	Merriam (2020)
1.16 %		Unspecified	47	4047	Puerto Rico (United States)	May 2015 - Jan 2018	Population	Morris (2020)
2.6 %		Unspecified	3	114	Ponce, Puerto Rico (United States)	01 May 2017 - 28 Feb 2020	Population	Alvarado-Domenech (2022)
4 %		Unspecified	18	442	continental United States and Hawaii (United States)	15 Jan 2016 - 22 Sep 2016	Population	Honein (2017)
4.5 %		Unspecified	18	404	New York City (United States)	Jan 2016 - Dec 2017	Hospital	Lee (2020)
5.26 %		Unspecified	5	95	Miami, Florida (United States)	01 Jan 2016 - 31 Dec 2017	Population	Walker (2020)
0 %		Unspecified	0	84	Saint Lucia	May 2015 - Jan 2018	Population	Morris (2020)
0 %		Unspecified	0	3463	Venezuela	May 2015 - Jan 2018	Population	Morris (2020)
%		Unspecified	3	21	Ho Chi Minh City (Vietnam)	Mar 2016 - Nov 2017	Hospital	Grant (2021)
0 %		Unspecified	0	286	Virgin Islands (U.S.)	May 2015 - Jan 2018	Population	Morris (2020)

Table B.16: Overview of extracted CZS proportion and pregnancy loss proportion parameters.

C PRISMA 2020 Checklists

Section & Topic	Item #	Checklist item	Reported (Yes/No)
**Title**			
Title	1	Identify the report as a systematic review.	Yes
**Background**			
Objectives	2	Provide an explicit statement of the main objective(s) or question(s) the review addresses.	Yes
**Methods**			
Eligibility criteria	3	Specify the inclusion and exclusion criteria for the review.	Yes
Information sources	4	Specify the information sources (e.g. databases, registers) used to identify studies and the date when each was last searched.	Yes
Risk of bias	5	Specify the methods used to assess risk of bias in the included studies.	Yes
Synthesis of results	6	Specify the methods used to present and synthesise results.	Yes
**Results**			
Included studies	7	Give the total number of included studies and participants and summarise relevant characteristics of studies.	Yes
Synthesis of results	8	Present results for main outcomes, preferably indicating the number of included studies and participants for each. If meta-analysis was done, report the summary estimate and confidence/credible interval. If comparing groups, indicate the direction of the effect (i.e. which group is favoured).	Yes
**Discussion**			
Limitations of evidence	9	Provide a brief summary of the limitations of the evidence included in the review (e.g. study risk of bias, inconsistency and imprecision).	Yes
Interpretation	10	Provide a general interpretation of the results and important implications.	Yes
**Other**			
Funding	11	Specify the primary source of funding for the review.	Yes
Registration	12	Provide the register name and registration number.	Yes

Table C.17: PRISMA 2020 Abstracts Checklist. ([14])

Section & Topic	Item #	Checklist item	Location where item is reported
**Title**			
Title	1	Identify the report as a systematic review.	page 1
**Abstract**			
Abstract	2	See the PRISMA 2020 for Abstracts checklist.	Table C.17
**Introduction**			
Rationale	3	Describe the rationale for the review in the context of existing knowledge.	page 3
Objectives	4	Provide an explicit statement of the objective(s) or question(s) the review addresses.	page 3
**Methods**			
Eligibility criteria	5	Specify the inclusion and exclusion criteria for the review and how studies were grouped for the syntheses.	page 8

Table C.18: PRISMA 2020 Checklist. ([14])

Section & Topic	Item #	Checklist item	Location where item is reported
Information sources	6	Specify all databases, registers, websites, organisations, reference lists and other sources searched or consulted to identify studies. Specify the date when each source was last searched or consulted.	page 8
Search strategy	7	Present the full search strategies for all databases, registers and websites, including any filters and limits used.	page 8, Figure 1, Section appendix A.1
Selection process	8	Specify the methods used to decide whether a study met the inclusion criteria of the review, including how many reviewers screened each record and each report retrieved, whether they worked independently, and if applicable, details of automation tools used in the process.	page 8, Section A.1
Data collection process	9	Specify the methods used to collect data from reports, including how many reviewers collected data from each report, whether they worked independently, any processes for obtaining or confirming data from study investigators, and if applicable, details of automation tools used in the process.	page 8/9, Section A.1
Data items	10a	List and define all outcomes for which data were sought. Specify whether all results that were compatible with each outcome domain in each study were sought (e.g. for all measures, time points, analyses), and if not, the methods used to decide which results to collect.	page 8/9, Sections A.1 and A.2
	10b	List and define all other variables for which data were sought (e.g. participant and intervention characteristics, funding sources). Describe any assumptions made about any missing or unclear information.	page 8/9, Sections A.1 and A.2
Study risk of bias assessment	11	Specify the methods used to assess risk of bias in the included studies, including details of the tool(s) used, how many reviewers assessed each study and whether they worked independently, and if applicable, details of automation tools used in the process.	page 8/9
Effect measures	12	Specify for each outcome the effect measure(s) (e.g. risk ratio, mean difference) used in the synthesis or presentation of results.	page 8/9 Section A.2
Synthesis methods	13a	Describe the processes used to decide which studies were eligible for each synthesis (e.g. tabulating the study intervention characteristics and comparing against the planned groups for each synthesis (item #5)).	page 8/9, Sections A.2 and A.3
	13b	Describe any methods required to prepare the data for presentation or synthesis, such as handling of missing summary statistics, or data conversions.	page 9, Sections A.2 and A.3
	13c	Describe any methods used to tabulate or visually display results of individual studies and syntheses.	page 9, Sections A.2 and A.3
	13d	Describe any methods used to synthesize results and provide a rationale for the choice(s). If meta-analysis was performed, describe the model(s), method(s) to identify the presence and extent of statistical heterogeneity, and software package(s) used.	page 9, Sections A.2, A.3, and A.3.1
	13e	Describe any methods used to explore possible causes of heterogeneity among study results (e.g. subgroup analysis, meta-regression).	page 9
	13f	Describe any sensitivity analyses conducted to assess robustness of the synthesized results.	-
Reporting bias assessment	14	Describe any methods used to assess risk of bias due to missing results in a synthesis (arising from reporting biases).	page 9, Figures B.29, B.30, B.30

Table C.18: PRISMA 2020 Checklist. ([14])

Section & Topic	Item #	Checklist item	Location where item is reported
Certainty assessment	15	Describe any methods used to assess certainty (or confidence) in the body of evidence for an outcome.	page 8/9, Section A
**Results**			
Study selection	16a	Describe the results of the search and selection process, from the number of records identified in the search to the number of studies included in the review, ideally using a flow diagram.	page 3, Figure 1
	16b	Cite studies that might appear to meet the inclusion criteria, but which were excluded, and explain why they were excluded.	Figure 1 and see epireview for list of excluded studies
Study characteristics	17	Cite each included study and present its characteristics.	pages 3-6, Section A.1, and list of included studies on epireview
Risk of bias in studies	18	Present assessments of risk of bias for each included study.	page 6, list of included studies on epireview, Section A.1
Results of individual studies	19	For all outcomes, present, for each study: (a) summary statistics for each group (where appropriate) and (b) an effect estimate and its precision (e.g. confidence/credible interval), ideally using structured tables or plots.	pages 3-6, Supplementary information pages 46-118
Results of syntheses	20a	For each synthesis, briefly summarise the characteristics and risk of bias among contributing studies.	page 5/6, Sections B.5 and B.6
	20b	Present results of all statistical syntheses conducted. If meta-analysis was done, present for each the summary estimate and its precision (e.g. confidence/credible interval) and measures of statistical heterogeneity. If comparing groups, describe the direction of the effect.	page 3-6, Sections B.5 and B.6
	20c	Present results of all investigations of possible causes of heterogeneity among study results.	page 3-6
	20d	Present results of all sensitivity analyses conducted to assess the robustness of the synthesized results.	page 3-6
Reporting biases	21	Present assessments of risk of bias due to missing results (arising from reporting biases) for each synthesis assessed.	pages 3-6
Certainty of evidence	22	Present assessments of certainty (or confidence) in the body of evidence for each outcome assessed.	pages 3-6
**Discussion**			

Table C.18: PRISMA 2020 Checklist. ([14])

Section & Topic	Item #	Checklist item	Location where item is reported
Discussion	23a	Provide a general interpretation of the results in the context of other evidence.	page 8-10
	23b	Discuss any limitations of the evidence included in the review.	page 6-8
	23c	Discuss any limitations of the review processes used.	page 6-8
	23d	Discuss implications of the results for practice, policy, and future research.	page 6-8
**Other Information**			
Registration and protocol	24a	Provide registration information for the review, including register name and registration number, or state that the review was not registered.	page 8
	24b	Indicate where the review protocol can be accessed, or state that a protocol was not prepared.	page 8
	24c	Describe and explain any amendments to information provided at registration or in the protocol.	-
Support	25	Describe sources of financial or non-financial support for the review, and the role of the funders or sponsors in the review.	page 10
Competing interests	26	Declare any competing interests of review authors.	page 10
Availability of data, code and other materials	27	Report which of the following are publicly available and where they can be found: template data collection forms; data extracted from included studies; data used for all analyses; analytic code; any other materials used in the review.	page 10

Table C.18: PRISMA 2020 Checklist. ([14])

References

1. Mehand MS, Al-Shorbaji F, Millett P, and Murgue B. The WHO R&D Blueprint: 2018 review of emerging infectious diseases requiring urgent research and development efforts. Antiviral research 2018; 159:63
2. Cuomo-Dannenburg G, McCain K, McCabe R, Unwin HJT, Doohan P, Nash RK, Hicks JT, Charniga K, Geismar C, Lambert B, Nikitin D, Skarp J, Wardle J, Kont M, Bhatia S, Imai N, Elsland Sv, Cori A, Morgenstern C, Morris A, Forna A, Dighe A, Cori A, Hamlet A, Lambert B, Whittaker C, Morgenstern C, Geismar C, Nikitin D, Jorgensen D, Knock E, Unwin E, Cuomo-Dannenburg G, Thompson H, Routledge I, Skarp J, Hicks J, Fraser K, Charniga K, McCain K, Geidelberg L, Cattarino L, Kont M, Baguelin M, Imai N, Moghaddas N, Doohan P, Nash R, McCabe R, Elsland Sv, Bhatia S, Radhakrishnan S, Perez ZC, and Wardle J. Marburg virus disease outbreaks, mathematical models, and disease parameters: a systematic review. The Lancet Infectious Diseases. 2024 May 1; 24. Publisher: Elsevier:e307–e317. DOI: 10.1016/S1473-3099(23)00515-7. Available from: https://www.thelancet.com/journals/laninf/article/PIIS1473-3099(23)00515-7/abstract [Accessed on: 2024 Nov 25]
3. Nash RK, Bhatia S, Morgenstern C, Doohan P, Jorgensen D, McCain K, McCabe R, Nikitin D, Forna A, Cuomo-Dannenburg G, Hicks JT, Sheppard RJ, Naidoo T, Elsland Sv, Geismar C, Rawson T, Leuba SI, Wardle J, Routledge I, Fraser K, Imai-Eaton N, Cori A, and Unwin HJT. Ebola virus disease mathematical models and epidemiological parameters: a systematic review. The Lancet Infectious Diseases. 2024 Aug 7; 0. Publisher: Elsevier. DOI: 10.1016/S1473-3099(24)00374-8. Available from: https://www.thelancet.com/journals/laninf/article/PIIS1473-3099(24)00374-8/abstract [Accessed on: 2024 Nov 25]
4. Doohan P, Jorgensen D, Naidoo TM, McCain K, Hicks JT, McCabe R, Bhatia S, Charniga K, Cuomo-Dannenburg G, Hamlet A, Nash RK, Nikitin D, Rawson T, Sheppard RJ, Unwin HJT, Elsland Sv, Cori A, Morgenstern C, Imai-Eaton N, Morris A, Forna A, Dighe A, Vicco A, Hartner AM, Cori A, Hamlet A, Lambert B, Daniels BC, Whittaker C, Morgenstern C, Santoni C, Geismar C, Nikitin D, Jorgensen D, Dee D, Knock E, Unwin E, Cuomo-Dannenburg G, Thompson H, Dorigatti I, Routledge I, Wardle J, Skarp J, Hicks J, Parchani K, Fraser K, Charniga K, McCain K, Drake K, Geidelberg L, Cattarino L, Kusumgar M, Kont M, Baguelin M, Imai-Eaton N, Guzman PP, Doohan P, Lietar P, Christen P, Nash R, Fitzjohn R, Sheppard R, Johnson R, McCabe R, Elsland Sv, Bhatia S, Leuba S, Ruybal-Pesantez S, Radhakrishnan S, Rawson T, Naidoo T, and Perez ZC. Lassa fever outbreaks, mathematical models, and disease parameters: a systematic review and meta-analysis. The Lancet Global Health. 2024 Dec 1; 12. Publisher: Elsevier:e1962–e1972. DOI: 10.1016/S2214-109X(24)00379-6. Available from: https://www.thelancet.com/journals/langlo/article/PIIS2214-109X(24)00379-6/fulltext [Accessed on: 2024 Nov 25]
5. Morgenstern C, Rawson T, Routledge I, Kont M, Imai-Eaton N, Skarp J, Doohan P, McCain K, Johnson R, Unwin HJT, Naidoo T, Dee DP, Parchani K, Daniels BNC, Vicco A, Drake KO, Christen P, Sheppard RJ, Leuba SI, Hicks JT, McCabe R, Nash RK, Santoni CN, Group PER, Cuomo-Dannenburg G, Elsland Sv, Bhatia S, and Cori A. Severe acute respiratory syndrome (SARS) mathematical models and disease parameters: a systematic review and meta-analysis. Pages: 2024.08.13.24311934. 2024 Aug 14. DOI: 10.1101/2024.08.13.24311934. Available from: https://www.medrxiv.org/content/10.1101/2024.08.13.24311934v1 [Accessed on: 2024 Nov 25]
6. Balduzzi S, Rücker G, and Schwarzer G. How to perform a meta-analysis with R: a practical tutorial. Evidence-Based Mental Health. 2019 :153–60
7. Robbiani DF, Olsen PC, Costa F, Wang Q, Oliveira TY, Nery N, Aromolaran A, Rosário MS do, Sacramento GA, Cruz JS, Khouri R, Wunder EA, Mattos A, Paula Freitas B de, Sarno M, Archanjo G, Daltro D, Carvalho GBS, Pimentel K, Siqueira IC de, Almeida JRM de, Henriques DF, Lima JA, Vasconcelos PFC, Schaefer-Babajew D, Azzopardi SA, Bozzacco L, Gazumyan A, Belfort R, Alcântara AP, Carvalho G, Moreira L, Araujo K, Reis MG, Keesler RI, Coffey LL, Tisoncik-Go J, Gale M, Rajagopal L, Adams Waldorf KM, Dudley DM, Simmons HA, Mejia A, O'Connor DH, Steinbach RJ, Haese N, Smith J, Lewis A, Colgin L, Roberts V, Frias A, Kelleher M, Hirsch A, Streblow DN, Rice CM, MacDonald MR, Almeida ARP de, Van Rompay KKA, Ko AI, and Nussenzweig MC. Risk of Zika microcephaly correlates with features of maternal

antibodies. The Journal of Experimental Medicine. 2019 Oct 7; 216:2302–15. DOI: 10.1084/jem.20191061

8. Harrer M, Cuijpers P, A FT, and Ebert DD. Doing Meta-Analysis With R: A Hands-On Guide. 1st. Boca Raton, FL and London: Chapman & Hall/CRC Press, 2021
9. Singapore Zika Study Group. Outbreak of Zika virus infection in Singapore: an epidemiological, entomological, virological, and clinical analysis. The Lancet Infectious Diseases. 2017 Aug; 17:813–21. DOI: 10.1016/S1473-3099(17)30249-9
10. Riou J, Poletto C, and Boëlle PY. A comparative analysis of Chikungunya and Zika transmission. Epidemics. 2017 Jun; 19:43–52. DOI: 10.1016/j.epidem.2017.01.001
11. Dantas E, Tosin M, and Cunha Jr A. Calibration of a SEIR–SEI epidemic model to describe the Zika virus outbreak in Brazil. Applied Mathematics and Computation. 2018 Dec 1; 338:249–59. DOI: 10.1016/j.amc.2018.06.024. Available from: https://www.sciencedirect.com/science/article/pii/S0096300318305125 [Accessed on: 2025 Jun 17]
12. Tuncer N, Marctheva M, LaBarre B, and Payoute S. Structural and Practical Identifiability Analysis of Zika Epidemiological Models. Bulletin of Mathematical Biology. 2018 Aug; 80:2209–41. DOI: 10.1007/s11538-018-0453-z
13. Rahman M, Bekele-Maxwell K, Cates LL, Banks HT, and Vaidya NK. Modeling Zika Virus Transmission Dynamics: Parameter Estimates, Disease Characteristics, and Prevention. Scientific Reports. 2019 Jul 22; 9. Publisher: Nature Publishing Group:10575. DOI: 10.1038/s41598-019-46218-4. Available from: https://www.nature.com/articles/s41598-019-46218-4 [Accessed on: 2025 Jun 17]
14. Page MJ, McKenzie JE, Bossuyt PM, Boutron I, Hoffmann TC, Mulrow CD, Shamseer L, Tetzlaff JM, Akl EA, Brennan SE, Chou R, Glanville J, Grimshaw JM, Hróbjartsson A, Lalu MM, Li T, Loder EW, Mayo-Wilson E, McDonald S, McGuinness LA, Stewart LA, Thomas J, Tricco AC, Welch VA, Whiting P, and Moher D. The PRISMA 2020 statement: an updated guideline for reporting systematic reviews. BMJ. 2021 Mar 29; 372. Publisher: British Medical Journal Publishing Group Section: Research Methods & Reporting:n71. DOI: 10.1136/bmj.n71. Available from: https://www.bmj.com/content/372/bmj.n71 [Accessed on: 2025 May 15]
