# Supplementary material for: A systematic review and meta-analysis of Zika virus epidemiology
Source: Nat Health. 2026 Feb 13;1(3):355–67. doi: 10.1038/s44360-025-00051-4 (PMC12965883; doi:10.1038/s44360-025-00051-4)
Supplement: Supplementary file 2 — Reporting Summary [file 44360_2025_51_MOESM2_ESM.pdf]

nature portfolio

Corresponding author(s): Kelly McCain, Anna Vicco, Ilaria Dorigatti

Last updated by author(s): Nov 20, 2025

Reporting Summary

Nature Portfolio wishes to improve the reproducibility of the work that we publish. This form provides structure for consistency and transparency in reporting. For further information on Nature Portfolio policies, see our Editorial Policies and the Editorial Policy Checklist.

Statistics

For all statistical analyses, confirm that the following items are present in the figure legend, table legend, main text, or Methods section.

n/a	Confirmed	
☐	☒	The exact sample size (*n*) for each experimental group/condition, given as a discrete number and unit of measurement
☒	☐	A statement on whether measurements were taken from distinct samples or whether the same sample was measured repeatedly
☐	☒	The statistical test(s) used AND whether they are one- or two-sided<br>*Only common tests should be described solely by name; describe more complex techniques in the Methods section.*
☒	☐	A description of all covariates tested
☐	☒	A description of any assumptions or corrections, such as tests of normality and adjustment for multiple comparisons
☐	☒	A full description of the statistical parameters including central tendency (e.g. means) or other basic estimates (e.g. regression coefficient) AND variation (e.g. standard deviation) or associated estimates of uncertainty (e.g. confidence intervals)
☒	☐	For null hypothesis testing, the test statistic (e.g. *F*, *t*, *r*) with confidence intervals, effect sizes, degrees of freedom and *P* value noted<br>*Give P values as exact values whenever suitable.*
☒	☐	For Bayesian analysis, information on the choice of priors and Markov chain Monte Carlo settings
☒	☐	For hierarchical and complex designs, identification of the appropriate level for tests and full reporting of outcomes
☒	☐	Estimates of effect sizes (e.g. Cohen's *d*, Pearson's *r*), indicating how they were calculated

Our web collection on statistics for biologists contains articles on many of the points above.

Software and code

Policy information about availability of computer code

Data collection	Screening and full-text review were performed using Covidence software (https://www.covidence.org/). Data extractions were performed with a custom-made Microsoft Access database (version 2305). Data are available at https://github.com/mrc-ide/epireview/.
Data analysis	Code to reproduce the published analysis is available at: https://doi.org/10.5281/zenodo.17201039. A vignette with code to reproduce the plots in the main text can be found at: https://mrc-ide.github.io/priority-pathogens/articles/pathogen_zika.html. All analysis was conducted in R (version 4.4.2). Packages used include: epireview (1.4.5), orderly2(1.99.14), prioritypathogens(https://github.com/mrc-ide/priority-pathogens/tree/zika_v2 or found here: https://doi.org/10.5281/zenodo.17201039), metafor(4.8-0), meta(8.2-1), estmeansd(1.0.1), mixdist(0.5-5), ggplot2(4.0.0), ggsci(3.2.0), sf(1.0-21), ragg(1.4.0), ggspatial(1.1.10), ggforce(0.5.0), png(0.1-8), grid(4.5.1), patchwork(1.3.2), gridExtra(2.3), readxl(1.4.5), harrypotter(2.1.1), rnaturalearth(1.1.0), rnaturalearthdata(1.0.0), ggrepel(0.9.6), gt(1.0.0), scales(1.4.0), gpkg(0.0.12), RSQLite(2.4.3), countrycode(1.6.1), ggpattern(1.2.1), tidyverse(2.0.0), stringr(1.5.2), maps(3.4.3), tidygeocoder(1.0.6).

For manuscripts utilizing custom algorithms or software that are central to the research but not yet described in published literature, software must be made available to editors and reviewers. We strongly encourage code deposition in a community repository (e.g. GitHub). See the Nature Portfolio guidelines for submitting code & software for further information.

Data

Policy information about availability of data

All manuscripts must include a data availability statement. This statement should provide the following information, where applicable:

- Accession codes, unique identifiers, or web links for publicly available datasets
- A description of any restrictions on data availability
- For clinical datasets or third party data, please ensure that the statement adheres to our policy

Data extractions were performed with a custom-made Microsoft Access database (version 2305). Data are available at https://github.com/mrc-ide/epireview/. There are no restrictions on data availability.

Research involving human participants, their data, or biological material

Policy information about studies with human participants or human data. See also policy information about sex, gender (identity/presentation), and sexual orientation and race, ethnicity and racism.

Reporting on sex and gender	We assessed the probability of Zika congenital syndrome in pregnant women, as defined by the included papers, using secondary data extracted from the included papers.
Reporting on race, ethnicity, or other socially relevant groupings	We did not extract information on race, ethnicity, or other socially relevant groupings.
Population characteristics	We extracted information about age, sex/gender (as defined in the papers), Zika positivity based on diagnostic and serological tests, and population type (e.g. children, pregnant women, hospitalized patients, population-based participants), and location (country and admin 1 unit).
Recruitment	We used secondary data extracted from papers included in our review. In our meta-analysis, we evaluated potential bias using funnel plots.
Ethics oversight	No ethics approval was required because we used only previously published secondary data.

Note that full information on the approval of the study protocol must also be provided in the manuscript.

Field-specific reporting

Please select the one below that is the best fit for your research. If you are not sure, read the appropriate sections before making your selection.

☒ Life sciences ☐ Behavioural & social sciences ☐ Ecological, evolutionary & environmental sciences

For a reference copy of the document with all sections, see nature.com/documents/nr-reporting-summary-flat.pdf

Life sciences study design

All studies must disclose on these points even when the disclosure is negative.

Sample size	No sample size calculation was performed because we used only previously published secondary data.
Data exclusions	In the main analysis, data from papers with a quality assessment score below 50% were excluded. For the meta-analysis, we used stringent criteria for inclusion, specified in the Methods section and the Extended Methods section in the Appendix. Specifically, we conducted meta-analyses for the proportion of symptomatic cases, probability of CZS and probability of pregnancy loss among confirmed ZIKV-infected mothers. For the latter two, we only included estimates with at least 10 pregnant women with confirmed ZIKV infection and with a study design that did not select for the outcome (CZS or miscarriage), using the meta R package.
Replication	We did not conduct any experiments, but all analysis can be reproduced using the code at: https://doi.org/10.5281/zenodo.17201039.
Randomization	This is not relevant to our study as we only used previously published secondary data.
Blinding	This is not relevant to our study as we only used previously published secondary data.

Reporting for specific materials, systems and methods

We require information from authors about some types of materials, experimental systems and methods used in many studies. Here, indicate whether each material, system or method listed is relevant to your study. If you are not sure if a list item applies to your research, read the appropriate section before selecting a response.

Materials & experimental systems

n/a	Involved in the study
☒	☐ Antibodies
☒	☐ Eukaryotic cell lines
☒	☐ Palaeontology and archaeology
☒	☐ Animals and other organisms
☒	☐ Clinical data
☒	☐ Dual use research of concern
☒	☐ Plants

Methods

n/a	Involved in the study
☒	☐ ChIP-seq
☒	☐ Flow cytometry
☒	☐ MRI-based neuroimaging

Plants

Seed stocks	NA
Novel plant genotypes	NA
Authentication	NA
